# Stereodivergent Synthesis of Three Contiguous Stereogenic Centers by Cu/Ir‐Catalyzed Borylallylation

**DOI:** 10.1002/anie.202523140

**Published:** 2025-12-22

**Authors:** Suman Das, Stanna K. Dorn, M. Kevin Brown

**Affiliations:** ^1^ Department of Chemistry Indiana University Bloomington 800 E. Kirkwood Ave Bloomington IN 47405 USA

**Keywords:** β‐Lycorane, Alkene difunctionalization, Carboallylation, Carboboration, Cooperative catalysis, Stereodivergent synthesis

## Abstract

Polyfunctional compounds bearing multiple stereogenic centers are synthetically valuable and important for complex molecule preparation. Herein, a Cu/Ir‐catalyzed borylallylation of electron deficient alkenes is presented. The reaction operates by the cooperative function of a chiral Cu‐complex and a chiral Ir‐complex to generate products with high levels of stereocontrol. The method also allows for the stereodivergent synthesis of acyclic products with control of three stereogenic centers. Importantly, the products contain C─B, ester, and alkene functionalities, which allows for a diverse range of products to be generated. Finally, the highly functional nature of the products has been exploited in an efficient formal synthesis of the complex natural product (+)‐(β)‐Lycorane.

## Graphical abstract

An alkene borylallylation strategy enabled by cooperative Cu/Ir‐catalysis has been uncovered for accessing three contiguous stereogenic centres. This strategy is notable given the inherent challenges of constructing molecules with multiple stereocenters while maintaining high selectivity bearing diverse functional groups. An application of the method has been showcased for the synthesis of (+)‐β‐Lycorane, with diverse range of substrates tolerance.

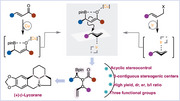

Alkene difunctionalizations are a critical class of reaction for chemical synthesis as rapid molecular complexity can be generated from simple reagents.^[^ ^1^, ^2^, ^3^ ^]^ Of the many methods that have been reported, alkene carboboration reactions are of high significance.^[^ ^4^, ^5^ ^]^ The utility of these reactions largely stems from the generation of a new C─C bond and a synthetically useful C─B bond in one step.^[^ ^6^ ^]^ Of the methods that have been developed, Cu‐catalyzed,^[^ ^7^, ^8^, ^9^, ^10^ ^]^ Cu/Pd‐catalyzed carboboration,^[^ ^11^, ^12^, ^13^, ^14^, ^15^, ^16^, ^17^, ^18^, ^19^ ^]^ and Ni‐catalyzed^[^ ^20^, ^21^, ^22^, ^23^, ^24^ ^]^ processes have been the most widely investigated (Scheme 1a). Analysis of these methods reveal that effective protocols have been developed for generation of two stereogenic centers from bond formation at the positions of the alkene (Scheme 1a). Recently, You reported a method for generation of two stereogenic centers by reaction of styrenes with Ir‐π‐allyl complexes (Scheme 1a).^[^ ^25^ ^]^ Methods that allow for control of three stereogenic centers and use secondary Csp3 electrophiles that allow for stereogenic center formation are not known. Synthesis of molecules with three or more contiguous stereogenic centers is challenging and often requires multistep approaches. Development of methods that can precisely control stereochemistry with three or more contiguous stereogenic centers would be of high value to streamline synthesis (Scheme 1b).

**Scheme 1:**
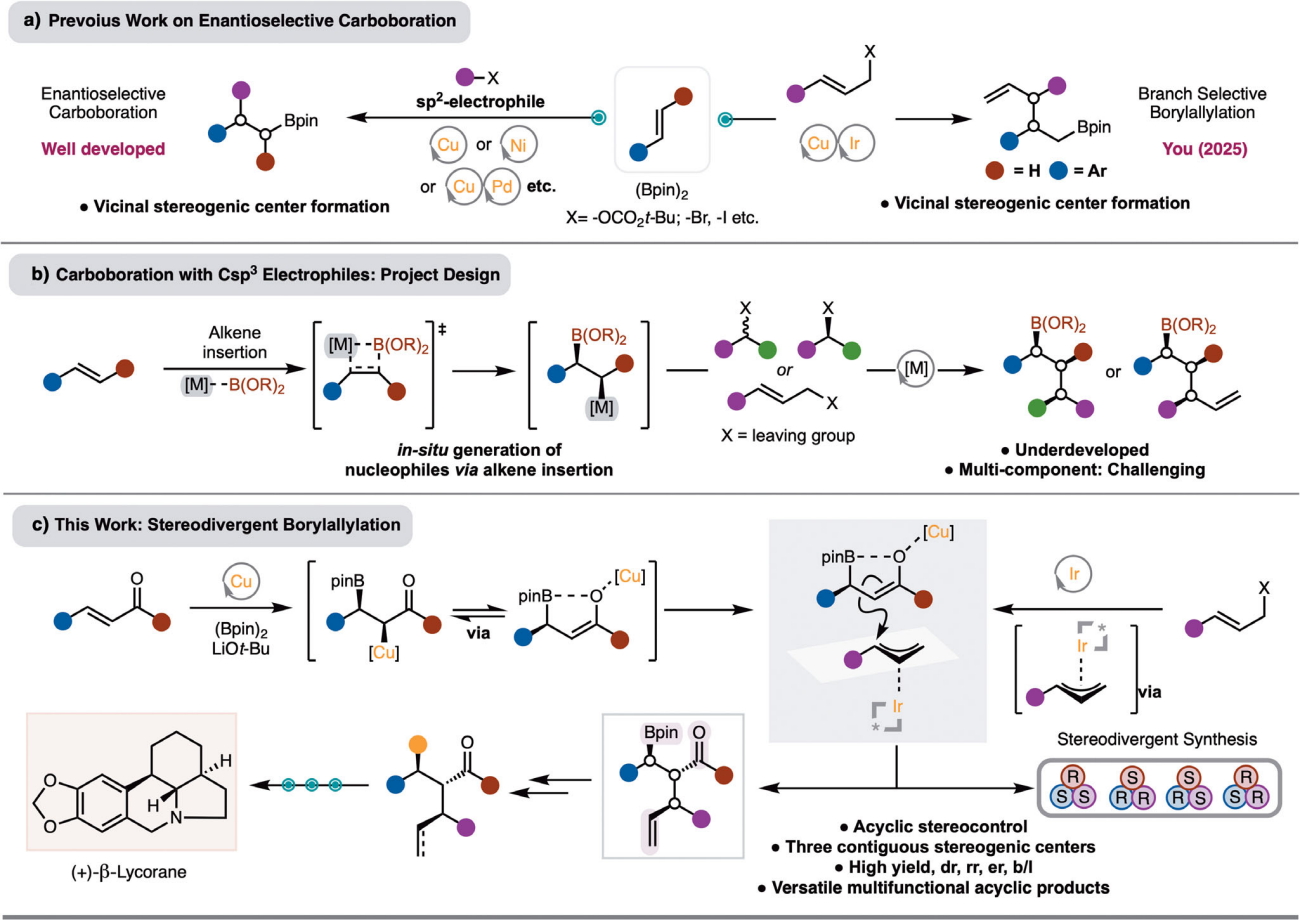
a) Previous work on enantioselective alkene carboboration. b) Alkene carboboration with alkyl electrophiles: reaction design. c) Stereodivergent alkene carboboration reactions.

To address this challenge, we elected to combine a Cu‐catalyzed alkene borylation with an Ir‐catalyzed allylation reaction (Scheme 1c). In recent years, Ir‐catalyzed allylation has been extensively developed for the enantioselective combination of either linear or branched allylic alcohols with a variety of nucleophiles to deliver the branched products.^[^ ^26^, ^27^, ^28^, ^29^, ^30^, ^31^ ^]^ In particular, (cooperative) catalytic methods with prochiral and chiral nucleophiles have been advanced.^[^ ^32^, ^33^, ^34^, ^35^, ^36^, ^37^ ^]^ There is substantial literature precedent for Ir‐catalyzed allylation with enolate nucleophiles to generated two stereogenic centers.^[^ ^38^, ^39^, ^40^, ^41^, ^42^, ^43^, ^44^, ^45^ ^]^ However, forming three contiguous stereogenic centers with control of enantioselectivity and diastereoselectivity are rare and limited to cyclic systems, and thus heavily favored to achieve *anti*‐selectivity, or acyclic systems with sterically biased substituents, which limits scope.^[^ ^46^, ^47^ ^]^

We endeavored to develop the Cu/Ir‐borylallyation of α,β‐unsaturated esters (Scheme 1c). This is important because the products would contain three different functional groups – C─B bond, alkene, and ester – that are all easily amenable to functionalization. This feature has been exploited in the efficient formal synthesis of the complex natural product Lycorane. Finally, a key goal is to achieve the synthesis of multiple stereoisomers by changing the enantiomer of chiral ligands of either Ir‐ and/or Cu‐catalysts.

Initial studies focused on reaction of ethyl cinnamate (**1**) with linear allylic alcohol derivative **2** promoted by Cu‐ and Ir‐catalysts (Scheme 2). Reactivity was probed with NHC‐Cu‐complexes due to their demonstrated high reactivity in alkene borylation reactions.^[^ ^48^, ^49^, ^50^ ^]^ Pleasingly, good reactivity was observed with SIMesCuCl and [Ir‐(*S,S,S*)]^[^ ^51^ ^]^ (Scheme 2, entry 1). Branched allylic carbonates were also evaluated, however, low yields were observed (see Supporting Information for details). Due to the achiral nature of the Cu‐complexes a 1:1 mixture of diastereomers **3** and **4** was observed; however, the chiral Ir‐complex allowed for high facial selectivity (96:4 er for formation of **3**). To achieve a diastereoselective reaction, chiral NHC─Cu complexes were explored. While moderate success was found with McQuadeCuCl (Scheme 2, entry 2),^[^ ^52^ ^]^ challenges with modification of chiral NHC complexes prompted the exploration of commercially available chiral bisphosphine ligands with Cu‐complexes. While several chiral bisphosphines were screened, an initial hit was observed with (*R,R*)‐DIOP (Scheme 2, entry 4). Notably, the use of ferrocene derived JosiPhos type ligand L_3_ allowed for synthesis of **3**/**4** 90:10 dr and >99:1 er (Scheme 1c, entry 5).^[^ ^53^, ^54^ ^]^ Continued evaluation led to the finding that ferrocene derived (*S,S*)‐Foxap L_4_ led to formation of **3** in 92:8 dr and >99:1 er (Scheme 2, entry 6).^[^ ^55^, ^56^, ^57^ ^]^ It is worth nothing that the diasteromeric (*S,R*)‐Foxap ligand led to lower levels of yield and diastereoselectivity (See Supporting Information for details). Optimization of this result with evaluation of solvent and catalyst loading led to the formation of **3** in 98:2 dr and >99:1 er (Scheme 2, entry 10). Further control experiments were carried out to demonstrate that Cu‐ and Ir‐catalysts are both necessary for the reaction to proceed. (Scheme 2, entries 11 and 12).

**Scheme 2:**
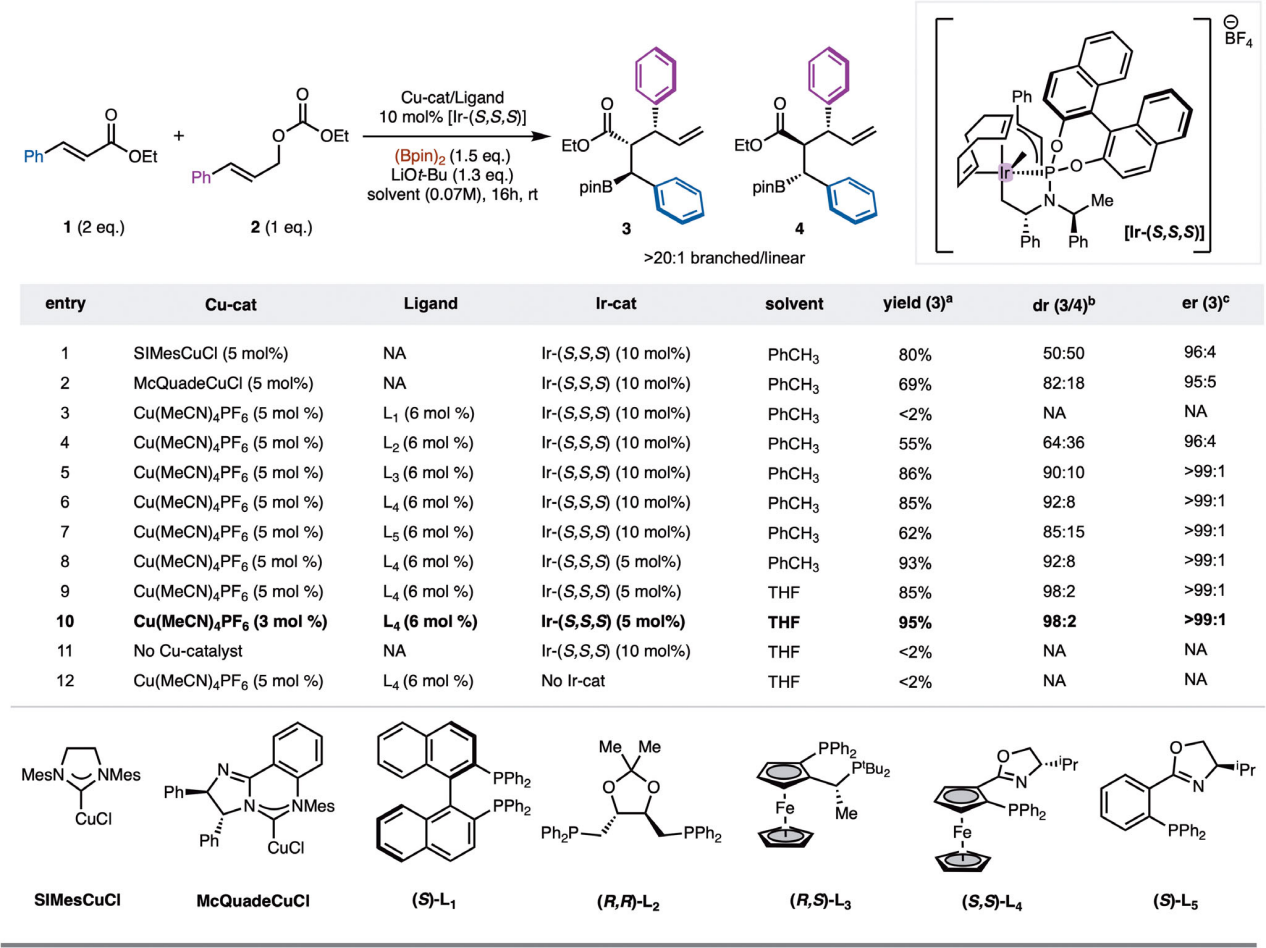
Borylallylation and reaction optimization. All reactions were performed on a 0.1 mmol scale. a) NMR yields and b/l ratio were determined analysis of the unpurified reaction mixture by ^1^H NMR using CH_2_Br_2_ as internal standard; b) dr was determined by GC.

With an optimized set of conditions in hand, the scope of the process was explored (Scheme 3). It should be noted that the products were oxidized to facilitate purification and enantiomeric ratio determination by HPLC with a chiral column. Initially, investigation of the α,β‐unsaturated ester unit was conducted. Modification of the cinnamate ester revealed that substitution with electron‐releasing (products **6**, **7**, and **11**), electron‐withdrawing (products **8** and **9**), and sterically demanding groups (products **10** and **11**) were tolerated. However, for the 2‐OMe aryl‐based substrate, slightly lower levels of diastereoselectivity were observed likely due to steric bulk of the ─OMe group. In addition, various heteroaryl groups did not impede the reaction such as thiophene (product **13**), indole (product **14**), and furan (product **15**). In the case of alkyl groups, reaction with ethyl crotonate worked well, but in slightly reduced dr (product **16**). With larger alkyl groups such as *n*‐alkyl (product **17**), or cyclohexyl (product **18**) high levels of dr were observed. Finally, α,β,γ, and δ‐unsaturated esters can be used to provide access to 1,6‐dienes (product **19**).

**Scheme 3:**
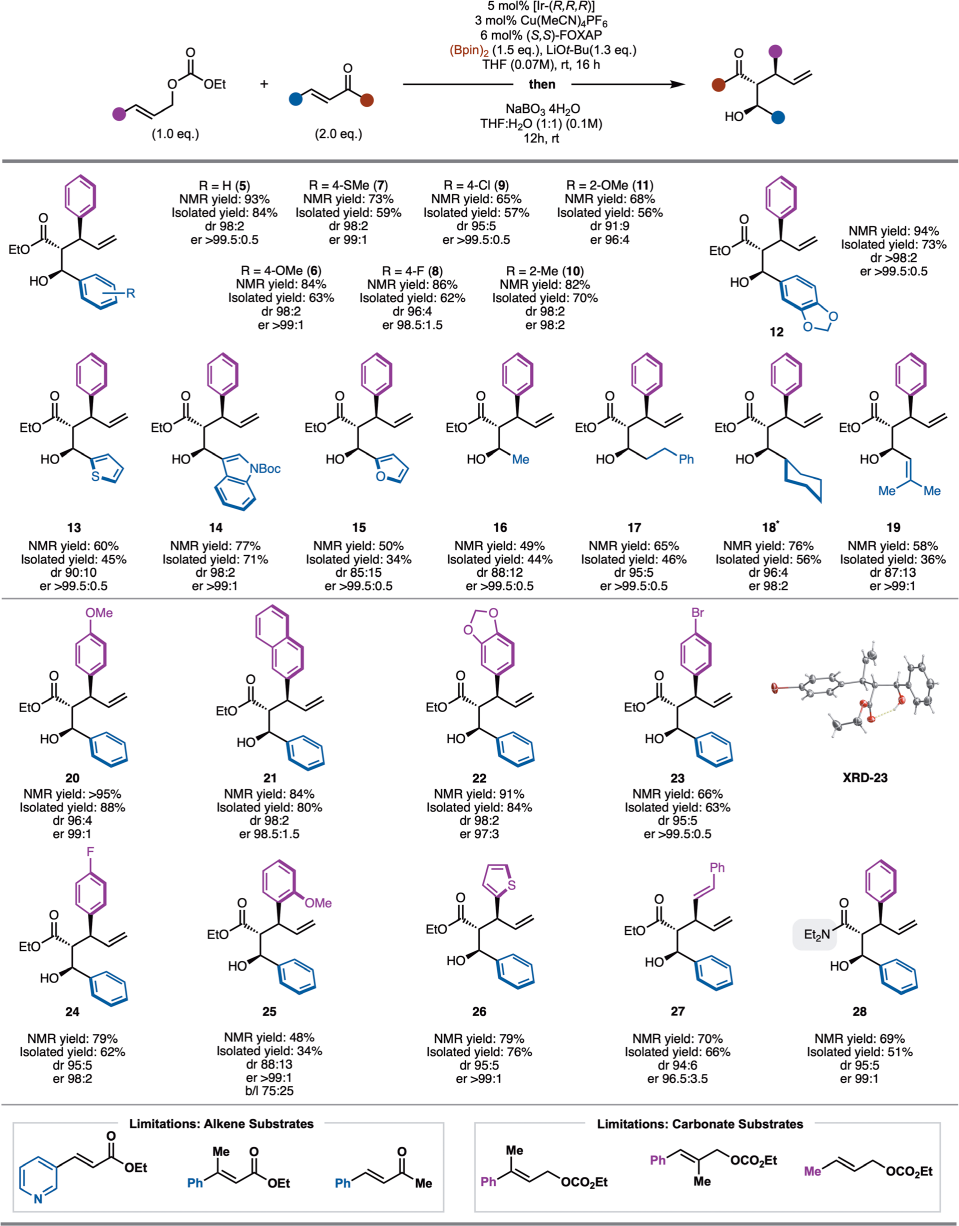
Reaction scope. All reactions were performed on a 0.1 mmol scale; yields reported as the average of two independent runs. NMR yields and dr reported from the unpurified reaction mixture prior to oxidation of the C─B bond. ^*^ Oxidation was carried out using H_2_O_2_ and 4M NaOH.

With respect to the allylic alcohol derivatives, aryl groups with electron‐donating (products **20** and **22**) and withdrawing (products **23** and **24**) function smoothly with high diastereoselectivity and enantioselectivity. For the 4‐Br aryl substrate a crystal structure was obtained to confirm the absolute stereochemistry of the products using (*S,S*)‐Foxap and [Ir‐(*R*,*R*,*R*)]. Sterically demanding substituents worked, but in reduced yield (product **25**). In this case, the linear product was observed likely due to steric pressure incurred during addition to the internal position of the Ir‐π‐allyl complex. Finally, tolerance to thiophene (product **26**) and use of dienyl allylic carbonate (product **27**) was shown. In one example, it was demonstrated that the ester is not required as an α,β‐unsaturated amide worked well (product **28**).

A proposed catalytic cycle for the process is presented in Scheme 4a. Based on literature precedence, generation of L_n_CuBpin (**I**) and borylcupration of the α,β‐unsaturated ester likely leads to formation of **II**. It is probable that the enolate coordinates with the Bpin unit to rigidify the structure (**II**).^[^ ^49^, ^50^ ^]^ At the same time, Ir‐π‐allyl complex **III** is formed by reaction of [Ir*] and the allylic carbonate. Addition of enolate (**II**) occurs from the face opposite the β‐substituent at the internal position of the Ir‐π‐allyl complex **III** to generate the observed product. Based on the proposed catalytic cycle (Scheme 4a), control of stereochemistry at C1 is dictated by the enantiomer of chiral Cu‐complex, the stereochemistry at C2 is based on substrate control, and finally, the stereochemistry at C3 is established by the enantiomer of chiral Ir‐catalyst. Therefore, of the possible eight stereoisomers that could be formed, four should be accessible through various combination of the chiral catalysts. This is demonstrated in Scheme 4b. With either [Ir‐(*R*,*R*,*R*)] or [Ir‐(*S*,*S*,*S*)] and either [Cu‐(*R*,*R*)‐L_4_] or [Cu‐(*S*,*S*)‐L_4_] the four stereoisomers **3**–**5**, **29** can be prepared in high yield, diastereoselectivity, and enantioselectivity. It does not appear that a significant matched or mismatched effect between chiral catalysts was observed (Scheme 4b).

**Scheme 4:**
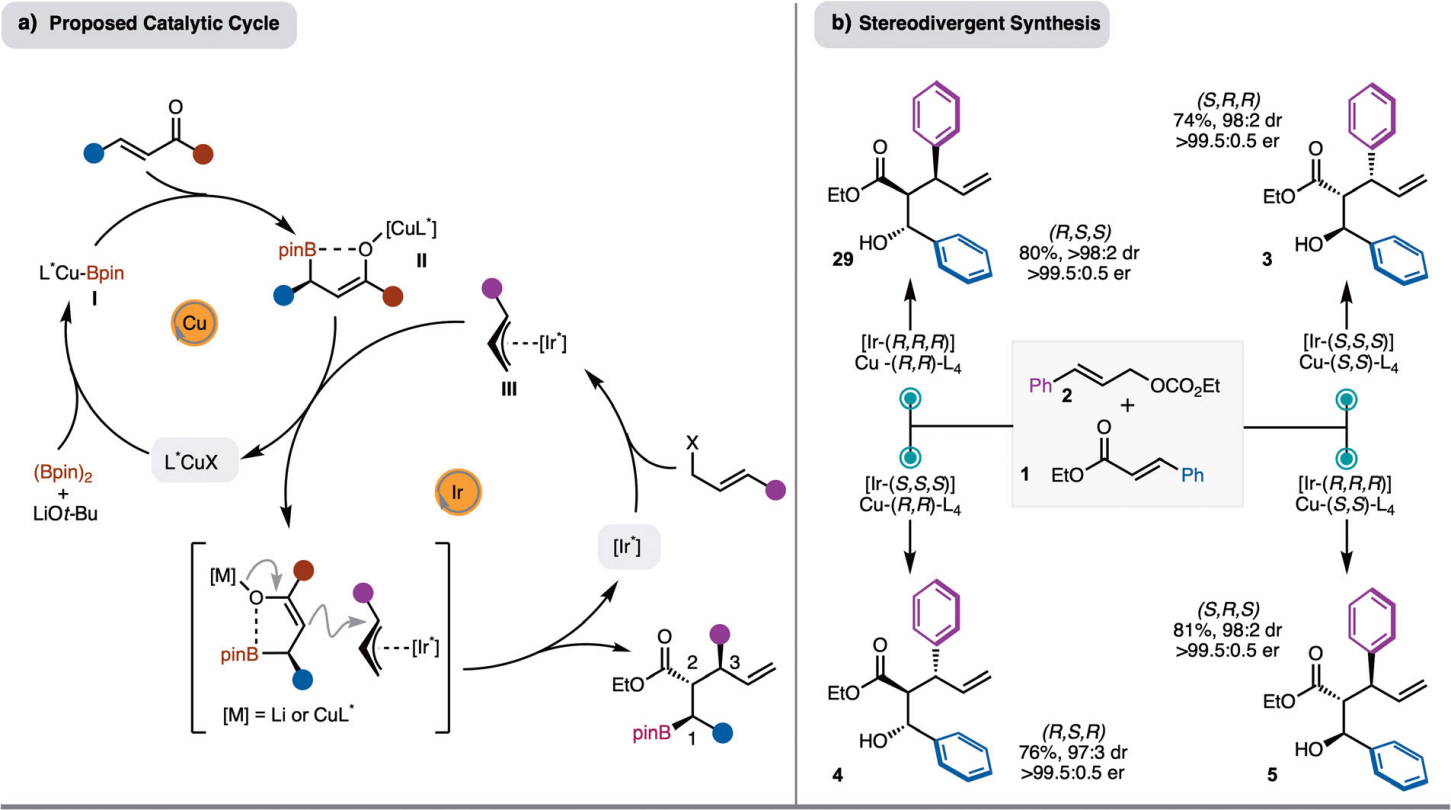
a) Proposed catalytic cycle. b) Stereodivergent synthesis.

A scale up synthesis was conducted on a 1.0 mmol scale between cyclohexyl substituted α,β‐unsaturated ester and allylic carbonate **2** to achieve the allyl‐borylated product **30** in 56% yield, >95:5 dr, and >98:2 er (Scheme 5a). Later, further transformation of the products was carried out (Scheme 5a). Based on recent developments from the Morken group, stereospecific Cu‐catalyzed benzoylation and alkynylation can be carried out (products **31** and **32**) with high yields and no erosion of diastereoselectivity.^[^ ^58^ ^]^ In addition to oxidation of the C─B bond (see Scheme 2), Cu‐catalyzed amination is also effective (product **33**). Additional stereospecific transformation of the C─B bond by Evans–Zweifel alkenylation (product **34**)^[^ ^59^, ^60^ ^]^ and Matteson homologation with dibromomethane worked smoothly (product **35**).^[^ ^61^ ^]^ Finally, reduction of the ester leads to chiral 1,3‐diol **36** and Ir‐catalyzed hydroboration of the alkene allows for synthesis of 1,5‐diborylated compound **37**.^[^ ^62^ ^]^

**Scheme 5:**
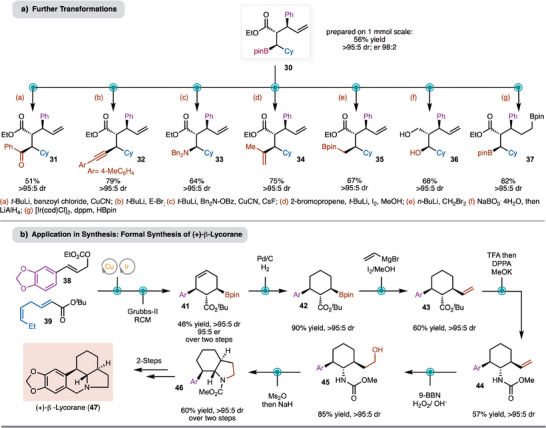
a) Further transformations b) Application in complex molecule synthesis.

It is important to note that due to the presence of three distinct functional groups within the direct products of borylallylation, independent and facile modification can be carried out to arrive at a diverse range of structures. In this regard, a formal synthesis of (+)‐(β)‐Lycorane is presented here utilizing this methodology as a key step (Scheme 5b). The synthesis commenced with Cu/Ir‐catalyzed allylboration of allyl carbonate **38** and α,β‐unsaturated ester **39** to obtain the corresponding product in 65%–69% NMR yield and >95:5 dr (er 95:5 after oxidation of C─Bpin; details in Supporting Information). This product was directly subjected to a ring‐closing metathesis (RCM) to achieve cyclohexene derived product (**41**) in 46% overall yield and >95:5 dr. Subsequent, hydrogenation allowed for synthesis of cyclohexane **42** (>95% yield; >95:5 dr). Zweifel alkynylation of the secondary boronate ester led to installation of the vinyl group in product **43**. Treatment with TFA resulted in formation of the corresponding carboxylic acid, which was subjected to DPPA to induce a Curtius rearrangement to provide carbamate **44**. Hydroboration–oxidation of **44** resulted in formation of the corresponding alcohol **45**, which upon treatment with Ms_2_O then NaH led to formation of carbamate **46**. This intermediate was previously reported by Yang et al. in their total synthesis of (+)‐(β)‐Lycorane (**47**)**.^[^** ^63^ ^]^

In conclusion, we have developed a stereodivergent process for the Cu/Ir‐catalyzed borylallylation of unsaturated esters. The method allows for the stereodivergent synthesis of three contiguous stereogenic centers. In addition, the products are of high utility due to the presence of three distinct functional groups, which has allowed for the concise enantioselective synthesis of the complex natural product Lycorane. Further advancement of the Cu‐catalyzed borylation reactions in the area of cooperative catalysis are underway.

## Supporting Information

The authors have cited additional references within the Supporting Information.^[^ ^30^, ^31^ ^]^

## Conflict of Interests

The authors declare no conflict of interest.

## Supporting information



Supporting Information

Supporting Information

## Data Availability

The data that support the findings of this study are available in the supplementary material of this article.
