# Supplementary material for: Stereodivergent Synthesis of Three Contiguous Stereogenic Centers by Cu/Ir‐Catalyzed Borylallylation
Source: Angew Chem Int Ed Engl. 2025 Dec 22;65(8):e23140. doi: 10.1002/anie.202523140 (PMC12802513; doi:10.1002/anie.202523140)
Supplement: Supplementary file 1 — Supporting Information [file ANIE-65-e23140-s001.pdf]

Stereodivergent Synthesis of Three Contiguous Stereogenic Centers by Cu/Ir-Catalyzed Borylallylation

Suman Das, Stanna K. Dorn and M. Kevin Brown*

† Department of Chemistry, Indiana University, 800 E. Kirkwood Ave, Bloomington, IN 47405, United States

Table of contents

1. General information 02

2. Reagents 02

3. General procedure for the synthesis of alkenes 05

4. General procedure for the synthesis of allylic carbonates 07

5. Synthesis of (*R,R*) and (*S,S*)-FOXAP ligand and Ir-complex 10

6. General procedure for the Cu/Ir-cooperative borylallylation reaction 12

7. Optimization of the reaction 15

8. Experimental characterization data 21

9. Stereodivergent synthesis 35

10. Scale-up synthesis and Further functionalization 39

11. Formal Synthesis of (+)-(β)-Lycorane 47

12. Crystal structure: absolute configuration of product 53

13. NMR data 69

14. HPLC data 115

15. Reference 166

1. General Information:

Infrared (IR) spectra were recorded on Bruker Tensor II FT-IR Spectrometer, n_{max} in cm-1. Bands are characterized as broad (br), strong (s) for >70% transmittance, medium (m) for 40-70% transmittance, and weak (w) for <40% transmittance. ^{1}H NMR spectra were recorded at room temperature on a Varian I400 (400 MHz), Varian VXR400 (400 MHz), Varian I500 (500 MHz), a Varian I600 (600 MHz) spectrometer, and/or a Bruker Ascend™ 500 MHz (equipped with cryoprobe). Chemical shifts are reported in ppm from tetramethyl silane with the solvent resonance as the internal standard ($CHCl_3$: λ 7.26 ppm). Data are reported as follows: chemical shift, multiplicity (s = singlet, d = doublet, t = triplet, q = quartet, br = broad, m = multiplet, app. = apparent), coupling constants (Hz), and integration. ^{13}C NMR spectra were recorded on a Varian I400 (100 MHz), Varian I500 (125 MHz), and/or a Bruker Ascend™ 500 MHz (125 MHz, equipped with cryoprobe) spectrometer with complete proton decoupling. Chemical shifts are reported in ppm from tetramethyl silane with the solvent resonance as the internal standard ($CDCl_3$: δ77.16 ppm). Unless otherwise noted, all reactions have been carried out with distilled and degassed solvents under an atmosphere of dry N_2 in oven- (135 °C) and flame-dried glassware with standard vacuum-line techniques. Tetrahydrofuran (THF) was purified under a positive pressure of dry argon by passage through two columns of activated alumina. Toluene was purified under a positive pressure of dry argon by passage through columns of activated alumina and Q5 (Grubbs apparatus). All work-up and purification procedures were carried out with reagent grade solvents (purchased from Sigma-Aldrich) in air. Standard column chromatography techniques were carried out using ZEO prep 60/40-63 µm silica gel. For samples that were unstable on silica, purification was done using neutral aluminum oxide (activated, Brockman I 58 Å pore size, Oakwood). For difficult separations, medium-pressure liquid chromatography (MPLC) was performed using a Teledyne ISCO Combi Flash Rf 150 instrument. Optical rotations were measured on a PerkinElmer 241 polarimeter at 589 nm wavelength (sodium D-line) using a standard 10 cm cell (1 mL). Specific rotations (if reported), $[\alpha]_D^{20}$, are reported in degree mL/(g·dm) at the specific temperature. Concentrations (c) are given in grams per 100 mL of the specific solvent. Chiral HPLC analysis was performed on an Agilent 1220 Infinity LC system.

2. Reagents:

***(E)*-Ethyl cinnamate** was purchased from Sigma Aldrich and used as received.
(*R*)-(+)-1,1'-bi-2-naphthol was purchased from CombiBlocks and used as received.
Bis(pinacolato)diboron was purchased from Oakwood Chemicals and recrystallized from pentane prior to use.
4-Phenyl-3-buten-2-one was purchased from Ambeed and used as received.
***O*-benzoyl-*N*,*N*-dibenzylhydroxylamine** was purchased from Ambeed and used as received.
CuCN was purchased from Sigma Aldrich and used as received.
Styrene was purchased from Sigma Aldrich and used as received.
Benzoyl chloride was purchased from Sigma Aldrich and used as received.
1-(bromoethynyl)-4-methylbenzene was prepared according to literature procedures.
***n*-butyllithium (2.5 M in hexanes)** was purchased from Sigma Aldrich and used as received.
***t*-butyllithium (2.5 M in hexanes)** was purchased from Sigma Aldrich and used as received.
Calcium hydride (CaH_2) was purchased from Oakwood or VWR (Bean Town Chemical) and used as received.

(tert-Butoxycarbonylmethylene) triphenylphosphorane was purchased from Ambeed and used as received.
(*S,S*)-FOXAP was purchased from Strem and used as received.
trans-2,4-Hexadien-1-ol was purchased from Acros organics and used as received.
DIBAL-H was purchased from Sigma Aldrich and used as received.
triethyl phosphono acetate was purchased from Oakwood and used as received.
Ethyl chloroformate was purchased from Sigma Aldrich and used as received.
Methyl chloroformate was purchased from Sigma Aldrich and used as received.
Sodium Borohydride was purchased from Oakwood and used as received.
Dess-Martin periodinane was purchased from Oakwood and used as received.
cis-3-Hexen-1-ol was purchased from Oakwood and used as received.
N-Bromo succinimide was purchased from Oakwood and used after recrystallization in water.
Pinacolborane (HBpin) was purchased from Oakwood and used as received.
Bis(diphenylphosphino)methane was purchased from Strem and used as received.
$LiAlH_4$ was purchased from Sigma Aldrich and used as received.
Grubbs gen-II was purchased from Sigma Aldrich and used as received.
Chloro-1,5-cyclooctadiene iridium(I) dimer was purchased from Ambeed and used as received.
Chloro-1,5-cyclooctene iridium(I) dimer was purchased from Strem and used as received.
Cinnamyl alcohol was purchased from Sigma Aldrich and used as received.
CuCl (99.99%) was purchased from Sigma-Aldrich and purified by washing with 1M HCl (3 x 3 mL), ethanol (3 x 3 mL), and Et_2O (3 x 3 mL) and dried in vacuo before use.
(1,5-cyclooctadiene)-(methoxy)iridium(I) dimer was purchased from TCI and used as received.
Di-µ-chlorotetraethylene dirhodium(I) was purchased from Strem and used as received.
4-dimethylaminopyridine (DMAP) was purchased from Oakwood and used as received.
(*S*)-(+)-(2,6-dimethyl-3,5-dioxa-4-phospha-cyclohepta[2,1-a;3,4-a']dinaphthalen-4-yl)dimethylamine was purchased from Strem and used as received.
(*3aR,8aR*)-(-)-(2,2-dimethyl-4,4,8,8-tetraphenyl-tetrahydro-[1,3]dioxolo[4,5-e][1,3,2]dioxaphosphin-6-yl)dimethylamine was purchased from Strem and used as received.
(*S*)-4-(dinaphtho[2,1-d:1',2'-f] [1,3,2] dioxaphosphepin-4-yl) morpholine (S)-MorfPhos was purchased from Strem and used as received.
(*S*)-(+)-(3,5-dioxa-4-phospha-cyclohepta[2,1-a;3,4-a’] dinaphthalen-4- yl) benzyl(methyl)amine was purchased from Strem and used as received.
(*S*)-(+)-(3,5-dioxa-4-phospha-cyclohepta[2,1-a;3,4-a'‘] dinaphthalen-4-yl) bis[(1S)-1-phenylethyl amine was purchased from Strem and used as received.
(*R*)-(+)-N-(3,5-dioxa-4-phosphacyclohepta[2,1-a;3,4-a′] dinaphthalen-4-yl)-dibenzo[b,f]azepine was prepared in accordance with literature procedures.
(*S*)-(+)-N-(3,5-dioxa-4-phosphacyclohepta[2,1-a;3,4-a′] dinaphthalen-4-yl)-dibenzo[b,f]azepine was purchased from Ambeed and used as received.
(*S*)-(+) -(3,5-dioxa-4-phospha-cyclohepta[2,1-a;3,4-a’] dinaphthalen-4-yl) dimethylamine (*S*)- MonoPhos was purchased from Strem and used as received.
Di-tert-butyl dicarbonate (Boc anhydride) was purchased from Oakwood and used as received.
Ethyl acrylate was purchased from TCI and purified via neat filtration through a 2 cm pad of dry silica in a 5.75-inch pipette, and vigorously sparged with nitrogen for at least 30 minutes prior to use.
Phosphorus trichloride was purchased from Alfa Aesar and used as received.
Pyridine was purchased from Macron and used after distillation over CaH_2.

Cu-McQuade catalyst prepared using literature procedure.[1]
Lithium tert-butoxide was purchased from Strem and used as received.
((*2R,3R*)-6-mesityl-2,3-diphenyl-2,3,5,6-tetrahydroimidazo[1,2-c] quinazolin-5-yl) copper(I) chloride was prepared in accordance with literature procedures.
1-phenylprop-2-en-1-ol was prepared according to literature procedures.
Phosphorus (III) chloride was purchased from Alfa Aesar and used as received.
Potassium tert-butoxide was purchased from Strem and used as received. Potassium
SIMesCuCl was prepared in accordance with literature procedures.
Sodium hydride (60% dispersion in mineral oil) was purchased from Sigma Aldrich and used as received.
cinnamyl ethyl carbonate was prepared in accordance with literature procedures.
***trans*-3-Phenyl-2-propen-1-ol** was purchased from Sigma Aldrich and used as received.
DIBAL-H solution 1M was purchased from Sigma Aldrich and used as received.
Methyl chlorocarbonate was purchased from Sigma Aldrich and used as received.
Ethyl chloroformate was purchased from Sigma Aldrich and used as received.
[Ir-(R,R,R)]- catalyst and [Ir-(S,S,S)]- catalyst was prepared in accordance with literature procedures.[2]
Sodium perborate tetrahydrate ($NaBO_3 \cdot 4H_2O$) was purchased from Merck and used as received.
30% H_2O_2 in H_2O was purchased from Macron and used as received.
Sodium sulfate (anhydrous) was purchased from VWR and used as received.
Lithium *tert*-butoxide was purchased from Strem and used as received.
Triethylamine was purchased from EMD Millipore and distilled over CaH_2 prior to use.
Vinyl magnesium bromide (1.0 M in THF) was purchased from Sigma Aldrich and used as received.

3. **General Procedure for the synthesis of alkene substrates:**

The substrates were synthesized according to the literature reported procedure with some modifications:

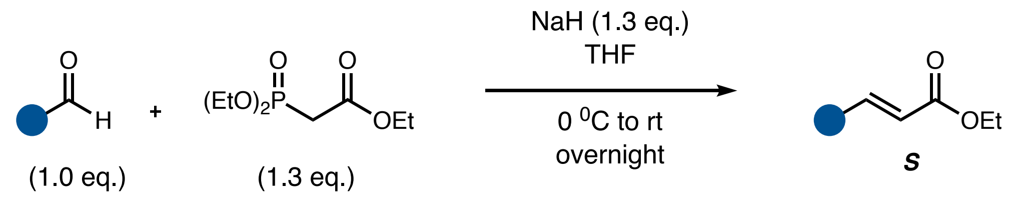

Sodium hydride in 60% mineral oil (0.36 g, 1.50 eq., 15.0 mmol) was dissolved in THF (47.0 mL), and triethyl phosphonoacetate (1.20 mL, 1.20 eq., 6.0 mmol) was added dropwise to the suspension at 0 °C. The mixture was stirred until gas evolution had ceased. Then, the aldehyde or ketone (1.0 eq., 10.0 mmol) in THF (3.0 mL) was added by syringe. The reaction was stirred at room temperature and monitored by TLC. The reaction mixture was quenched with saturated aqueous

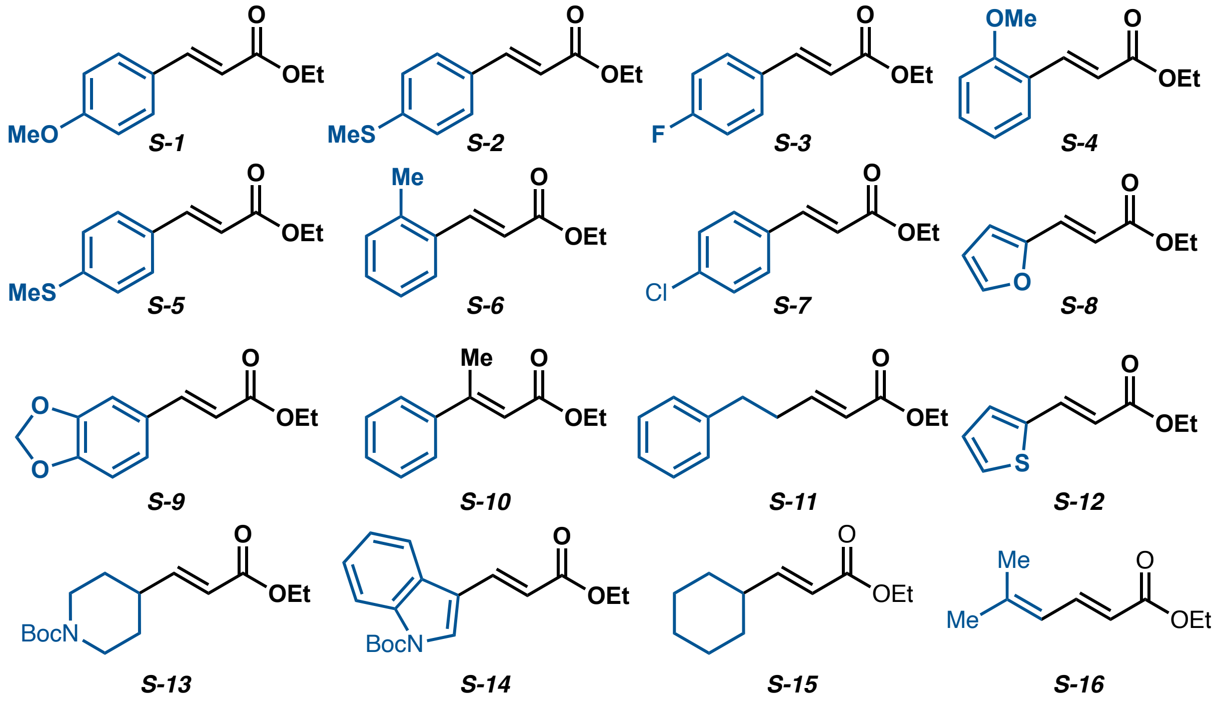

NH_4Cl solution. The organic phase was separated, and the aqueous layer was extracted with EtOAc. The combined organic phases were washed with saturated brine solution, dried over anhydrous sodium sulfate, and concentrated under vacuum pressure. Purification by silica gel

chromatography (Hexane: EtOAC=40:1 to 10:1) gave the alkene as an oil or solid. The product NMR were matched with following literature data

S-1& S-2*[3]*; S-3*[4]*; S-4*[5]*; S-5*[6]*; S-6*[7]*, S-7*[8]*, S-8 &S-12*[9]*, S-9*[10]*, S-10*[11]*, S-11*[12]*, S-13*[13]*, S-14*[14]*, S-15 & S-16*[15]*.

Synthesis of α,β-unsaturated *t*-butyl ester (*S-17*):

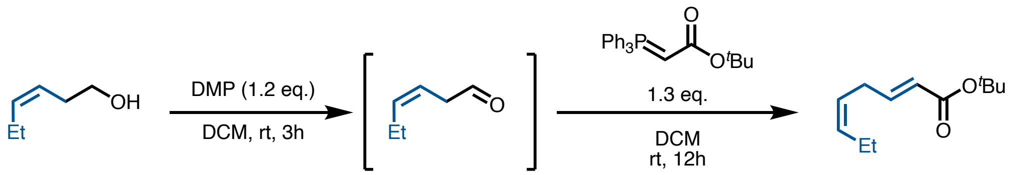

***tert-butyl (2E,5Z)-octa-2,5-dienoate* (*S-17*)**

To a solution of homoallylic alcohol (1 eq.) in DCM (0.4 M) at 0 °C was added Dess-Martin periodinane (1.2 eq.) portion wise. The reaction mixture was stirred for 3 hours at room temperature. After that the reaction quenched by the addition of saturated aqueous $Na_2S_2O_3$ and washing with saturated aqueous $Na_2S_2O_3$, then saturated aqueous $NaHCO_3$, and brine, the organic layer was dried over anhydrous sodium sulfate and concentrated under reduced pressure to afford the corresponding alcohol which was used in the next step without purification. ***(During work-up mak sure that the solid floating in the funnel should be removed before the next step to avoid lower yield)

The aldehyde (1 eq.) from last step was dissolved in anhydrous DCM and added to the tert-butyl 2-(triphenyl-l5-phosphaneylidene) acetate (1.2 eq.) in DCM (0.2 M). The reaction mixture was stirred overnight at room temperature and then solvent was evaporated. The product was obtained after doing flash column chromatography on silica gel as light-yellow oil, 52% yield (unoptimized) over two steps.

^{1}H NMR (500 MHz, $CDCl_3$) δ 6.84 (dt, *J* = 15.6, 6.3 Hz, 1H), 5.75 (dt, *J* = 15.6, 1.8 Hz, 1H), 5.57 – 5.48 (m, 1H), 5.39 – 5.30 (m, 1H), 2.91 (ddt, *J* = 7.8, 6.6, 1.7 Hz, 2H), 2.04 (pd, *J* = 7.5, 1.6 Hz, 2H), 1.60 – 1.41 (m, 9H), 1.24 – 0.67 (m, 3H).
^{13}C NMR (126 MHz, $CDCl_3$) δ 166.1, 146.0, 134.3, 123.8, 123.1, 80.1, 29.7, 28.2, 20.5, 14.1
HRMS (EI): Calculated for $C_{22}H_{26}O_3Na$ [M+]:196.1458, Found: 196.1460

4. **General Procedure for the synthesis of electrophile substrates:**

The substrates were synthesized according to the literature reported procedure with some modifications:

Method: A

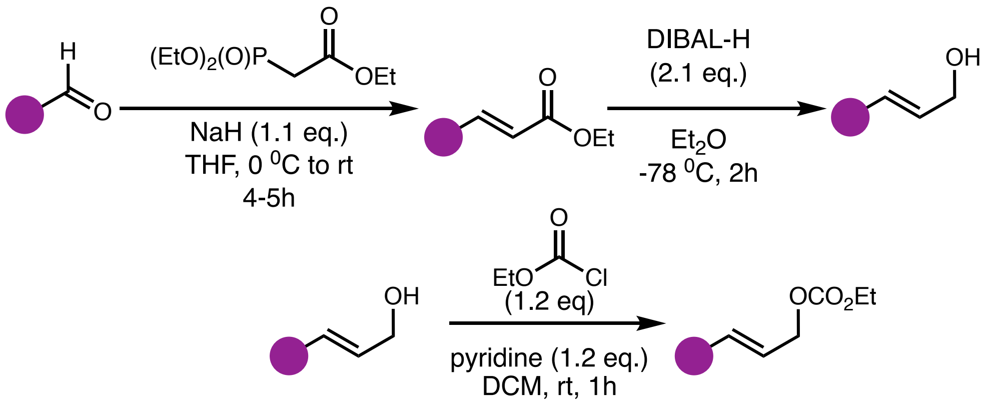

Step:1

Sodium hydride (0.36 g, 1.5 eq., 15 mmol) was dissolved in THF (47 mL), and triethyl phosphonoacetate (1.2 mL, 1.2 eq., 6.0 mmol) was added dropwise to the suspension at 0 °C. The mixture was stirred until gas evolution had ceased. Then, the aldehyde or ketone (1.0 eq., 10 mmol) in THF (3.0 mL) was added by syringe. The reaction was stirred at room temperature and monitored by TLC. The reaction mixture was quenched with saturated aqueous NH_4Cl solution. The organic phase was separated, and the aqueous layer was extracted with EtOAc. The combined organic phases were washed with saturated aqueous brine solution, dried over anhydrous sodium sulfate, and concentrated under vacuum pressure. Purification by silica gel chromatography (Hexane: EtOAC = 40:1 to 10:1) gave the alkene as an oil or solid. The product NMR were matched with following literature data

Step:2

To a solution of the above obtained α,β-unsaturated ester (1.0 eq.) in Et_2O (0.2 M) at -78 °C was added dropwise DIBAL-H (1.0 M, 2.1 eq.) and the reaction mixture was stirred until complete consumption of the α,β-unsaturated ester (~2 h). The reaction was then allowed to warm to rt and a sat. aqueous solution of potassium sodium tartrate (Rochelle salt) was added, and the reaction mixture was stirred for 1 h. The aqueous layer was then extracted with Et_2O (3x15 mL), and the combined organic layers were washed with brine, dried over sodium sulfate and concentrated under reduced pressure. The crude allylic alcohol was used in the next step without further purification.

Step:3

To a solution of the allylic alcohol (1.0 eq.) in DCM (0.4 M) at 0 °C was added pyridine (1.1 eq. and the reaction mixture was stirred for 15 min. Ethyl chloroformate (1.1 eq.) was then added dropwise, the reaction mixture was allowed to warm to rt and stirred until complete consumption of the starting material (~1 h). The reaction mixture was washed with sat. aqueous NH_4Cl, brine, dried over sodium sulfate and concentrated under reduced pressure. The crude product was purified by column chromatography with (Hexane: EtOAC=40:1 to 10:1) as eluent to afford the desired allylic carbonate. The characterization data were then matched with data reported in literature.

Method: B

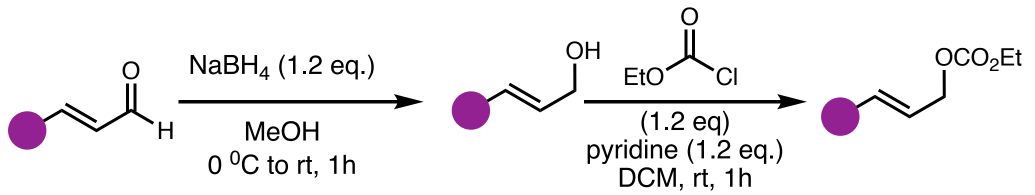

Step:1
To flame dried100 mL round-bottom flask were added α,β-unsaturated aldehyde (20 mmol, 1.0 eq.), MeOH (30 mL), and $NaBH_4$ (20.4 mmol, 1.2 eq.) at 0 °C. After stirring for 5 min, the ice bath was removed, and the resulting mixture was stirred for additional 2h at rt. After starting material was consumed completely monitoring by TLC, the reaction was quenched with H_2O (30 mL) and extracted with DCM (50 mL × 3). The combined organic layer was dried and purified by flash chromatography on silica gel to afford allylic alcohol (eluent: Hexane/ethyl acetate = 20/1 to 10/1 to 5/1): as an oil, which was used for next step directly.

Step:2
To a solution of the allylic alcohol (1.0 eq.) in DCM (0.4 M) at 0 °C was added pyridine (1.1 eq. and the reaction mixture was stirred for 15 min. Ethyl chloroformate (1.1 eq.) was then added dropwise, the reaction mixture was allowed to warm to rt and stirred until complete consumption of the starting material (~1 h). The reaction mixture was washed with sat. aqueous NH_4Cl, brine, dried over sodium sulfate and concentrated under reduced pressure. The crude product was purified by column chromatography with (Hexane: EtOAC=40:1 to 10:1) as eluent to afford the desired allylic carbonate. The characterization data were then matched with data reported in literature.

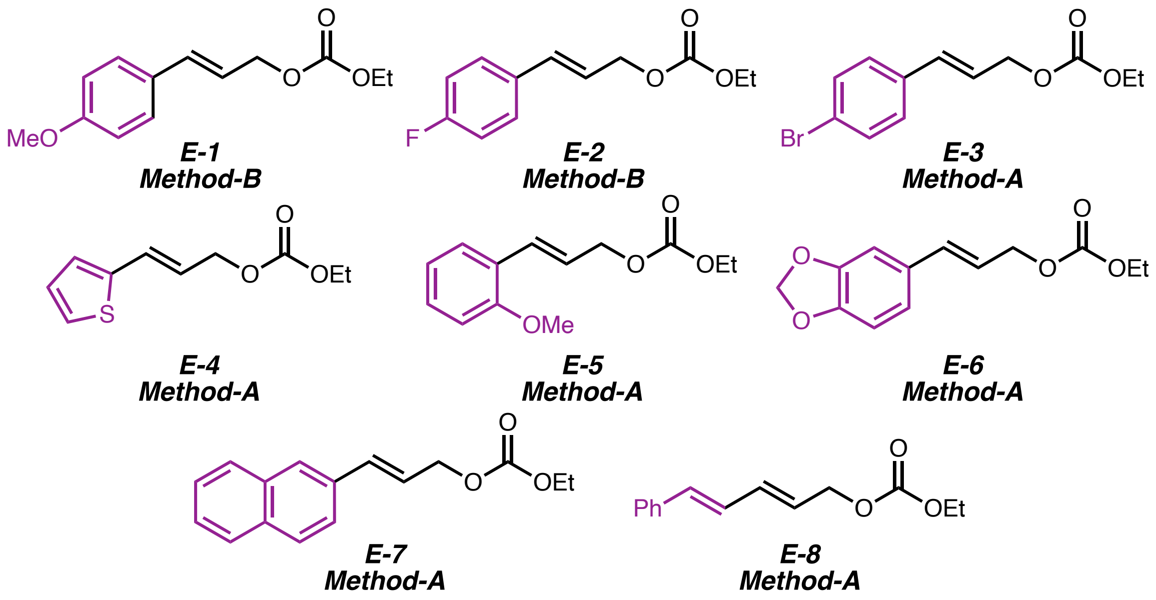

E-1, E-3, E-3, E-5, E-6, E-7, E-8 synthesized by literature procedure.[16] and ***E-4*** was synthesized by literature procedure.[17]

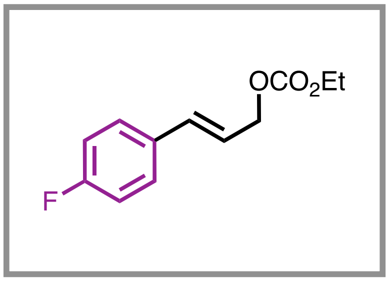

(E)-ethyl (3-(4-fluorophenyl)allyl) carbonate (E-9)

The title compound was prepared using Method-A as colorless oil with 95% isolated yield. The product was purified by silica-gel flash column chromatography (2-3% Ethyl acetate: Hexane; R_f= 0.5 in 10% Ethyl acetate: Hexane).

^{1}H NMR (500 MHz, $CDCl_3$) δ 7.39 – 7.32 (m, 2H), 7.05 – 6.97 (m, 2H), 6.65 (dd, *J* = 15.9, 1.5 Hz, 1H), 6.21 (dt, *J* = 15.8, 6.4 Hz, 1H), 4.77 (dd, *J* = 6.4, 1.4 Hz, 2H), 4.22 (q, *J* = 7.1 Hz, 2H), 1.32 (t, *J* = 7.1 Hz, 3H).

^{13}C NMR (126 MHz, $CDCl_3$) δ 162.8 (d, *J* = 247.7 Hz), 155.2, 133.7, 132.4 (d, *J* = 3.3 Hz), 128.4 (d, *J* = 8.1 Hz), 122.5 (d, *J* = 2.3 Hz), 115.7 (d, *J* = 21.7 Hz), 68.2, 64.3, 14.4

^{19}F NMR (471 MHz, $CDCl_3$) δ -113.51 (q, *J* = 4.3 Hz)

HRMS (EI): Calculated for $C_{22}H_{26}O_3Na$ [M+]:224.0843, Found: 224.0846

5. (a) General Procedure for (S,S)-FOXAP and (R,R)-FOXAP:

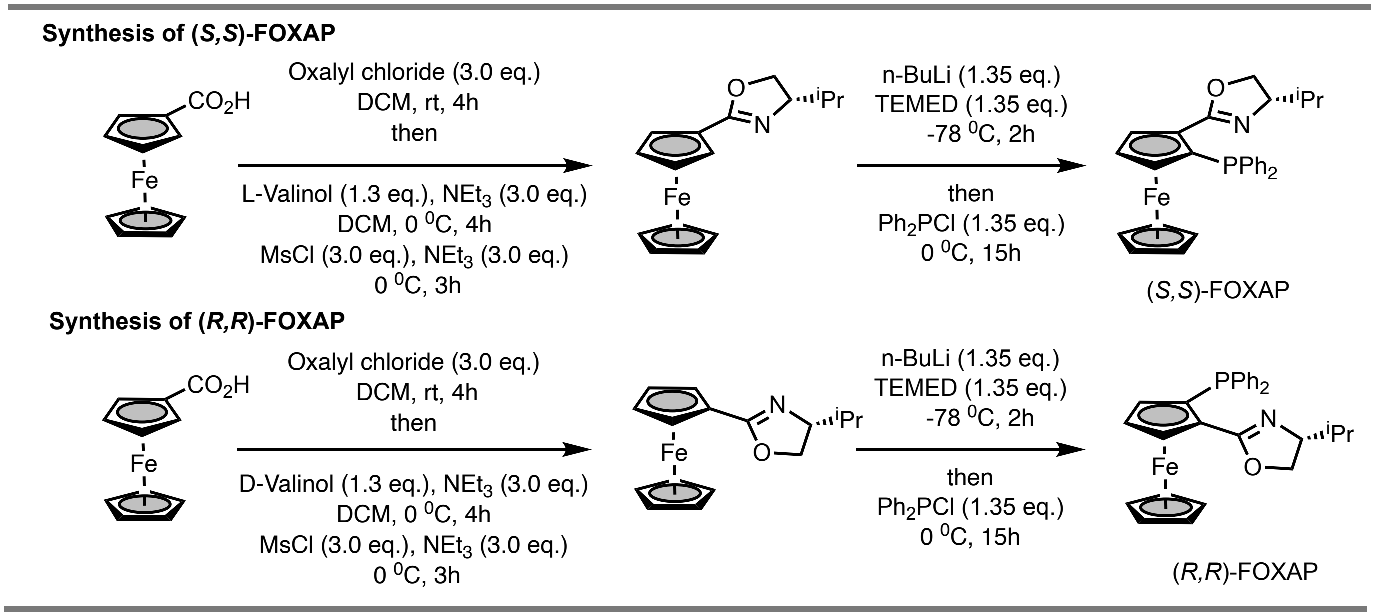

Step:1

In a flame dried 250 round-bottom flask was charged magnetic stir bar was added Ferrocenecarboxaldehyde (2.0 g, 1 eq., 8.7 mmol) in dry DCM (60 mL) and Oxalyl chloride (2.3 mL, 3 eq., 26.1 mmol) and stirred for 4 hours at room temperature under an inert atmosphere. Then the solvent was removed under reduced pressure, to get a red solid. The solid was then dissolved in dry DCM (40 mL) and slowly added to a stirred mixture of L-valinol or D-valinol (1.17 g, 1.3 eq., 11.3 mmol), Triethylamine (3.6 mL, 3 eq., 26.1 mmol) in DCM (40 mL) at 0 °C under an inert atmosphere. Stirring was continued for 4 hours, after which Methanesulfonyl chloride (1.35 mL, 2 eq., 17.4 mmol) and Triethylamine (3.6 mL, 3 eq., 26.1 mmol) were added, with stirring continued for a further 3 hours. Then saturated $NaHCO_3$ (75 ml) and DCM (75 ml) were added to the flask, and the layers separated. The aqueous layer was extracted with further portions of DCM (2 x 75 ml), the organic layers combined, dried over anhydrous sodium sulfate and the solvent removed under reduced pressure. Further purification was done using silica gel column chromatography afforded a deep red crystalline solid (~90-93% yield). The NMR data further matched with the known compound reported in literature.[18]

Step:2

To a solution of (*S*) or (*R*)-Oxazole (2.67 g, 1 eq., 8.9 mmol) in Diethyl ether (103 mL) was added TEMED (1.8 mL, 1.35 eq., 12.13 mmol), the solution was cooled to -78 °C before the addition of n-Butyllithium (8.7 mL, 1.4 M, 1.35 eq., 12.1 mmol) over a period of 2 minutes. The reaction was stirred at -78 °C for 2 hours, the cooling bath was exchanged for 0 °C bath for 15 min before the addition of Chlorodiphenylphosphine (2.3 mL, 1.35 eq., 12.1 mmol). Then the cooling bath was removed and after 15 minutes the reaction was quenched with saturated $NaHCO_3$ (45 mL, de-gassed), the mixture was left at room temperature under N_2 for 5h. The reaction mixture was

diluted with DCM (45 mL) the aqueous phase separated and extracted with DCM (45 mL). The combined organics were washed with saturated $NaHCO_3$ (50 mL), water (45 mL) and brine (50 mL) then dried over anhydrous sodium sulfate, and the solvent removed under reduced pressure. Further purification was done using silica gel column chromatography afforded the FOXAP ligand as orange or yellow color 35%-38% yield. The analytical data was matched with the literature.[19]

(b) General procedure for synthesis of [Ir-(*S,S,S*)] and [Ir-(*R,R,R*)] complex:

[Ir-(*S,S,S*)] and [Ir-(*R,R,R*)] were synthesiszed according to the literuature procedure (ref.-2).

6. **General Procedure A: Cu/Ir-catalyzed Racemic Borylallylation of electron-deficient alkenes: Racemic reaction**

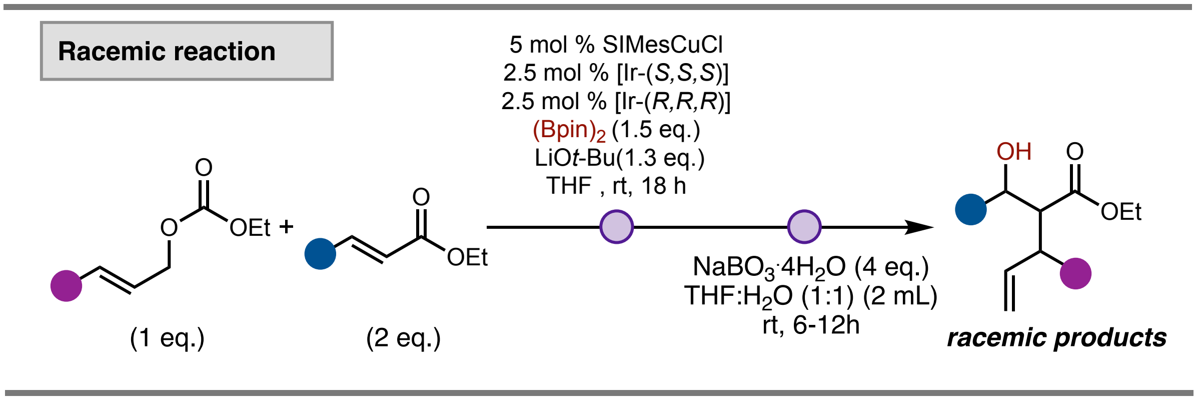

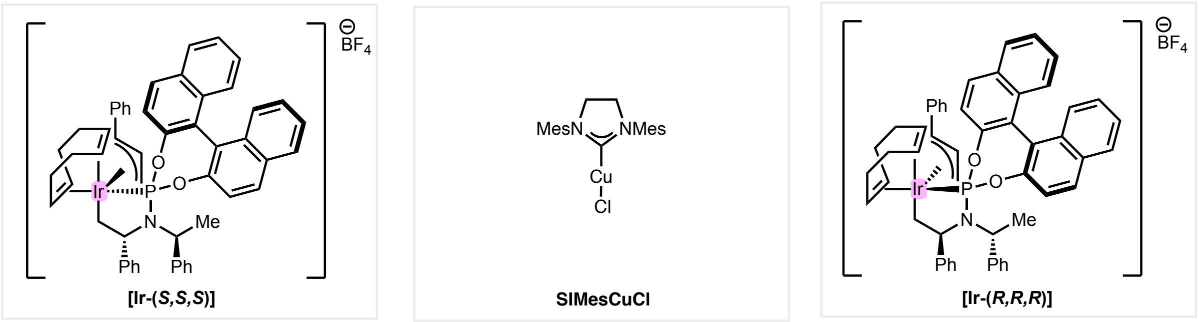

In an N_2-filled glovebox, to a flame-dried 13 × 100 mm screw cap vial with a magnetic stir bar, was added [Ir-(*R,R,R*)]-Complex (2.65 mg, 2.5 µmol, 2.5 mol%), [Ir-(*S,S,S*)]-Complex (2.65 mg, 2.5 µmol, 2.5 mol%), SIMesCuCl (2.1 mg, 5.0 µmol, 5.0 mol%), B_2pin_2 (38.1 mg, 0.15 mmol, 1.5 eq.), LiO*t*Bu (10.4 mg, 0.13 mmol, 1.3 eq.) and (*alkene (0.2 mmol, 0.2 eq.) if it is solid*). was added in that order. Then the vial was sealed with a rubber septum and lined with Teflon tape, removed from the glove box, and placed under a positive pressure of N_2. To a separate vial the electrophile, cinnamyl ethyl carbonate (0.1 mmol, 1.0 eq.) and the alkene (0.2 mmol, 2.0 eq.) was added and dissolved in 1.0 mL of THF. Then the solution was added to the reaction vial rinsing the sides of the vial. *(Note: it is important that the vial is stirring vigorously while the liquid reagents are added to prohibit aggregation of the solid reagents, which typically results in lower yields)*. Followed by another 0.5 mL of THF was added by rinsing the walls of the vial resulting a light to deep brown color of the reaction mixture. Next, the septum was quickly replaced with a Teflon lined screw cap and the reaction was stirred at room temperature for 18 hours. After 18 hours, the reaction was quenched with sat. NH_4Cl (3.0 mL), the two phases were separated, and the aqueous phase was back extracted with diethyl ether (2.0 mL × 3). The combined organic phases were dried through silica gel plug and concentrated in-vacuo. The crude organic residue further dissolved in 2.0 mL THF:H2O (1:1) and $NaBO_3{\cdot}4H_2O$ (4.0 eq.) added and the reaction mixture was stirred at room temperature for overnight. After the reaction mixture was quenched

with 3.0 mL of sat. sodium thiosulfate solution, and the organic phase separated and extracted with diethyl ether (2.0 mL × 3) and dried over anhydrous Sodium sulfate and concentrated in-vacuo. The organic residue further purified by column chromatography.

General Procedure B: Cu/Ir-catalyzed Enantioselective Borylallylation of electron-deficient alkenes: Enantioselective reaction

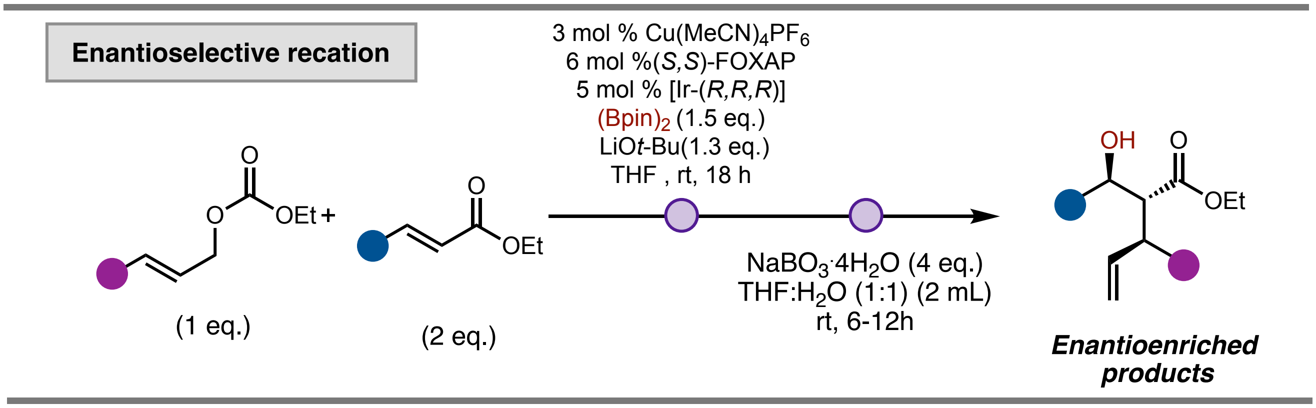

In an N_2-filled glovebox, to a flame-dried 13 × 100 mm screw cap vial with a magnetic stir bar, was added [Ir-(*R,R,R*)]-Complex (5.25 mg, 5.0 µmol, 5.0 mol%), $Cu(MeCN)_4PF_6$ (1.12 mg, 3.0 µmol, 3.0 mol%), (*S,S*)-FOXAP (2.9 mg, 6.0 µmol, 6.0 mol%), B_2pin_2 (38.1 mg, 0.15 mmol, 1.5 eq.), LiO*t*Bu (10.4 mg, 0.13 mmol, 1.3 eq.) and (*alkene (0.2 mmol, 0.2 eq.) if it is solid*). was added in that order. Then the vial was sealed with a rubber septum and lined with Teflon tape, removed from the glove box, and placed under a positive pressure of N_2. To a separate vial the electrophile, cinnamyl ethyl carbonate (0.1 mmol, 1.0 eq.) and the alkene (0.2 mmol, 2.0 eq.) was added and dissolved in 1.0 mL of THF. Then the solution was added to the reaction vial rinsing the sides of the vial. *(Note: it is important that the vial is stirring vigorously while the liquid reagents are added to prohibit aggregation of the solid reagents, which typically results in lower yields)*. Followed by another 0.5 mL of THF was added by rinsing the walls of the vial resulting a light to deep brown color of the reaction mixture. Next, the septum was quickly replaced with a Teflon lined screw cap and the reaction was stirred at room temperature for 18 hours. After 18 hours, the reaction was quenched with sat. NH_4Cl (3.0 mL), the two phases were separated, and the aqueous phase was back extracted with diethyl ether (2.0 mL × 3). The combined organic phases were dried through silica gel plug and concentrated in-vacuo. The crude organic residue further dissolved in 2.0 mL THF:H2O (1:1) and $NaBO_3{\cdot}4H_2O$ (4.0 eq.) added and the reaction mixture was stirred at room temperature for overnight. After the reaction mixture was quenched with 3.0 mL of sat. sodium thiosulfate solution, and the organic phase separated and extracted with diethyl ether (2.0 mL × 3) and dried over anhydrous Sodium sulfate and concentrated in-vacuo. The organic residue further purified by column chromatography.

General Procedure C: Cu/Ir-catalyzed Enantioselective Borylallylation of electron-deficient alkenes: Enantioselective reaction

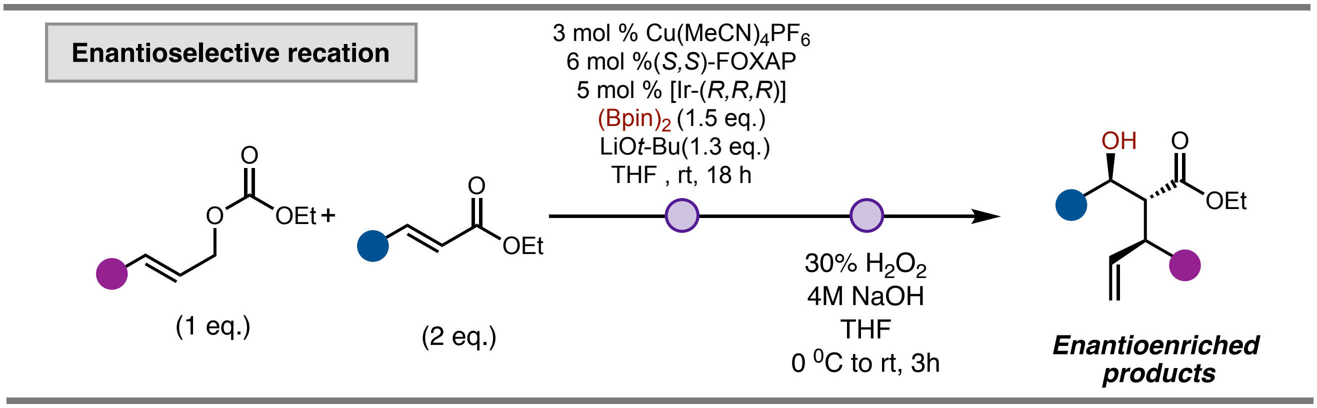

In an N_2-filled glovebox, to a flame-dried 13 × 100 mm screw cap vial with a magnetic stir bar, was added [Ir-(*R,R,R*)]-Complex (5.25 mg, 5.0 µmol, 5.0 mol%), $Cu(MeCN)_4PF_6$ (1.12 mg, 3.0 µmol, 3.0 mol%), (*S,S*)-FOXAP (2.9 mg, 6.0 µmol, 6.0 mol%), B_2pin_2 (38.1 mg, 0.15 mmol, 1.5 eq.), LiO*t*Bu (10.4 mg, 0.13 mmol, 1.3 eq.) and (*alkene (0.2 mmol, 0.2 eq.) if it is solid*). was added in that order. Then the vial was sealed with a rubber septum and lined with Teflon tape, removed from the glove box, and placed under a positive pressure of N_2. To a separate vial the electrophile, cinnamyl ethyl carbonate (0.1 mmol, 1.0 eq.) and the alkene (0.2 mmol, 2.0 eq.) was added and dissolved in 1.0 mL of THF. Then the solution was added to the reaction vial rinsing the sides of the vial. *(Note: it is important that the vial is stirring vigorously while the liquid reagents are added to prohibit aggregation of the solid reagents, which typically results in lower yields).* Followed by another 0.5 mL of THF was added by rinsing the walls of the vial resulting a light to deep brown color of the reaction mixture. Next, the septum was quickly replaced with a Teflon lined screw cap and the reaction was stirred at room temperature for 18 hours. After 18 hours, the reaction was quenched with sat. NH_4Cl (3.0 mL), the two phases were separated, and the aqueous phase was back extracted with diethyl ether (2.0 mL × 3). The crude residue obtained was dissolved in 2 mL of THF then 4M NaOH (2 mL) was added. The resulting biphasic mixture was stirred vigorously and cooled to 0 °C before 30% H_2O_2 was added. The flask was removed from the ice bath and allowed to stir at room temperature for 3 h. The reaction was diluted with H_2O (3 mL) then extracted with diethyl ether (3 x 5 mL). The combined organic dried over anhydrous Sodium sulfate and concentrated in-vacuo. The organic residue further purified by column chromatography.

7. Optimization of the reaction condition:

General procedure for condition optimization: In a flame-dried 13x100 screw capped vial equipped with a stir bar was taken into the N_2-filled glovebox. Ir-complex, $Cu(MeCN)_4PF_6$, Ligand, B_2pin_2 and LiO*t*-Bu were added. The vial was then sealed with a septum placed and taken out from the glovebox and placed into N_2-line. Then allyl carbonate electrophile and the alkene were dissolved in THF added the reaction vial via a syringe. The septum on the reaction vial was quickly replaced by a screw cap and the reaction was stirred at room temperature for 18 hours. Upon completion, the reaction was quenched by addition of 1.0 mL of saturated NH_4Cl and extracted with diethyl ether (1.0 mLx3). The solvent was evaporated and CH_2Br_2 (7 µL, 0.1 mmol) was added as an internal standard for crude ^{1}H NMR analysis to determine the yield and dr. then the crude again dissolve in 2.0 mL THF:Water (1:1) and $NaBO_3{\cdot}4H_2O$ (4.0 eq.) was added and stirred at room temperature for overnight. The alcohol product then purified by column chromatography and further used for HPLC analysis.

Optimization of the reaction: Table-1

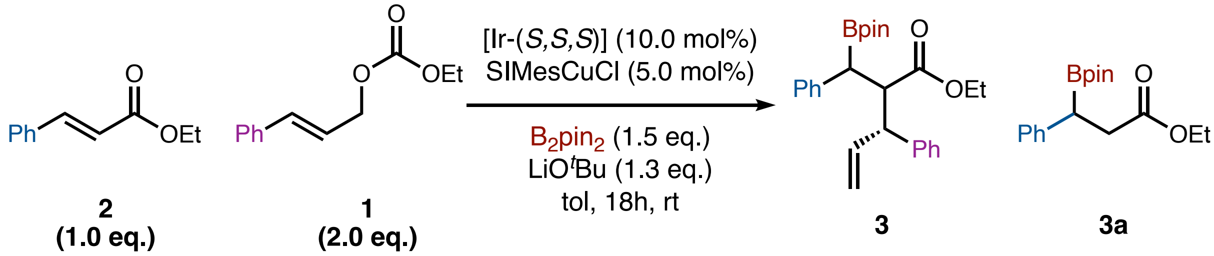

2 **(1.0 eq.)** **1** **(2.0 eq.)** **3** **3a**

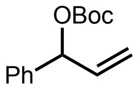

1a

entry	variation	3(br)[a]	3a[a]	dr[b]	er[c]
1	$[Ir(cod)Cl]_2$ (2 mol%)+ L_1 (4 mol%)	17%	45%	1:1	-
2	$[Ir(cod)Cl]_2$ (2 mol%)+ L_2 (4 mol%)	15%	53%	1:1	-
3	$[Ir(cod)Cl]_2$ (2 mol%)+ L_3 (4 mol%)	43%	23%	1:1	97:3
4	$[Ir(cod)Cl]_2$ (2 mol%)+ L_3 (4 mol%)**	68%	27%	1:1	95:5
5	[Ir]-cyclometalated complex**	80%	>20%	1:1	96:4
6	[Ir]-cyclometalated complex** and branch allylic carbonate (1a)	30%	>40%	1:1	ND

(**)2.0 eq. of 2 and 1.0 eq. of 1 used

[a] NMR yield determined by analysis of ^{1}HNMR of crude reaction mixture using CH_2Br_2 as internal standard.

[b] dr ratio determined by analysis of ^{1}HNMR of crude reaction mixture.

[c]enantiomeric ratio determined by HPLC analysis using chiral column after oxidation C-Bpin bond.

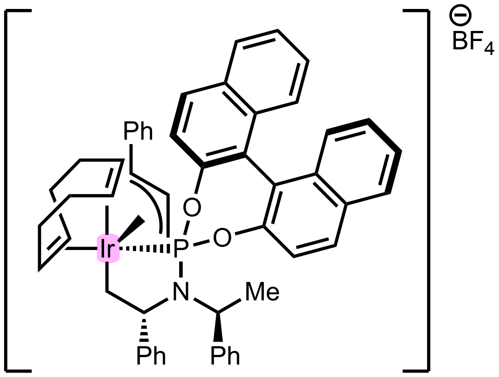

[Ir-(*S,S,S*)]

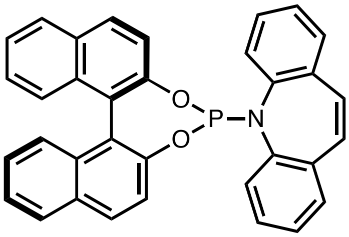

(*R*)-L_1

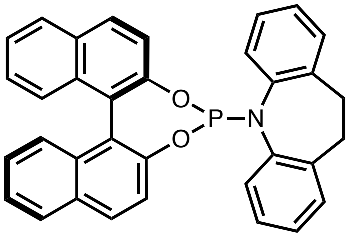

(*R*)-L_2

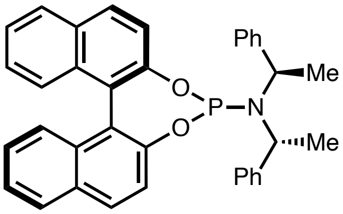

(*R,R,R*)-L_3

Alkene loading screening: Table-2

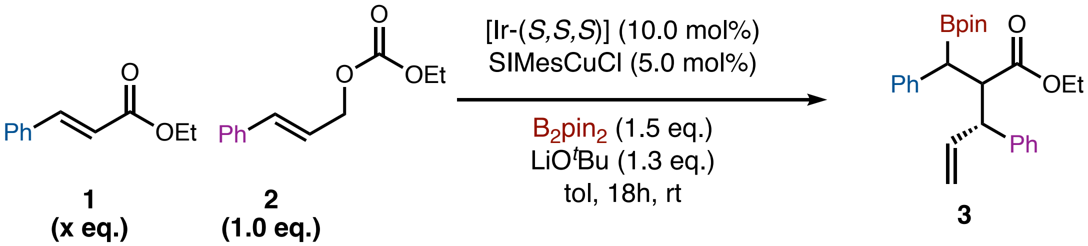

1
(x eq.)

2
(1.0 eq.)

3

entry	x (eq.)	3(br)[a]	b/l(rr)[b]	dr[c]	er[d]
1	1.0	50%	>20:1	1:1	96:4
2	1.25	58%	>20:1	1:1	95:5
3	1.5	74%	>20:1	1:1	95:5
4	2.0	80%	>20:1	1:1	95:5

[a] NMR yield determined by analysis of ^{1}HNMR of crude reaction mixture using CH_2Br_2 as internal standard.
[b] b/l ratio determined by analysis of ^{1}HNMR of crude reaction mixture.
[c] dr ratio determined by analysis of ^{1}HNMR of crude reaction mixture.
[d] enantiomeric ratio determined by HPLC analysis using chiral column after oxidation C-Bpin bond.

Phosphine ligands screening: Table-3

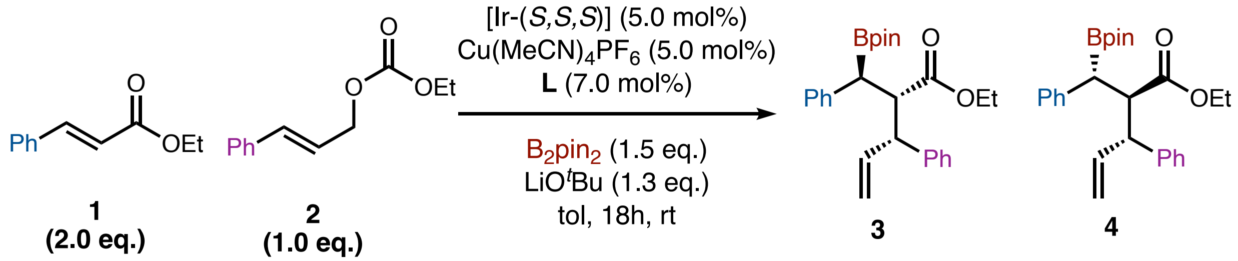

L	br[a]	b/l	dr(3/4)[b]	er[c]
**L1**	<2%; 95% RSM	-	-	-
**L2**	55%	>20:1	64:36	96:4
**L3**	86%	>20:1	90:10	99.5:0.5
**L4**	85%	>20:1	**94:6**	99.9:0.1
**L5**	62%	>20:1	85:15	98:2
**L6**	90%	>20:1	86:15	99.9:0.1
**L7**	86%	>20:1	90:10	99.5:0.5
**L8**	100%	>20:1	67:33	98:2 & 96:4
**L9**	91%	>20:1	76:24	98:2 & 92:8
**L10**	62%	>20:1	55:45	99.5:0.5
**L11**	77%	>20:1	77:23	99.5:0.5

[a] NMR yield determined by analysis of ^{1}HNMR of crude reaction mixture using CH_2Br_2 as internal standard.
[b] dr ratio determined by analysis of ^{1}HNMR of crude reaction mixture and GC analysis
[c]enantiomeric ratio determined by HPLC analysis using chiral column after oxidation C-Bpin bond.

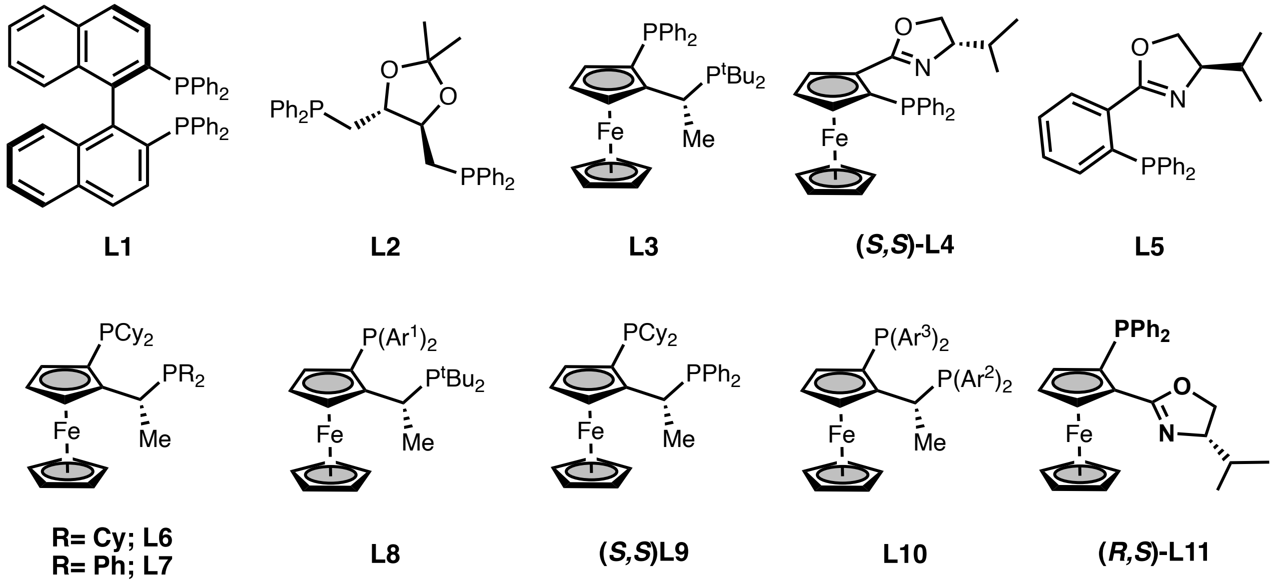

Ar = 3,5-di-*tert*-butyl-4-methoxyphenyl; Ar^1 = 2-methylphenyl; Ar^2 = 3,5-di-methylphenyl; Ar^3 = 3,5-bis(trifluoromethyl)phenyl)

Solvents screening: Table-4

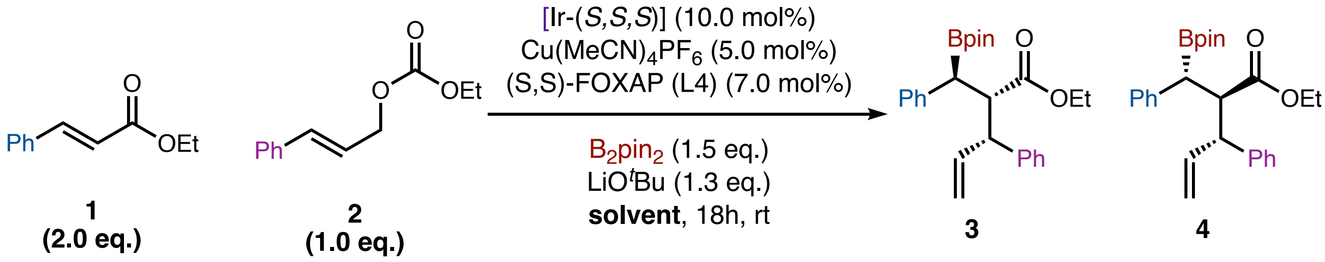

solvent	3(br)[a]	b/l(rr)[b]	dr(3/4)[c]	er[d]
Toluene	85%	>20:1	94:6	99.9:0.1
Diethyl ether	85%	>20:1	92:8	99.9:0.1
Toluene:THF (10:1)	72%	>20:1	92:8	99.9:0.1
THF	93%	>20:1	98:2	99.9:0.1

[a] NMR yield determined by analysis of ^{1}HNMR of crude reaction mixture using CH_2Br_2 as internal standard.
[b] dr ratio determined by analysis of ^{1}HNMR of crude reaction mixture and GC analysis
[c]enantiomeric ratio determined by HPLC analysis using chiral column after oxidation C-Bpin bond.

[Ir]- catalyst loading screening: Table-5

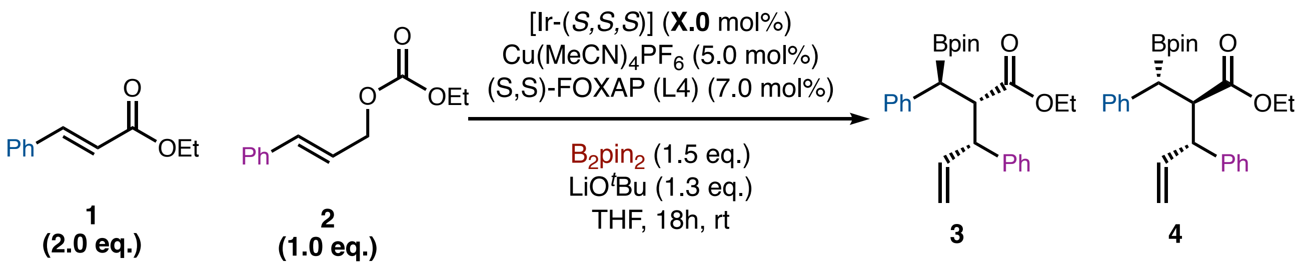

X mol%	3(br)[a]	b/l(rr)[b]	dr(3/4)[c]	er[d]
2.5 mol%	41%	>20:1	98:2	99.9:0.1
5.0 mol%	85%	>20:1	98:2	99.9:0.1
7.5 mol%	90%	>20:1	98:2	99.9:0.1

[a] NMR yield determined by analysis of ^{1}HNMR of crude reaction mixture using CH_2Br_2 as internal standard.
[b] dr ratio determined by analysis of ^{1}HNMR of crude reaction mixture and GC analysis
[c]enantiomeric ratio determined by HPLC analysis using chiral column after oxidation C-Bpin bond.

-R group screening: Table-6

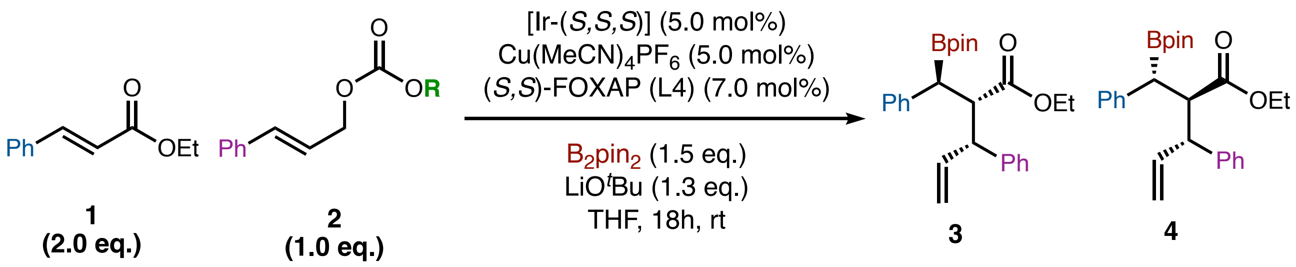

-R	3(br)[a]	b/l(rr)[b]	dr[c]	er[d]
-Me	80%	>20:1	97:3	99.9:0.1
-Et	85%	>20:1	98:2	99.9:0.1
-*t*Bu	60%	>20:1	95:5	99:1
$-CH_2CCl_3$	70%	>20:1	98:2	99:1

[a] NMR yield determined by analysis of ^{1}HNMR of crude reaction mixture using CH_2Br_2 as internal standard.
[b] dr ratio determined by analysis of ^{1}HNMR of crude reaction mixture and GC analysis
[c]enantiomeric ratio determined by HPLC analysis using chiral column after oxidation C-Bpin bond.

Final Optimized condition: Table-7

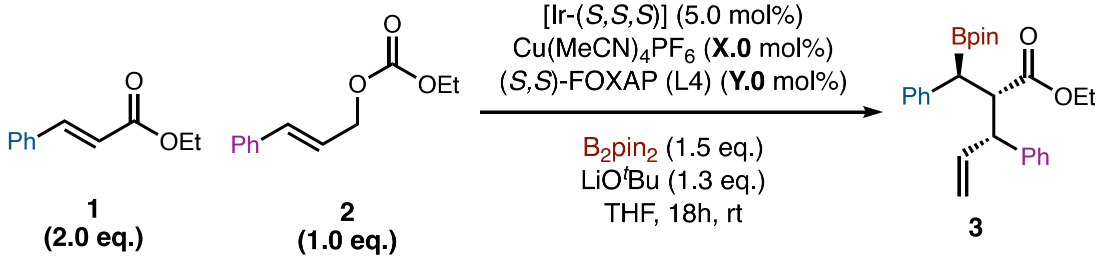

Variation	3(br)[a]	b/l(rr)[b]	dr[c]	er[d]
$Cu(MeCN)_4PF_6$ (**3.0** mol%)<br>(S,S)-FOXAP (L9) (**6.0** mol%	93%(84%)	>20:1	>98:2	99.9:0.1

isolated yield in parenthesis
[a] NMR yield determined by analysis of ^{1}HNMR of crude reaction mixture using CH_2Br_2 as internal standard.
[b] dr ratio determined by analysis of ^{1}HNMR of crude reaction mixture and GC analysis
[c]enantiomeric ratio determined by HPLC analysis using chiral column after oxidation C-Bpin bond.

Control experiments: Table-8

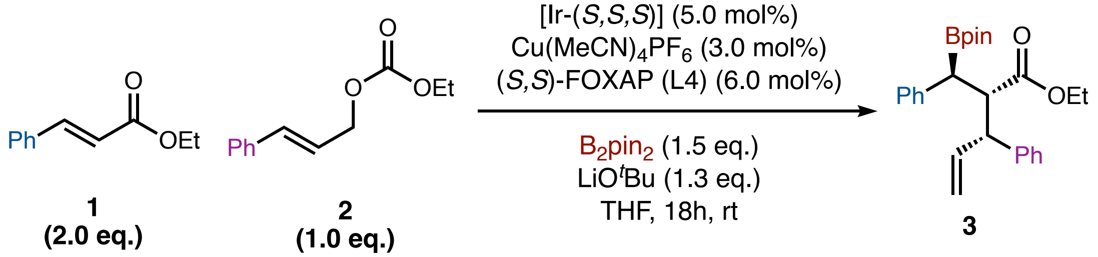

Variation	3(br)[a]	b/l(rr)[b]	dr[c]	er[d]
Cu cat.<br>**No [Ir-(*S,S,S*)]**	-	NA	NA	NA
**No Cu cat.**<br>[Ir-(*S,S,S*)] (5.0 mol%)	-	NA	NA	NA

isolated yield in parenthesis
[a] NMR yield determined by analysis of ^{1}HNMR of crude reaction mixture using CH_2Br_2 as internal standard.
[b] dr ratio determined by analysis of ^{1}HNMR of crude reaction mixture and GC analysis
[c]enantiomeric ratio determined by HPLC analysis using chiral column after oxidation C-Bpin bond.

8. Experimental Data:

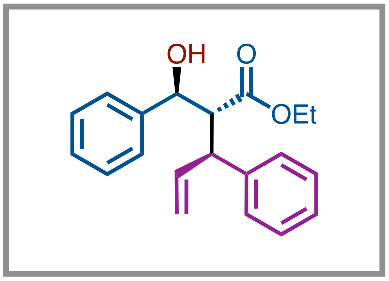

ethyl (2R,3S)-2-((S)-hydroxy(phenyl)methyl)-3-phenylpent-4-enoate (5)

The title compound was prepared according to General Procedure B with 93% NMR yield and >98:2 dr by ^{1}HNMR analysis of crude reaction mixture before C-Bpin oxidation. Purification by silica-gel flash column chromatography (Gradient: Hexane to 5-6% EtOAc:Hexanes) yields with 84% (after oxidation of C-Bpin bond) with >98:2 dr as colorless semi-solid compound.

^{1}H NMR (500 MHz, $CDCl_3$) δ 7.35 – 7.10 (m, 10H), 6.04 (ddd, *J* = 16.9, 10.1, 9.1 Hz, 1H), 5.32 (dt, *J* = 17.0, 1.0 Hz, 1H), 5.20 (dd, *J* = 10.1, 1.4 Hz, 1H), 5.02 (dd, *J* = 10.1, 2.9 Hz, 1H), 4.10 – 3.82 (m, 2H), 3.53 (qq, *J* = 7.3, 3.6 Hz, 2H), 3.04 (dd, *J* = 11.1, 3.0 Hz, 1H), 0.57 (t, *J* = 7.1 Hz, 3H).

^{13}C NMR (126 MHz, $CDCl_3$) δ 174.0, 142.8, 141.1, 138.3, 128.6, 128.4, 128.2, 127.4, 127.0, 125.4, 117.9, 71.7, 60.4, 57.2, 50.2, 13.5

HRMS (ESI): Calculated for $C_{20}H_{22}O_3Na$ [M+Na]: 333.1461, Found: 333.1455

HPLC: >99.5:0.5 enantiomeric ratio; ChiralPak IA-3 column using 95:5 Hexane: IPA as gradient, 1.0 mL/min, 220 nm. τ_{major} = 10.79 min and τ_{minor} = 18.26 min.

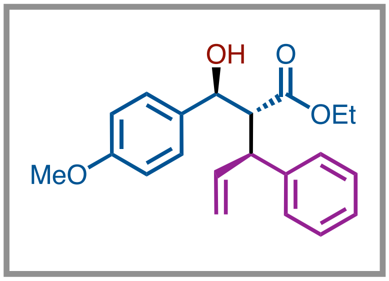

ethyl (2R,3S)-2-((S)-hydroxy(4-methoxyphenyl) methyl)-3-phenylpent-4-enoate (6)

The title compound was prepared according to General Procedure B with 84% NMR yield and 98:2 dr by ^{1}HNMR analysis of crude reaction mixture before C-Bpin oxidation. Purification by silica-gel flash column chromatography (Gradient: Hexane to 8-9% EtOAc:Hexanes) yields with 63% (after oxidation of C-Bpin bond) with >98:2 dr as colorless semi-solid compound.

^{1}H NMR (500 MHz, $CDCl_3$) δ 7.28 (d, *J* = 7.4 Hz, 1H), 7.26 (d, *J* = 2.5 Hz, 1H), 7.25 – 7.21 (m, 4H), 7.19 (td, *J* = 6.9, 1.5 Hz, 1H), 6.88 – 6.82 (m, 2H), 6.07 (ddd, *J* = 16.9, 10.1, 9.0 Hz, 1H),

5.34 (dt, J = 17.0, 1.2 Hz, 1H), 5.22 (dd, J = 10.1, 1.5 Hz, 1H), 5.01 (dd, J = 9.9, 3.1 Hz, 1H), 4.02 – 3.91 (m, 2H), 3.79 (s, 3H), 3.61 (qq, J = 10.8, 7.2 Hz, 2H), 3.06 (dd, J = 11.0, 3.1 Hz, 1H), 0.66 (t, J = 7.1 Hz, 3H).
^{13}C NMR (126 MHz, CDCl$_3$) δ 174.1, 158.9, 141.2, 138.3, 134.9, 128.6, 128.2, 127.0, 126.6, 117.8, 113.8, 71.4, 60.4, 57.2, 55.4, 50.3, 13.6
HRMS (ESI): Calculated for $C_{21}H_{24}O_4Na$ [M+Na]: 363.1566, Found: 363.1561
HPLC: >99.5:0.5 enantiomeric ratio; ChiralPak IA-3 column using 95:5 Hexane: IPA as gradient, 1.0 mL/min, 220 nm. τ_{major} = 17.40 min and τ_{minor} = 20.82 min.

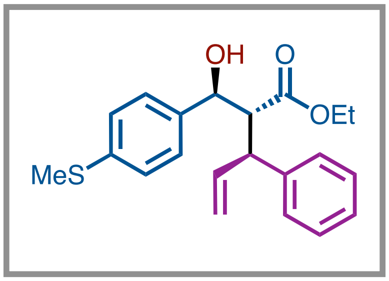

ethyl (2R,3S)-2-((S)-hydroxy(4-(methylthio) phenyl) methyl)-3-phenylpent-4-enoate (7)
The title compound was prepared according to General Procedure B with 73% NMR yield and 98:2 dr by crude ^{1}HNMR analysis of crude reaction mixture before C-Bpin oxidation. Purification by silica-gel flash column chromatography (Gradient: Hexane to 9-10% EtOAc: Hexanes) yields with 59% (after oxidation of C-Bpin bond) with >98:2 dr as colorless oil compound.
^{1}H NMR (500 MHz, CDCl$_3$) δ 7.35 – 7.18 (m, 9H), 6.12 (ddd, J = 16.9, 10.1, 9.1 Hz, 1H), 5.40 (dt, J = 17.0, 1.2 Hz, 1H), 5.28 (dd, J = 10.1, 1.4 Hz, 1H), 5.06 (dd, J = 10.0, 3.0 Hz, 1H), 4.08 (d, J = 10.1 Hz, 1H), 4.03 (dd, J = 11.1, 9.1 Hz, 1H), 3.64 (dddd, J = 17.9, 10.8, 7.1, 3.6 Hz, 2H), 3.10 (dd, J = 11.1, 3.0 Hz, 1H), 2.50 (s, 3H), 0.68 (t, J = 7.1 Hz, 3H).
^{13}C NMR (126 MHz, CDCl$_3$) δ 174.0, 141.0, 139.8, 138.2, 137.4, 128.6, 128.2, 127.1, 126.7, 126.0, 118.0, 71.4, 60.5, 57.0, 50.3, 16.1, 13.6
HRMS(ESI): Calculated for $C_{21}H_{24}SO_3Na$ [M+Na]: 379.1338, Found: 379.1334
HPLC: >99.5:0.5 enantiomeric ratio; Phenomenex Lux 3u Cellulose-1 column using 95:5 Hexane: IPA as gradient, 0.5 mL/min, 220 nm. τ_{minor} = 19.84 min and τ_{major} = 26.24 min.

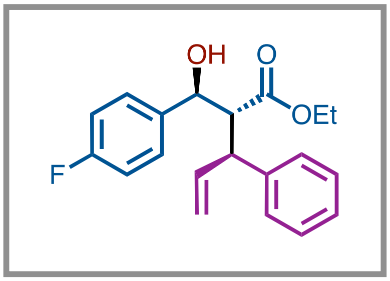

ethyl (2R,3S)-2-((S)-(4-fluorophenyl) (hydroxy)methyl)-3-phenylpent-4-enoate (8)
The title compound was prepared according to General Procedure B with 86% NMR yield and 96:4 dr by crude ^{1}HNMR analysis of crude reaction mixture before C-Bpin oxidation. Purification

by silica-gel flash column chromatography (Gradient: Hexane to 6-7% EtOAc: Hexanes) yields with 62% (after oxidation of C-Bpin bond) with 96:4 dr as colorless oil compound.
^{1}H NMR (500 MHz, CDCl$_3$) δ 7.29 – 7.23 (m, 4H), 7.23 – 7.14 (m, 3H), 7.03 – 6.94 (m, 2H), 6.06 (ddd, *J* = 16.9, 10.1, 9.1 Hz, 1H), 5.34 (dt, *J* = 17.0, 1.1 Hz, 1H), 5.23 (dd, *J* = 10.2, 1.5 Hz, 1H), 5.02 (dd, *J* = 10.0, 2.9 Hz, 1H), 4.07 (d, *J* = 10.0 Hz, 1H), 3.97 (dd, *J* = 11.1, 9.1 Hz, 1H), 3.57 (qd, *J* = 7.1, 6.0 Hz, 2H), 3.03 (dd, *J* = 11.1, 3.0 Hz, 1H), 0.63 (t, *J* = 7.1 Hz, 3H)
^{13}C NMR (126 MHz, CDCl$_3$) δ 174.0, 162.1 (d, *J* = 245.1 Hz), 141.0, 138.6, 138.6, 138.1, 128.4 (d, *J* = 62.6 Hz), 127.1 (d, *J* = 3.7 Hz), 127.0, 118.0, 115.2 (d, *J* = 21.3 Hz), 71.2, 60.5, 57.2, 50.2, 13.6
^{19}F NMR (471 MHz, CDCl$_3$) δ -112.47 – -123.47 (m)
HRMS(ESI): Calculated for $C_{20}H_{21}FO_3Na$ [M+Na]: 351.1366, Found: 351.1363
HPLC: 98.5:1.5 enantiomeric ratio; Phenomenex Lux 3u Cellulose-2 column using 90:10 Hexane: IPA as gradient, 1.0 mL/min, 220 nm. τ_{minor} = 7.69 min and τ_{major} = 11.58 min.

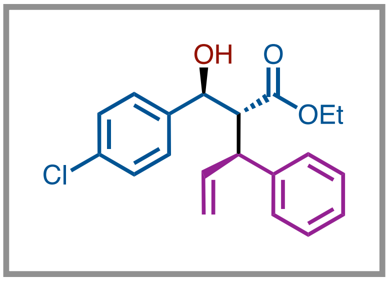

ethyl (2R,3S)-2-((S)-(4-chlorophenyl) (hydroxy)methyl)-3-phenylpent-4-enoate (9)
The title compound was prepared according to General Procedure B with 65% NMR yield and 95:5 dr by crude ^{1}HNMR analysis of crude reaction mixture before C-Bpin oxidation. Purification by silica-gel flash column chromatography (Gradient: Hexane to 6-7% EtOAc: Hexanes) yields with 57% (after oxidation of C-Bpin bond) with >98:2 dr as colorless oil compound.
^{1}H NMR (500 MHz, CDCl$_3$) δ 7.33 – 7.16 (m, 9H), 6.08 (ddd, *J* = 17.0, 10.1, 9.2 Hz, 1H), 5.37 (dt, *J* = 17.0, 1.1 Hz, 1H), 5.25 (dd, *J* = 10.2, 1.4 Hz, 1H), 5.03 (dd, *J* = 10.1, 2.9 Hz, 1H), 4.13 (dd, *J* = 10.1, 1.0 Hz, 1H), 3.99 (dd, *J* = 11.1, 9.2 Hz, 1H), 3.53-3.66 (m, 2H), 3.04 (dd, *J* = 11.1, 2.9 Hz, 1H), 0.64 (t, *J* = 7.1 Hz, 3H)
^{13}C NMR (126 MHz, CDCl$_3$) δ 173.9, 141.4, 140.9, 138.0, 133.1, 128.7, 128.5, 128.1, 127.1, 126.8, 118.1, 71.2, 60.6, 56.9, 50.3, 13.5
HRMS(ESI): Calculated for $C_{20}H_{21}ClO_3Na$ [M+Na]: 367.1071, Found: 367.1068

HPLC: >99.5:0.5 enantiomeric ratio; Chiral Pak IA-3 column using 95:5 Hexane: IPA as gradient, 0.5 mL/min, 220 nm. τ_{major} = 11.67 min and τ_{minor} = 13.31 min.

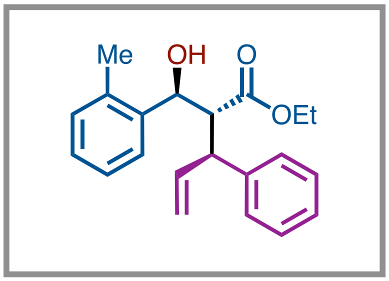

ethyl (2R,3S)-2-((S)-hydroxy(o-tolyl) methyl)-3-phenylpent-4-enoate (10)

The title compound was prepared according to General Procedure B with 82% NMR yield and 98:2 dr by ^{1}HNMR analysis of crude reaction mixture before C-Bpin oxidation. Purification by silica-gel flash column chromatography (Gradient: Hexane to 7-8% EtOAc: Hexanes) yields with 70% (after oxidation of C-Bpin bond) with >98:2 dr as colorless powder compound.

^{1}H NMR (500 MHz, $CDCl_3$) δ 7.30 – 7.09 (m, 9H), 6.10 (dt, *J* = 16.9, 9.7 Hz, 1H), 5.45 – 5.37 (m, 1H), 5.27 (ddd, *J* = 10.0, 6.6, 2.0 Hz, 2H), 4.27 – 4.21 (m, 1H), 4.07 – 3.98 (m, 1H), 3.69 – 3.53 (m, 2H), 2.99 – 2.92 (m, 1H), 2.32 (s, 3H), 0.65 (t, *J* = 7.1 Hz, 3H).

^{13}C NMR (126 MHz, $CDCl_3$) δ 174.0, 141.2, 140.7, 138.0, 133.6, 130.5, 128.6, 128.1, 127.3, 127.0, 126.1, 124.9, 118.2, 68.8, 60.4, 55.1, 50.6, 19.2, 13.6

HRMS(ESI): Calculated for $C_{21}H_{24}O_3Na$ [M+Na]: 347.1617, Found: 347.1613

HPLC: 97:3 enantiomeric ratio; Chiral Pak IA-3 column using 95:5 Hexane: IPA as gradient, 1.0 mL/min, 220 nm. τ_{major} = 9.66 min and τ_{minor} = 16.36 min.

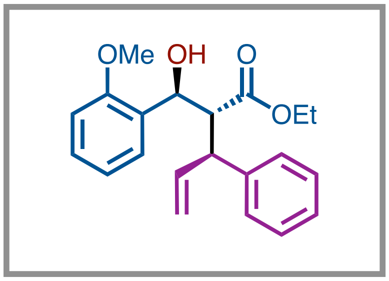

ethyl (2R,3S)-2-((S)-hydroxy(2-methoxyphenyl) methyl)-3-phenylpent-4-enoate (11)

The title compound was prepared according to General Procedure B with 68% NMR yield and 91:9 dr by ^{1}HNMR analysis of crude reaction mixture before C-Bpin oxidation. Purification by silica-gel flash column chromatography (Gradient: Hexane to 9-10% EtOAc: Hexanes) yields with 56% (after oxidation of C-Bpin bond) with 96:4 dr as colorless oil compound.

^{1}H NMR (500 MHz, $CDCl_3$) δ 7.33 – 7.10 (m, 7H), 6.94 (td, *J* = 7.5, 1.1 Hz, 1H), 6.86 (dd, *J* = 8.2, 1.0 Hz, 1H), 6.12 (ddd, *J* = 17.0, 10.2, 8.8 Hz, 1H), 5.44 – 5.33 (m, 2H), 5.24 (dd, *J* = 10.2, 1.5 Hz, 1H), 4.13 (d, *J* = 10.3 Hz, 1H), 4.03 (dd, *J* = 11.3, 8.9 Hz, 1H), 3.89 (s, 3H), 3.58 (dddd, *J* = 17.9, 10.8, 7.2, 3.6 Hz, 2H), 3.29 (dd, *J* = 11.3, 2.7 Hz, 1H), 0.63 (t, *J* = 7.1 Hz, 3H).

^{13}C NMR (126 MHz, $CDCl_3$) δ 174.3, 155.8, 141.7, 138.4, 130.9, 128.5, 128.3, 128.2, 126.8, 126.1, 120.6, 117.2, 110.1, 67.6, 60.2, 55.5, 54.5, 50.1, 13.6

HRMS(ESI): Calculated for $C_{21}H_{24}O_4Na$ [M+Na]: 363.1566, Found: 363.1562
HPLC: 96:4 enantiomeric ratio; Chiral Pak IA-3 column using 95:5 Hexane: IPA as gradient, 1.0 mL/min, 220 nm. τ_{major} = 12.82 min and τ_{minor} = 14.23 min.

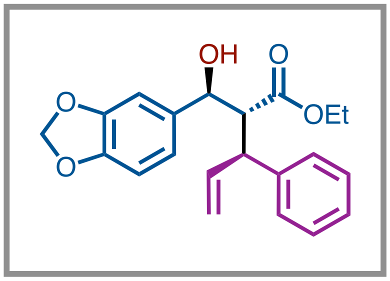

ethyl (2R,3S)-2-((S)-benzo[d][1,3] dioxol-5-yl(hydroxy)methyl)-3-phenylpent-4-enoate (12)
The title compound was prepared according to General Procedure B with 94% NMR yield and 98:2 dr by ^{1}HNMR analysis of crude reaction mixture before C-Bpin oxidation. Purification by silica-gel flash column chromatography (Gradient: Hexane to 9-10% EtOAc: Hexanes) yields with 73% (after oxidation of C-Bpin bond) with >98:2 dr as colorless oil compound.
^{1}H NMR (500 MHz, $CDCl_3$) δ 7.31 – 7.25 (m, 2H), 7.24 – 7.16 (m, 3H), 6.81 (dd, *J* = 1.5, 0.8 Hz, 1H), 6.79 – 6.71 (m, 2H), 6.06 (ddd, *J* = 16.9, 10.1, 9.1 Hz, 1H), 5.93 (s, 2H), 5.34 (dt, *J* = 16.9, 1.1 Hz, 1H), 5.23 (dd, *J* = 10.2, 1.4 Hz, 1H), 4.96 (dd, *J* = 9.8, 3.0 Hz, 1H), 4.02 (d, *J* = 9.8 Hz, 1H), 3.96 (dd, *J* = 11.0, 9.1 Hz, 1H), 3.64 (qq, *J* = 7.3, 3.6 Hz, 2H), 3.03 (dd, *J* = 11.0, 3.0 Hz, 1H), 0.69 (t, *J* = 7.1 Hz, 3H)
^{13}C NMR (126 MHz, $CDCl_3$) δ 174.1, 147.8, 146.8, 141.1, 138.2, 137.0, 128.6, 128.2, 127.1, 118.6, 117.9, 108.2, 106.2, 101.1, 71.6, 60.5, 57.2, 50.3, 13.6
HRMS (ESI): Calculated for $C_{21}H_{22}O_5Na$ [M+Na]: 377.1359, Found: 377.1356
HPLC: >99.5:0.5 enantiomeric ratio; ChiralPak IA-3 column using 95:5 Hexane: IPA as gradient, 1.0 mL/min, 220 nm. τ_{major} = 19.174 min and τ_{minor} = 24.576 min.

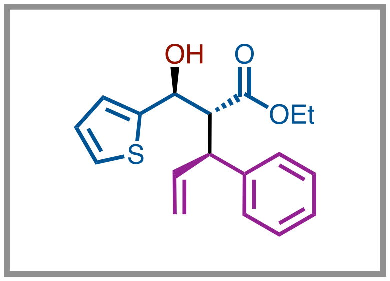

ethyl (2R,3S)-2-((S)-hydroxy(thiophen-2-yl) methyl)-3-phenylpent-4-enoate (13)
The title compound was prepared according to General Procedure B with 60% NMR yield and 90:10 dr by ^{1}HNMR analysis of crude reaction mixture before C-Bpin oxidation. Purification by silica-gel flash column chromatography (Gradient: Hexane to 9-10% EtOAc: Hexanes) yields with 45% (after oxidation of C-Bpin bond) with >95:5 dr as colorless oil compound.
^{1}H NMR (500 MHz, $CDCl_3$) δ 7.22 – 7.17 (m, 2H), 7.16 – 7.09 (m, 4H), 6.87 – 6.80 (m, 2H), 5.98 (ddd, *J* = 16.9, 10.1, 9.1 Hz, 1H), 5.25 (dt, *J* = 17.0, 1.1 Hz, 1H), 5.12 (ddd, *J* = 10.3, 7.7, 2.2

Hz, 2H), 4.18 (d, J = 10.5 Hz, 1H), 3.90 (dd, J = 11.0, 9.1 Hz, 1H), 3.58 (qd, J = 7.1, 2.1 Hz, 2H), 3.09 (dd, J = 11.0, 2.9 Hz, 1H), 0.62 (t, J = 7.1 Hz, 3H).
^{13}C NMR (126 MHz, $CDCl_3$) δ 174.2, 147.5, 140.9, 137.9, 128.7, 128.2, 127.1, 126.9, 124.6, 123.1, 118.1, 68.9, 60.7, 56.5, 50.2, 13.6
HRMS(ESI): Calculated for $C_{18}H_{20}SO_3Na$ [M+Na]: 339.1025, Found: 339.1021
HPLC: >99.5:0.5 enantiomeric ratio; Chiral Pak IA-3 column using 95:5 Hexane: IPA as gradient, 1.0 mL/min, 220 nm. τ_{major} = 10.35 min and τ_{minor} = 15.96 min.

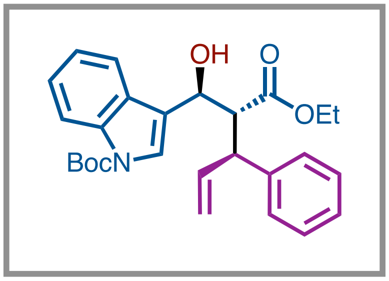

tert-butyl 3-((1S,2R,3S)-2-(ethoxycarbonyl)-1-hydroxy-3-phenylpent-4-en-1-yl)-1H-indole-1-carboxylate (14)
The title compound was prepared according to General Procedure B with 77% NMR yield and 98:2 dr by ^{1}HNMR analysis of crude reaction mixture before C-Bpin oxidation. Purification by silica-gel flash column chromatography (Gradient: Hexane to 5-6% EtOAc: Hexanes) yields with 71% (after oxidation of C-Bpin bond) with >98:2 dr as colorless semi-solid compound.
^{1}H NMR (500 MHz, $CDCl_3$) δ 8.08 (d, J = 8.2 Hz, 1H), 7.54 (d, J = 7.7 Hz, 1H), 7.43 (s, 1H), 7.29 – 7.24 (m, 1H), 7.24 – 7.17 (m, 5H), 7.16 – 7.11 (m, 1H), 6.08 (ddd, J = 17.0, 10.1, 9.2 Hz, 1H), 5.34 (dt, J = 16.9, 1.1 Hz, 1H), 5.27 – 5.18 (m, 2H), 4.06 – 3.97 (m, 2H), 3.61 (qd, J = 7.1, 3.3 Hz, 2H), 3.21 (dd, J = 11.0, 2.7 Hz, 1H), 1.58 (s, 9H), 0.63 (t, J = 7.1 Hz, 3H)
^{13}C NMR (126 MHz, $CDCl_3$) δ 174.4, 149.8, 141.1, 138.2, 135.9, 128.8, 128.6, 128.2, 127.1, 124.7, 122.9, 122.8, 122.3, 119.7, 118.1, 115.5, 83.8, 66.3, 60.6, 54.8, 50.2, 28.3, 13.6
HRMS (ESI): Calculated for $C_{27}H_{31}NO_5Na$ [M+Na]: 472.2094, Found: 472.2089
HPLC: >99.5:0.5 enantiomeric ratio; Phenomenex Lux 3u Cellulose-2 column using 97.5:2.5 Hexane: IPA as gradient, 0.5 mL/min, 220 nm. τ_{major} = 22.64 min and τ_{minor} = 33.54 min.

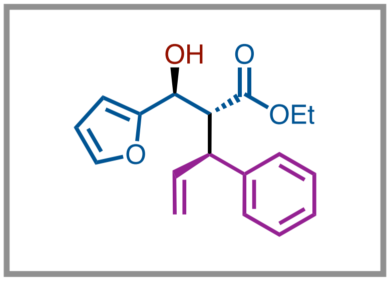

ethyl (2R,3S)-2-((S)-furan-2-yl(hydroxy)methyl)-3-phenylpent-4-enoate (15)
The title compound was prepared according to General Procedure B with 50% NMR yield and 85:15 dr by ^{1}HNMR analysis of crude reaction mixture before C-Bpin oxidation... Purification by

silica-gel flash column chromatography (Gradient: Hexane to 9-10% EtOAc: Hexanes) yields with 34% (after oxidation of C-Bpin bond) with 90:10 dr as colorless oil compound.

^{1}H NMR (500 MHz, $CDCl_3$; major diastereomer) δ 7.36 – 7.32 (m, 1H), 7.31 – 7.27 (m, 2H), 7.25 – 7.17 (m, 3H), 6.30 (dd, *J* = 3.2, 1.8 Hz, 1H), 6.25 (dt, *J* = 3.3, 0.9 Hz, 1H), 6.04 (dddd, *J* = 16.9, 12.8, 10.2, 9.2 Hz, 1H), 5.33 (dt, *J* = 16.9, 1.2 Hz, 1H), 5.21 (dd, *J* = 10.2, 1.5 Hz, 1H), 5.00 (ddd, *J* = 10.7, 3.0, 0.9 Hz, 1H), 3.96 (dd, *J* = 10.9, 9.0 Hz, 2H), 3.79 – 3.65 (m, 2H), 3.19 (dd, *J* = 11.1, 2.9 Hz, 1H), 0.78 (t, *J* = 7.1 Hz, 3H).

^{13}C NMR (126 MHz, $CDCl_3$; major diastereomer) δ 174.1, 155.5, 141.9, 140.8, 137.9, 128.5, 128.1, 127.0, 117.8, 110.2, 106.1, 66.7, 60.5, 53.9, 49.8, 13.5

HRMS(ESI): Calculated for $C_{18}H_{20}O_4Na$ [M+Na]: 323.1253, Found: 323.1247

HPLC: >99.5:0.5 enantiomeric ratio; Chiral Pak IA-3 column using 90:10 Hexane: IPA as gradient, 1.0 mL/min, 220 nm. τ_{major} = 7.99 min and τ_{minor} = 10.71 min.

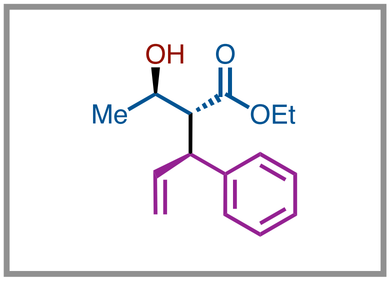

ethyl (2R,3S)-2-((R)-1-hydroxyethyl)-3-phenylpent-4-enoate (16)

The title compound was prepared according to General Procedure B with 49% NMR yield and 88:12 dr by ^{1}HNMR analysis of crude reaction mixture before C-Bpin oxidation. Purification by silica-gel flash column chromatography (Gradient: Hexane to 8-10% EtOAc: Hexanes) yields with 44% (after oxidation of C-Bpin bond) with 90:10 dr as colorless oil compound.

^{1}H NMR (500 MHz, $CDCl_3$) δ 7.30 – 7.23 (m, 2H), 7.21 – 7.13 (m, 3H), 5.94 (ddd, *J* = 17.0, 10.1, 9.1 Hz, 1H), 5.23 (ddd, *J* = 17.1, 1.5, 0.8 Hz, 1H), 5.13 (dd, *J* = 10.1, 1.5 Hz, 1H), 4.05 (t, *J* = 6.5 Hz, 1H), 3.87 (dd, *J* = 11.2, 9.1 Hz, 1H), 3.80 (q, *J* = 7.1 Hz, 2H), 2.96 (d, *J* = 10.0 Hz, 1H), 2.71 (dd, *J* = 11.2, 2.7 Hz, 1H), 1.22 (d, *J* = 6.6 Hz, 3H), 0.84 (t, *J* = 7.1 Hz, 3H)

^{13}C NMR (126 MHz, $CDCl_3$) δ 174.2, 141.5, 138.6, 128.6, 128.2, 126.9, 117.4, 65.9, 60.5, 56.5, 50.0, 22.7, 13.9

HRMS(ESI): Calculated for $C_{15}H_{20}O_3Na$ [M+Na]: 271.1304, Found: 271.1299

HPLC: >99.5:0.5 enantiomeric ratio; Chiral Pak IA-3 column using 98:2 Hexane: IPA as gradient, 1.0 mL/min, 220 nm. τ_{major} = 10.85 min and τ_{minor} = 11.60 min.

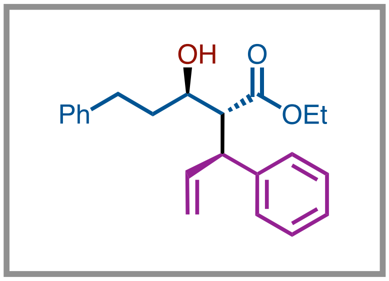

ethyl (2R,3S)-2-((R)-1-hydroxy-3-phenylpropyl)-3-phenylpent-4-enoate (17)

The title compound was prepared according to General Procedure B with 65% NMR yield and 95:5 dr by ^{1}HNMR analysis of crude reaction mixture before C-Bpin oxidation. Purification by silica-gel flash column chromatography (Gradient: Hexane to 8-10% EtOAc: Hexanes) yields with 46% (after oxidation of C-Bpin bond) with 95:5 dr as colorless oil compound.

^{1}H NMR (500 MHz, $CDCl_3$) δ 7.37 – 7.30 (m, 4H), 7.28 – 7.22 (m, 6H), 5.99 (ddd, *J* = 17.0, 10.1, 9.1 Hz, 1H), 5.31 (dt, *J* = 17.0, 1.2 Hz, 1H), 5.20 (dd, *J* = 10.2, 1.5 Hz, 1H), 4.05 – 3.91 (m, 2H), 3.90 – 3.77 (m, 2H), 3.08 (d, *J* = 10.7 Hz, 1H), 2.94 (ddd, *J* = 13.7, 9.6, 6.1 Hz, 1H), 2.84 (dd, *J* = 11.2, 2.6 Hz, 1H), 2.76 (ddd, *J* = 13.7, 9.5, 6.9 Hz, 1H), 1.86 – 1.74 (m, 2H), 0.88 (t, *J* = 7.1 Hz, 3H).

^{13}C NMR (126 MHz, $CDCl_3$) δ 174.4, 142.0, 141.4, 138.4, 128.6, 128.6, 128.5, 128.2, 127.0, 126.0, 117.5, 69.4, 60.6, 55.1, 50.0, 38.7, 32.6, 14.5

HRMS(ESI): Calculated for $C_{22}H_{26}O_3Na$ [M+Na]: 361.1774, Found: 361.1767

HPLC: 99:1 enantiomeric ratio; Phenomenex Lux 3u Cellulose-2 column using 98:2 Hexane: IPA as gradient, 1mL/min, 220 nm. τ_{minor} = 27.03 min and τ_{major} = 33.56 min.

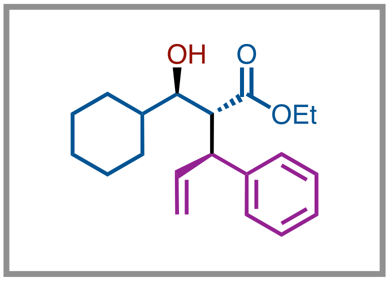

ethyl (2R,3S)-2-((R)-cyclohexyl(hydroxy)methyl)-3-phenylpent-4-enoate (18)

The title compound was prepared according to General Procedure C with 76% NMR yield and 96:4 dr by ^{1}HNMR analysis of crude reaction mixture before C-Bpin oxidation. Purification by silica-gel flash column chromatography (Gradient: Hexane to 3-4% EtOAc: Hexanes) yields with 56% (after oxidation of C-Bpin bond) with 98:2 dr as colorless powder compound.

^{1}H NMR (500 MHz, $CDCl_3$) δ 7.29 – 7.24 (m, 2H), 7.23 – 7.16 (m, 3H), 5.93 (ddd, *J* = 17.0, 10.2, 9.1 Hz, 1H), 5.25 (dt, *J* = 17.0, 1.2 Hz, 1H), 5.15 (dd, *J* = 10.1, 1.6 Hz, 1H), 3.94 (dd, *J* = 11.2, 9.1 Hz, 1H), 3.87 – 3.71 (m, 2H), 3.48 (ddd, *J* = 11.2, 9.1, 2.3 Hz, 1H), 3.00 – 2.92 (m, 2H), 2.17 –

2.06 (m, 1H), 1.83 – 1.69 (m, 3H), 1.68 – 1.59 (m, 1H), 1.24 – 1.09 (m, 4H), 1.04 – 0.89 (m, 2H), 0.84 (t, *J* = 7.1 Hz, 3H).
^{13}C NMR (126 MHz, CDCl$_3$) δ 174.7, 141.6, 138.5, 128.5, 128.2, 126.9, 117.5, 74.6, 60.4, 52.0, 50.0, 43.5, 23.0, 29.4, 26.5, 26.1, 26.0, 13.8
HRMS(ESI): Calculated for $C_{20}H_{28}O_3Na$ [M+Na]: 339.1930, Found: 339.1925
HPLC: 98:2 enantiomeric ratio; Chiral Pak IA-3 column using 90:10 Hexane: IPA as gradient, 1mL/min, 220 nm. τ_{major} = 5.49 min and τ_{minor} = 7.82 min

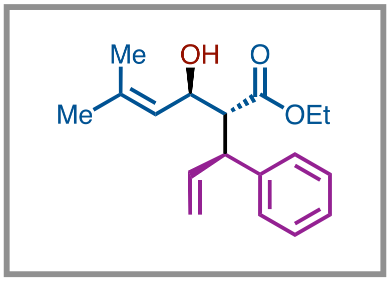

ethyl (2R,3R)-3-hydroxy-5-methyl-2-((S)-1-phenylallyl) hex-4-enoate (19)
The title compound was prepared according to General Procedure B with 58% NMR yield and 87:13 dr by ^{1}HNMR analysis of crude reaction mixture before C-Bpin oxidation. Purification by silica-gel flash column chromatography (Gradient: Hexane to 7-8% EtOAc: Hexanes) yields with 36% (after oxidation of C-Bpin bond) with 98:2 dr as colorless semi-solid compound.
^{1}H NMR (500 MHz, CDCl$_3$) δ 7.28 – 7.24 (m, 2H), 7.20 (dt, J = 8.1, 1.8 Hz, 3H), 6.02 (ddd, J = 17.0, 10.2, 9.1 Hz, 1H), 5.32 – 5.23 (m, 1H), 5.22 – 5.12 (m, 2H), 4.61 (td, J = 9.2, 3.3 Hz, 1H), 3.93 – 3.77 (m, 3H), 3.11 (d, J = 9.6 Hz, 1H), 2.74 (dd, J = 10.8, 3.3 Hz, 1H), 1.71 (d, J = 1.4 Hz, 3H), 1.67 (d, J = 1.3 Hz, 3H), 0.86 (t, J = 7.1 Hz, 3H)
^{13}C NMR (126 MHz, CDCl$_3$) δ 174.3, 141.4, 138.6, 135.8, 128.6, 128.2, 126.9, 125.8, 117.3, 67.2, 60.5, 55.8, 49.9, 25.9, 18.4, 13.9
HRMS (ESI): Calculated for $C_{18}H_{24}O_3Na$ [M+Na]: 311.1617, Found: 311.1615
HPLC: >99.5:0.5 enantiomeric ratio; Phenomenex Lux 3u Cellulose-2 column using 98:2 Hexane: IPA as gradient, 1mL/min, 220 nm. τ_{major} = 18.77 min and τ_{minor} = 24.35 min.

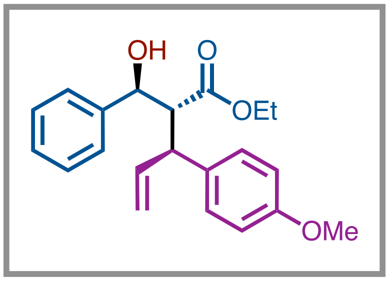

ethyl (2R,3S)-2-((S)-hydroxy(phenyl)methyl)-3-(4-methoxyphenyl) pent-4-enoate (20)
The title compound was prepared according to General Procedure C with >95% NMR yield and 96:4 dr by ^{1}HNMR analysis of crude reaction mixture before C-Bpin oxidation. Purification by

silica-gel flash column chromatography (Gradient: Hexane to 6-7% EtOAc:Hexanes) yields with 88% (after oxidation of C-Bpin bond) with >98:2 dr as colorless semi-solid compound.

^{1}H NMR (500 MHz, $CDCl_3$) δ 7.36 – 7.28 (m, 4H), 7.23 (ddt, *J* = 8.6, 5.5, 2.3 Hz, 1H), 7.20 – 7.12 (m, 2H), 6.85 – 6.78 (m, 2H), 6.06 (ddd, *J* = 16.9, 10.1, 8.9 Hz, 1H), 5.33 (dt, *J* = 17.0, 1.2 Hz, 1H), 5.22 (dd, *J* = 10.1, 1.6 Hz, 1H), 5.05 (dd, *J* = 10.1, 3.0 Hz, 1H), 4.03 (d, *J* = 10.0 Hz, 1H), 3.97 (dd, *J* = 11.0, 9.0 Hz, 1H), 3.76 (s, 3H), 3.61 (q, *J* = 7.1 Hz, 2H), 3.06 (dd, *J* = 11.1, 3.0 Hz, 1H), 0.67 (t, *J* = 7.1 Hz, 3H).

^{13}C NMR (126 MHz, $CDCl_3$) δ 174.1, 158.6, 142.8, 138.5, 133.2, 129.1, 128.4, 127.4, 125.4, 117.4, 114.0, 71.7, 60.4, 57.3, 55.4, 49.3, 13.6

HRMS (ESI): Calculated for $C_{21}H_{24}O_4Na$ [M+Na]: 363.1566, Found: 363.1563

HPLC: 99:1 enantiomeric ratio; Phenomenex Lux 3u Cellulose-2 column using 95:5 Hexane: IPA as gradient, 1.0 mL/min, 220 nm. τ_{minor} = 14.99 min and τ_{major} = 36.84 min.

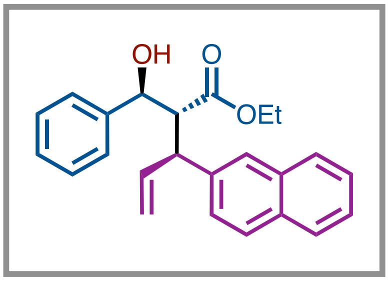

ethyl (2R,3S)-2-((S)-hydroxy(phenyl)methyl)-3-(naphthalen-2-yl) pent-4-enoate (21)

The title compound was prepared according to General Procedure B with 84% NMR yield and 98:2 dr by ^{1}HNMR analysis of crude reaction mixture before C-Bpin oxidation. Purification by silica-gel flash column chromatography (Gradient: Hexane to 5-6% EtOAc: Hexanes) yields with 80% (after oxidation of C-Bpin bond) with >98:2 dr as colorless oil compound.

^{1}H NMR (500 MHz, $CDCl_3$) δ 7.79 (dd, *J* = 8.8, 2.6 Hz, 3H), 7.70 (d, *J* = 1.8 Hz, 1H), 7.50 – 7.38 (m, 3H), 7.38 – 7.31 (m, 4H), 7.30 – 7.22 (m, 1H), 6.19 (dt, *J* = 17.0, 9.5 Hz, 1H), 5.42 (dd, *J* = 16.9, 1.4 Hz, 1H), 5.29 (dd, *J* = 10.1, 1.4 Hz, 1H), 5.14 (dd, *J* = 10.1, 2.9 Hz, 1H), 4.22 (dd, *J* = 11.1, 9.0 Hz, 1H), 4.09 (dd, *J* = 10.1, 1.9 Hz, 1H), 3.58 – 3.45 (m, 2H), 3.24 (dd, *J* = 11.1, 2.9 Hz, 1H), 0.48 (t, *J* = 7.1 Hz, 3H).

^{13}C NMR (126 MHz, $CDCl_3$) δ 173.9, 142.8, 138.6, 138.2, 133.6, 132.6, 128.4, 128.3, 127.8, 127.7, 127.4, 126.8, 126.3, 126.2, 125.8, 125.4, 118.1, 71.8, 60.4, 57.0, 50.3, 13.4

HRMS(ESI): Calculated for $C_{24}H_{24}O_3Na$ [M+Na]: 383.1587, Found: 363.1584

HPLC: 98.5:1.5 enantiomeric ratio; Chiral Pak IA-3 column using 95:5 Hexane: IPA as gradient, 1.0 mL/min, 220 nm. τ_{minor} = 6.56 min and τ_{major} = 10.39 min.

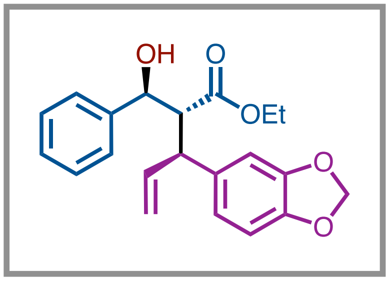

ethyl (2R,3S)-3-(benzo[d][1,3] dioxol-5-yl)-2-((S)-hydroxy(phenyl)methyl) pent-4-enoate (22)

The title compound was prepared according to General Procedure B with 91% NMR yield and 98:2 dr by ^{1}HNMR analysis of crude reaction mixture before C-Bpin oxidation. Purification by silica-gel flash column chromatography (Gradient: Hexane to 5-6% EtOAc: Hexanes) yields with 84% (after oxidation of C-Bpin bond) with 98:2 dr as colorless oil compound.

^{1}H NMR (500 MHz, $CDCl_3$) δ 7.37 – 7.26 (m, 4H), 7.26 – 7.20 (m, 1H), 6.77 – 6.66 (m, 3H), 6.03 (ddd, J = 16.9, 10.2, 9.0 Hz, 1H), 5.90 (s, 2H), 5.34 (dt, J = 17.0, 1.1 Hz, 1H), 5.23 (dd, J = 10.1, 1.4 Hz, 1H), 5.03 (dd, J = 10.0, 3.0 Hz, 1H), 4.01 – 3.90 (m, 2H), 3.73 – 3.59 (m, 2H), 3.03 (dd, J = 11.1, 3.0 Hz, 1H), 0.72 (t, J = 7.1 Hz, 3H).

^{13}C NMR (126 MHz, $CDCl_3$) δ 173.9, 147.7, 146.5, 142.7, 138.3, 135.0, 128.4, 127.4, 125.3, 121.3, 117.7, 108.5, 108.3, 101.0, 71.7, 60.4, 57.3, 49.7, 14.2

HRMS(ESI): Calculated for $C_{21}H_{21}O_5Na$ [M+Na]: 377.1359, Found: 377.1353

HPLC: >99.5:0.5 enantiomeric ratio; Phenomenex Lux 3u Cellulose-2 column using 90:10 Hexane: IPA as gradient, 1.0 mL/min, 220 nm. τ_{minor} = 10.21 min and τ_{major} = 18.27min.

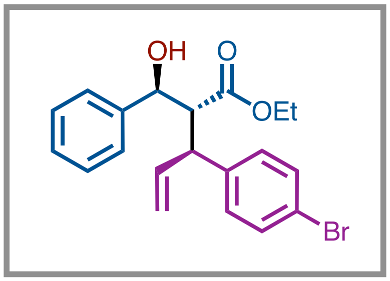

ethyl (2R,3S)-3-(4-bromophenyl)-2-((S)-hydroxy(phenyl)methyl) pent-4-enoate (23)

The title compound was prepared according to General Procedure B with 66% NMR yield and 95:5 dr by ^{1}HNMR analysis of crude reaction mixture before C-Bpin oxidation. Purification by silica-gel flash column chromatography (Gradient: Hexane to 5-6% EtOAc: Hexanes) yields with 63% (after oxidation of C-Bpin bond) with >98: dr as colorless oil compound.

^{1}H NMR (500 MHz, $CDCl_3$) δ 7.44 – 7.37 (m, 2H), 7.36 – 7.27 (m, 4H), 7.26 – 7.21 (m, 1H), 7.15 – 7.08 (m, 2H), 6.03 (ddd, J = 16.9, 10.1, 9.0 Hz, 1H), 5.35 (dt, J = 17.0, 1.1 Hz, 1H), 5.25 (dd, J = 10.0, 1.4 Hz, 1H), 5.05 (dd, J = 10.1, 3.0 Hz, 1H), 4.03 – 3.92 (m, 2H), 3.62 (qd, J = 7.1, 0.9 Hz, 2H), 3.06 (dd, J = 11.1, 3.1 Hz, 1H), 0.68 (t, J = 7.2 Hz, 3H).

^{13}C NMR (126 MHz, $CDCl_3$) δ 173.7, 142.5, 140.2, 137.7, 131.7, 129.9, 128.4, 127.5, 125.3, 120.8, 118.3, 71.7, 60.6, 57.0, 49.5, 13.6

HRMS(ESI): Calculated for $C_{20}H_{21}BrO_3Na$ [M+Na]: 411.0566, Found: 411.0564

HPLC: >99.5:0.5 enantiomeric ratio; Phenomenex Lux 3u Cellulose-1 column using 95:5 Hexane: IPA as gradient, 0.5 mL/min, 220 nm. τ_{major} = 20.08 min and τ_{minor} = 21.20 min.

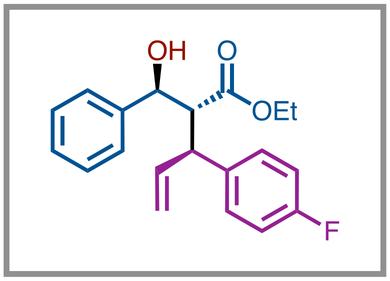

ethyl (2R,3S)-3-(4-fluorophenyl)-2-((S)-hydroxy(phenyl)methyl) pent-4-enoate (24)

The title compound was prepared according to General Procedure B with 79% NMR yield and 95:5 dr by ^{1}HNMR analysis of crude reaction mixture before C-Bpin oxidation. Purification by silica-gel flash column chromatography (Gradient: Hexane to 5-7% EtOAc:Hexanes) yields with 62% (after oxidation of C-Bpin bond) with >98:2 dr as colorless semi-solid compound.

^{1}H NMR (500 MHz, $CDCl_3$) δ 7.40 – 7.32 (m, 4H), 7.31 – 7.22 (m, 3H), 7.01 (t, J = 8.7 Hz, 2H), 6.09 (ddd, J = 16.9, 10.1, 9.0 Hz, 1H), 5.39 (d, J = 16.9 Hz, 1H), 5.29 (dd, J = 10.1, 1.4 Hz, 1H), 5.10 (dd, J = 10.1, 3.0 Hz, 1H), 4.09 – 4.00 (m, 2H), 3.69 – 3.61 (m, 2H), 3.10 (dd, J = 11.1, 3.0 Hz, 1H), 0.71 (t, J = 7.1 Hz, 3H).

^{13}C NMR (126 MHz, $CDCl_3$) δ 173.9, 161.9 (d, J = 245.2 Hz), 142.6, 138.0, 136.9 (d, J = 3.3 Hz), 129.7 (d, J = 7.9 Hz), 128.4, 127.5, 125.3, 118.0, 115.4 (d, J = 21.3 Hz), 71.7, 60.5, 57.3, 49.3, 13.6

^{19}F NMR (471 MHz, $CDCl_3$) δ -115.48 – -116.38 (m)

HRMS (ESI): Calculated for $C_{20}H_{21}O_3FNa$ [M+Na]: 351.1366, Found: 351.1363

HPLC: 98:2 enantiomeric ratio; ChiralPak IA-3 column using 95:5 Hexane: IPA as gradient, 1.0 mL/min, 220 nm. τ_{major} = 12.37 min and τ_{minor} = 13.66 min.

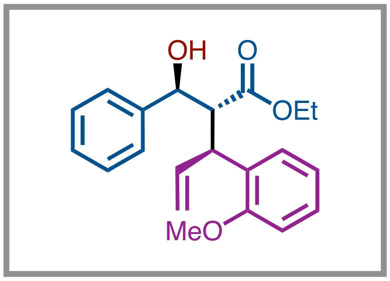

ethyl (2R,3S)-2-((S)-hydroxy(phenyl)methyl)-3-(2-methoxyphenyl) pent-4-enoate (25)

The title compound was prepared according to General Procedure B with 48% NMR yield and 88:12 dr by ^{1}HNMR analysis of crude reaction mixture before C-Bpin oxidation. Purification by silica-gel flash column chromatography (Gradient: Hexane to 5-6% EtOAc: Hexanes) yields with 34% (after oxidation of C-Bpin bond) with >95:5 dr and 80:20 (b/l ratio) as colorless oil compound.

^{1}H NMR (500 MHz, CDCl$_3$) δ 7.31 (d, *J* = 4.4 Hz, 4H), 7.25 – 7.20 (m, 1H), 7.17 (td, *J* = 7.4, 1.6 Hz, 2H), 6.91 – 6.80 (m, 2H), 6.25 (ddd, *J* = 17.0, 10.0, 9.2 Hz, 1H), 5.35 (ddd, *J* = 17.0, 1.7, 0.8 Hz, 1H), 5.19 (dd, *J* = 10.0, 1.8 Hz, 1H), 5.02 (dd, *J* = 10.1, 2.9 Hz, 1H), 4.28 (dd, *J* = 11.0, 9.2 Hz, 1H), 4.16 (d, *J* = 10.1 Hz, 1H), 3.84 (s, 3H), 3.66 – 3.51 (m, 2H), 3.41 (dd, *J* = 11.0, 2.8 Hz, 1H), 0.64 (t, *J* = 7.1 Hz, 3H).
^{13}C NMR (126 MHz, CDCl$_3$) δ 174.4, 157.6, 143.3, 137.7, 129.8, 129.3, 128.3, 128.2, 127.2, 125.5, 120.7, 117.9, 111.2, 71.7, 60.2, 55.6, 54.7, 46.8, 13.6
HRMS(ESI): Calculated for $C_{21}H_{24}O_4Na$ [M+Na]: 363.1566, Found: 363.1560
HPLC: >99.5:0.5 enantiomeric ratio; Phenomenex Lux 3u Cellulose-1 column using 95:5 Hexane: IPA as gradient, 1.0 mL/min, 220 nm. τ_{minor} = 7.92 min and τ_{major} = 9.34 min.

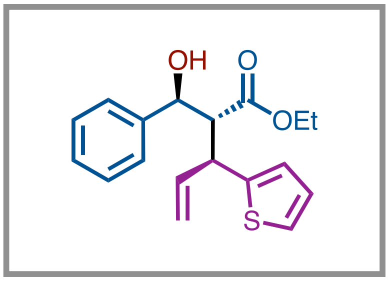

ethyl (2R,3S)-2-((S)-hydroxy(phenyl)methyl)-3-(thiophen-2-yl) pent-4-enoate (26)
The title compound was prepared according to General Procedure B with 79% NMR yield and 95:5 dr by ^{1}HNMR analysis of crude reaction mixture before C-Bpin oxidation. Purification by silica-gel flash column chromatography (Gradient: Hexane to 5-6% EtOAc: Hexanes) yields with 76% (after oxidation of C-Bpin bond) with >98:2 dr as colorless oil compound.
^{1}H NMR (500 MHz, CDCl$_3$) δ 7.36 – 7.28 (m, 4H), 7.27 – 7.22 (m, 1H), 7.17 (dd, *J* = 5.1, 1.2 Hz, 1H), 6.93 – 6.83 (m, 2H), 6.08 (ddd, *J* = 16.9, 10.0, 9.0 Hz, 1H), 5.38 (dt, *J* = 16.9, 1.1 Hz, 1H), 5.27 (dd, *J* = 10.1, 1.3 Hz, 1H), 5.05 (dd, *J* = 9.9, 3.2 Hz, 1H), 4.32 (dd, *J* = 10.5, 9.1 Hz, 1H), 3.94 (d, *J* = 9.9 Hz, 1H), 3.80 – 3.66 (m, 2H), 3.06 (dd, *J* = 10.5, 3.2 Hz, 1H), 0.78 (t, *J* = 7.1 Hz, 3H).
^{13}C NMR (126 MHz, CDCl$_3$) δ 173.7, 144.3, 142.4, 137.9, 128.4, 127.5, 126.7, 125.4, 124.9, 124.2, 118.1, 71.7, 60.7, 58.3, 45.3, 13.7
HRMS(ESI): Calculated for $C_{18}H_{20}SO_3Na$ [M+Na]: 339.1025, Found: 339.1020
HPLC: >99.5:0.5 enantiomeric ratio; Phenomenex Lux 3u Cellulose-1 column using 98:2 Hexane: IPA as gradient, 0.5 mL/min, 220 nm. τ_{minor} = 34.85 min and τ_{major} = 36.75 min.

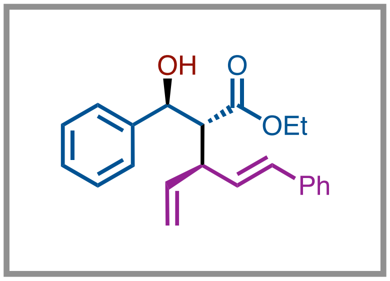

ethyl (2R,3R, E)-2-((S)-hydroxy(phenyl)methyl)-5-phenyl-3-vinylpent-4-enoate (27)

The title compound was prepared according to General Procedure B with 70% NMR yield and 94:6 dr by ^{1}HNMR analysis of crude reaction mixture before C-Bpin oxidation. Purification by silica-gel flash column chromatography (Gradient: Hexane to 5-6% EtOAc: Hexanes) yields with 66% (after oxidation of C-Bpin bond) with >95:5 dr as colorless oil.

^{1}H NMR (500 MHz, CDCl$_3$) δ 7.48 – 6.88 (m, 10H), 6.27 (d, *J* = 15.8 Hz, 1H), 6.06 (dd, *J* = 15.8, 8.8 Hz, 1H), 5.79 (ddd, *J* = 17.6, 10.2, 7.9 Hz, 1H), 5.17 (dt, *J* = 17.1, 1.3 Hz, 1H), 5.10 (dd, *J* = 10.4, 1.4 Hz, 1H), 4.87 (dd, *J* = 8.5, 4.8 Hz, 1H), 3.95 – 3.79 (m, 2H), 3.55 (d, *J* = 8.6 Hz, 1H), 3.31 (q, *J* = 8.6 Hz, 1H), 2.80 (dd, *J* = 8.9, 4.8 Hz, 1H), 0.90 (t, *J* = 7.1 Hz, 3H).

^{13}C NMR (126 MHz, CDCl$_3$) 174.0, 142.3, 137.6, 137.2, 131.9, 128.63, 128.61, 128.5, 127.7, 127.6, 126.4, 125.9, 117.4, 72.3, 60.7, 56.6, 47.4, 14.2

HRMS(ESI): Calculated for $C_{22}H_{24}O_3Na$ [M+Na]: 359.1617, Found: 359.1611

HPLC: 96.5:3.5 enantiomeric ratio; Phenomenex Lux 3u Cellulose-1 column using 95:5 Hexane: IPA as gradient, 1.0 mL/min, 220 nm. τ_{minor} = 11.66 min and τ_{major} = 20.33 min

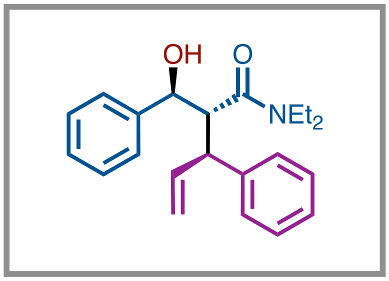

(2R,3S)-N, N-diethyl-2-((S)-hydroxy(phenyl)methyl)-3-phenylpent-4-enamide (28)

The title compound was prepared according to General Procedure B with 69% NMR yield and 95:5 dr by ^{1}HNMR analysis of crude reaction mixture before C-Bpin oxidation. Purification by silica-gel flash column chromatography (Gradient: Hexane to 7-8% EtOAc: Hexanes) yields with 51% (after oxidation of C-Bpin bond) with >98:2 dr as white solid.

^{1}H NMR (500 MHz, CDCl$_3$) δ 7.45 – 6.96 (m, 10H), 6.31 – 6.19 (m, 2H), 5.47 (dd, *J* = 17.0, 1.5 Hz, 1H), 5.34 (dd, *J* = 10.1, 1.5 Hz, 1H), 5.07 (dd, *J* = 9.2, 2.3 Hz, 1H), 4.13 (dd, *J* = 11.0, 9.4 Hz, 1H), 3.13 (dq, *J* = 14.0, 7.1 Hz, 1H), 2.99 (dd, *J* = 11.0, 2.3 Hz, 1H), 2.65 (dq, *J* = 14.1, 7.1 Hz, 1H), 2.56 (dq, *J* = 14.5, 7.2 Hz, 1H), 2.21 (dq, *J* = 14.5, 7.2 Hz, 1H), 0.58 (t, *J* = 7.1 Hz, 3H), 0.11 (t, *J* = 7.2 Hz, 3H).

^{13}C NMR (126 MHz, CDCl$_3$) δ 172.7, 144.3, 141.8, 137.7, 128.5, 128.32, 128.30, 127.2, 126.8, 125.7, 118.5, 72.7, 52.6, 50.5, 41.5, 39.6, 12.7, 12.1

HRMS(ESI): Calculated for $C_{22}H_{27}NO_2Na$ [M+Na]: 360.1934, Found: 360.1928

HPLC: 99:1 enantiomeric ratio; Phenomenex Lux 3u Cellulose-3 column using 95:5 Hexane: IPA as gradient, 1.0 mL/min, 220 nm. τ_{minor} = 5.76 min and τ_{major} = 6.88 min

9. Stereodivergent synthesis to access four major stereoisomers:

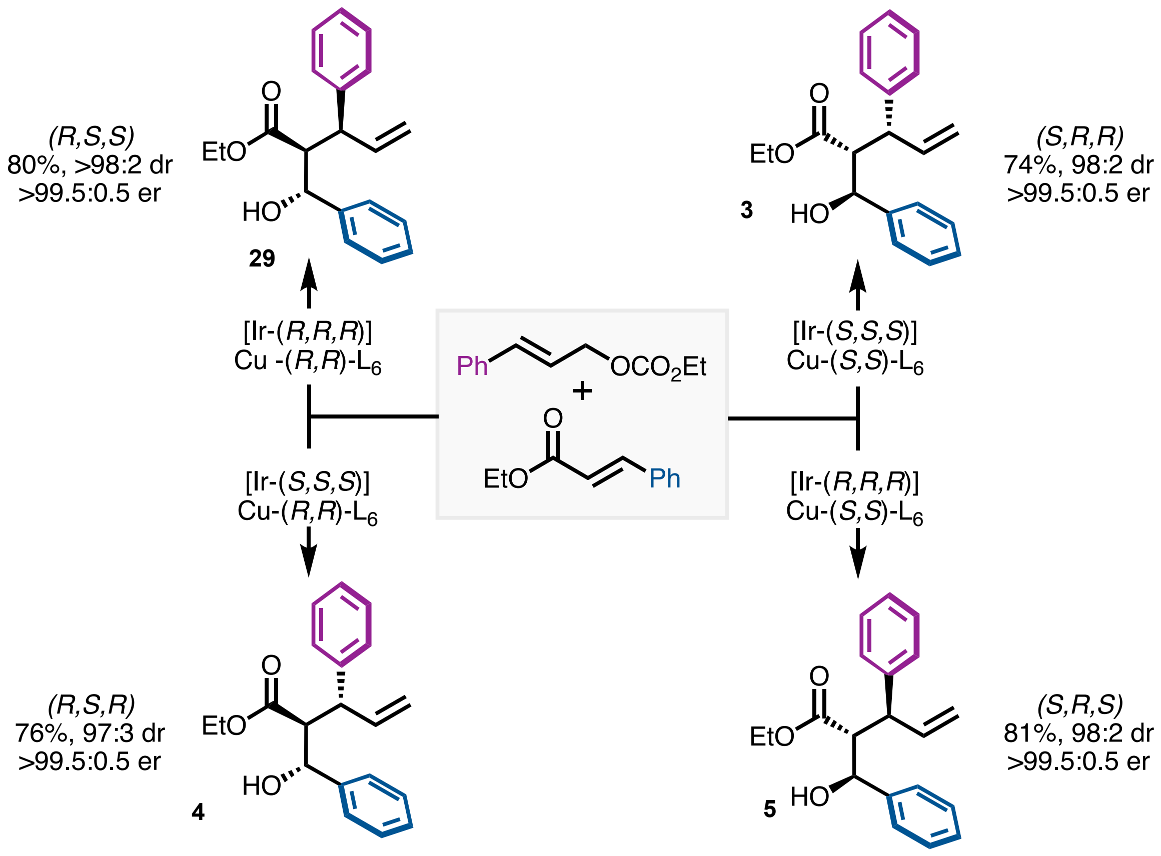

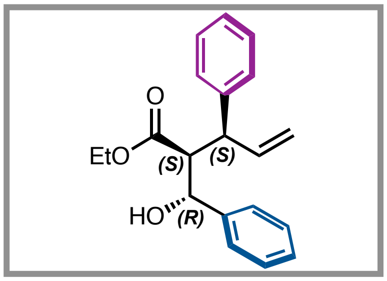

ethyl (2S,3S)-2-((R)-hydroxy(phenyl)methyl)-3-phenylpent-4-enoate (29)
The title compound was prepared according to General Procedure B using **[Ir-(*R,R,R*)]** catalyst and **(*R,R*)-FOXAP ligand with Cu(MeCN)$_4$PF$_6$** as the catalysts pair with >98:2 dr by ^{1}HNMR analysis of crude reaction mixture before C-Bpin oxidation. Purification by silica-gel flash column chromatography (Gradient: Hexane to 5-6% EtOAc: Hexanes) yields with 80% (after oxidation of C-Bpin bond) with >98:2 dr as white powder compound.
^{1}H NMR, ^{13}C NMR were matched with the and HRMS data were mathched compound (3)

HPLC: >99.5:0.5 enantiomeric ratio; ChiralPak IA-3 column using 95:5 Hexane: IPA as gradient, 1.0 mL/min, 220 nm. τ_{major} = 11.70 min and τ_{minor} = 16.28 min.

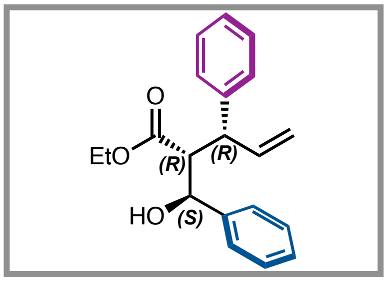

ethyl (2R,3R)-2-((S)-hydroxy(phenyl)methyl)-3-phenylpent-4-enoate (3)

The title compound was prepared according to General Procedure B using **[Ir-(*S,S,S*)]** catalyst and **(*S,S*)-FOXAP ligand with Cu(MeCN)$_4$PF$_6$** as the catalysts pair with 98:2 dr by ^{1}HNMR analysis of crude reaction mixture before C-Bpin oxidation. Purification by silica-gel flash column chromatography (Gradient: Hexane to 5-6% EtOAc: Hexanes) yields with 74% (after oxidation of C-Bpin bond) with >98:2 dr as white powder compound.

^{1}H NMR (500 MHz, CDCl$_3$) δ 7.57 – 6.88 (m, 10H), 5.96 (ddd, *J* = 16.9, 10.1, 9.0 Hz, 1H), 5.02 (dt, *J* = 17.0, 1.2 Hz, 1H), 4.95 (dd, *J* = 10.1, 1.5 Hz, 1H), 4.38 (s, 1H), 3.93 – 3.83 (m, 3H), 3.73 – 3.60 (m, 1H), 3.02 (dd, *J* = 10.9, 3.4 Hz, 1H), 0.90 (t, *J* = 7.1 Hz, 3H).
^{13}C NMR (126 MHz, CDCl$_3$) δ 174.2, 142.5, 1401.0, 138.6, 129.1, 128.3, 128.1, 127.4, 127.2, 125.3, 116.5, 71.6, 60.7, 57.7, 50.2, 14.1
HRMS (ESI): Calculated for $C_{20}H_{22}O_3Na$ [M+Na]: 333.1461, Found: 333.1457
HPLC: >99.5:0.5 enantiomeric ratio; ChiralPak IA-3 column using 95:5 Hexane: IPA as gradient, 1.0 mL/min, 220 nm. τ_{minor} = 11.91 min and τ_{major} = 16.13 min.

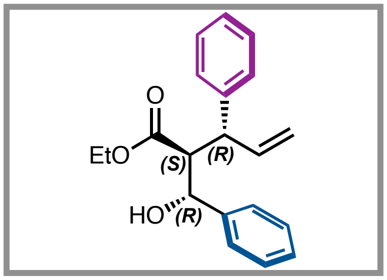

ethyl (2S,3R)-2-((R)-hydroxy(phenyl)methyl)-3-phenylpent-4-enoate (4)

The title compound was prepared according to General Procedure B using **[Ir-(*S,S,S*)]** catalyst and **(*R,R*)-FOXAP ligand with Cu(MeCN)$_4$PF$_6$** as the catalysts pair with 97:3 dr by ^{1}HNMR analysis of crude reaction mixture before C-Bpin oxidation. Purification by silica-gel flash column chromatography (Gradient: Hexane to 5-6% EtOAc: Hexanes) yields with 76% (after oxidation of C-Bpin bond) with >98:2 dr as colorless oil compound.

^{1}H NMR, ^{13}C NMR were matched with the and HRMS data were mathched compound (3)
HPLC: >99.5:0.5 enantiomeric ratio; ChiralPak IA-3 column using 95:5 Hexane: IPA as gradient, 1.0 mL/min, 220 nm. τ_{minor} = 10.89 min and τ_{major} = 17.94 min.

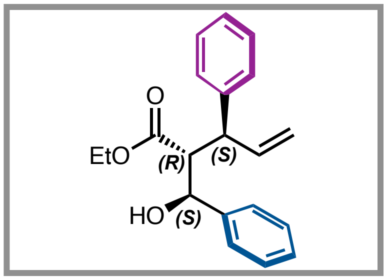

^{1}H NMR, ^{13}C NMR were matched with the and HRMS data were mathched compound (5)
ethyl (2R,3S)-2-((S)-hydroxy(phenyl)methyl)-3-phenylpent-4-enoate (5)
The title compound was prepared according to General Procedure B **[Ir-(*R,R,R*)]** catalyst and **(*S,S*)-FOXAP ligand with Cu(MeCN)$_4$PF$_6$** as the catalysts pair with >98:2 dr by ^{1}HNMR analysis of crude reaction mixture before C-Bpin oxidation. Purification by silica-gel flash column chromatography (Gradient: Hexane to 5-6% EtOAc:Hexanes) yields with 84% (after oxidation of C-Bpin bond) with >98:2 dr as colorless semi-solid compound.
^{1}H NMR (500 MHz, CDCl$_3$) δ 7.35 – 7.10 (m, 10H), 6.04 (ddd, *J* = 16.9, 10.1, 9.1 Hz, 1H), 5.32 (dt, *J* = 17.0, 1.0 Hz, 1H), 5.20 (dd, *J* = 10.1, 1.4 Hz, 1H), 5.02 (dd, *J* = 10.1, 2.9 Hz, 1H), 4.10 – 3.82 (m, 2H), 3.53 (qq, *J* = 7.3, 3.6 Hz, 2H), 3.04 (dd, *J* = 11.1, 3.0 Hz, 1H), 0.57 (t, *J* = 7.1 Hz, 3H).
^{13}C NMR (126 MHz, CDCl$_3$) δ 174.0, 142.8, 141.1, 138.3, 128.6, 128.4, 128.2, 127.4, 127.0, 125.4, 117.9, 71.7, 60.4, 57.2, 50.2, 13.5
HRMS (ESI): Calculated for $C_{20}H_{22}O_3Na$ [M+Na]: 333.1461, Found: 333.1455
HPLC: >99.5:0.5 enantiomeric ratio; ChiralPak IA-3 column using 95:5 Hexane: IPA as gradient, 1.0 mL/min, 220 nm. τ_{major} = 10.79 min and τ_{minor} = 18.26 min.

Unsuccessful substrates:

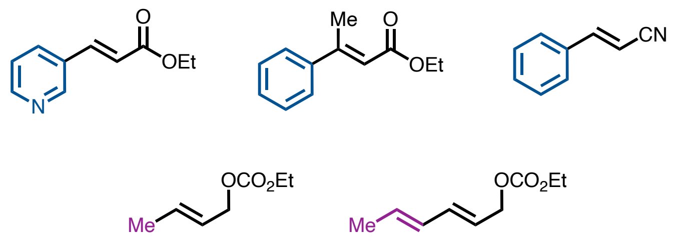

10. (a) Scale-up synthesis:

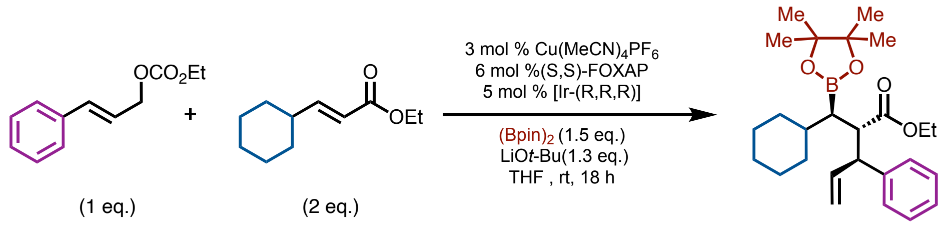

ethyl (2S,3S)-2-((R)-cyclohexyl(4,4,5,5-tetramethyl-1,3,2-dioxaborolan-2-yl) methyl)-3-phenylpent-4-enoate (30)

In an N_2-filled glovebox, to a flame-dried 50 mL round-bottom flask with a magnetic stir bar, was added [Ir-(*R,R,R*)]-Complex (52.5 mg, 50.0 µmol, 5.0 mol%), B_2pin_2 (0.38 g, 1.5 mmol, 1.5 eq.), LiO*t*Bu (104 mg, 1.3 mmol, 1.3 eq.) was added in that order. Then in a separate flame dried 2-dram vial equipped with a magnetic stir bar was added $Cu(MeCN)_4PF_6$ (11.2 mg, 30 µmol, 3.0 mol%), (*S,S*)-FOXAP (29 mg, 60.0 µmol, 6.0 mol%). Then both the vial anf flask were sealed with a rubber septum and lined with Teflon tape, removed from the glove box, and placed under a positive pressure of N_2. After that 4 ml of freshly prepared THF was added to the vial and stirred at room temperature for 10 minutes. In the meantime, to a separate vial the electrophile, cinnamyl ethyl carbonate (1.0 mmol, 1.0 eq.) and the alkene (2.0 mmol, 2.0 eq.) was added and dissolved in 11 mL of THF. Then the substrate solution was added to the reaction flask rinsing the sides of the vial followed by the Cu-catalyst solution. *(Note: it is important that the vial is stirring vigorously while the liquid reagents are added to prohibit aggregation of the solid reagents, which typically results in lower yields).* Next, the reaction was stirred at room temperature for 18 hours. After 18 hours, the reaction was quenched with sat. NH_4Cl (15 mL), the two phases were separated, and the aqueous phase was back extracted with diethyl ether (15 mL × 2). The combined organic phases were dried over anhydrous sodium sulfate and concentrated in-vacuo. The organic residue further purified by flash column chromatography, 1-2% Ethyl acetate in hexane afford the product as colorless oil (which further solidified after keeping in the freezer for 24h) 56% yield and >95:5 dr.

^{1}H NMR (500 MHz, $CDCl_3$) δ 7.23 (td, *J* = 7.3, 1.5 Hz, 2H), 7.18 – 7.11 (m, 3H), 5.98 (ddd, *J* = 17.0, 10.2, 8.9 Hz, 1H), 5.21 (ddd, *J* = 17.0, 1.9, 0.9 Hz, 1H), 5.12 (dd, *J* = 10.3, 1.9 Hz, 1H), 3.88 – 3.76 (m, 2H), 3.72 (dq, *J* = 10.8, 7.1 Hz, 1H), 3.02 (dd, *J* = 10.2, 5.0 Hz, 1H), 2.00 – 1.92 (m, 1H), 1.80 – 1.59 (m, 4H), 1.36 – 1.08 (m, 17H), 1.00 – 0.90 (m, 2H), 0.84 (t, *J* = 7.1 Hz, 3H).

^{13}C NMR (126 MHz, $CDCl_3$) δ 174.7, 142.5, 140.0, 128.2, 128.1, 126.4, 116.7, 82.9, 59.7, 53.4, 51.5, 50.0, 37.1, 32.9, 32.1, 26.7, 26.63, 26.62, 25.5, 24.5, 13.8

HRMS(ESI): Calculated for $C_{26}H_{39}BO_4Na$ [M+Na]: 449.2833, Found: 449.2828

(b) Further Transformation:

A. Benzoylation:

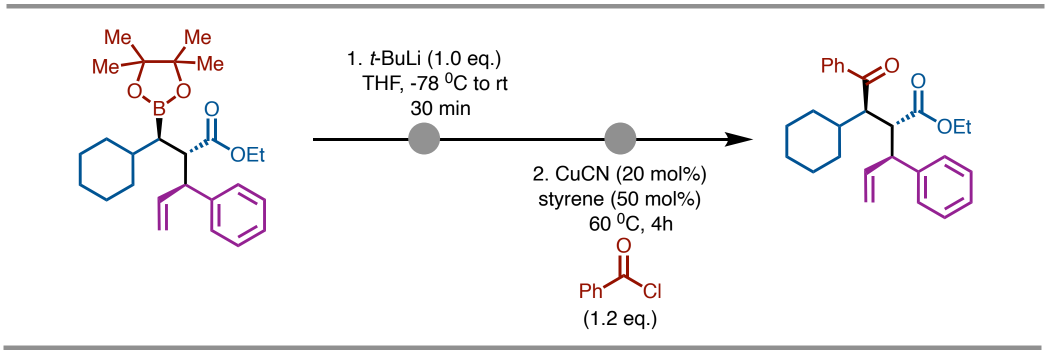

ethyl (2R,3S)-2-((R)-1-cyclohexyl-2-oxo-2-phenylethyl)-3-phenylpent-4-enoate (31)

The title compound was synthesized in accordance with the following modified literature procedure[20]: In a flame-dried 2-dram vial equipped with a magnetic stir bar was added *ethyl (2S,3S)-2-((R)-cyclohexyl(4,4,5,5-tetramethyl-1,3,2-dioxaborolan-2-yl)methyl)-3-phenylpent-4-enoate* (35 mg, 1 eq., 82 µmol) then the vial was sealed with a septum and evacuated-backfilled with N_2 three times, followed by THF (0.2 mL) was added. The vial was cooled to -78 °C and tert-butyllithium (51 µL, 1.6 M, 1 eq., 82 µmol) was added dropwise by a syringe. The vial was then allowed to warm to room temperature and further stirred for 30 minutes. The reaction vial was then transferred into the glove box, styrene (4.7 µL, 0.5 eq., 41 µmol) CuCN (1.5 mg, 0.2 eq., 16 µmol) and Benzoyl chloride (14 mg, 11 µL, 1.2 eq., 98 µmol) were added. The vial was sealed with septum cap and removed from the glove box. The septum was replaced with Teflon coated cap and heated to 60 °C (oil bath) and stirred for 4 hours. The reaction mixture was subsequently diluted with diethyl ether and was passed through a silica gel plug using diethyl ether as eluent. The solvent was removed under reduced pressure. The crude product was purified by column chromatography 51% Yield >95:5 dr (silica gel, 4-5% EtOAc in Hexanes; R_f= 0.3 in 10% EtOAc:Hexane) to affored the desired product.

^{1}H NMR (500 MHz, $CDCl_3$) δ 7.90 – 7.84 (m, 2H), 7.57 – 7.50 (m, 1H), 7.42 (dd, *J* = 8.4, 7.1 Hz, 2H), 7.30 – 7.23 (m, 2H), 7.23 – 7.10 (m, 3H), 6.10 (ddd, *J* = 17.0, 10.1, 8.8 Hz, 1H), 5.11 (dd, *J* = 10.3, 1.4 Hz, 1H), 4.96 (dt, *J* = 17.0, 1.2 Hz, 1H), 3.86 (qq, *J* = 10.7, 7.1 Hz, 2H), 3.77 (t, *J* = 6.7 Hz, 1H), 3.55 (t, *J* = 8.6 Hz, 1H), 3.47 (dd, *J* = 8.3, 6.7 Hz, 1H), 2.00 (tdt, *J* = 11.9, 6.3, 2.9 Hz, 1H), 1.90 (dt, *J* = 13.6, 3.1 Hz, 1H), 1.76 – 1.57 (m, 5H), 1.18 – 1.00 (m, 2H), 0.99 – 0.78 (m, 5H).

^{13}C NMR (126 MHz, $CDCl_3$) δ 203.6, 172.6, 141.9, 139.9, 139.8, 132.8, 128.5, 128.5, 128.5, 128.0, 126.8, 116.6, 60.4, 51.3, 50.5, 50.0, 38.8, 32.7, 30.0, 27.0, 26.5, 26.4, 13.9

HRMS(ESI): Calculated for $C_{27}H_{32}34Na$ [M+Na]: 427.2243, Found: 427.22.35

B. **Alkynylation:**

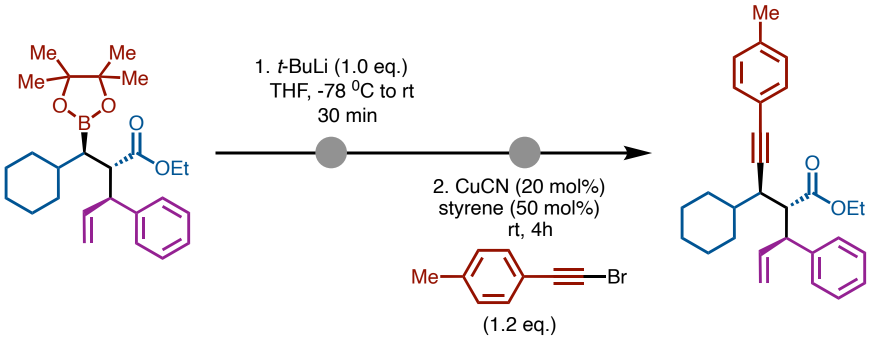

ethyl (2R,3S)-2-((R)-1-cyclohexyl-3-(p-tolyl) prop-2-yn-1-yl)-3-phenylpent-4-enoate (32)

The title compound was synthesized in accordance with the following modified literature procedure (ref.-20): A flame-dried 2-dram vial equipped with a stir bar was charged with *ethyl (2S,3S)-2-((R)-cyclohexyl(4,4,5,5-tetramethyl-1,3,2-dioxaborolan-2-yl)methyl)-3-phenylpent-4-enoate* (30 mg, 1 eq., 70 µmol) and THF (0.2 mL) and evacuated and backfilled with N_2 x 3. The reaction mixture was then cooled to -78 ºC and tert-butyllithium (47 µL, 1.5 M, 1 eq., 70 µmol) was added dropwise by a syringe. The reaction mixture was then allowed to warm to room temperature and stirred for 30 minutes. The reaction vial was transferred into the glovebox, styrene (4.0 µL, 0.5 eq., 35 µmol) (must be added before electrophile), copper(I) cyanide (1.3 mg, 0.2 eq., 14 µmol) and 1-(bromoethynyl)-4-methylbenzene (16 mg, 1.2 eq., 84 µmol)) were added. The vial was sealed with septum cap and removed from the glovebox. The vial was stirred for 12 hours at 25 ºC. The reaction mixture was diluted with diethyl ether and was passed through a silica gel plug with diethyl ether as eluent. The solvent was removed under reduced pressure. The crude product was purified by Combi Flash column chromatography to furnish the desired product 79%, >95:5 dr as pale-yellow oil.

^{1}H NMR (500 MHz, CDCl$_3$) δ 7.42 – 7.28 (m, 2H), 7.27 (m, 4H), 7.23 – 7.14 (m, 1H), 7.12 (d, *J* = 7.8 Hz, 2H), 5.87 (dt, *J* = 16.9, 9.8 Hz, 1H), 5.35 – 5.26 (m, 1H), 5.19 – 5.07 (m, 1H), 4.00 (dd, *J* = 11.3, 9.4 Hz, 1H), 3.85 (q, *J* = 7.1 Hz, 2H), 3.08 (dd, *J* = 11.3, 4.3 Hz, 1H), 2.72 (dd, *J* = 9.2, 4.4 Hz, 1H), 2.35 (s, 3H), 2.23 – 2.15 (m, 1H), 2.10 (dd, *J* = 12.3, 3.4 Hz, 1H), 1.80 – 1.72 (m, 2H), 1.69 – 1.63 (m, 1H), 1.46 (tdt, *J* = 12.5, 6.8, 2.3 Hz, 1H), 1.25 – 0.97 (m, 5H), 0.94 (t, *J* = 7.1 Hz, 3H)

^{13}C NMR (126 MHz, CDCl$_3$) δ 171.8, 142.2, 139.1, 137.6, 131.6, 129.1, 128.6, 128.1, 126.7, 121.3, 117.01, 88.0, 85.1, 59.9, 51.8, 50.6, 40.23, 40.22, 32.0, 31.4, 26.6, 26.4, 26.3, 21.6, 14.1

HRMS(ESI): Calculated for $C_{29}H_{34}O_2Na$ [M+Na]: 437.2451, Found: 437.2444

C. Amination:

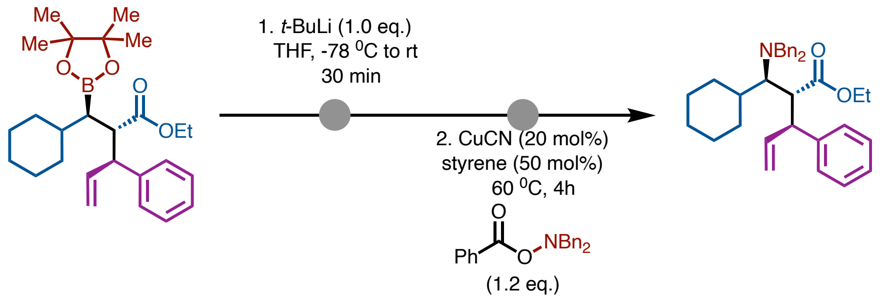

ethyl (2R,3S)-2-((R)-cyclohexyl(dibenzylamino)methyl)-3-phenylpent-4-enoate (33)
The title compound was synthesized in accordance with the following modified literature procedure (ref.-20): In a flame-dried 2-dram vial equipped with a magnetic stir bar was added *ethyl (2S,3S)-2-((R)-cyclohexyl(4,4,5,5-tetramethyl-1,3,2-dioxaborolan-2-yl) methyl)-3-phenylpent-4-enoate* (35 mg, 1 eq., 82 µmol). The vial was capped with a rubber septum and coated with Teflon. After evacuating and backfilled with N2 (X 3), THF (0.2 mL) was added to the reaction vial under N_2 atmosphere. The vial was cooled to -78 °C and tert-butyllithium (51 µL, 1.6 M, 1 eq., 82 µmol) was added dropwise by a syringe. The vial was then allowed to warm to room temperature and further stirred for 30 minutes. The reaction vial was then transferred into the glove box, styrene (4.7 µL, 0.5 eq., 41 µmol) CuCN (1.5 mg, 0.2 eq., 16 µmol), cesium fluoride (25 mg, 2 eq., 0.16 mmol) and O-benzoyl-N,N-dibenzylhydroxylamine (31 mg, 1.2 eq., 98 µmol) were added. The vial was sealed with plastic cap and removed from the glove box. The vial was heated to 60 °C (oil bath) and stirred for 4 hours. The reaction mixture was subsequently diluted with diethyl ether and was passed through a silica gel plug using diethyl ether as eluent. The solvent was removed under reduced pressure. The crude product was purified by column chromatography 64% yield and >95:5 dr (silica gel, 2-3% EtOAc in Hexanes; R_f= 0.6 in 10% EtOAc:Hexane) to furnish the desired product.
^{1}H NMR (500 MHz, $CDCl_3$) δ 7.49 – 7.40 (m, 4H), 7.32 (t, *J* = 7.5 Hz, 5H), 7.28 – 7.12 (m, 6H), 5.46 (ddd, *J* = 17.2, 10.3, 8.5 Hz, 1H), 4.70 (dd, *J* = 10.3, 1.4 Hz, 1H), 4.44 – 4.33 (m, 1H), 3.95 – 3.80 (m, 2H), 3.80 – 3.71 (m, 4H), 3.65 (dq, *J* = 10.8, 7.0 Hz, 1H), 3.24 (dd, *J* = 10.9, 3.6 Hz, 1H), 3.07 (dd, *J* = 6.7, 3.5 Hz, 1H), 2.00 – 1.84 (m, 3H), 1.67-1.82 (s, 3H), 1.33 – 1.14 (m, 5H), 0.86 – 0.77 (m, 3H).
^{13}C NMR (126 MHz, $CDCl_3$) δ 173.4, 143.6, 140.6, 129.5, 128.4, 128.2, 128.0, 126.9, 126.2, 115.4, 63.8, 60.0, 55.8, 50.1, 40.1, 32.8, 32.1, 26.8, 26.6, 22.8, 13.6
HRMS(ESI): Calculated for $C_{34}H_{42}O_2N$ [M+H]: 496.3210, Found: 496.3198

D. **Zweifel Olefination:**

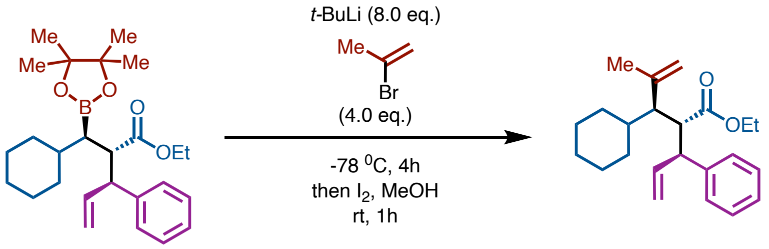

ethyl (2S,3R)-3-cyclohexyl-4-methyl-2-((S)-1-phenylallyl) pent-4-enoate (34)
The product was synthesized according to the following modified literature procedure[21]: A flame-dried 2-dram vial under N_2 was charged with 2-bromoprop-1-ene (25 µL, 4 eq., 0.28 mmol), THF (1.5 mL) was added, and the solution was cooled to -78 °C in a dry ice/acetone bath. *t*-BuLi (solution in pentane) (0.35 mL, 1.59 M, 8 eq., 0.56 mmol) was added dropwise and the solution stirred at -78 °C for 1 h. A solution of *ethyl (2S,3S)-2-((R)-cyclohexyl(4,4,5,5-tetramethyl-1,3,2-dioxaborolan-2-yl)methyl)-3-phenylpent-4-enoate* (30 mg, 1 eq., 70 µmol) in THF (0.4 mL) was added dropwise at -78 °C and the mixture was allowed to stir at -78 °C for 3 h. A solution of Iodine (71 mg, 4 eq., 0.28 mmol) in MeOH (0.3 mL) was added dropwise down the side of the flask and the reaction was stirred at -78 °C for 30 minutes then allowed to warm to room temperature and stirred for 1 h. The reaction was quenched upon the addition of saturated $Na_2S_2O_3$ solution (3.0 mL) and stirred for another 1 h. The organic layer was separated, and the aqueous phase extracted with EtOAc (3 x 3 mL). The combined organic layers were washed with brine (4.0 mL), and organic phase reextracted, dried over anhydrous sodium sulfate and concentrated under reduced pressure. Crude material was purified by silica gel column chromatography 0-1% ethyl acetate in Hexane afforded the product as colorless oil, 75% yield and >95:5 dr.
^{1}H NMR (500 MHz, $CDCl_3$) δ 7.25 – 7.11 (m, 5H), 5.83 (ddd, *J* = 17.1, 10.2, 9.1 Hz, 1H), 5.14 – 5.03 (m, 2H), 4.95 (dt, *J* = 2.8, 1.4 Hz, 1H), 4.80 (d, *J* = 2.4 Hz, 1H), 3.84 (dq, *J* = 10.8, 7.1 Hz, 1H), 3.77 – 3.71 (m, 1H), 3.70 – 3.65 (m, 1H), 3.26 (dd, *J* = 11.0, 4.5 Hz, 1H), 2.33 (dd, *J* = 10.0, 4.6 Hz, 1H), 2.28 – 2.19 (m, 1H), 1.85 – 1.75 (m, 1H), 1.74 – 1.60 (m, 6H), 1.35 – 1.11 (m, 4H), 1.03 – 0.89 (m, 4H), 0.89 – 0.76 (m, 1H).
^{13}C NMR (126 MHz, $CDCl_3$) δ 173.0, 143.7, 143.7, 140.8, 128.5, 127.8, 126.3, 116.4, 115.8, 59.8, 53.0, 50.3, 48.9, 37.3, 31.9, 31.7, 26.8, 26.6, 26.5, 20.7, 13.9
HRMS(ESI): Calculated for $C_{23}H_{32}O_2Na$ [M+Na]: 363.2294, Found: 363.2284

A. **Matteson Homologation:**

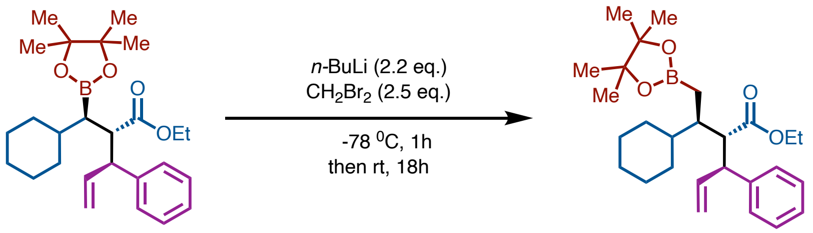

ethyl (2S,3S)-2-((R)-1-cyclohexyl-2-(4,4,5,5-tetramethyl-1,3,2-dioxaborolan-2-yl) ethyl)-3-phenylpent-4-enoate (35)

The product was synthesized according to the following modified literature procedure[21]: A flame-dried 2-dram vial equipped with a stir bar was charged with *ethyl (2S,3S)-2-((R)-cyclohexyl(4,4,5,5-tetramethyl-1,3,2-dioxaborolan-2-yl)methyl)-3-phenylpent-4-enoate* (45 mg, 1 eq., 0.11 mmol) and evacuated and backfilled with N_2 x 3, then THF (1.65 mL) added followed by CH_2Br_2 (18 µL, 2.5 eq., 0.26 mmol) added sequentially via syringe and the mixture was cooled to -78 ^{0}C in a dry ice/acetone bath. n-BuLi (in hexane) (0.15 mL, 1.58 M, 2.2 eq., 0.23 mmol) was added drop wise via syringe over 10 minutes. The reaction was stirred at -78 °C for 60 minutes and then warmed to room temperature and stirred for 18 h. The reaction was quenched with H_2O (5 mL), the layers were separated, and the aqueous layer was extracted with ethyl acetate (3 X 3 mL). The combined organic layers were dried over anhydrous sodium sulfate and concentrated in vacuo. Purification via Combi Flash Column chromatography (gradient: 1-2% ethyl acetate in hexane) yield 67%, >95:5 dr as colorless oil.

^{1}H NMR (500 MHz, $CDCl_3$) δ 7.26 – 7.12 (m, 5H), 5.84 (dt, *J* = 16.9, 9.8 Hz, 1H), 5.12 (dd, *J* = 17.0, 1.7 Hz, 1H), 5.06 (dd, *J* = 10.1, 1.7 Hz, 1H), 3.87 – 3.73 (m, 2H), 3.74 – 3.66 (m, 1H), 3.05 (dd, *J* = 11.5, 2.9 Hz, 1H), 2.06 – 1.92 (m, 2H), 1.78 – 1.58 (m, 4H), 1.25 (d, *J* = 1.7 Hz, 12H), 1.22 – 0.94 (m, 7H), 0.91 (t, *J* = 7.1 Hz, 3H), 0.70 (dd, *J* = 16.6, 9.2 Hz, 1H)

^{13}C NMR (126 MHz, $CDCl_3$) δ 173.3, 143.1, 139.8, 128.5, 128.0, 126.5, 116.5, 82.9, 59.6, 51.7, 51.3, 43.0, 39.7, 31.4, 30.6, 26.9, 26.83, 26.82, 25.1, 24.8, 14.1

HRMS(ESI): Calculated for $C_{27}H_{41}BO_4Na$ [M+Na]: 463.2990, Found: 463.2990

E. Ester reduction:

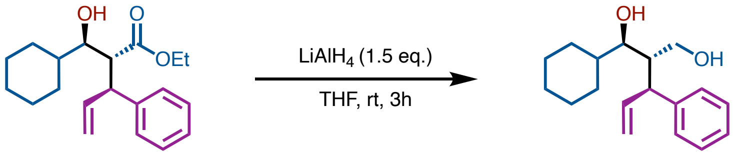

(1R,2S)-1-cyclohexyl-2-((S)-1-phenylallyl) propane-1,3-diol (36)

The product was synthesized according to the following procedure: The compound *ethyl (2R,3S)-2-((R)-cyclohexyl(hydroxy)methyl)-3-phenylpent-4-enoate* (25 mg, 0.08 mmol, 1.0 eq.) was taken in 2-dram vial under N_2 atmosphere containing $LiAlH_4$ (4.8 mg, 0.13 mmol, 1.0 eq.) in 1.3 mL of THF and stirred for 3h. After stirring for 3 h, H_2O (2 mL/mmol of substrate) and 1.0 N HCl (2 mL/mmol of substrate) solution at 0 ^{0}C were added and the solutions were extracted with Et_2O (3 x 3 mL). The extracts were dried over anhydrous Na_2SO_4 and concentrated in vacuo. The residue was purified via silica gel column chromatography (R_f= 0.5 in 40% EtOAc:Hexane) 15%-20% EtOAc in hexanes to afford the desired product with 68% yield and >20:1 dr as white solid.

^{1}H NMR (500 MHz, $CDCl_3$) δ 7.44 – 7.13 (m, 5H), 6.00 (dt, *J* = 17.0, 9.8 Hz, 1H), 5.20 (dd, *J* = 16.9, 1.8 Hz, 1H), 5.09 (dd, *J* = 10.0, 1.7 Hz, 1H), 3.92 – 3.84 (m, 2H), 3.71 – 3.60 (m, 1H), 3.32 (d, *J* = 11.2 Hz, 1H), 2.66 – 2.08 (m, 3H), 1.89 (dd, *J* = 10.9, 2.6 Hz, 1H), 1.85 – 1.56 (m, 6H), 1.23 – 1.10 (m, 1H), 1.05 – 0.82 (m, 3H).
^{13}C NMR (126 MHz, $CDCl_3$) δ 143.5, 140.9, 128.9, 128.0, 126.6, 116.1, 77.5, 60.8, 49.00, 44.4, 41.6, 30.1, 29.4, 26.5, 26.1, 26.1
HRMS(ESI): Calculated for $C_{18}H_{26}O_2Na$ [M+Na]: 297.1825, Found: 297.1820

F. **Hydroboration:**

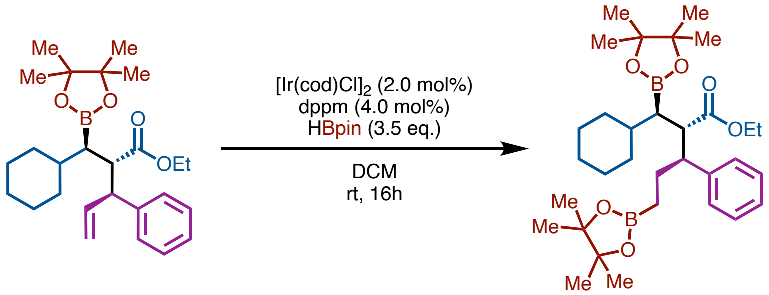

ethyl (2S,3S)-2-((R)-cyclohexyl(4,4,5,5-tetramethyl-1,3,2-dioxaborolan-2-yl) methyl)-3-phenyl-5-(4,4,5,5-tetramethyl-1,3,2-dioxaborolan-2-yl) pentanoate (37)

The product was synthesized according to the following modified literature procedure[22]: In a flame-dried 2-dram vial equipped with a stir bar was charged with *ethyl (2S,3S)-2-((R)-cyclohexyl(4,4,5,5-tetramethyl-1,3,2-dioxaborolan-2-yl)methyl)-3-phenylpent-4-enoate* (30 mg, 1 eq., 70 µmol) was taken along with $[Ir(cod)Cl]_2$ (1.3 mg, 0.03 eq., 2.0 µmol), and diphenylphosphanylmethyl(diphenyl)phosphane (dppm) (1.5 mg, 0.06 eq., 4.0 µmol) under a positive pressure of N_2. Then 1.5 mL of DCM was added to it, followed by the addition of Pinacolatoborane (HBpin) (34 µL, 3.3 eq., 0.23 mmol) and the resulting solution was stirred at room temperature for 16 h. After, the reaction mixture was concentrated under reduced pressure and the residue was purified by silica gel flash column chromatography 2-4% ethyl acetate in Hexane afforded the product as colorless oil, 82% yield and >95:5 dr.

^{1}H NMR (500 MHz, $CDCl_3$) δ 7.22 – 7.15 (m, 2H), 7.15 – 7.04 (m, 1H), 7.08 – 7.02 (m, 2H), 3.89 – 3.74 (m, 2H), 3.00 – 2.87 (m, 2H), 2.06 (ddd, J = 11.1, 5.9, 3.7 Hz, 1H), 1.89 – 1.47 (m, 8H), 1.30 – 1.18 (m, 26H), 1.15 – 0.99 (m, 2H), 0.95 (t, J = 7.2 Hz, 3H), 0.92 – 0.83 (m, 1H), 0.70 – 0.52 (m, 2H).

^{13}C NMR (126 MHz, $CDCl_3$) δ 175.2, 142.4, 128.9, 127.9, 126.3, 83.0, 82.9, 59.7, 50.0, 48.4, 36.9, 32.9, 32.3, 27.9, 27.0, 27.0, 26.9, 25.3, 25.0, 25.0, 24.8, 14.1

HRMS(ESI): Calculated for $C_{32}H_{53}B_2O_6Na$ [M+H]: 555.4022, Found: 555.4025

11. Formal synthesis of (+)-β-Lycorane:

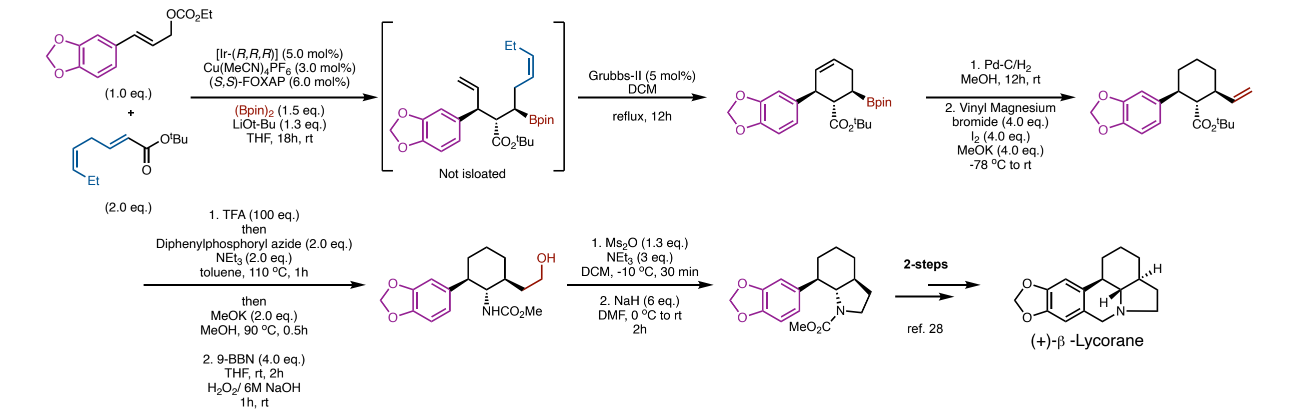

Synthesis:

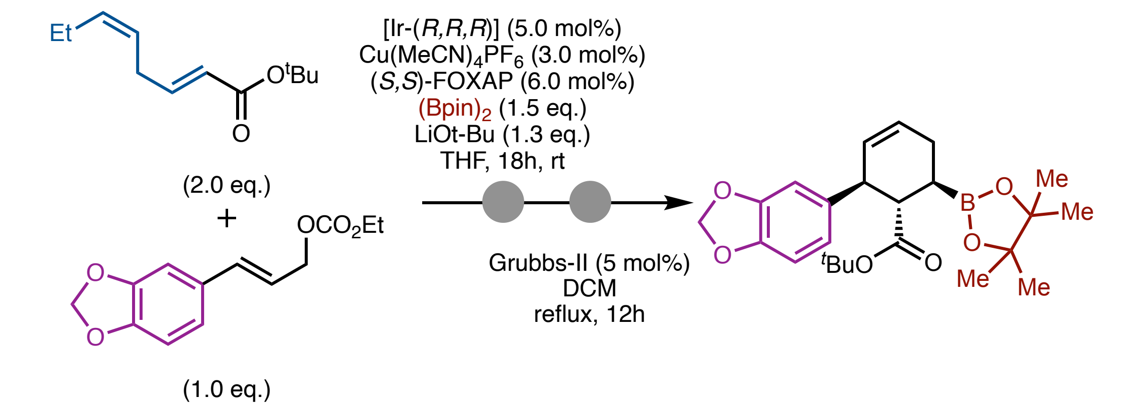

tert-butyl (1S,2S,6R)-2-(benzo[d][1,3]dioxol-5-yl)-6-(4,4,5,5-tetramethyl-1,3,2-dioxaborolan-2-yl)cyclohex-3-ene-1-carboxylate

In an N_2-filled glovebox, to a flame-dried 50 mL round-bottom flask with a magnetic stir bar, was added [Ir-(*R,R,R*)]-Complex (52.5 mg, 50.0 μmol, 5.0 mol%), B_2pin_2 (0.38 g, 1.5 mmol, 1.5 eq.), LiO*t*Bu (104 mg, 1.3 mmol, 1.3 eq.) was added in that order. Then in a separate flame dried 2-dram vial equipped with a magnetic stir bar was added $Cu(MeCN)_4PF_6$ (11.2 mg, 30 μmol, 3.0 mol%), (*S,S*)-FOXAP (29 mg, 60.0 μmol, 6.0 mol%). Then both the vial and the flask were sealed with a rubber septum and lined with Teflon tape, removed from the glove box, and placed under a positive pressure of N_2. After that 4.0 mL of freshly prepared THF was added to the vial and stirred at room temperature for 10 minutes. In the meantime, to a separate vial the electrophile, cinnamyl ethyl carbonate (1.0 mmol, 1.0 eq.) and the alkene (2.0 mmol, 2.0 eq.) was added and dissolved in 11 mL of THF. Then the substrate solution was added to the reaction flask rinsing the sides of the vial followed by the Cu-catalyst solution. *(Note: it is important that the vial is stirring vigorously*

while the liquid reagents are added to prohibit aggregation of the solid reagents, which typically results in lower yields). Next, the reaction was stirred at room temperature for 18 hours. After 18 hours, the reaction was quenched with sat. NH_4Cl (15 mL), the two phases were separated, and the aqueous phase was back extracted with diethyl ether (15 mL × 2). The combined organic phases were dried through silica gel plug and concentrated in-vacuo. The crude organic residue further purified by flash column chromatography (R_f= 0.5 in 10% ethyl acetate in hexane). But due to some unknown impurity in the same R_f value the product the characterization was unsuccessful in this step, just collecting all the fraction on that R_f value and concentrated and subjected to next step.

The crude from last step was taken into 2-dram vial, was added Grubbs catalyst (2nd generation, 8.4 mg, 0.01 mmol, 5.0 mol %), followed by anhydrous DCM (0.05 M), then the vial sealed with electrical tape and stirred at 50 ^{0}C for 12h. Then the solvent was evaporated, and crude material was purified by silica-gel flash column chromatography (R_f= 0.35 in 10% EtOAc:Hexane) (3-5% ethyl acetate in hexane) afforded the product as light-yellow oil 46% and >95:5 dr.

^{1}H NMR (500 MHz, $CDCl_3$) δ 6.72 – 6.64 (m, 2H), 6.62 (dd, *J* = 7.9, 1.7 Hz, 1H), 5.92 – 5.86 (m, 2H), 5.80 (ddt, *J* = 10.0, 5.0, 2.5 Hz, 1H), 5.55 (dq, *J* = 9.9, 2.1 Hz, 1H), 3.39 (ddq, *J* = 9.2, 4.7, 2.4 Hz, 1H), 2.44 (dd, *J* = 12.2, 10.5 Hz, 1H), 2.26 – 2.05 (m, 2H), 1.62 – 1.51 (m, 1H), 1.29 – 1.17 (m, 21H).
^{13}C NMR (126 MHz, $CDCl_3$) δ 174.8, 147.4, 146.0, 138.2, 130.5, 126.8, 121.6, 108.7, 107.8, 100.7, 83.3, 79.9, 50.4, 46.7, 28.0, 26.2, 24.7, 24.7
HRMS(ESI): Calculated for $C_{24}H_{33}O_6BNa$ [M+Na]: 451.2262, Found: 451.2262

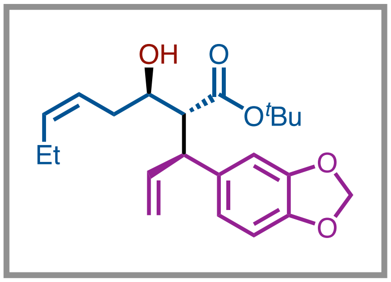

tert-butyl (2R,3R,Z)-2-((S)-1-(benzo[d][1,3]dioxol-5-yl)allyl)-3-hydroxyoct-5-enoate(40)
The title compound was prepared according to General Procedure B using **[Ir-(*R,R,R*)] and (*S,S*)-FOXAP** ligand on Cu-catalyst with 70% NMR yield and 95:5 dr by ^{1}HNMR analysis of crude reaction mixture before C-Bpin oxidation. Purification by silica-gel flash column chromatography (Gradient: Hexane to 3-5% EtOAc:Hexanes) yields with 45% (after oxidation of C-Bpin bond) with >95:5 dr as colorless oil compound.
^{1}H NMR (500 MHz, $CDCl_3$) δ 6.76 – 6.64 (m, 3H), 5.94 – 5.89 (m, 2H), 5.84 (ddd, *J* = 16.9, 10.0, 8.9 Hz, 1H), 5.57 – 5.47 (m, 1H), 5.43 – 5.34 (m, 1H), 5.20 (dt, *J* = 16.9, 1.2 Hz, 1H), 5.11 (dd, *J* = 10.0, 1.5 Hz, 1H), 3.84 – 3.75 (m, 2H), 3.32 (d, *J* = 10.5 Hz, 1H), 2.66 (dd, *J* = 11.4, 2.3 Hz, 1H), 2.40 – 2.30 (m, 1H), 2.18 (dtd, *J* = 14.5, 7.5, 1.3 Hz, 1H), 2.05 (pd, *J* = 7.5, 1.6 Hz, 2H), 1.17 (s, 9H), 0.97 (t, *J* = 7.5 Hz, 3H).

^{13}C NMR (126 MHz, CDCl$_3$) δ 173.8, 147.7, 146.4, 138.9, 135.5, 134.5, 124.5, 121.7, 117.0, 108.9, 108.3, 101.0, 81.8, 70.3, 54.1, 49.7, 34.8, 27.9, 20.9, 14.4
HPLC: 95:5 enantiomeric ratio; Phenomenex Lux 3u Cellulose-1 column using 98:2 Hexane: IPA as gradient, 0.5 mL/min, 220 nm. τ_{minor} = 12.24 min and τ_{major} = 17.04 min.

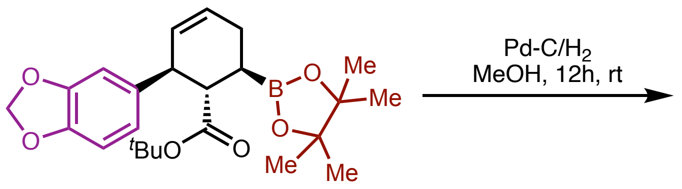

tert-butyl (1S,2S,6R)-2-(benzo[d][1,3]dioxol-5-yl)-6-(4,4,5,5-tetramethyl-1,3,2-dioxaborolan-2-yl)cyclohexane-1-carboxylate

The product was synthesized according to the following modified literature procedure[23]. The title compound was made according to the following procedure. In a flame-dried 25 mL round-bottom flask, equipped with a stir bar was cooled under vacuum. *tert-butyl (1S,2S,6R)-2-(benzo[d][1,3]dioxol-5-yl)-6-(4,4,5,5-tetramethyl-1,3,2-dioxaborolan-2-yl)cyclohex-3-ene-1-carboxylate* (190 mg, 1 eq., 0.45 mmol) and Pd/C 10% (90 mg) were added into the vial. After evacuated/backfilled with N_2 (X 3), dry MeOH (4.5 mL) was added. After degassing the solution with H_2 using a needle connected to H_2 balloon, the reaction mixture was stirred at room temperature for 12 h with H_2 balloon attached. After that, the reaction mixture was filtered throught a pad of celite. The reaction mixture was concentrated under high vaccum obtain the product (R_f= 0.35 in 10% EtOAc:Hexane) as an pale yellow oil >95% crude yield, >95:5 dr which was further subjected to next step without purification.

^{1}H NMR (500 MHz, CDCl$_3$) δ 6.74 – 6.61 (m, 3H), 5.88 (d, J = 1.7 Hz, 2H), 2.55 (td, J = 11.4, 2.9 Hz, 1H), 2.39 (t, J = 11.1 Hz, 1H), 1.89 – 1.81 (m, 3H), 1.50 – 1.26 (m, 4H), 1.21 (d, J = 3.9 Hz, 12H), 1.13 (s, 9H).
^{13}C NMR (126 MHz, CDCl$_3$) δ 174.4, 147.4, 145.9, 139.2, 121.1, 108.5, 107.9, 100.8, 83.3, 79.56, 52.1, 48.7, 35.1, 27.9, 27.3, 27.0, 24.9, 24.8
HRMS(ESI): Calculated for $C_{24}H_{35}O_6BNa$ [M+Na]: 453.2418, Found: 453.2419

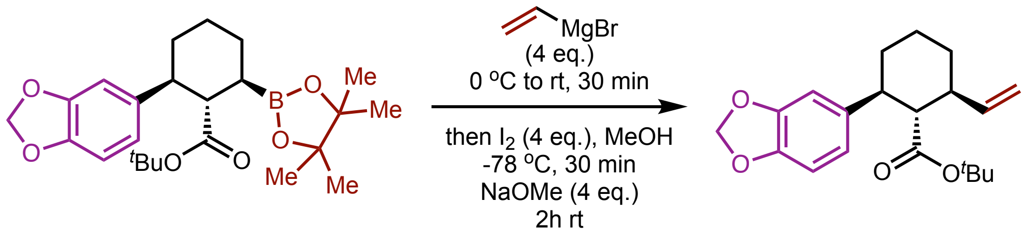

tert-butyl (1S,2S,6S)-2-(benzo[d][1,3]dioxol-5-yl)-6-vinylcyclohexane-1-carboxylate

The title compound was prepared according to a known procedure with slight modification[24]. In a flame-dried 25 mL round-bottom flask, equipped with a stir bar was cooled under vacuum., *tert-butyl (1S,2S,6R)-2-(benzo[d][1,3]dioxol-5-yl)-6-(4,4,5,5-tetramethyl-1,3,2-dioxaborolan-2-yl)cyclohexane-1-carboxylate* (145 mg, 1.0 eq., 0.34 mmol) and anhydrous THF (3.5 mL) were added under nitrogen atmosphere. Then, the solution was cooled to 0 °C and vinylmagnesium bromide (1.35 mL, 1.0 M in THF, 4 eq., 1.35 mmol) was slowly added. The mixture was warmed to room temperature and stirred for 30 min. The reaction was then cooled to -78 °C, and iodine (342 mg, 4.0 eq., 1.35 mmol) in anhydrous Methanol (0.7 mL) was slowly added and stirred for 30 min at this temperature. Then, Sodium methoxide (72.8 mg, 4.0 eq., 1.35 mmol) in anhydrous Methanol (1.3 mL) was added and the reaction mixture and allowed to warm to room temperature. After stirring for 2 h, a saturated aqueous solution of $Na_2S_2O_3$ (10 mL) was added. The reaction mixture was diluted with water, and the aqueous layer was extracted with EtOAc (10 mL x 2), the combined organic phase was dried over sodium sulfate, concentrated and purified by silica gel column (R_f= 0.5 in 10% EtOAc:Hexane) to afford the product with 60% yield after two steps and >95:5 dr.

^{1}H NMR (500 MHz, CDCl$_3$) δ 6.82 – 6.44 (m, 3H), 5.93 – 5.86 (m, 2H), 5.69 (ddd, *J* = 17.1, 10.3, 8.1 Hz, 1H), 5.05 (ddd, *J* = 17.1, 1.8, 0.9 Hz, 1H), 4.94 (dd, *J* = 10.2, 1.8 Hz, 1H), 2.68 (td, *J* = 11.7, 3.4 Hz, 1H), 2.33 (tdd, *J* = 11.5, 8.1, 3.4 Hz, 1H), 2.19 (t, *J* = 11.0 Hz, 1H), 1.89 – 1.77 (m, 3H), 1.57 – 1.35 (m, 2H), 1.31 – 1.18 (m, 1H), 1.15 (s, 9H).
^{13}C NMR (126 MHz, CDCl$_3$) δ 173.3, 147.5, 146.0, 141.0, 138.3, 121.0, 114.7, 108.2, 108.0, 100.8, 80.0, 56.4, 47.1, 45.5, 34.2, 31.8, 28.0, 25.8
HRMS(ESI): Calculated for $C_{20}H_{26}O_4Na$ [M+Na]: 353.1728, Found: 353.1732

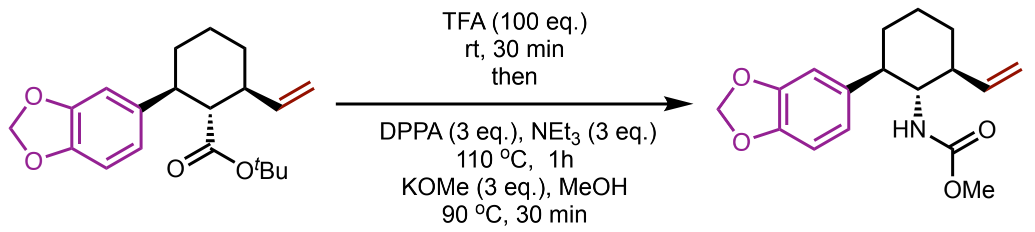

methyl ((1S,2R,6S)-2-(benzo[d][1,3]dioxol-5-yl)-6-vinylcyclohexyl)carbamate

The title compound was prepared according to a known procedure with slight modification[25]. In a flame dried 2-dram vial equipped with a stir bar was added *tert-butyl (1S,2S,6S)-2-(benzo[d][1,3]dioxol-5-yl)-6-vinylcyclohexane-1-carboxylate* (65 mg, 1 eq., 0.20 mmol) in Trifluoroacetic acid (1.5 mL, 100 eq., 20 mmol) at room temperature. The mixture was stirred for 0.5 h at room temperature. Then the reaction mixture was concentrated in vacuo to obtain a brown oil. Which was further dissolve in 0.5 mL of diethyl ether and 2 mL of hexane, then the solution was sonicated for 1 minute and concentrated under high vaccum. This process was repeated for another three times until a foam compound was observed and almost no TFA left. Then the residue was dissolve in tolune (0.8 mL) under N_2 atmosphere, followed by triethylamine (82 µL, 3 eq., 0.59 mmol) and Diphenylphosphoryl azide (0.13 mL, 3 eq., 0.59 mmol). The reaction mixture was

then heated to reflux (110 °C) and stirred at this temperature for 1 hour. The resulting solution was concentrated in vacuo. To this was added Methanol (4 mL) and Potassiummethoxide (41 mg, 3 eq., 0.59 mmol). The reaction mixture was heated to reflux (90 °C) for 30 min before cooling to room temperature. The reaction mixture was quenched with saturated NH_4Cl aq. (4 mL) and extracted with EtOAc (5 x 4 mL). The combined organic solution was dried over sodium sulfate, concentrated and purified by silica gel column (R_f= 0.65 in 40% EtOAc:Hexane) to afford the product as amorphous solid with 57% yield after two steps and >95:5 dr.

^{1}H NMR (500 MHz, $CDCl_3$) δ 6.74 – 6.59 (m, 3H), 5.91 (q, *J* = 1.5 Hz, 2H), 5.79 (dt, *J* = 17.8, 9.5 Hz, 1H), 5.07 – 4.95 (m, 2H), 4.35 – 4.01 (m, 1H), 3.46 (s, 4H), 2.55 – 2.22 (m, 1H), 2.15 – 1.95 (m, 1H), 1.93 – 1.75 (m, 3H), 1.58 – 1.33 (m, 3H).
^{13}C NMR (126 MHz, $CDCl_3$) δ 156.7, 147.7, 146.1, 140.9, 137.8, 120.6, 115.3, 108.2, 107.9, 100.9, 57.8, 52.0, 50.6, 50.4, 35.5, 32.6, 25.7
HRMS(ESI): Calculated for $C_{17}H_{21}O_4NNa$ [M+Na]: 326.1362, Found: 326.1357

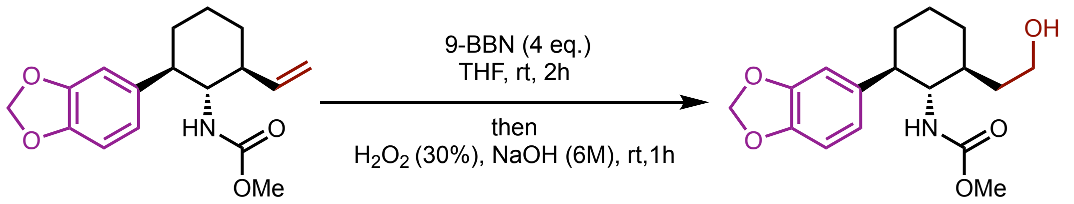

methyl ((1S,2R,6S)-2-(benzo[d][1,3]dioxol-5-yl)-6-(2-hydroxyethyl)cyclohexyl)carbamate
The product was synthesized according to the following modified literature procedure[26]. In a flame dride 2-dram vial was added methyl *((1S,2R,6S)-2-(benzo[d][1,3]dioxol-5-yl)-6-vinylcyclohexyl)carbamate* (15 mg, 1 Eq, 49 µmol) in dry THF (0.5 mL) under N_2-atmosphere. Then 9-BBN (0.40 mL, 0.5 M in THF , 4 eq., 0.20 mmol) was added and the mixture was stirred at room temperature for 2 h. Then 0.4 mL an aqueous solution of NaOH (6.0 M) and 0.4 mL H_2O_2 (30 % in water) were added at 0 ºC and the reaction mixture was stirred for 1 h at room temperature. The reaction was quenched with brine and the mixture extracted with ethyl acetate (3x3 mL). The combined organic solution was dried over sodium sulfate, concentrated and purified by silica gel column (R_f= 0.25 in 5% MeOH:DCM) to afford the product as an oil with 85% yield and >95:5 dr.

^{1}H NMR (500 MHz, $CDCl_3$) δ 6.85 – 6.52 (m, 3H), 5.90 (q, *J* = 1.5 Hz, 2H), 4.33 (d, *J* = 9.6 Hz, 1H), 3.85 – 3.61 (m, 2H), 3.42-3.55 (m, 3H), 2.37 (td, *J* = 11.6, 3.6 Hz, 1H), 1.98 – 1.76 (m, 5H), 1.67 – 1.30 (m, 6H).
^{13}C NMR (126 MHz, $CDCl_3$) δ 157.1, 147.6, 145.9, 137.8, 120.5, 108.1, 107.8, 100.8, 71.8, 60.47, 58.6, 52.0, 51.4, 41.4, 35.4, 34.9, 30.9, 25.8
HRMS(ESI): Calculated for $C_{17}H_{23}O_5NNa$ [M+Na]: 344.1474, Found: 344.1476

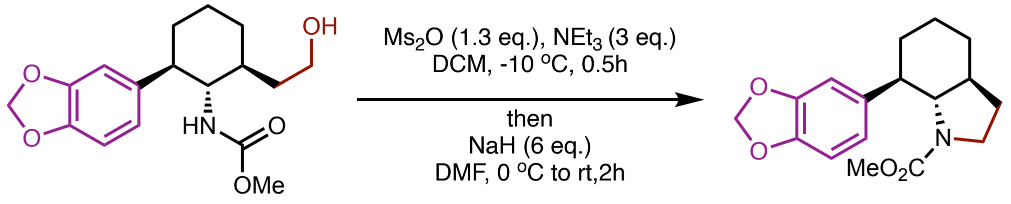

methyl (3aS,7R)-7-(benzo[d][1,3]dioxol-5-yl)octahydro-1H-indole-1-carboxylate

The product was synthesized according to the following modified literature procedure[27]. In a flame dride 2-dram vial was added *methyl ((1S,2R,6S)-2-(benzo[d][1,3]dioxol-5-yl)-6-(2-hydroxyethyl)cyclohexyl)carbamate* (12 mg, 1 eq., 37 µmol) and dissolved in DCM (0.8 mL) followed by triethylamine (16 µL, 3 eq., 0.11 mmol). Then a solution of Ms_2O (9.8 mg, 1.5 eq., 56 µmol) in DCM (0.8 mL) were added at -10 °C. The mixture was stirred for 30 minutes at this temperature and the resulting mixture was diluted with DCM (3 mL) and washed with saturated $NaHCO_3$ (3 mL) and brine (3 mL). the mixture extracted with ethyl acetate (3x3 mL). The combined organic solution was dried over sodium sulfate, concentrated under reduced pressure. The crude was used in the next without further purification. In a flame-dried, 2-dram vial with a magnetic stirring bar was added NaH (60% in dispersion in mineral oil, 4.3 g, 0.18 mmol, 6 eq.) and 0.2 mL of anhydrous DMF. The mixture then paced into the 0 °C ice-bath. In a separate 1-dram the crude from last step was dissolved in 0.2 mL DMF andded slowly to the solution of NaH in DMF at 0 °C. the reaction mixture was warmed to room temperature and stirred for further 2 h. The reaction was quenched by the slow addition of 1 mL of water to yield an oily residue, which was extracted with diethyl ether (3x3 mL). The combined organic solution was dried over anhydrous sodium sulfate, concentrated and purified by silica gel column chromatography (R_f= 0.75 in 40% EtOAc:Hexane) to afford the product as semi-solid with 60% yield over two steps and >95:5 dr. The ^{1}H NMR and ^{13}C NMR data are matched with the literature reported compound[28].

^{1}H NMR (500 MHz, $CDCl_3$) δ 6.75 – 6.67 (m, 2H), 6.61 (dd, J = 8.0, 1.7 Hz, 1H), 5.89 (dd, J = 11.8, 1.5 Hz, 2H), 3.73 (dd, J = 10.9, 7.7 Hz, 1H), 3.30 (m, 1H), 3.05-3.09 (m, 4H), 2.59-2.64 (m, 1H), 1.95-1.99(m, 1H), 1.94 – 1.85 (m, 2H), 1.84 – 1.74 (m, 2H), 1.67-171 (m 1H), 1.51 – 1.39 (m, 2H), 1.33 (m, 1H).

^{13}C NMR (126 MHz, $CDCl_3$) δ 157.8, 147.1, 145.3, 139.7, 120.5, 107.9, 107.5, 100.6, 68.4, 51.7, 51.3, 49.5, 49.0, 33.8, 30.3, 29.7, 26.5

12. **X-ray structure:**

A single crystal was obtained by slow evaporation from DCM/pentane solution. A colorless crystal (approximate dimensions 0.230 × 0.172 × 0.172 mm^3) was placed onto the tip of a MiTeGen loop and mounted on a Bruker Venture D8 diffractometer equipped with a PhotonIII detector at 173(2) K.

Compound-23

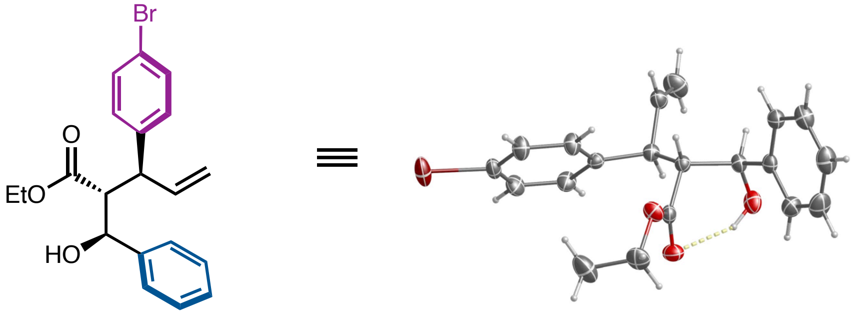

checkCIF/PLATON report

Structure factors have been supplied for datablock(s) 25003

THIS REPORT IS FOR GUIDANCE ONLY. IF USED AS PART OF A REVIEW PROCEDURE FOR PUBLICATION, IT SHOULD NOT REPLACE THE EXPERTISE OF AN EXPERIENCED CRYSTALLOGRAPHIC REFEREE.

No syntax errors found. CIF dictionary Interpreting this report

Datablock: 25003

```
Bond precision:  C-C = 0.0036 A        Wavelength=0.71073

Cell:             a=10.6686(5)     b=12.9319(7)    c=13.5490(7)
                  alpha=90         beta=90          gamma=90
Temperature:      173 K

                Calculated                 Reported
Volume          1869.29(17)                1869.29(17)
Space group     P 21 21 21                 P 21 21 21
Hall group      P 2ac 2ab                  P 2ac 2ab
Moiety formula  C20 H21 Br O3             C20 H21 Br O3
Sum formula     C20 H21 Br O3             C20 H21 Br O3
Mr              389.27                     389.28
Dx,g cm-3       1.383                      1.383
Z               4                          4
Mu (mm-1)       2.213                      2.213
F000            800.0                      800.0
F000'           799.19
h,k,lmax        14,17,18                   14,17,18
Nref            5063[ 2857]                5026
Tmin,Tmax       0.507,0.730                0.596,0.746
Tmin'           0.497
Correction method= # Reported T Limits: Tmin=0.596 Tmax=0.746
AbsCorr = MULTI-SCAN
Data completeness= 1.76/0.99     Theta(max)= 29.155

R(reflections)= 0.0280( 4626)                  wR2(reflections)=
                                                0.0721( 5026)
S = 1.058                Npar= 220
```

The following ALERTS were generated. Each ALERT has the format **test-name_ALERT_alert-type_alert-level**. Click on the hyperlinks for more details of the test.

```
 Alert level C
PLAT911_ALERT_3_C Missing FCF Refl Between Thmin & STh/L=    0.600          6 Report
                8  0  0,    8  0  2,    6  3  4,    0  8  4,    6  0  5,    0  3  9,
PLAT987_ALERT_1_C The Flack x is >> 0 -  Do a BASF/TWIN Refinement     Please Check
```

```
 Alert level G
PLAT007_ALERT_5_G Number of Unrefined Donor-H Atoms .............           1 Report
              H3O
PLAT033_ALERT_4_G Flack x Value Deviates > 3.0 * Sigma from Zero .      0.024 Note
PLAT093_ALERT_1_G No s.u.'s on H-positions, Refinement Reported as      mixed Check
PLAT791_ALERT_4_G Model has Chirality at C3         (Sohncke SpGr)          S Verify
PLAT791_ALERT_4_G Model has Chirality at C4         (Sohncke SpGr)          R Verify
PLAT791_ALERT_4_G Model has Chirality at C14        (Sohncke SpGr)          S Verify
PLAT969_ALERT_5_G The 'Henn et al.' R-Factor-gap value ..........      2.095 Note
              Predicted wR2: Based on SigI**2  3.44 or SHELX Weight  6.81
PLAT978_ALERT_2_G Number C-C Bonds with Positive Residual Density.          8 Info
```

```
   0 ALERT level A = Most likely a serious problem - resolve or explain
   0 ALERT level B = A potentially serious problem, consider carefully
   2 ALERT level C = Check. Ensure it is not caused by an omission or oversight
   8 ALERT level G = General information/check it is not something unexpected

   2 ALERT type 1 CIF construction/syntax error, inconsistent or missing data
   1 ALERT type 2 Indicator that the structure model may be wrong or deficient
   1 ALERT type 3 Indicator that the structure quality may be low
   4 ALERT type 4 Improvement, methodology, query or suggestion
   2 ALERT type 5 Informative message, check
```

It is advisable to attempt to resolve as many as possible of the alerts in all categories. Often the minor alerts point to easily fixed oversights, errors and omissions in your CIF or refinement strategy, so attention to these fine details can be worthwhile. In order to resolve some of the more serious problems it may be necessary to carry out additional measurements or structure refinements. However, the purpose of your study may justify the reported deviations and the more serious of these should normally be commented upon in the discussion or experimental section of a paper or in the "special_details" fields of the CIF. checkCIF was carefully designed to identify outliers and unusual parameters, but every test has its limitations and alerts that are not important in a particular case may appear. Conversely, the absence of alerts does not guarantee there are no aspects of the results needing attention. It is up to the individual to critically assess their own results and, if necessary, seek expert advice.

Publication of your CIF in IUCr journals

A basic structural check has been run on your CIF. These basic checks will be run on all CIFs submitted for publication in IUCr journals (*Acta Crystallographica*, *Journal of Applied Crystallography*, *Journal of Synchrotron Radiation*); however, if you intend to submit to *Acta Crystallographica Section C* or *E* or *IUCrData*, you should make sure that full publication checks are run on the final version of your CIF prior to submission.

Publication of your CIF in other journals

Please refer to the *Notes for Authors* of the relevant journal for any special instructions relating to CIF submission.

PLATON version of 19/12/2024; check.def file version of 19/12/2024

Datablock 25003 - ellipsoid plot

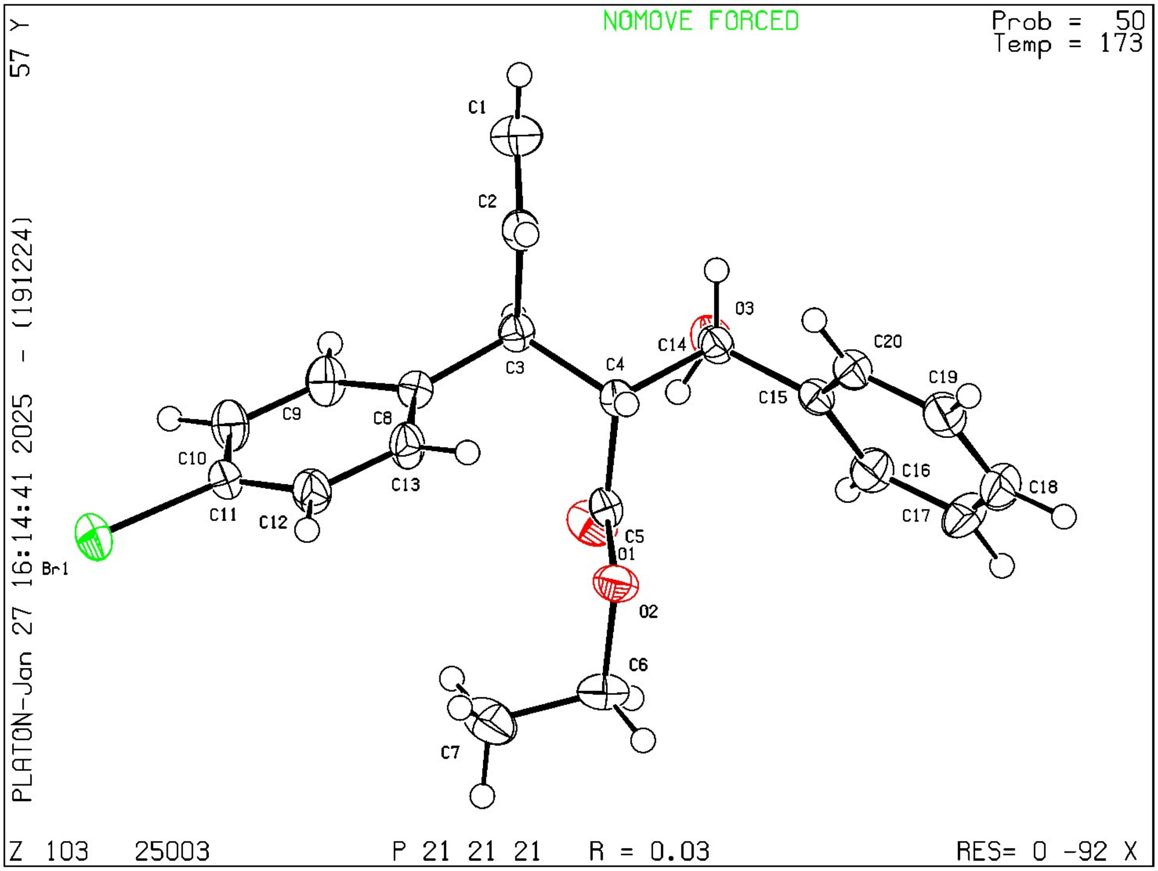

INDIANA UNIVERSITY DEPARTMENT OF CHEMISTRY

Molecular Structure Center

Report No. 25003
(KB-SD-III-708-B)

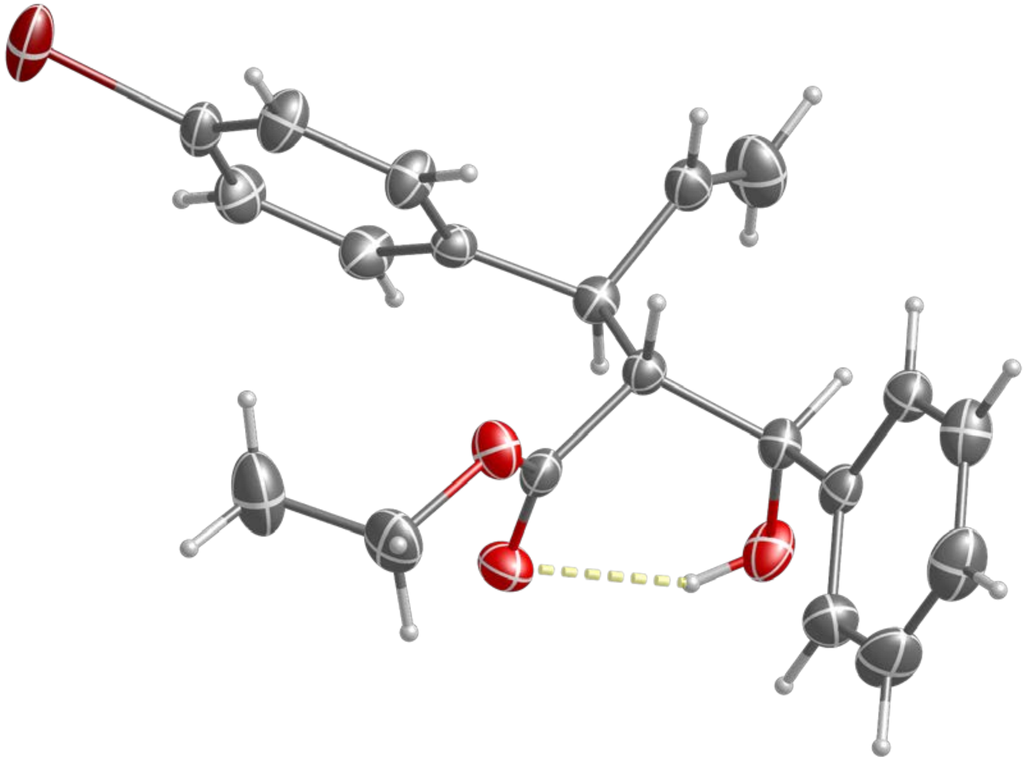

$C_{26}H_{33}BO_2$

Prepared for
Suman Das and Professor Kevin Brown

by M. Pink, January 27, 2025

The sample was submitted by Suman Das (research group of Kevin Brown, Department of Chemistry, Indiana University). A colorless crystal (approximate dimensions 0.230 × 0.172 × 0.172 mm^3) was placed onto the tip of a MiTeGen loop and mounted on a Bruker Venture D8 diffractometer equipped with a PhotonIII detector at 173(2) K.

Data collection

The data collection was carried out using Mo K_α radiation (multilayer mirror monochromator) with a frame time of 7 and 0.7 seconds and a detector distance of 4.00 cm. A collection strategy was calculated and complete data to a resolution of 0.77 Å (eight sets of frames) were collected with 1° ω and φ scans. A total of 1350 frames were collected. The total exposure time was 1.44 hours. The frames were integrated with the SAINT V8.41 package using a narrow-frame algorithm.[1] The integration of the data using an orthorhombic unit cell yielded a total of 48354 reflections to a maximum θ angle of 29.16° (0.73 Å resolution), of which 5026 were independent (average redundancy 9.62, completeness = 99.7%, Rint = 4.85%, Rsig = 2.95%) and 4626 (92.0%) were greater than $2\sigma(F^2)$. The final cell constants of a = 10.6686(5) Å, b = 12.9319(7) Å, c = 13.5490(7) Å, volume = 1869.29(17) $Å^3$, are based upon the refinement of the XYZ-centroids of 9992 reflections above 20 $\sigma(I)$ with $2.18° < 2\theta < 28.91°$. Data were corrected for absorption effects using the Multi-Scan method in SADABS 2016/2. The calculated minimum and maximum transmission coefficients (based on crystal size) are 0.546 and 0.744.[2] Table 1 contains additional crystal and refinement information.

Structure solution and refinement

The polar space group $P2_12_12_1$ was determined based on intensity statistics and systematic absences. The structure was solved and refined using the SHELX suite of programs.[3,4] An intrinsic-methods solution was calculated, which provided most non-hydrogen atoms from the E-map. Full-matrix least squares / difference Fourier cycles were performed, which located the remaining non-hydrogen atoms. All non-hydrogen atoms were refined with anisotropic displacement parameters. The hydrogen atoms were placed in ideal positions and refined as riding atoms with relative isotropic displacement parameters. The final anisotropic full-matrix least-squares refinement on F^2 with 220 variables against 5026 data points converged at R_1 = 2.80%, for the observed data and wR_2 = 7.21% for all data. The goodnessof-fit on F^2 was 1.06. The largest peak in the final difference electron density synthesis was 0.71 $e^-/Å^3$ and the deepest hole was −1.07 $e^-/Å^3$ with an RMS deviation of 0.056 $e^-/Å^3$. On the basis of the final model, the calculated density was 1.38 g/cm^3 and $F(000)$, 800 e^-.

1 SAINT V8.41 (2024), Bruker AXS, Madison, WI.
2 L. Krause, R. Herbst-Irmer, G. M. Sheldrick, D. Stalke:
Comparison of silver and molybdenum microfocus X-ray sources for single-crystal structure determination.
J. Appl. Cryst., 48, 3-10 (2015). doi:10.1107/S1600576714022985. 3 G. M. Sheldrick:
SHELXT--Integrated space-group and crystal-structure determination.
Acta Cryst. A71, 3-8 (2015). doi:10.1107/S2053273314026370.
4 G. M. Sheldrick:
Crystal structure refinement with SHELXL.
Acta Cryst. C71, 3-8 (2015). doi:10.1107/S2053229614024218.

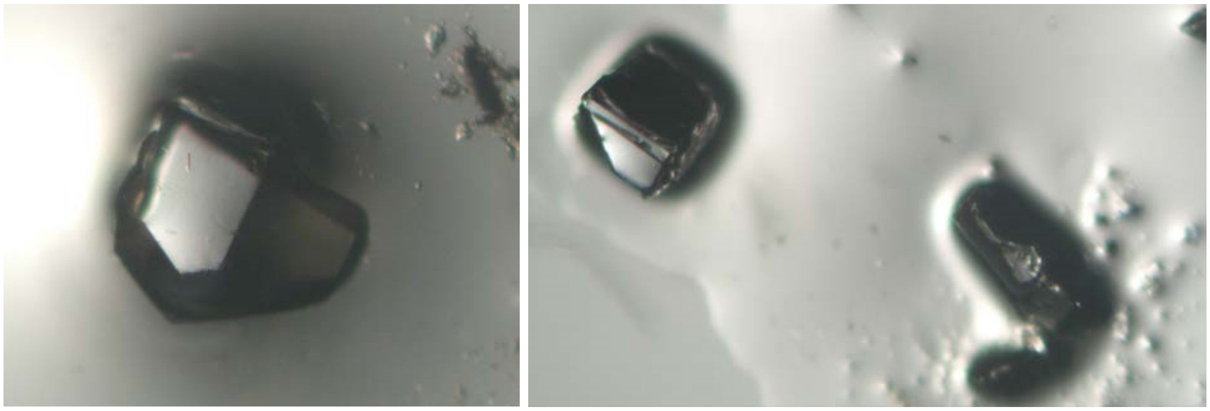

Bulk material.

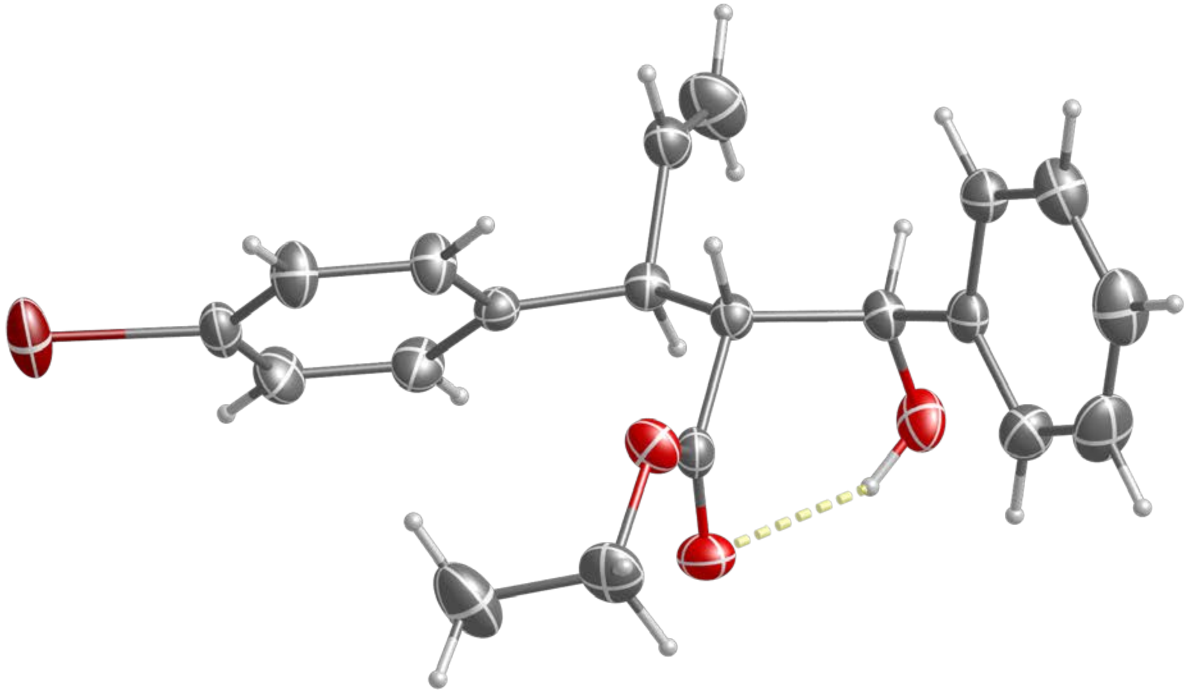

Formula unit.

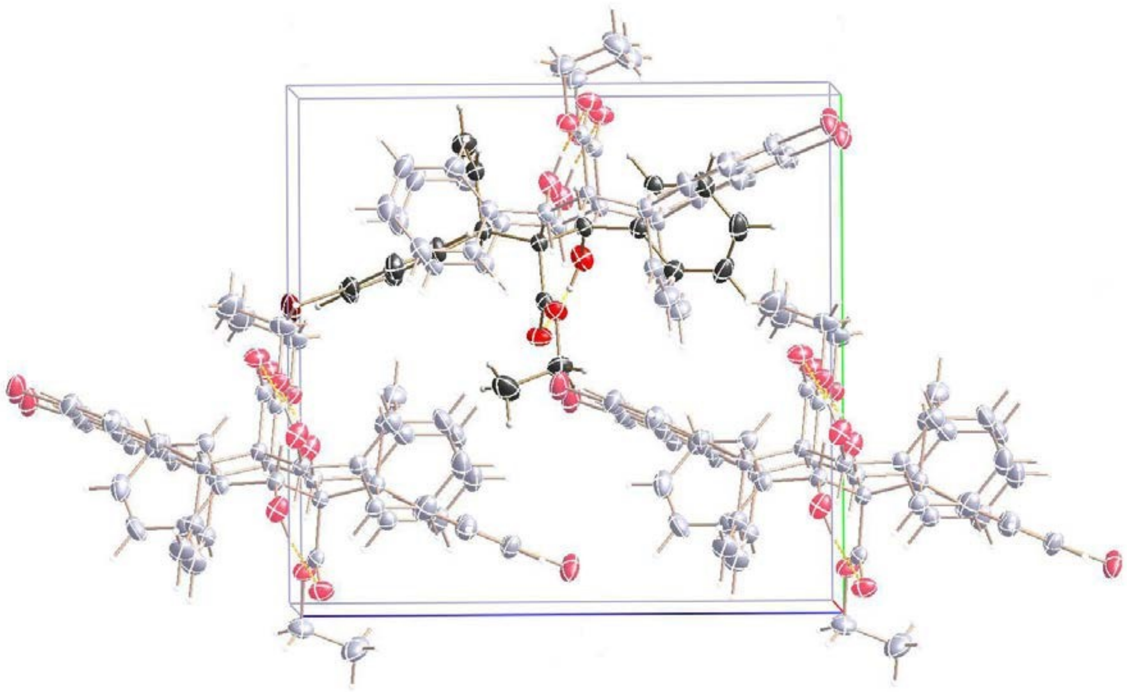

Cell plot, view along a.

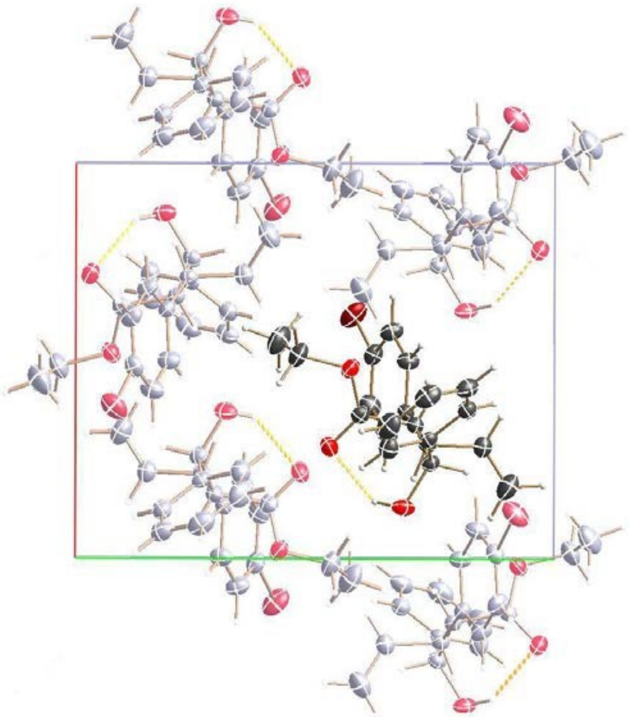

Cell plot, view along b.

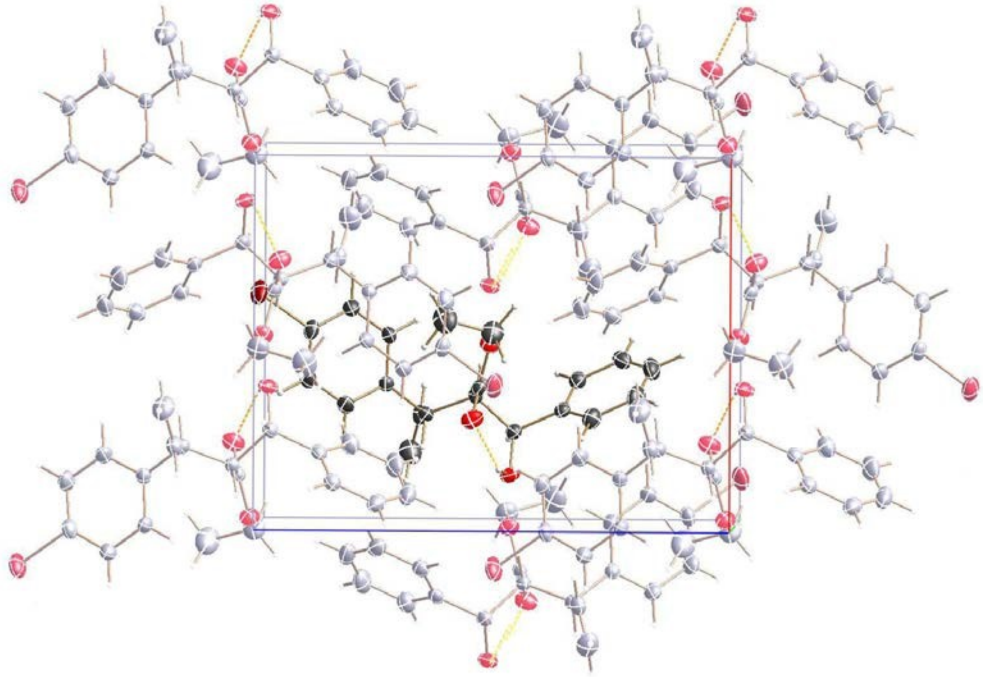

Cell plot, view along c.

Table 1. Crystal data and structure refinement for 25003.

Empirical formula	C20 H21 Br O3	
Formula weight	389.28	
Crystal color, shape, size	colorless block, 0.311 × 0.310 × 0.142 mm^3	
Temperature	173(2) K	
Wavelength	0.71073 Å	
Crystal system, space group	Orthorhombic, $P2_12_12_1$	
Unit cell dimensions	a = 10.6686(5) Å	α = 90°.
	b = 12.9319(7) Å	β = 90°.
	c = 13.5490(7) Å	γ = 90°.
Volume	1869.29(17) $Å^3$	
Z	4	
Density (calculated)	1.383 Mg/m^3	
Absorption coefficient	2.213 mm^{-1}	
F(000)	800	
Data collection		
Diffractometer	Venture D8, Bruker	
Source	I_{μ}3.0, Incoatec	
Detector	Photon III	
Theta range for data collection	2.177 to 29.155°.	
Index ranges	-12<=h<=14, -17<=k<=17, -18<=l<=18	
Reflections collected	48354	
Independent reflections	5026 [R_{int} = 0.0485]	
Observed Reflections	4626	
Completeness to theta = 25.242°	99.7 %	
Solution and Refinement		
Absorption correction	Semi-empirical from equivalents	
Max. and min. transmission	0.7458 and 0.5956	
Solution	Intrinsic methods	
Refinement method	Full-matrix least-squares on F^2	
Weighting scheme	$w = [\sigma^2Fo^2 + AP^2 + BP]^{-1}$, with $P = (Fo^2 + 2 Fc^2)/3$, A = 0.0323, B = 0.5308	
Data / restraints / parameters	5026 / 0 / 220	
Goodness-of-fit on F^2	1.058	
Final R indices [$I>2\sigma(I)$]	R1 = 0.0280, wR2 = 0.0698	
R indices (all data)	R1 = 0.0322, wR2 = 0.0721	
Absolute structure parameter	0.024(3), from anomalous dispersion	

Largest diff. peak and hole 0.708 and -1.074 e.Å^{-3}

Goodness-of-fit = $[\Sigma[w(F_o^2 - F_c^2)^2]/N_{observns} - N_{params})]^{1/2}$, all data.
R1 = $\Sigma(|F_o| - |F_c|) / \Sigma |F_o|$. $wR2 = [\Sigma[w(F_o^2 - F_c^2)^2] / \Sigma [w(F_o^2)^2]]^{1/2}$.

Table 2. Atomic coordinates (× 10^4) and equivalent isotropic displacement parameters (Å^2 × 10^3) for 25003. U_{eq} is defined as one third of the trace of the orthogonalized U^{ij} tensor.

	x	y	z	U(eq)
Br1	6143(1)	5803(1)	10033(1)	44(1)
O1	2815(2)	5304(1)	5527(2)	34(1)
O2	4806(2)	5742(1)	5173(1)	30(1)
O3	1277(1)	6849(2)	4763(1)	33(1)
C1	1802(3)	9037(2)	6909(2)	45(1)
C2	2820(2)	8512(2)	6691(2)	28(1)
C3	2873(2)	7356(2)	6551(2)	22(1)
C4	3368(2)	7098(2)	5506(2)	21(1)
C5	3625(2)	5952(2)	5414(2)	24(1)
C6	5142(3)	4650(2)	5097(3)	40(1)
C7	5498(4)	4246(3)	6085(3)	55(1)
C8	3682(2)	6902(2)	7370(2)	22(1)
C9	3137(2)	6501(2)	8216(2)	30(1)
C10	3854(3)	6160(2)	9007(2)	33(1)
C11	5137(2)	6222(2)	8937(2)	28(1)
C12	5723(2)	6602(2)	8112(2)	32(1)
C13	4987(2)	6949(2)	7327(2)	28(1)
C14	2412(2)	7423(2)	4704(2)	24(1)
C15	3004(2)	7373(2)	3679(2)	24(1)
C16	2807(3)	6551(2)	3045(2)	34(1)
C17	3364(3)	6546(3)	2111(2)	43(1)
C18	4111(3)	7361(3)	1814(2)	42(1)
C19	4324(2)	8177(2)	2449(2)	35(1)
C20	3771(2)	8183(2)	3382(2)	27(1)

Table 3. Bond lengths [Å] and angles [°] for 25003.

Br1-C11	1.910(2)	O1-C5	1.214(3)
O2-C5	1.330(3)	O2-C6	1.460(3)
O3-C14	1.422(3)	O3-H3O	0.8400
C1-C2	1.315(4)	C1-H1A	0.9500
C1-H1B	0.9500	C2-C3	1.508(4)
C2-H2	0.9500	C3-C8	1.523(3)
C3-C4	1.547(3)	C3-H3	1.0000
C4-C5	1.512(3)	C4-C14	1.548(3)
C4-H4	1.0000	C6-C7	1.486(5)
C6-H6A	0.9900	C6-H6B	0.9900
C7-H7A	0.9800	C7-H7B	0.9800
C7-H7C	0.9800	C8-C9	1.386(3)
C8-C13	1.395(3)	C9-C10	1.388(4)
C9-H9	0.9500	C10-C11	1.375(4)
C10-H10	0.9500	C11-C12	1.371(4)
C12-C13	1.396(3)	C12-H12	0.9500
C13-H13	0.9500	C14-C15	1.527(3)
C14-H14	1.0000	C15-C16	1.383(4)
C15-C20	1.390(4)	C16-C17	1.397(4)
C16-H16	0.9500	C17-C18	1.382(5)
C17-H17	0.9500	C18-C19	1.381(4)
C18-H18	0.9500	C19-C20	1.394(3)
C19-H19	0.9500	C20-H20	0.9500

C5-O2-C6	116.6(2)	C14-O3-H3O	109.5
C2-C1-H1A	120.0	C2-C1-H1B	120.0
H1A-C1-H1B	120.0	C1-C2-C3	124.9(3)
C1-C2-H2	117.6	C3-C2-H2	117.6
C2-C3-C8	108.14(19)	C2-C3-C4	109.95(19)
C8-C3-C4	112.99(18)	C2-C3-H3	108.6

C8-C3-H3	108.6	C4-C3-H3	108.6
C5-C4-C3	110.35(18)	C5-C4-C14	109.13(17)
C3-C4-C14	111.07(18)	C5-C4-H4	108.8
C3-C4-H4	108.8	C14-C4-H4	108.8
O1-C5-O2	124.3(2)	O1-C5-C4	122.5(2)
O2-C5-C4	113.15(18)	O2-C6-C7	109.8(3)
O2-C6-H6A	109.7	C7-C6-H6A	109.7
O2-C6-H6B	109.7	C7-C6-H6B	109.7
H6A-C6-H6B	108.2	C6-C7-H7A	109.5
C6-C7-H7B	109.5	H7A-C7-H7B	109.5
C6-C7-H7C	109.5	H7A-C7-H7C	109.5
H7B-C7-H7C	109.5	C9-C8-C13	118.0(2)
C9-C8-C3	120.6(2)	C13-C8-C3	121.3(2)
C8-C9-C10	121.7(2)	C8-C9-H9	119.1
C10-C9-H9	119.1	C11-C10-C9	118.5(2)
C11-C10-H10	120.8	C9-C10-H10	120.8
C12-C11-C10	122.1(2)	C12-C11-Br1	118.60(19)
C10-C11-Br1	119.30(19)	C11-C12-C13	118.7(2)
C11-C12-H12	120.7	C13-C12-H12	120.7
C8-C13-C12	121.1(2)	C8-C13-H13	119.5
C12-C13-H13	119.5	O3-C14-C15	112.39(19)
O3-C14-C4	112.31(18)	C15-C14-C4	110.78(18)
O3-C14-H14	107.0	C15-C14-H14	107.0
C4-C14-H14	107.0	C16-C15-C20	119.2(2)
C16-C15-C14	122.4(2)	C20-C15-C14	118.4(2)
C15-C16-C17	120.1(3)	C15-C16-H16	119.9
C17-C16-H16	119.9	C18-C17-C16	120.4(3)
C18-C17-H17	119.8	C16-C17-H17	119.8
C19-C18-C17	119.7(3)	C19-C18-H18	120.1
C17-C18-H18	120.1	C18-C19-C20	120.0(3)

C18-C19-H19	120.0	C20-C19-H19	120.0
C15-C20-C19	120.5(2)	C15-C20-H20	119.7
C19-C20-H20	119.7		

Table 4. Anisotropic displacement parameters (Å^2 × 10^3) for 25003. The anisotropic displacement factor exponent takes the form: $-2\pi^2[h^2 a^{*2}U^{11} + ... + 2 h k a^* b^* U^{12}]$

	U11	U22	U33	U23	U13	U12
Br1	52(1)	55(1)	26(1)	6(1)	-6(1)	22(1)
O1	32(1)	26(1)	45(1)	2(1)	-7(1)	-5(1)
O2	29(1)	27(1)	35(1)	2(1)	2(1)	7(1)
O3	20(1)	45(1)	35(1)	9(1)	-4(1)	-2(1)
C1	54(2)	38(2)	44(2)	-1(1)	6(1)	15(1)
C2	35(1)	28(1)	23(1)	2(1)	-2(1)	2(1)
C3	20(1)	26(1)	22(1)	2(1)	1(1)	-1(1)
C4	21(1)	23(1)	20(1)	2(1)	0(1)	-1(1)
C5	24(1)	27(1)	21(1)	3(1)	-5(1)	2(1)
C6	45(1)	30(1)	44(2)	-4(1)	1(1)	13(1)
C7	67(2)	44(2)	53(2)	14(2)	3(2)	20(2)
C8	22(1)	22(1)	21(1)	0(1)	0(1)	-3(1)
C9	27(1)	36(1)	27(1)	5(1)	2(1)	-9(1)
C10	38(1)	36(1)	25(1)	9(1)	2(1)	-6(1)
C11	35(1)	28(1)	21(1)	2(1)	-4(1)	6(1)
C12	25(1)	44(1)	26(1)	4(1)	0(1)	6(1)
C13	24(1)	40(1)	21(1)	6(1)	3(1)	-1(1)
C14	22(1)	26(1)	23(1)	3(1)	-1(1)	2(1)
C15	21(1)	28(1)	22(1)	1(1)	-4(1)	5(1)
C16	39(1)	32(1)	31(1)	-2(1)	-4(1)	1(1)

C17	54(2)	45(2)	29(1)	-15(1)	-3(1)	10(1)
C18	43(2)	55(2)	26(1)	0(1)	5(1)	14(1)
C19	29(1)	43(2)	32(1)	8(1)	4(1)	6(1)
C20	27(1)	29(1)	26(1)	1(1)	-2(1)	2(1)

Table 5. Hydrogen coordinates (× 10^4) and isotropic displacement parameters (Å^2 × 10^3) for 25003.

	x	y	z	U_{eq}
H3O	1428	6257	4988	42(8)
H1A	1026	8687	6990	54
H1B	1843	9766	6986	54
H2	3579	8888	6617	34
H3	2005	7071	6617	27
H4	4168	7484	5395	26
H6A	5852	4567	4634	48
H6B	4421	4252	4837	48
H7A	5812	3537	6018	82
H7B	4762	4249	6518	82
H7C	6153	4685	6370	82
H9	2250	6459	8256	36
H10	3466	5890	9583	39
H12	6612	6629	8076	38
H13	5382	7221	6755	34
H14	2195	8164	4831	28
H16	2292	5989	3244	41
H17	3229	5978	1679	51
H18	4476	7360	1174	50
H19	4848	8733	2251	42
H20	3920	8746	3817	33

Table 6. Torsion angles [°] for 25003.

C1-C2-C3-C8	-114.7(3)	C1-C2-C3-C4	121.5(3)
C2-C3-C4-C5	170.80(19)	C8-C3-C4-C5	49.9(2)
C2-C3-C4-C14	-68.0(2)	C8-C3-C4-C14	171.05(18)

C6-O2-C5-O1	-3.5(4)	C6-O2-C5-C4	178.3(2)
C3-C4-C5-O1	59.9(3)	C14-C4-C5-O1	-62.4(3)
C3-C4-C5-O2	-121.9(2)	C14-C4-C5-O2	115.8(2)
C5-O2-C6-C7	-86.0(3)	C2-C3-C8-C9	96.5(3)
C4-C3-C8-C9	-141.6(2)	C2-C3-C8-C13	-78.5(3)
C4-C3-C8-C13	43.4(3)	C13-C8-C9-C10	0.4(4)
C3-C8-C9-C10	-174.7(2)	C8-C9-C10-C11	-0.2(4)
C9-C10-C11-C12	-0.4(4)	C9-C10-C11-Br1	177.8(2)
C10-C11-C12-C13	0.9(4)	Br1-C11-C12-C13	-177.4(2)
C9-C8-C13-C12	0.1(4)	C3-C8-C13-C12	175.2(2)
C11-C12-C13-C8	-0.7(4)	C5-C4-C14-O3	57.0(2)
C3-C4-C14-O3	-64.9(2)	C5-C4-C14-C15	-69.6(2)
C3-C4-C14-C15	168.51(18)	O3-C14-C15-C16	-26.5(3)
C4-C14-C15-C16	100.0(3)	O3-C14-C15-C20	153.9(2)
C4-C14-C15-C20	-79.6(3)	C20-C15-C16-C17	-0.8(4)
C14-C15-C16-C17	179.6(2)	C15-C16-C17-C18	-0.2(5)
C16-C17-C18-C19	1.1(5)	C17-C18-C19-C20	-1.0(4)
C16-C15-C20-C19	0.9(4)	C14-C15-C20-C19	-179.5(2)
C18-C19-C20-C15	0.0(4)		

Table 7. Hydrogen bonds for 25003 [Å and °].

D-H...A	d(D-H)	d(H...A)	d(D...A)	<(DHA)
O3-H3O...O1	0.84	2.06	2.785(3)	144.3
C4-H4...O3#1	1.00	2.42	3.409(3)	170.1
C10-H10...O1#2	0.95	2.43	3.315(3)	155.8

Symmetry transformations used to generate equivalent atoms: #1 x+1/2,-y+3/2,-z+1 #2 -x+1/2,-y+1,z+1/2

13. NMR spectrum:

^{1}H NMR (500 MHz, $CDCl_3$) (E-9)

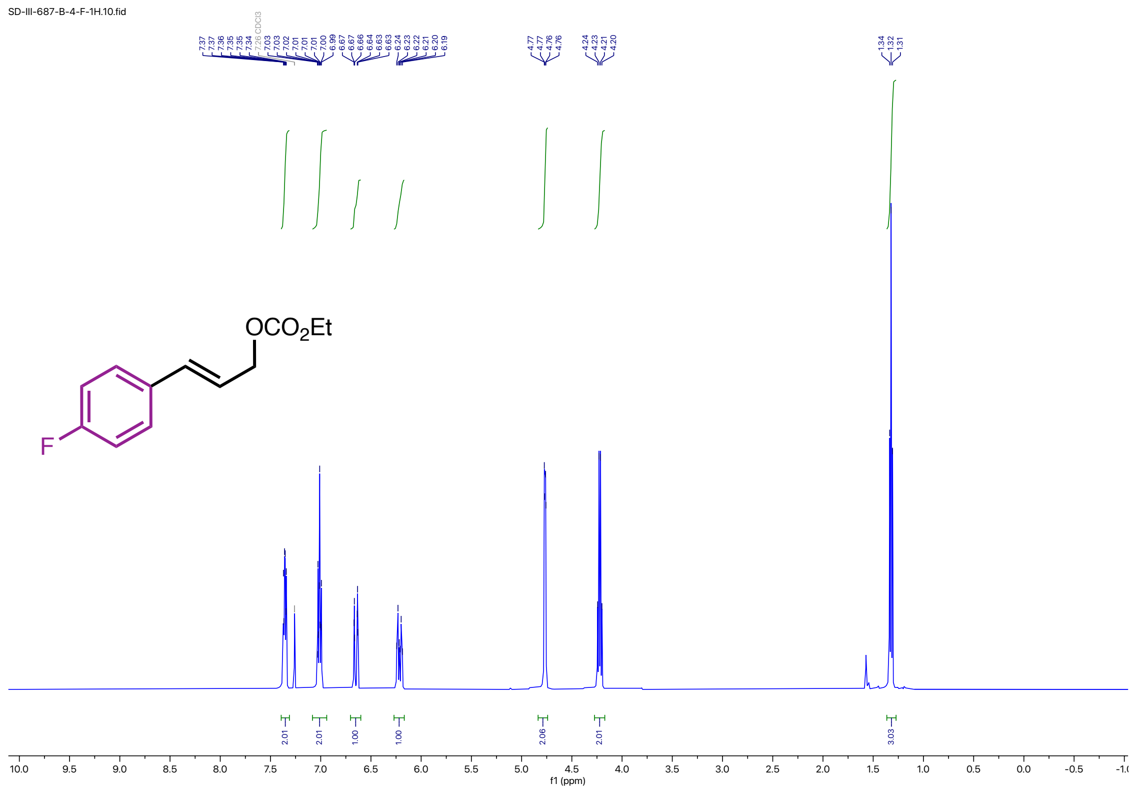

^{13}C NMR (126 MHz, $CDCl_3$) (E-9)

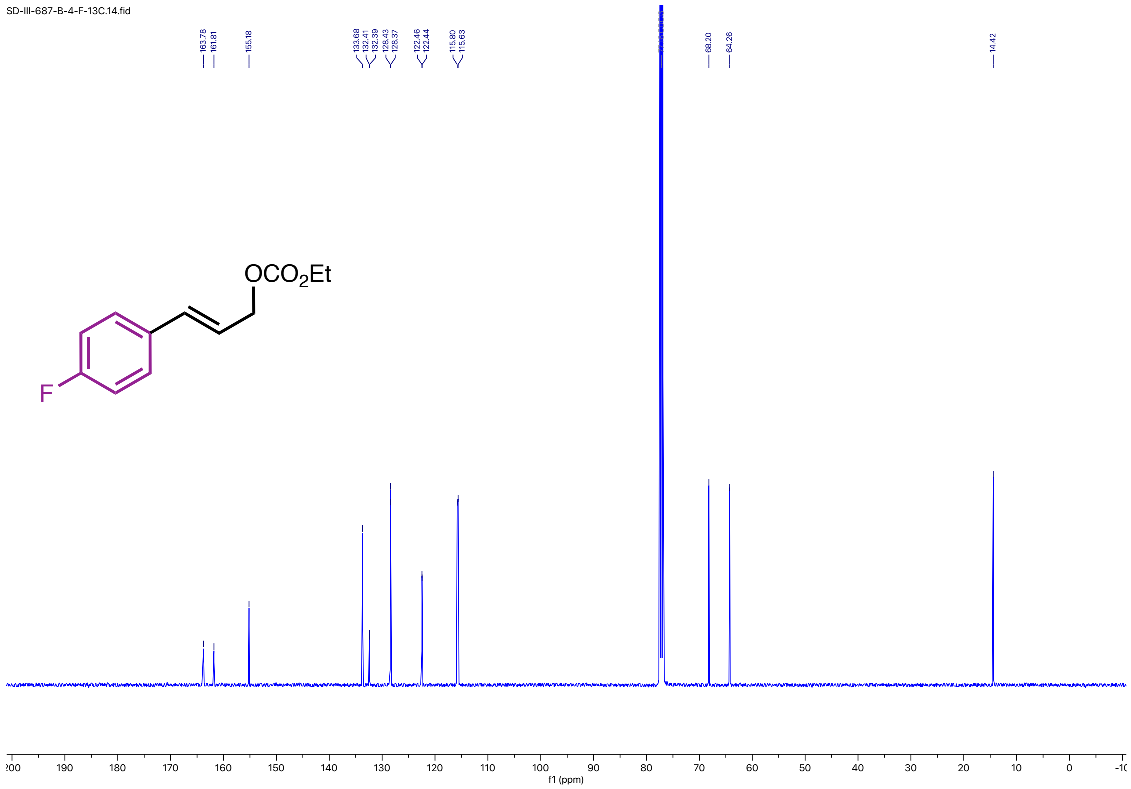

^{19}F NMR (471 MHz, $CDCl_3$) (E-9)

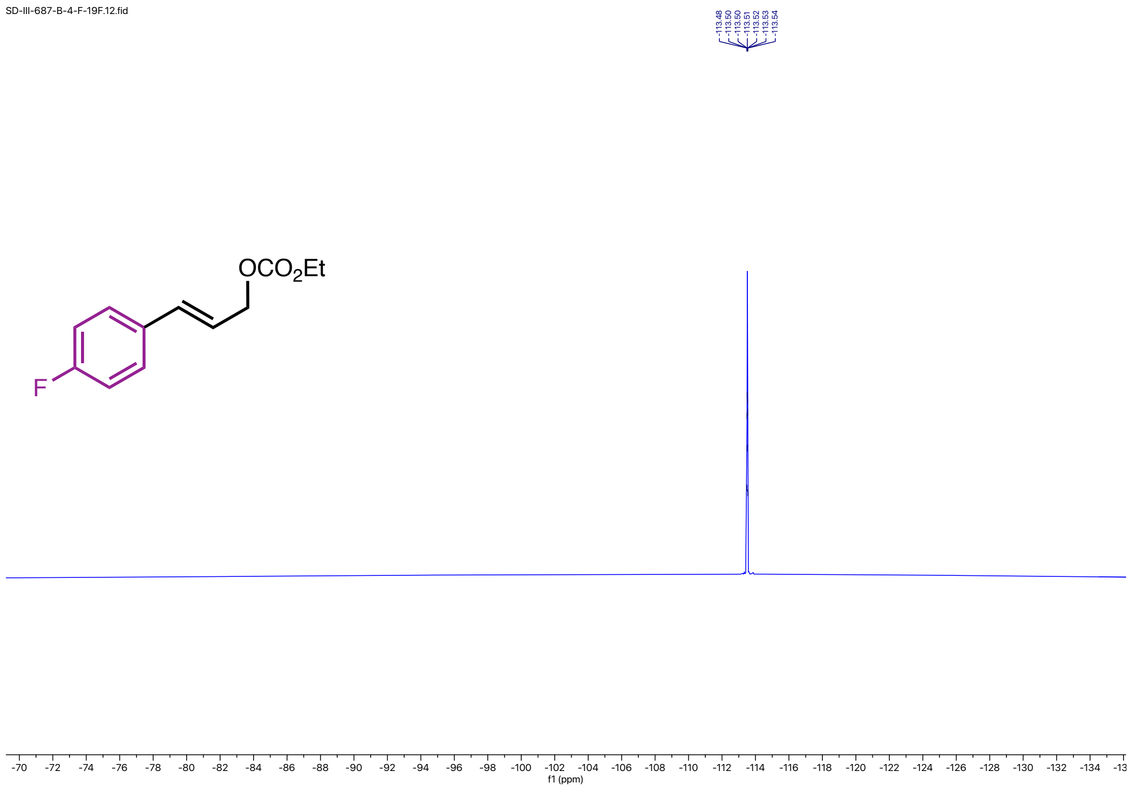

^{1}H NMR (500 MHz, $CDCl_3$) (S-17)

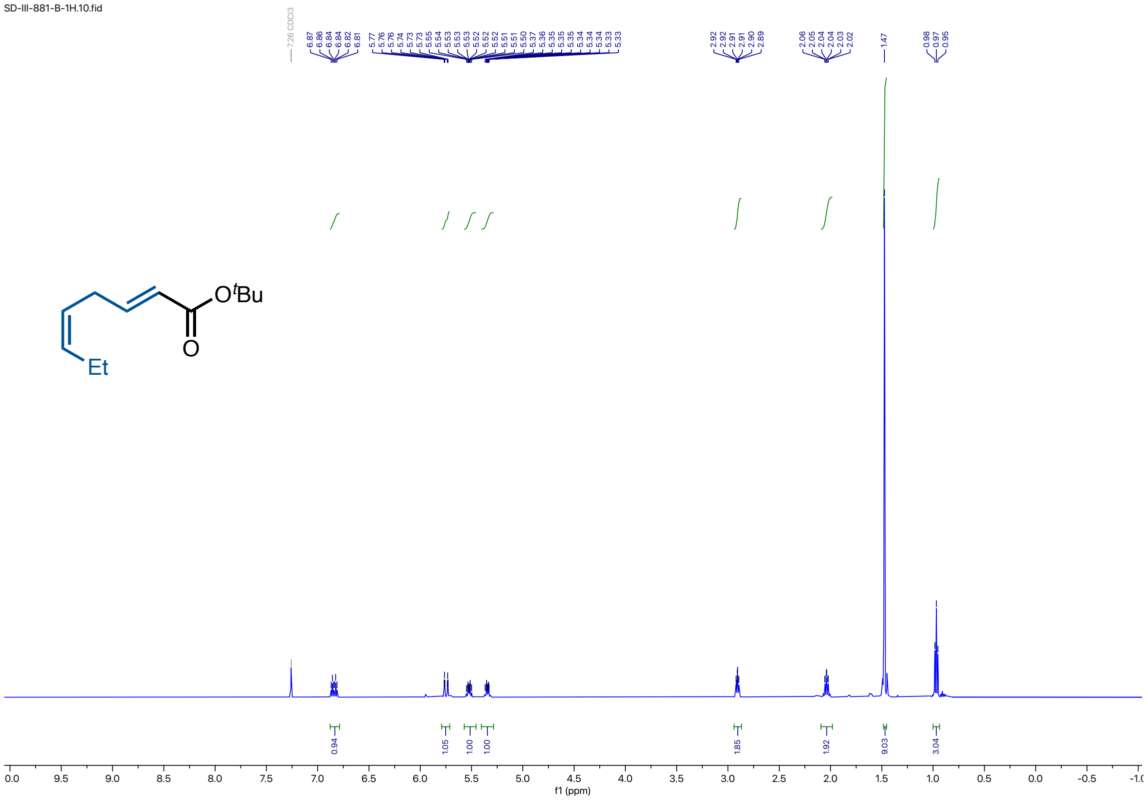

^{13}C NMR (126 MHz, $CDCl_3$) (S-17)

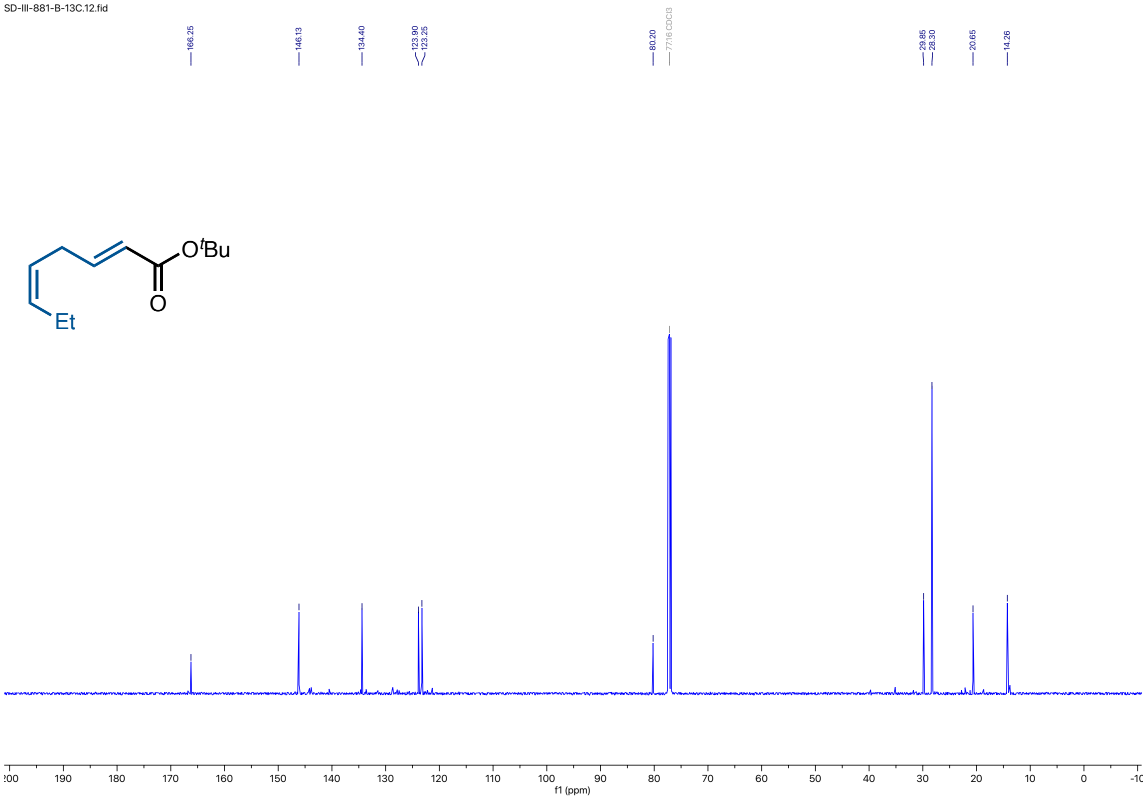

^{1}H NMR (500 MHz, $CDCl_3$) (5)

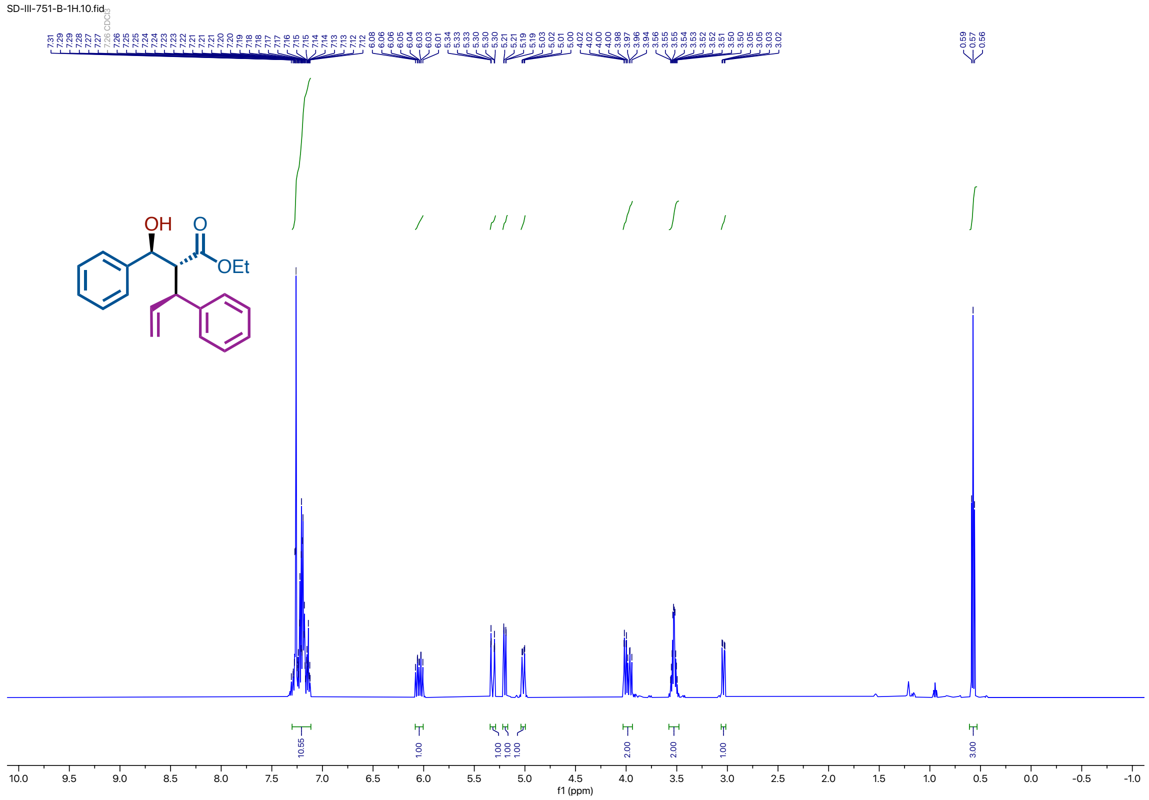

^{13}C NMR (126 MHz, $CDCl_3$) (5)

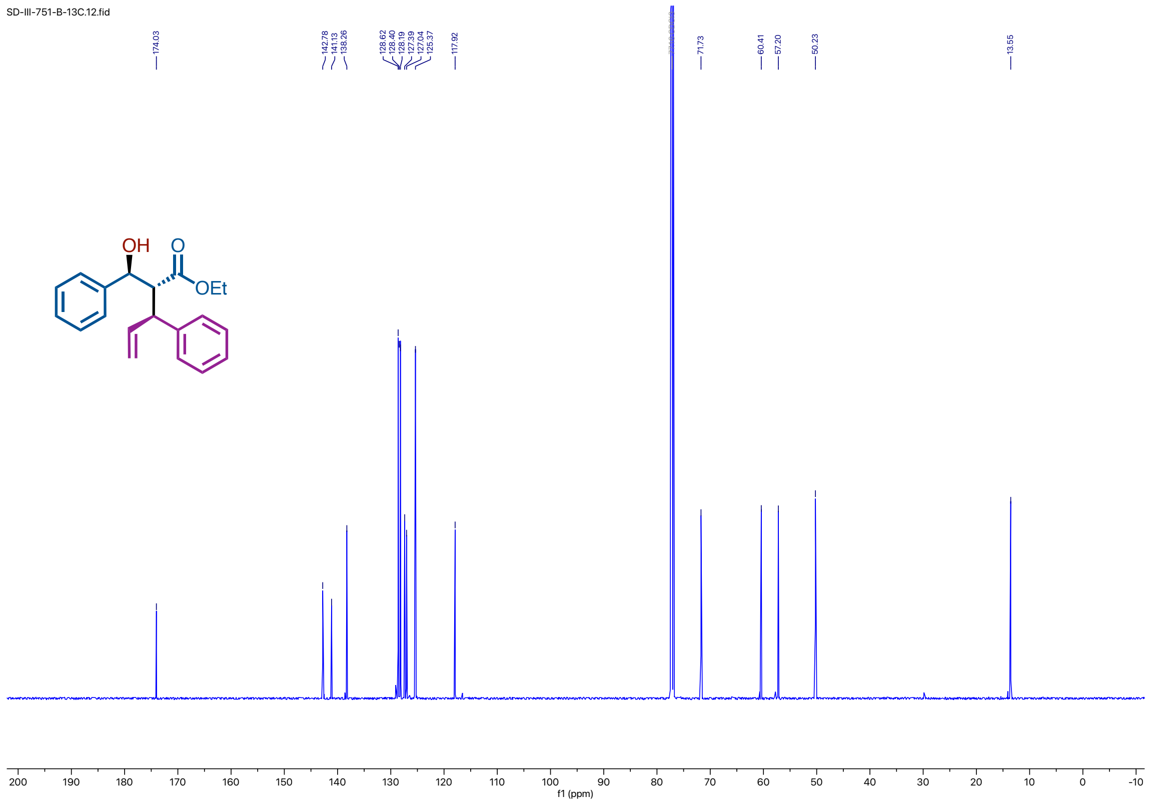

^{1}H NMR (500 MHz, $CDCl_3$) (6)

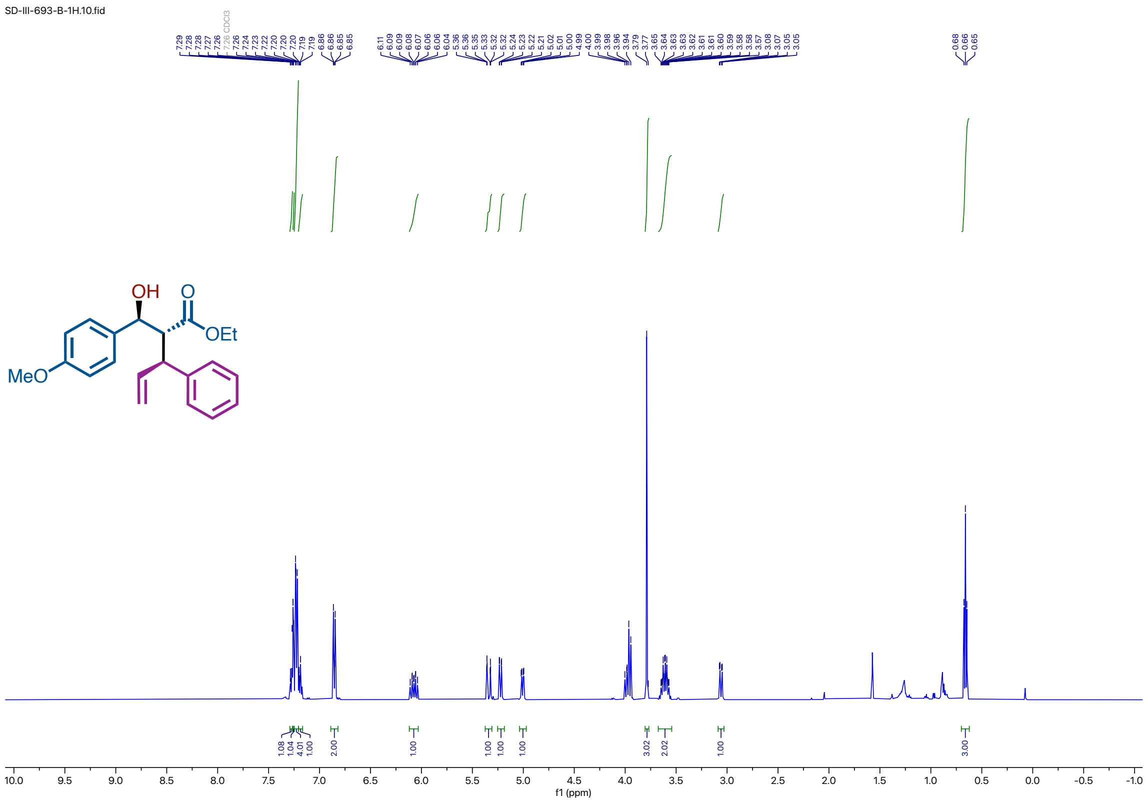

^{13}C NMR (126 MHz, $CDCl_3$) (6)

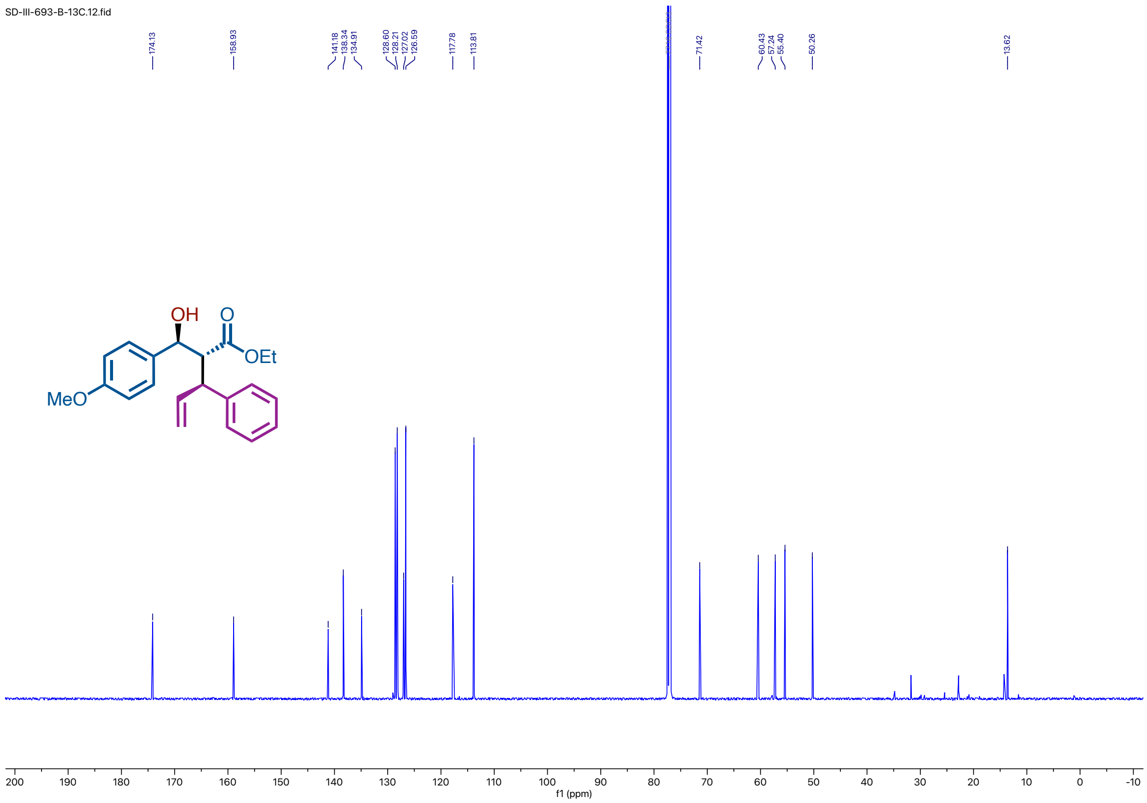

^{1}H NMR (500 MHz, $CDCl_3$) (7)

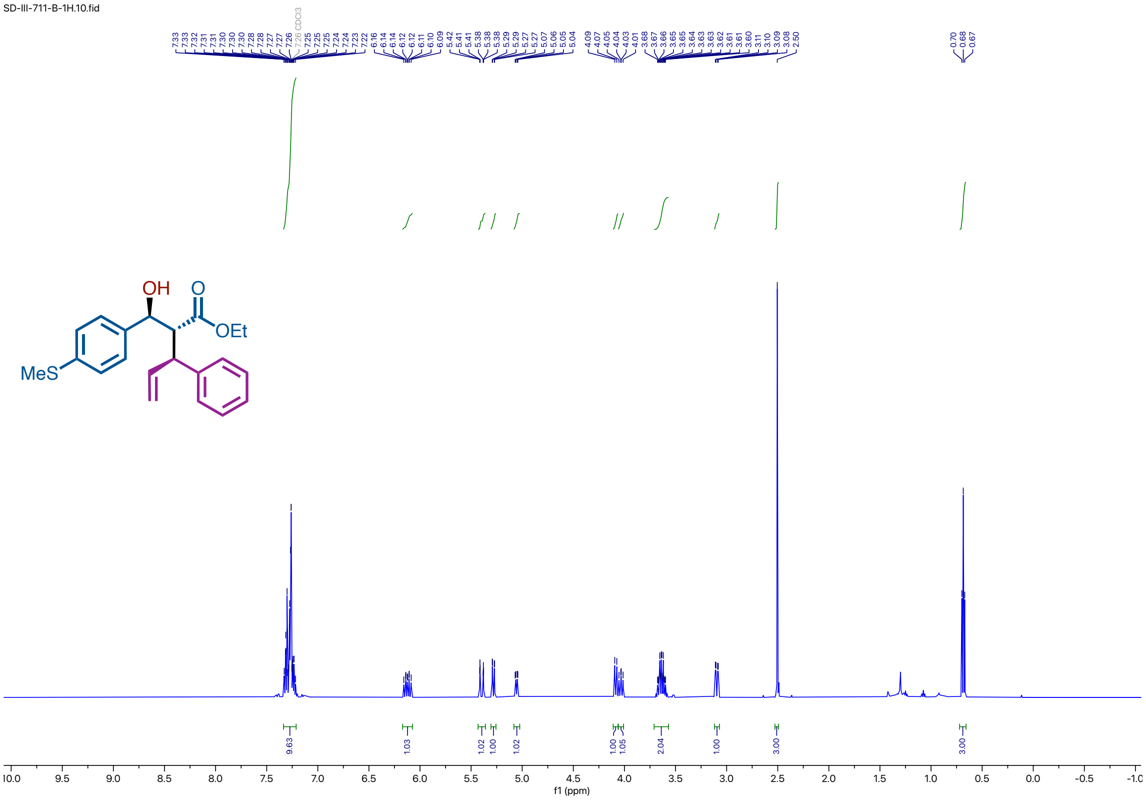

^{13}C NMR (126 MHz, $CDCl_3$) (7)

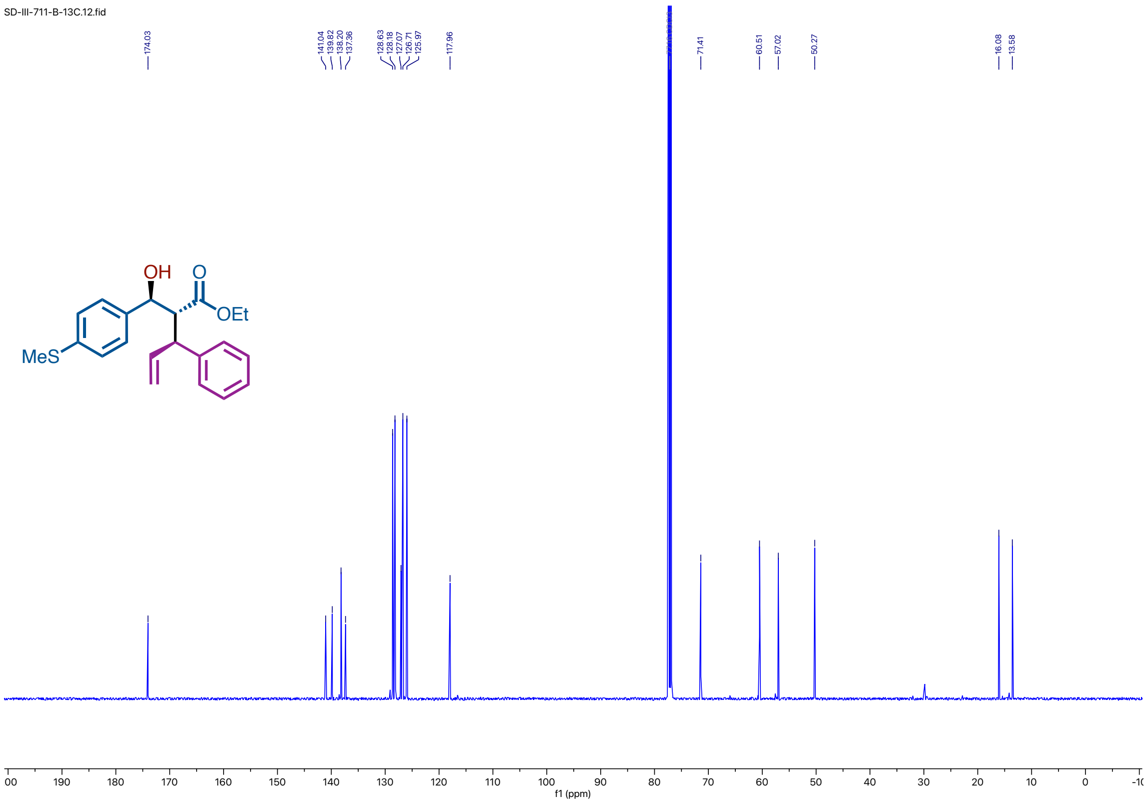

^{1}H NMR (500 MHz, $CDCl_3$) (8)

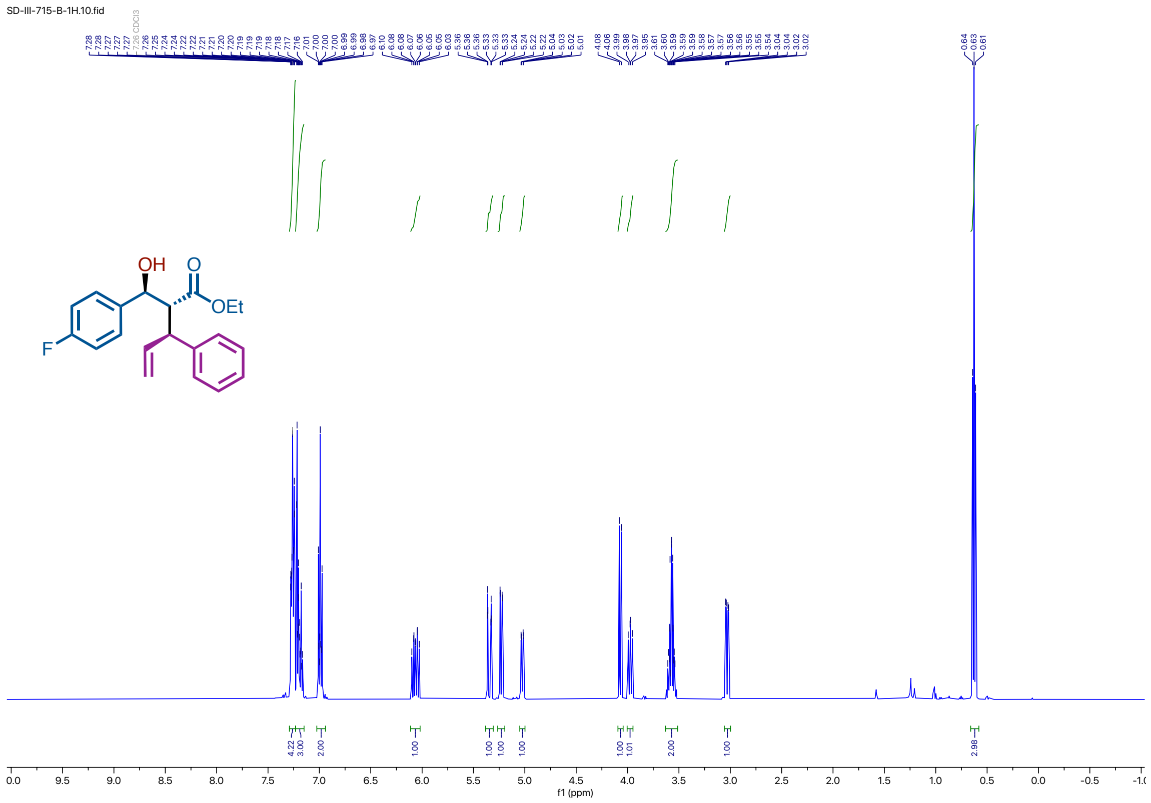

^{13}C NMR (126 MHz, $CDCl_3$) (8)

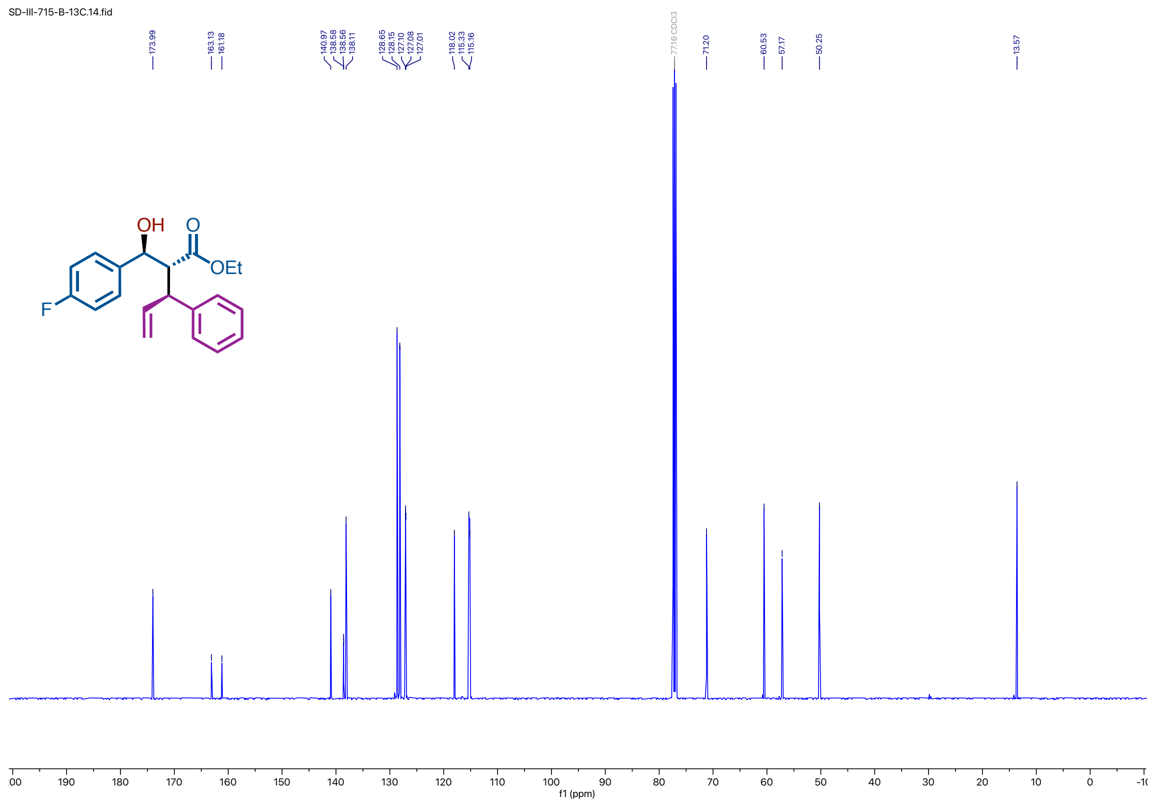

^{19}F NMR (471 MHz, $CDCl_3$) (8)

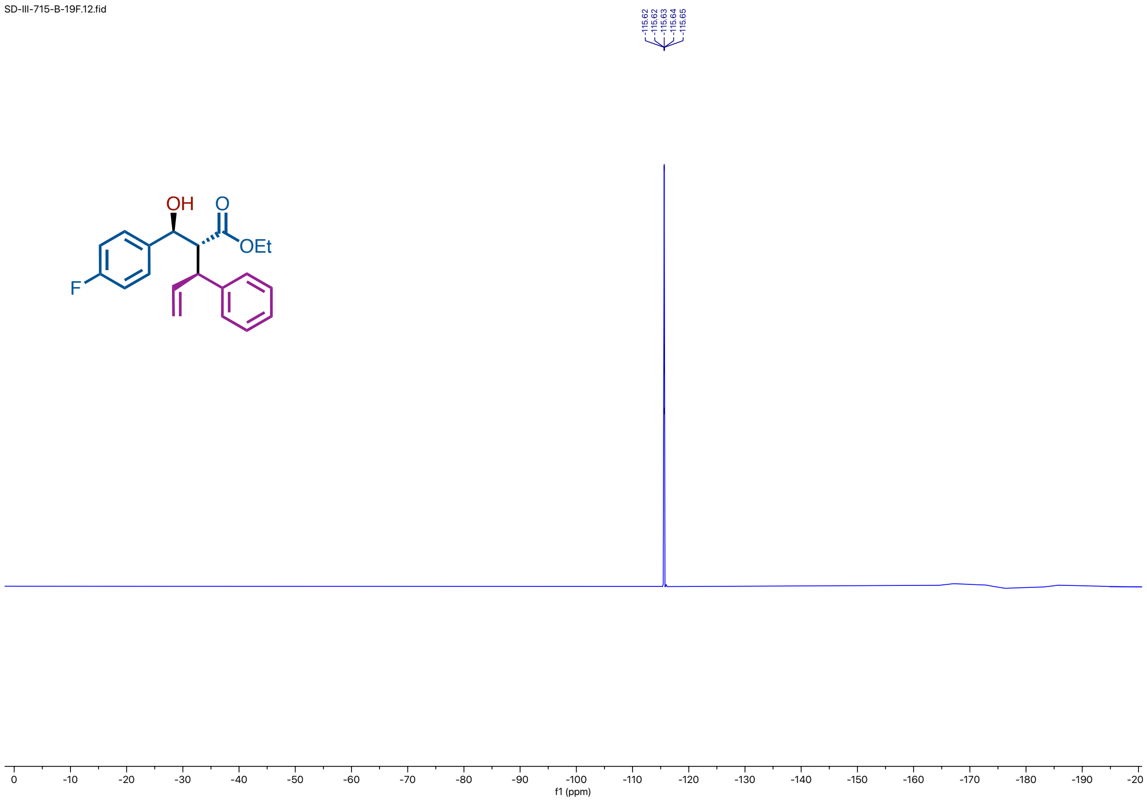

^{1}H NMR (500 MHz, $CDCl_3$) (9)

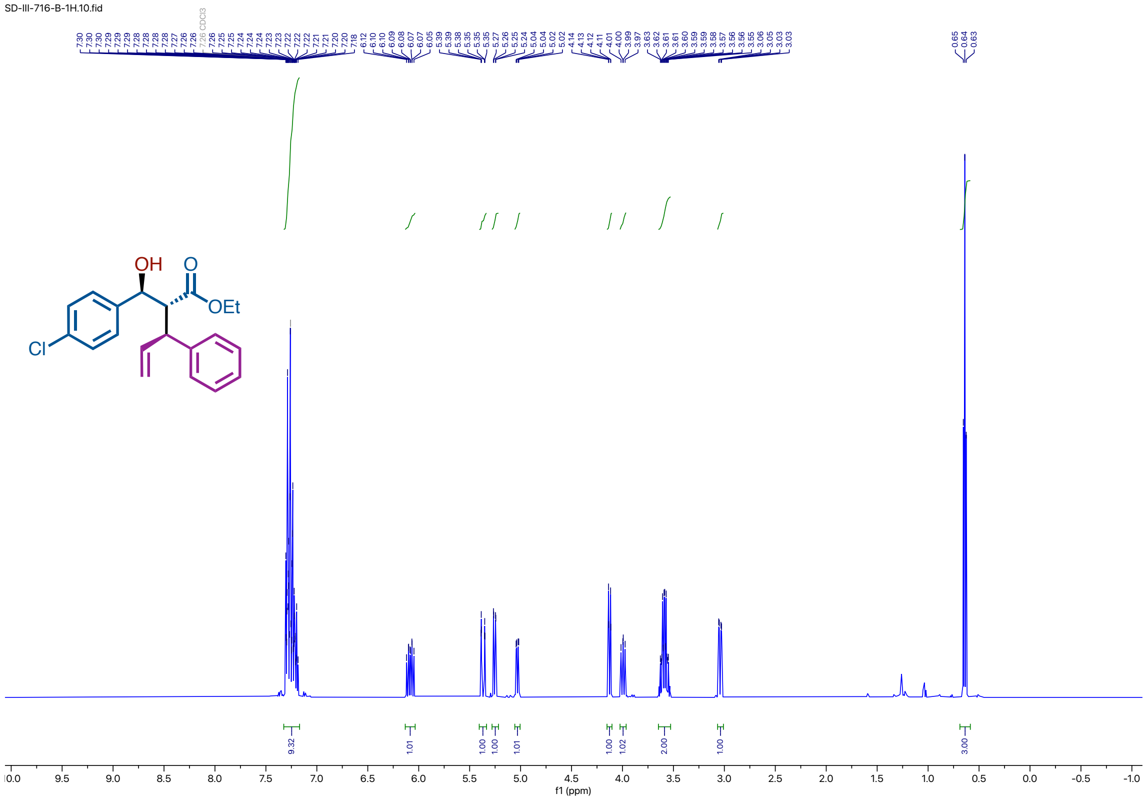

^{13}C NMR (126 MHz, $CDCl_3$) (9)

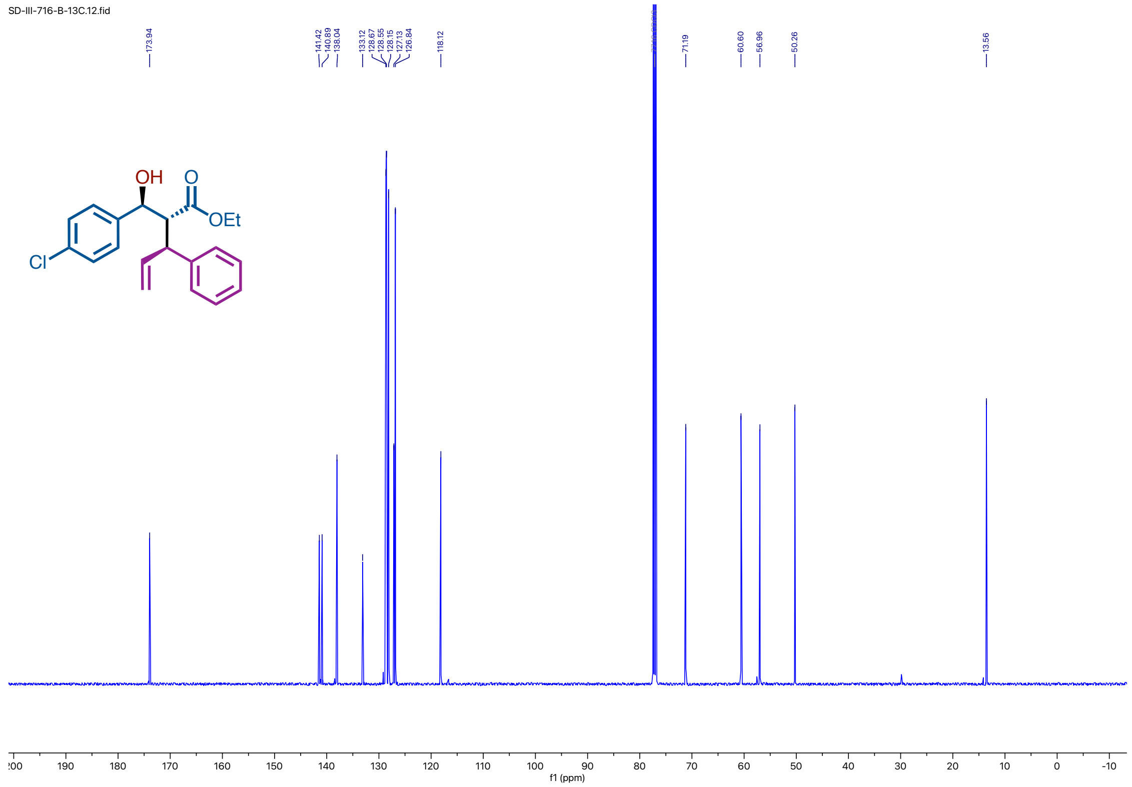

^{1}H NMR (500 MHz, $CDCl_3$) (10)

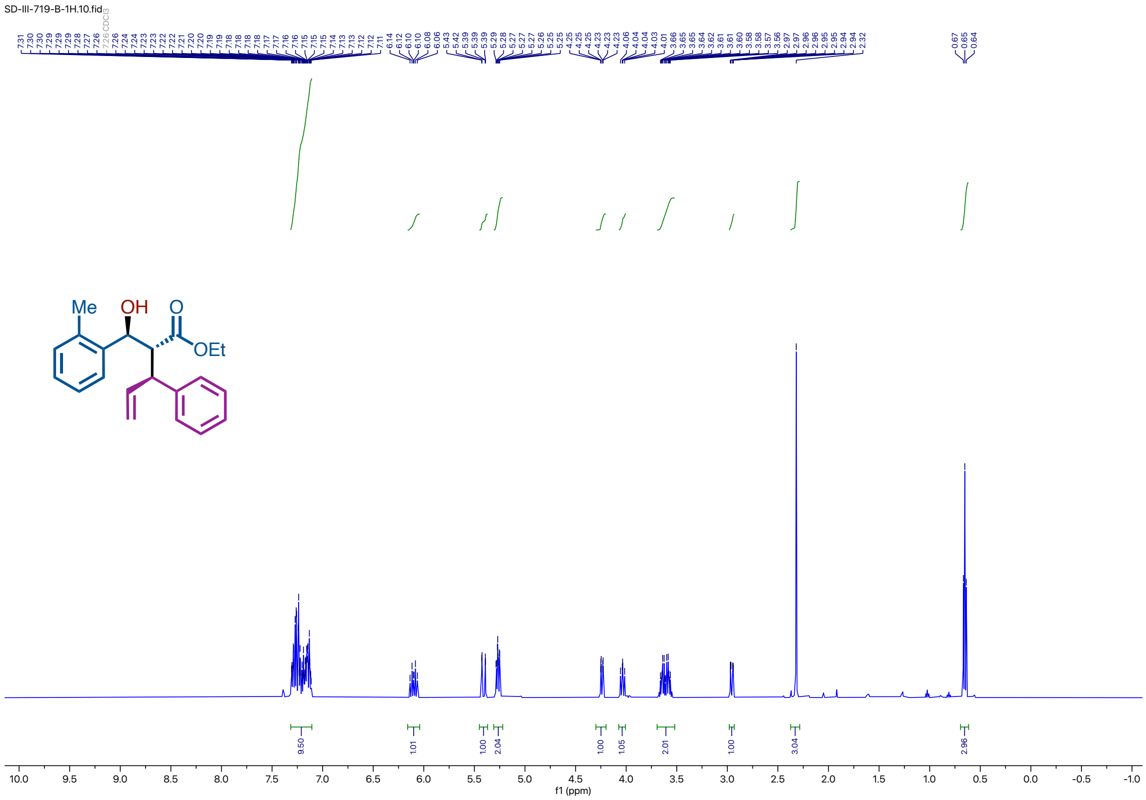

^{13}C NMR (126 MHz, $CDCl_3$) (10)

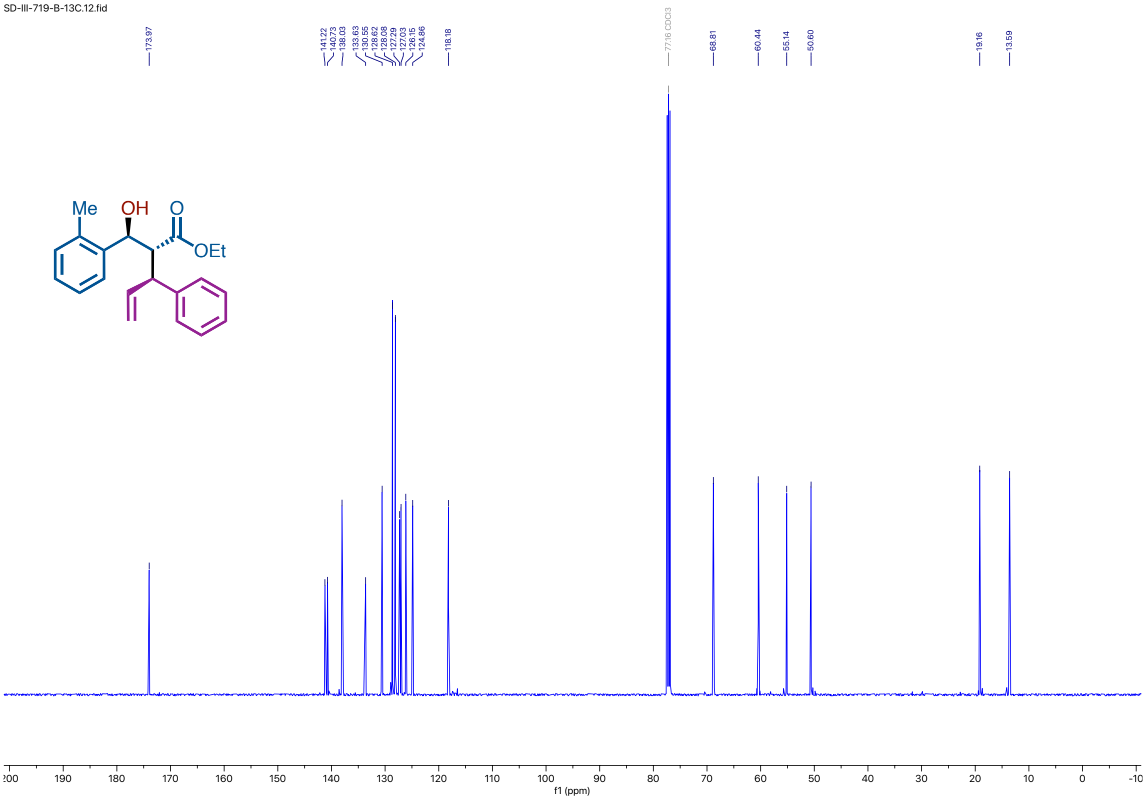

^{1}H NMR (500 MHz, $CDCl_3$) (11)

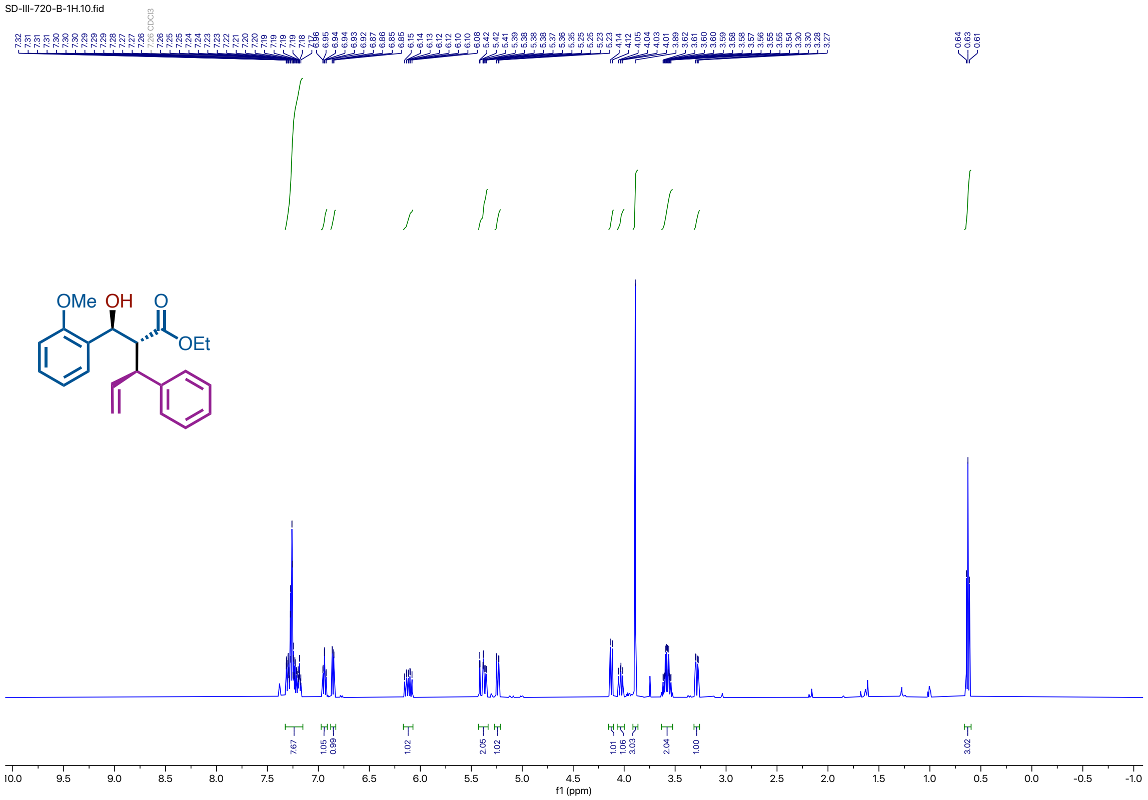

^{13}C NMR (126 MHz, $CDCl_3$) (11)

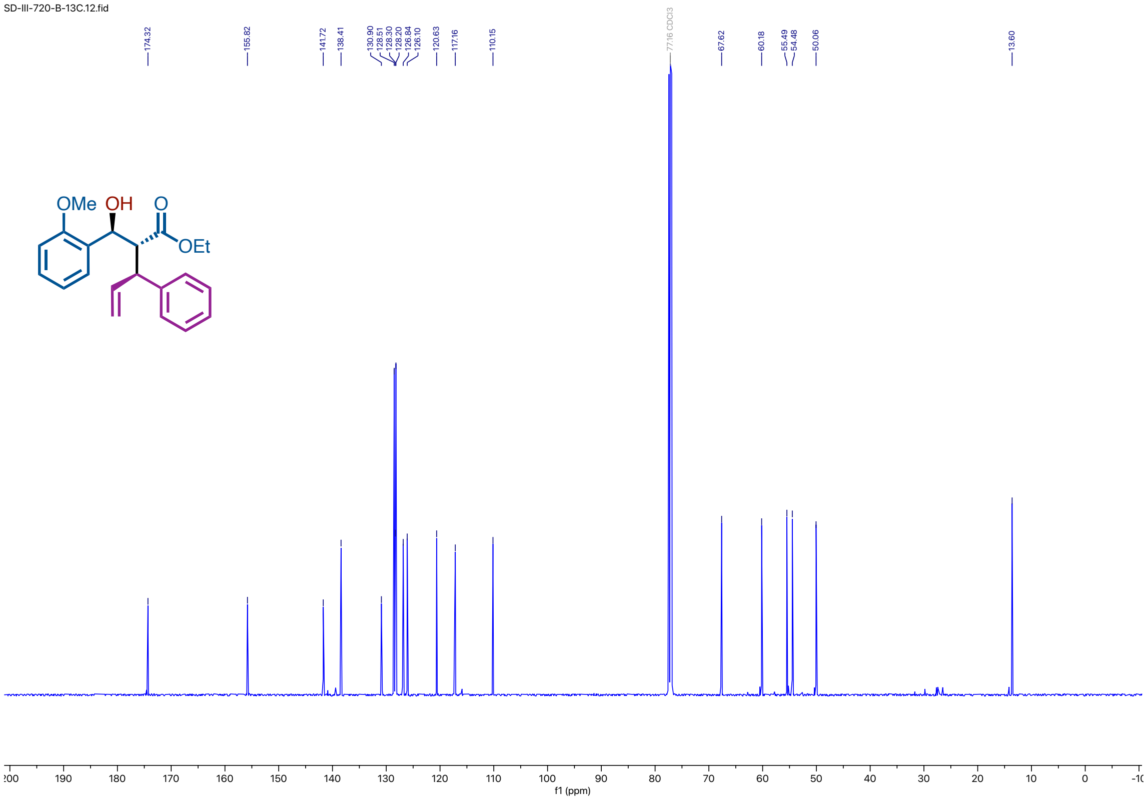

^{1}H NMR (500 MHz, $CDCl_3$) (12)

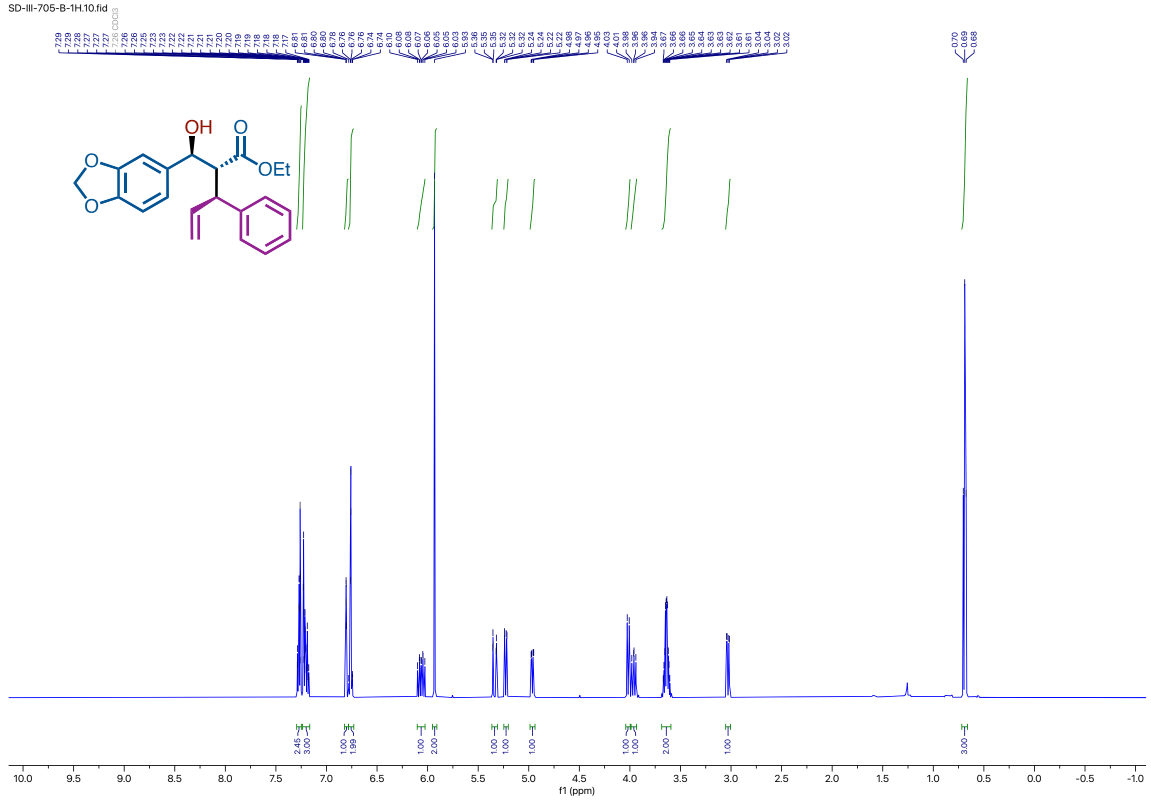

^{13}C NMR (126 MHz, $CDCl_3$) (12)

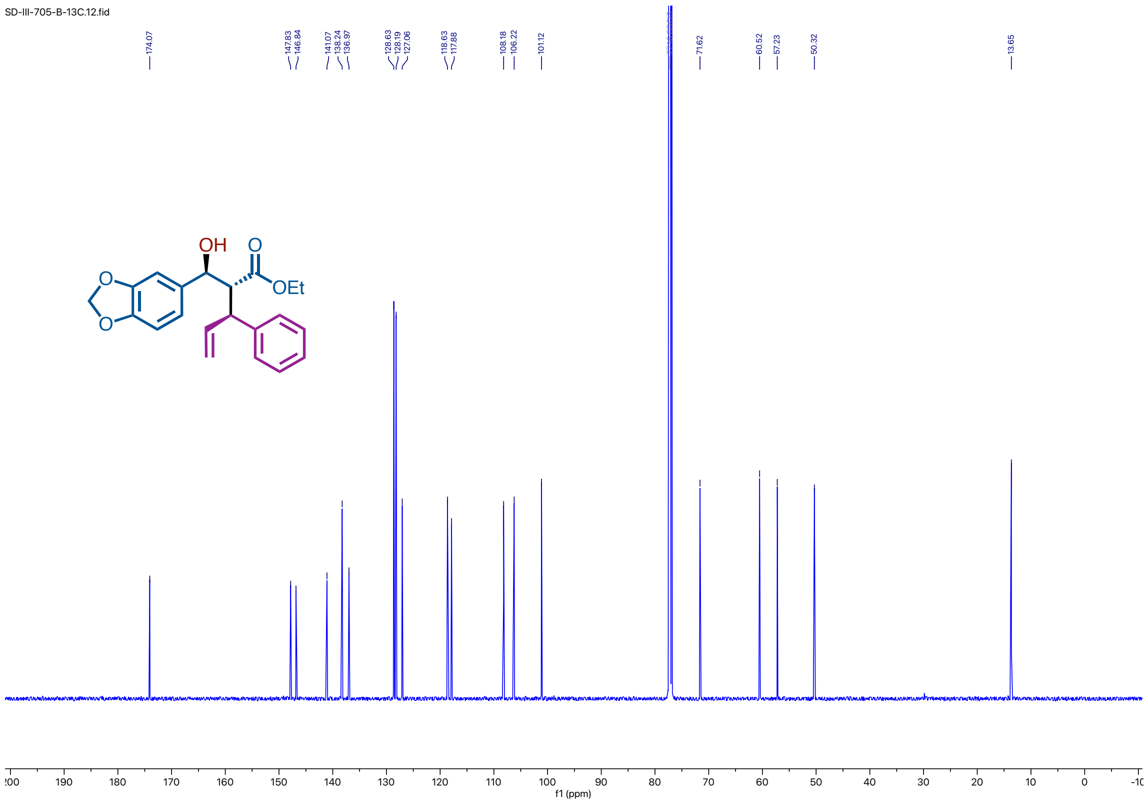

^{1}H NMR (500 MHz, $CDCl_3$) (13)

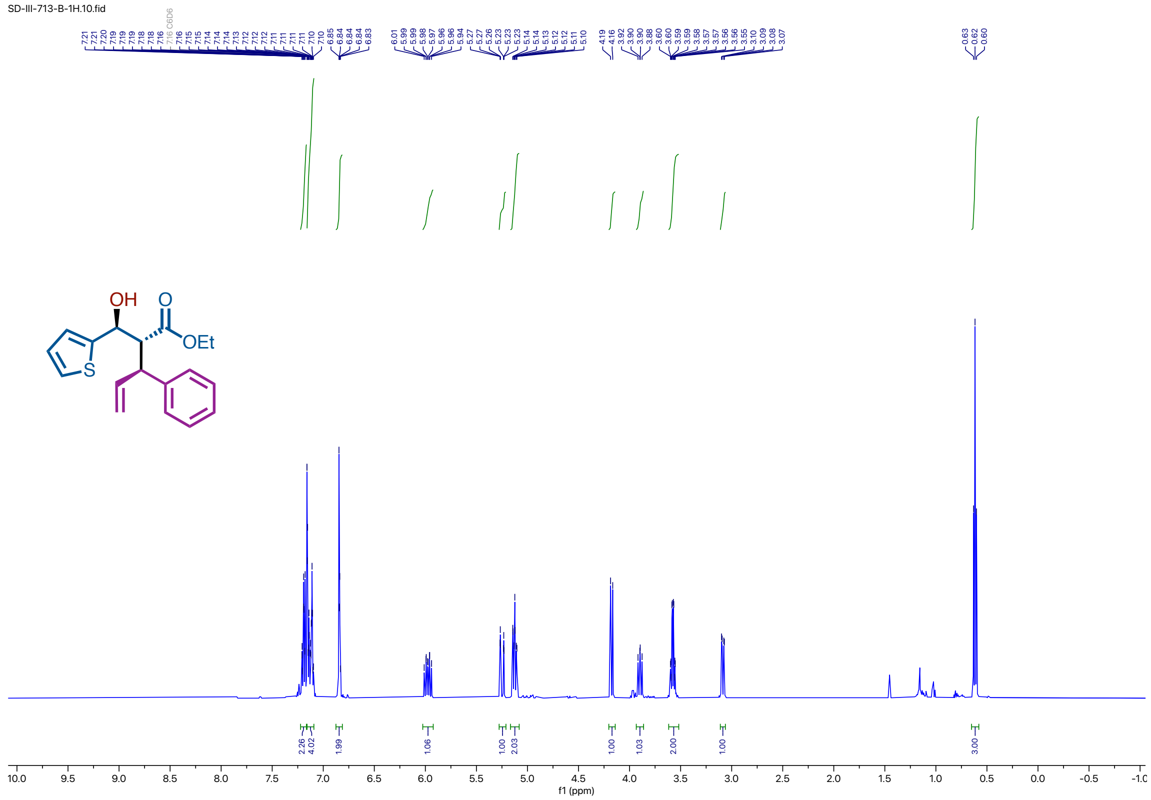

^{13}C NMR (126 MHz, $CDCl_3$) (13)

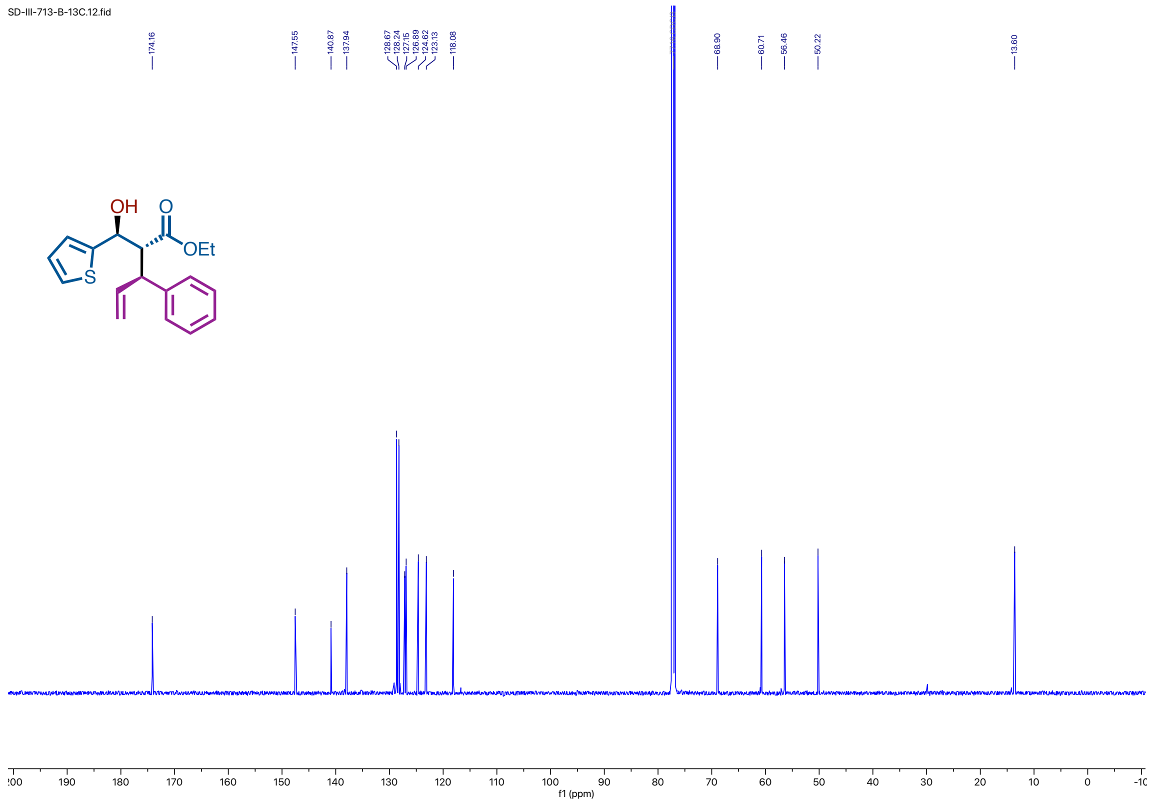

^{1}H NMR (500 MHz, $CDCl_3$) (14)

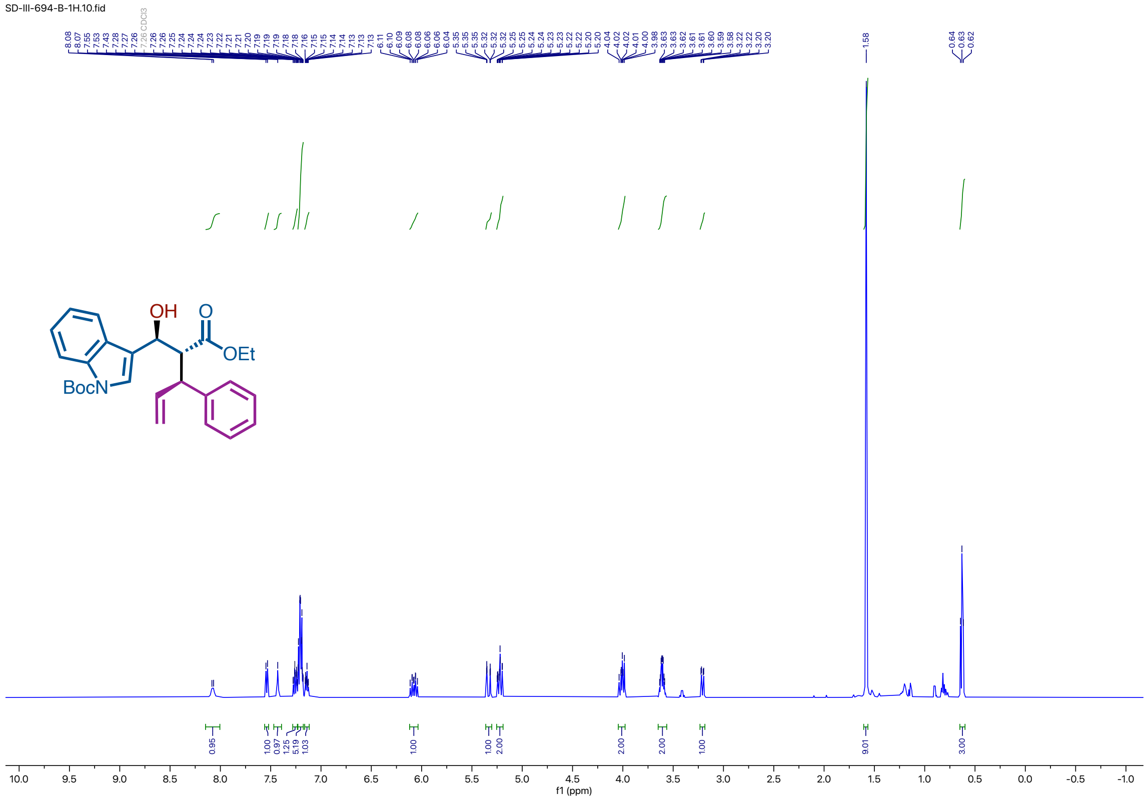

^{13}C NMR (126 MHz, $CDCl_3$) (14)

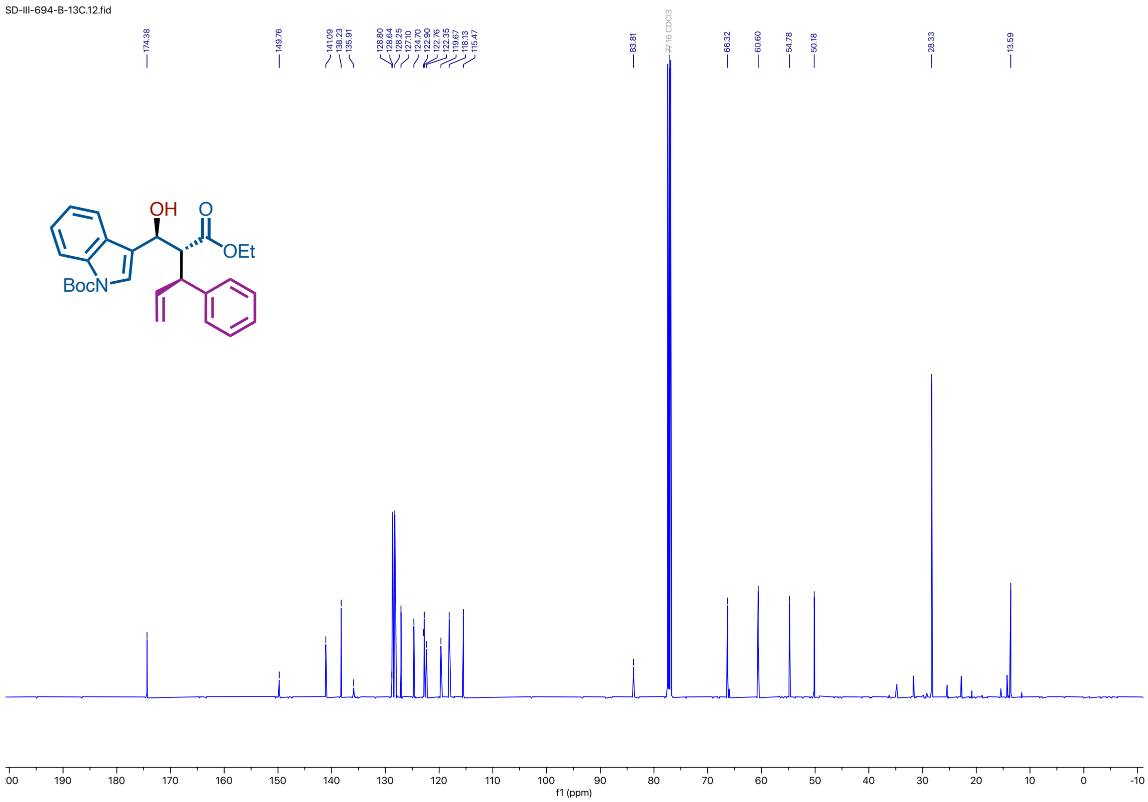

^{1}H NMR (500 MHz, $CDCl_3$) (15)

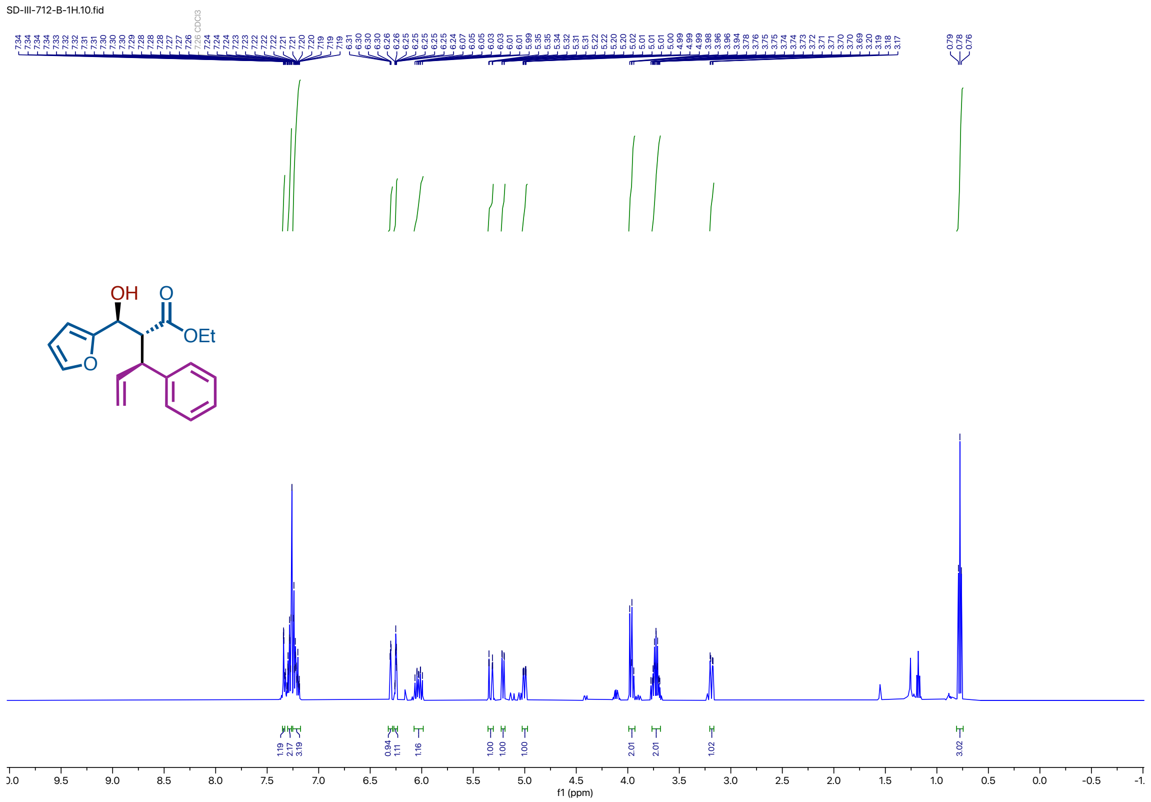

^{13}C NMR (126 MHz, $CDCl_3$) (15)

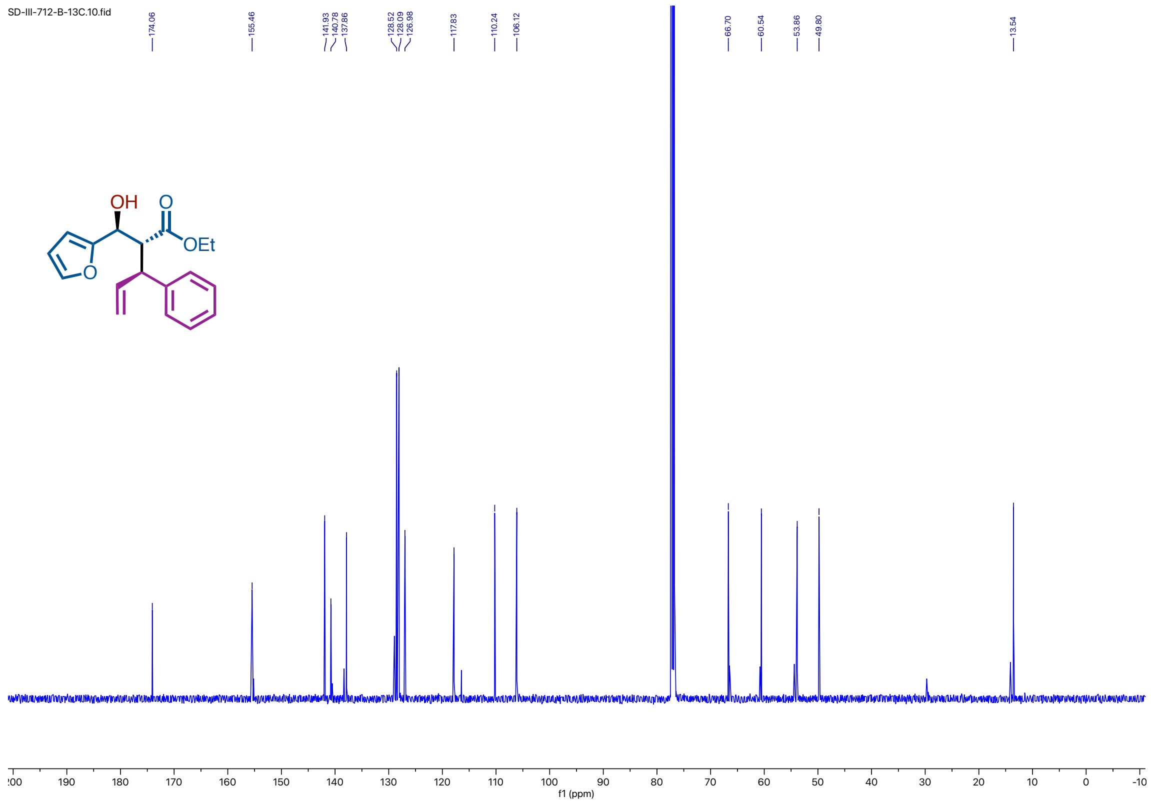

^{1}H NMR (500 MHz, $CDCl_3$) (16)

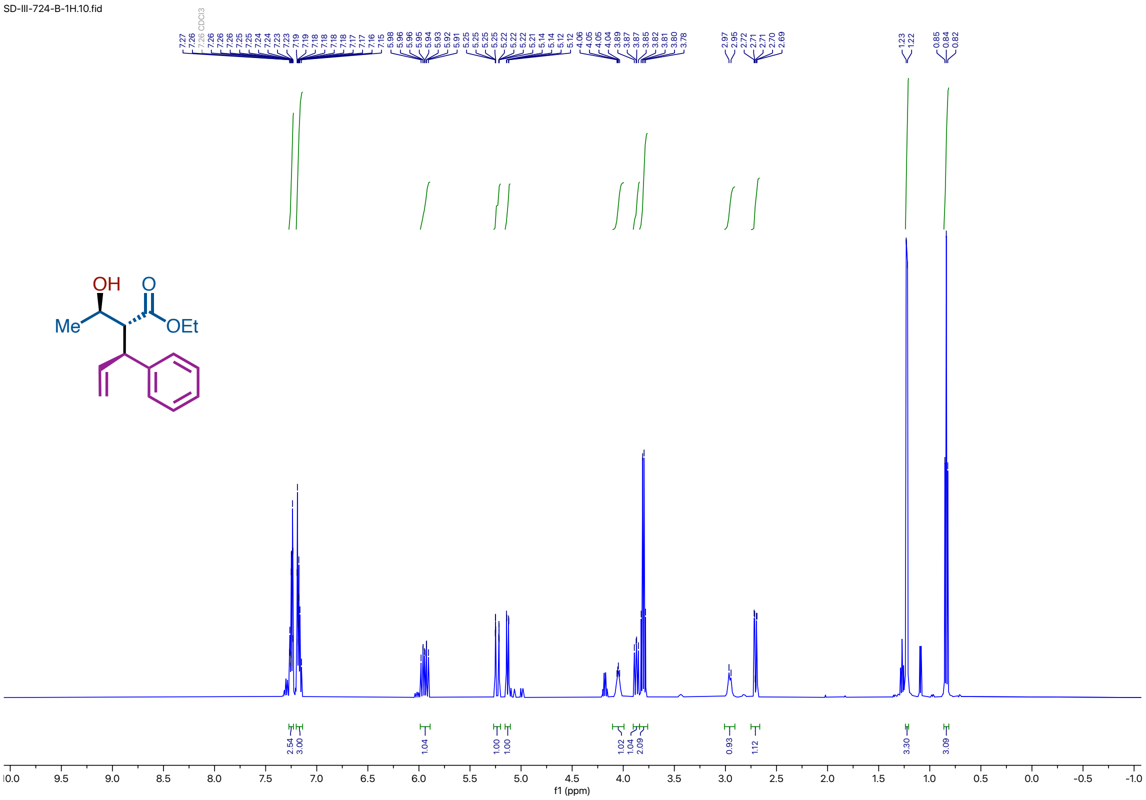

^{13}C NMR (126 MHz, $CDCl_3$) (16)

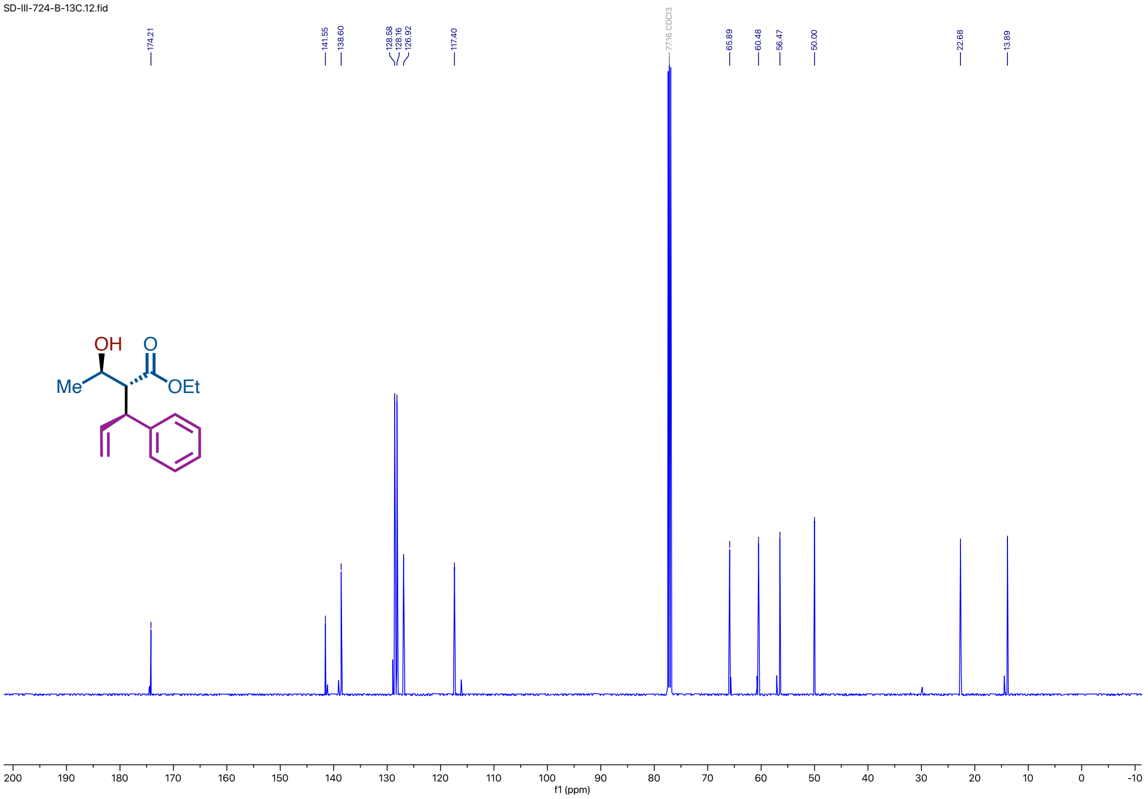

^{1}H NMR (500 MHz, $CDCl_3$) (17)

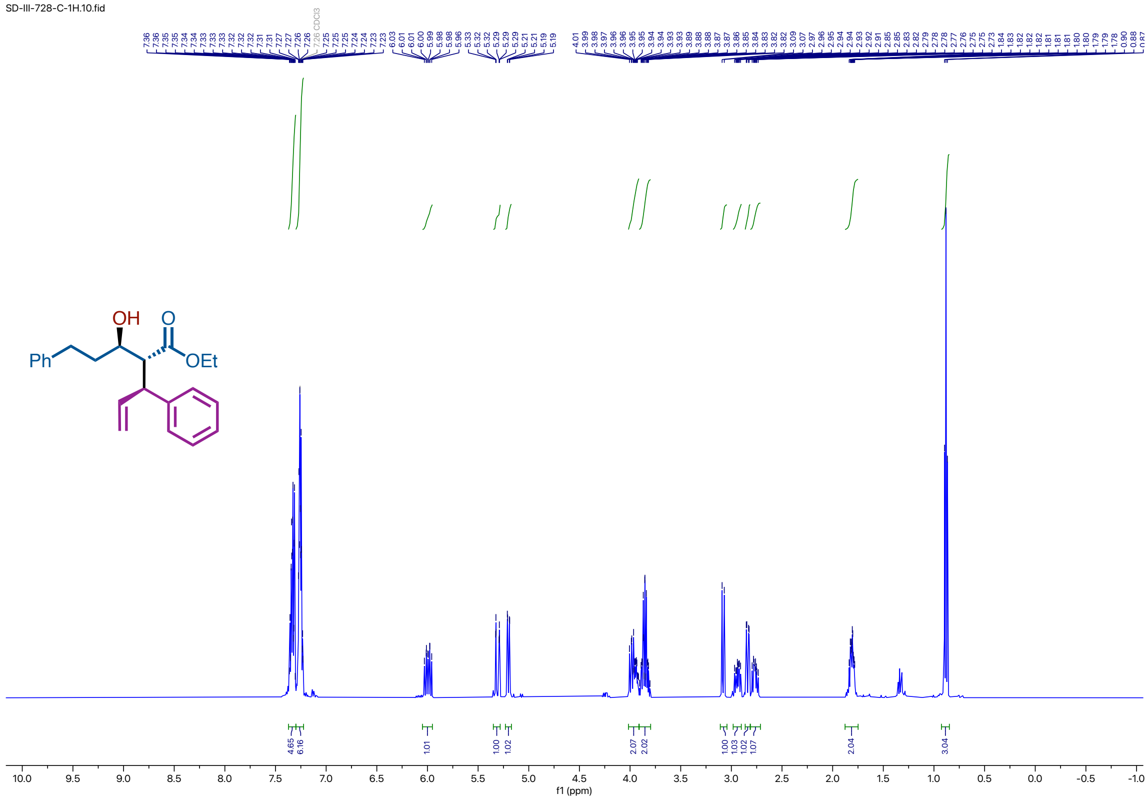

^{13}C NMR (126 MHz, $CDCl_3$) (17)

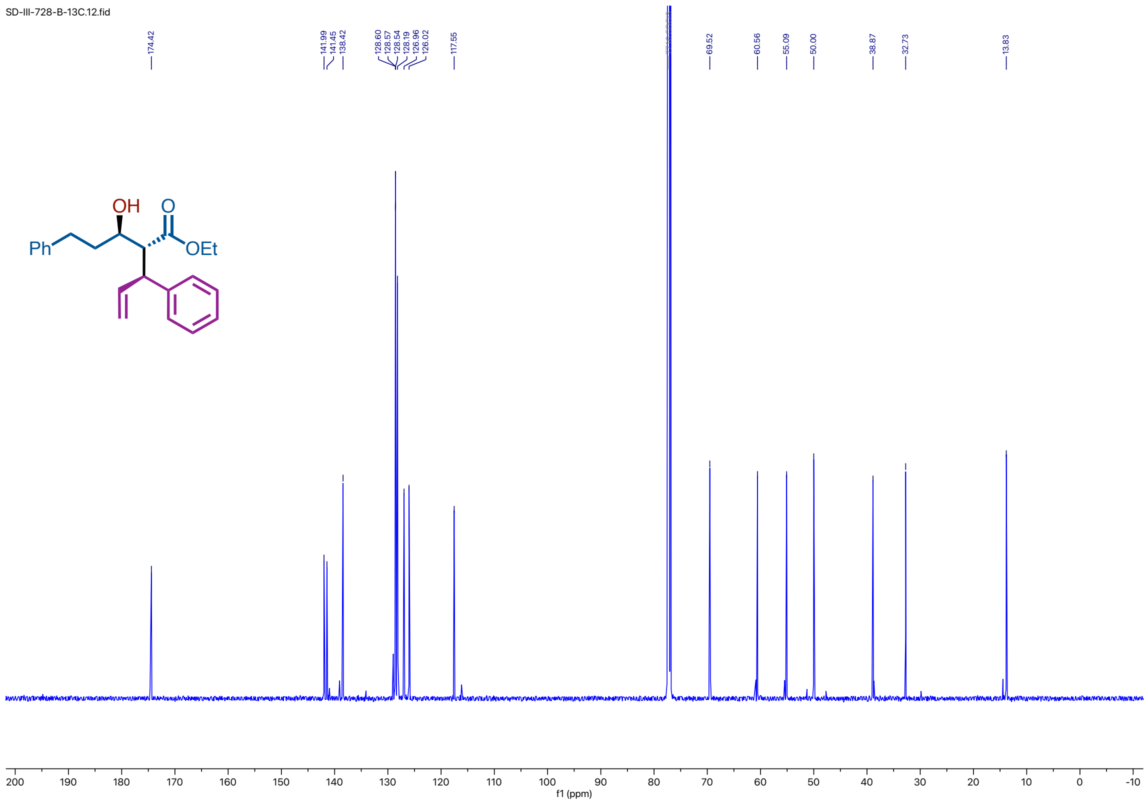

^{1}H NMR (500 MHz, $CDCl_3$) (18)

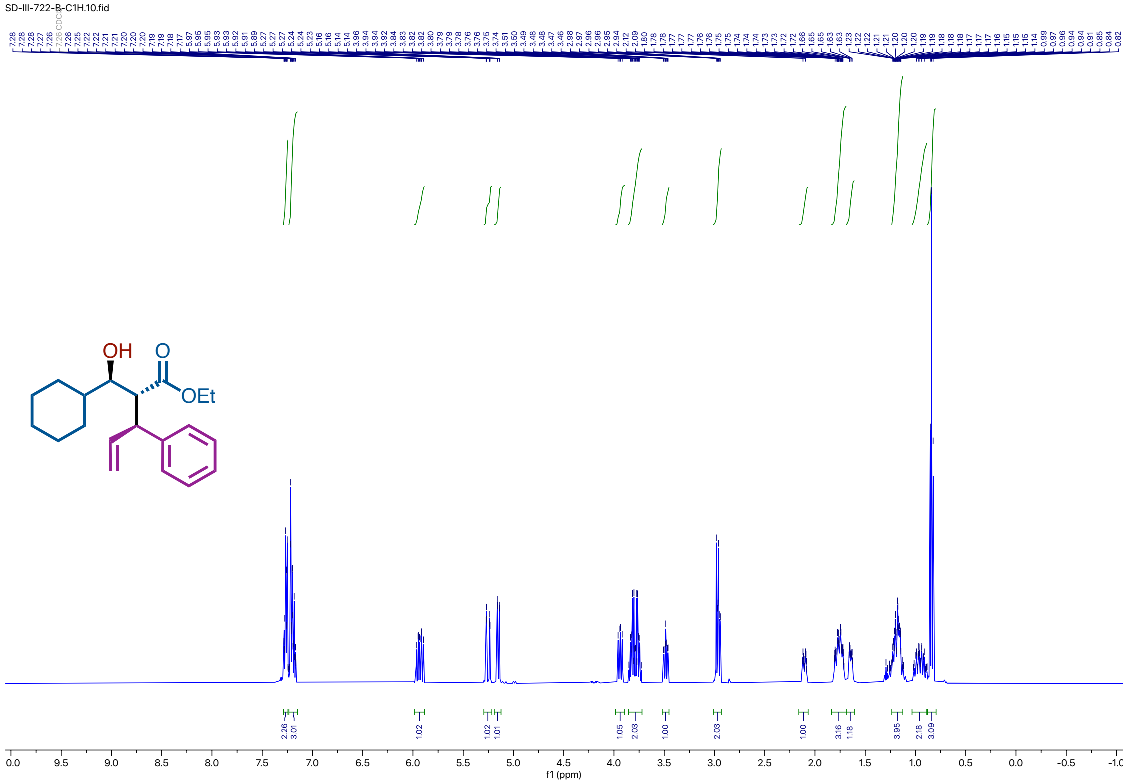

^{13}C NMR (126 MHz, $CDCl_3$) (18)

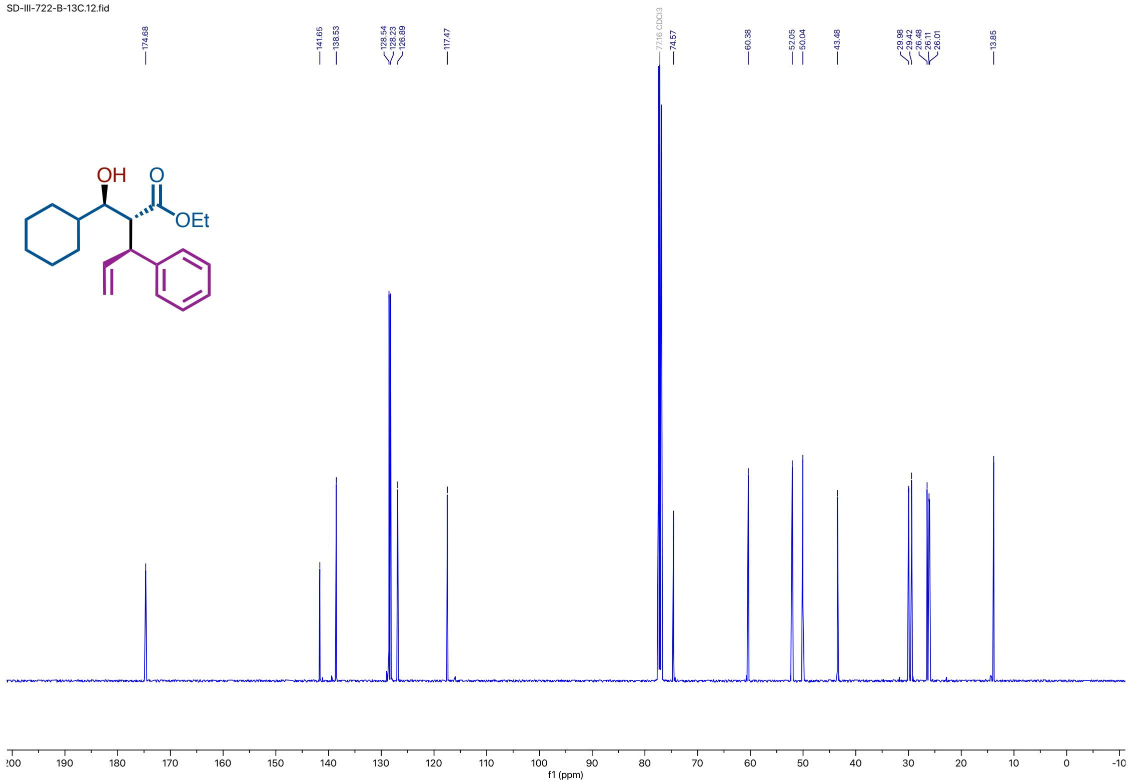

^{1}H NMR (500 MHz, $CDCl_3$) (19)

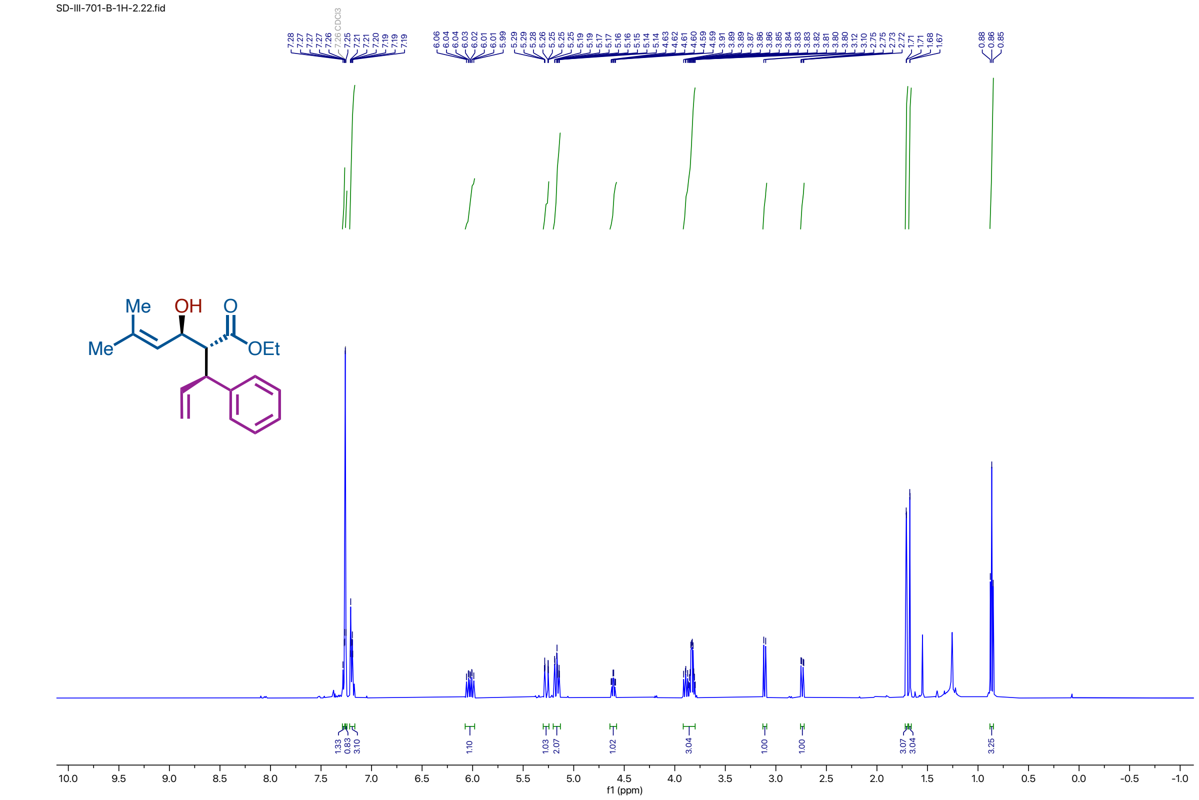

^{13}C NMR (126 MHz, $CDCl_3$) (19)

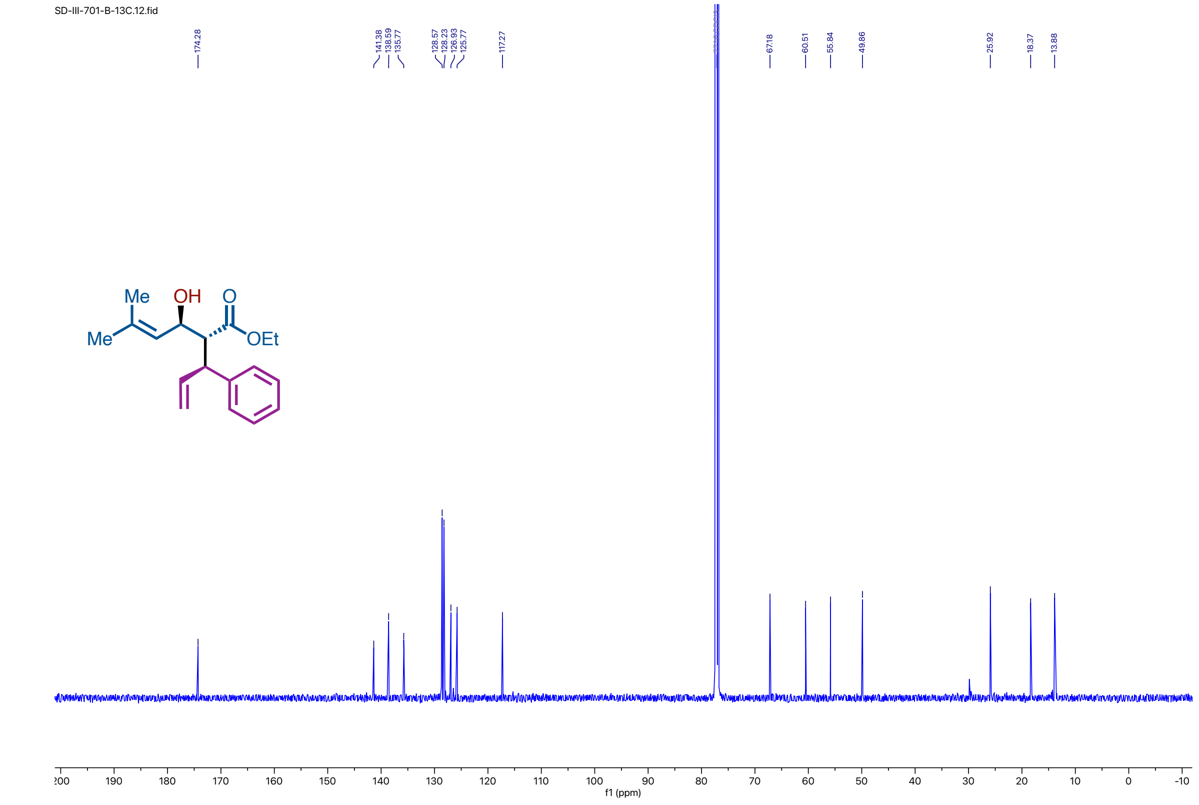

^{1}H NMR (500 MHz, $CDCl_3$) (20)

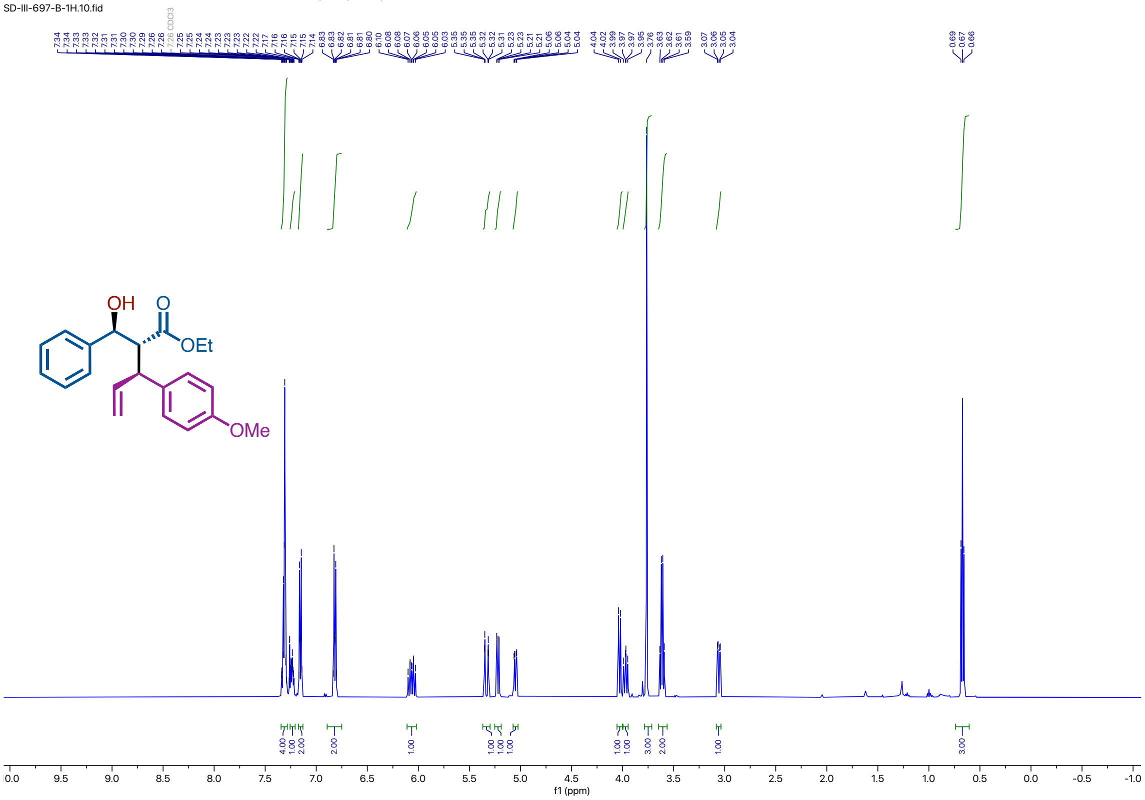

^{13}C NMR (126 MHz, $CDCl_3$) (20)

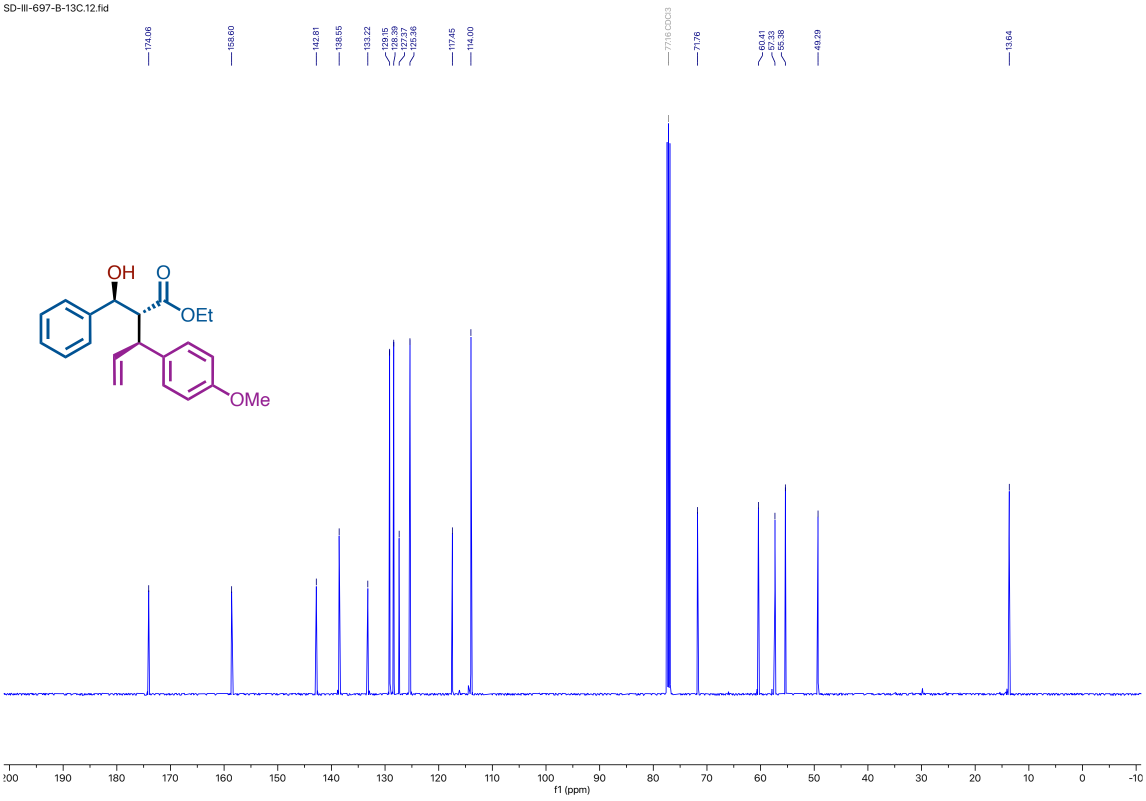

^{1}H NMR (500 MHz, $CDCl_3$) (21)

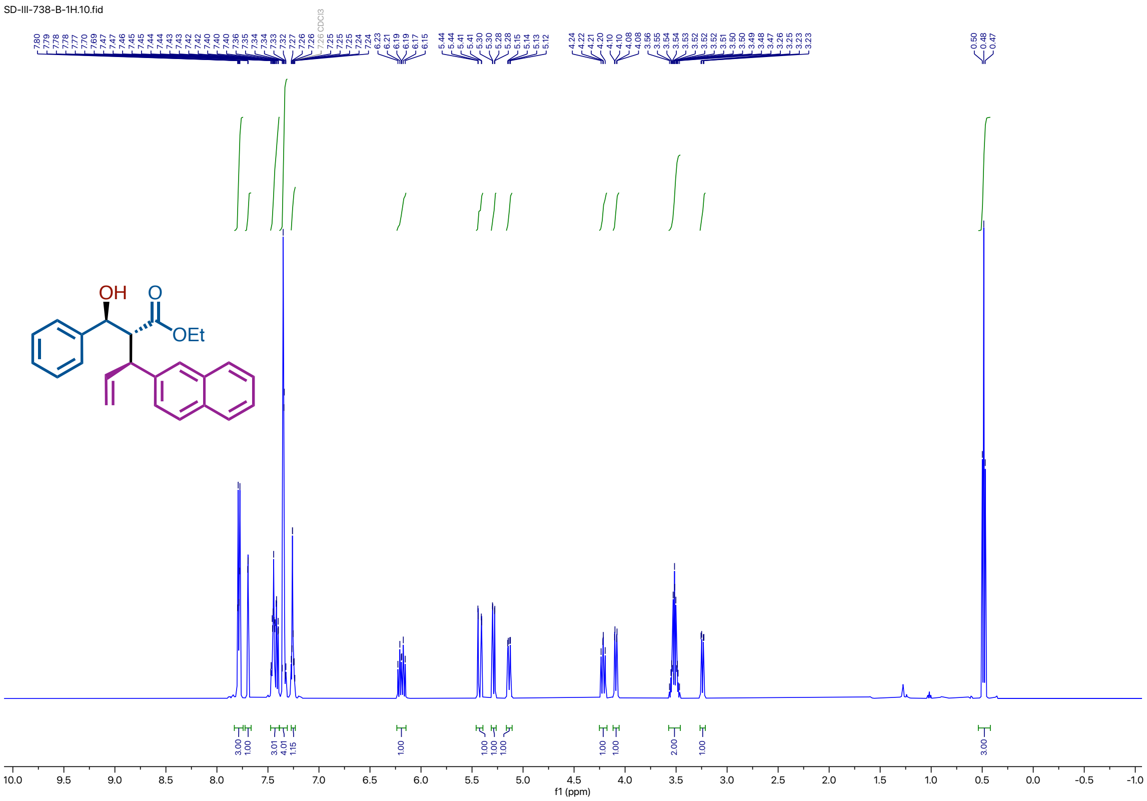

^{13}C NMR (126 MHz, $CDCl_3$) (21)

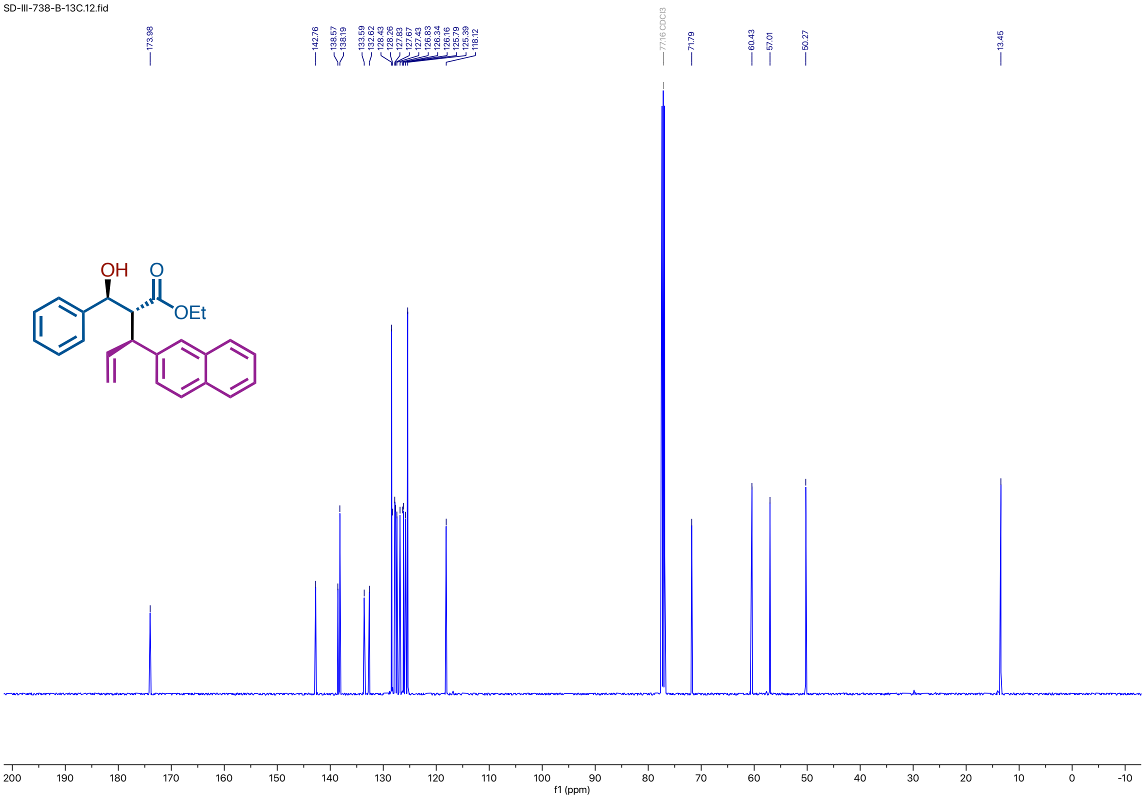

^{1}H NMR (500 MHz, $CDCl_3$) (22)

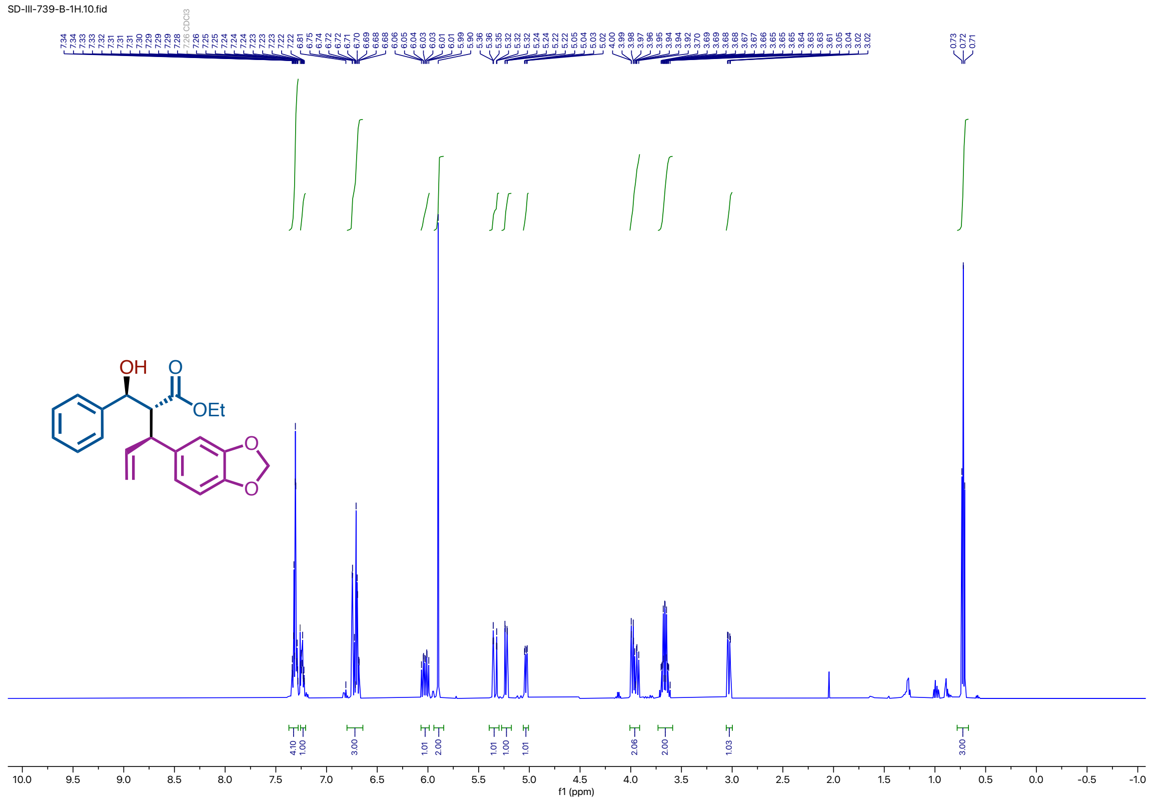

^{13}C NMR (126 MHz, $CDCl_3$) (22)

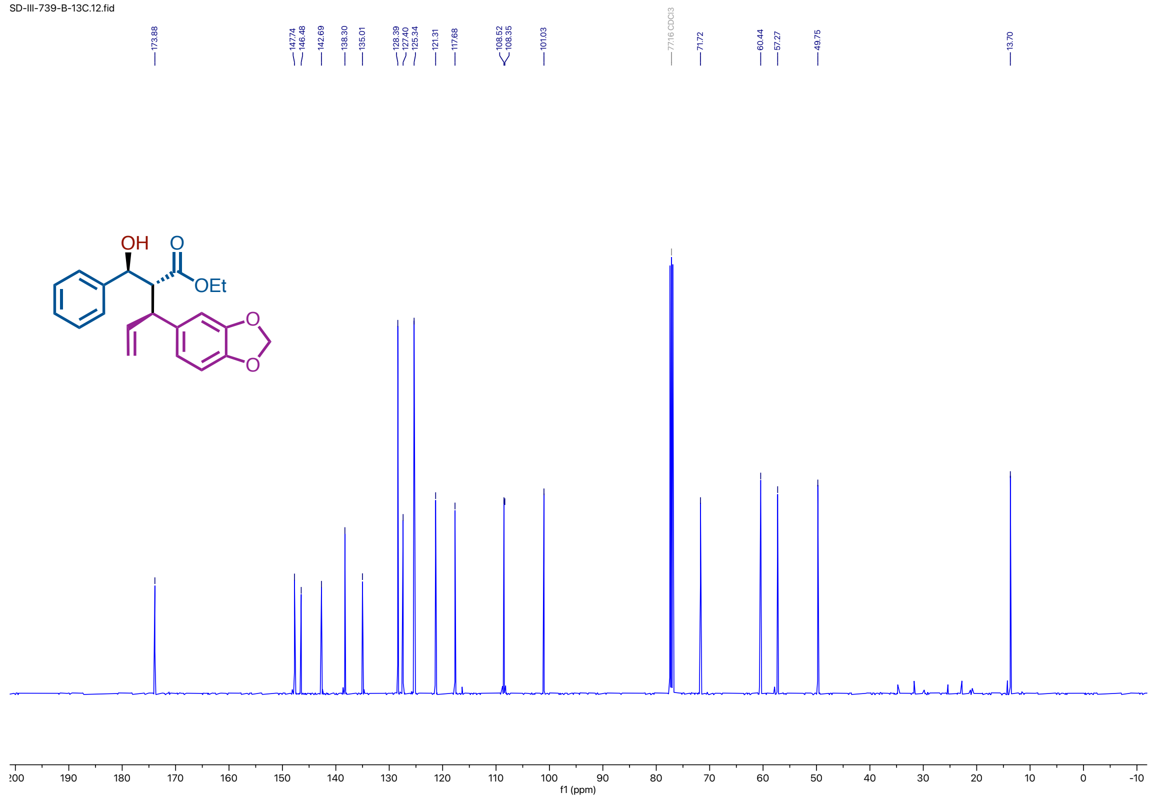

^{1}H NMR (500 MHz, CDCl$_3$) (23)

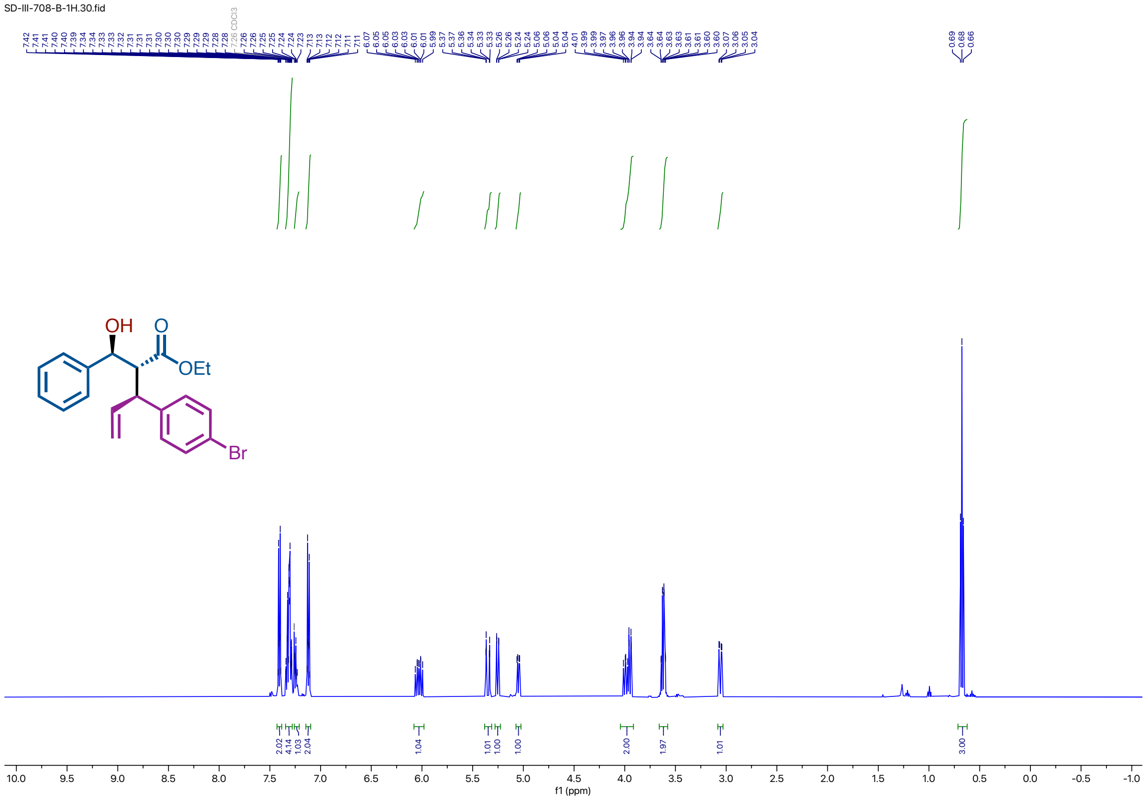

^{13}C NMR (126 MHz, CDCl$_3$) (23)

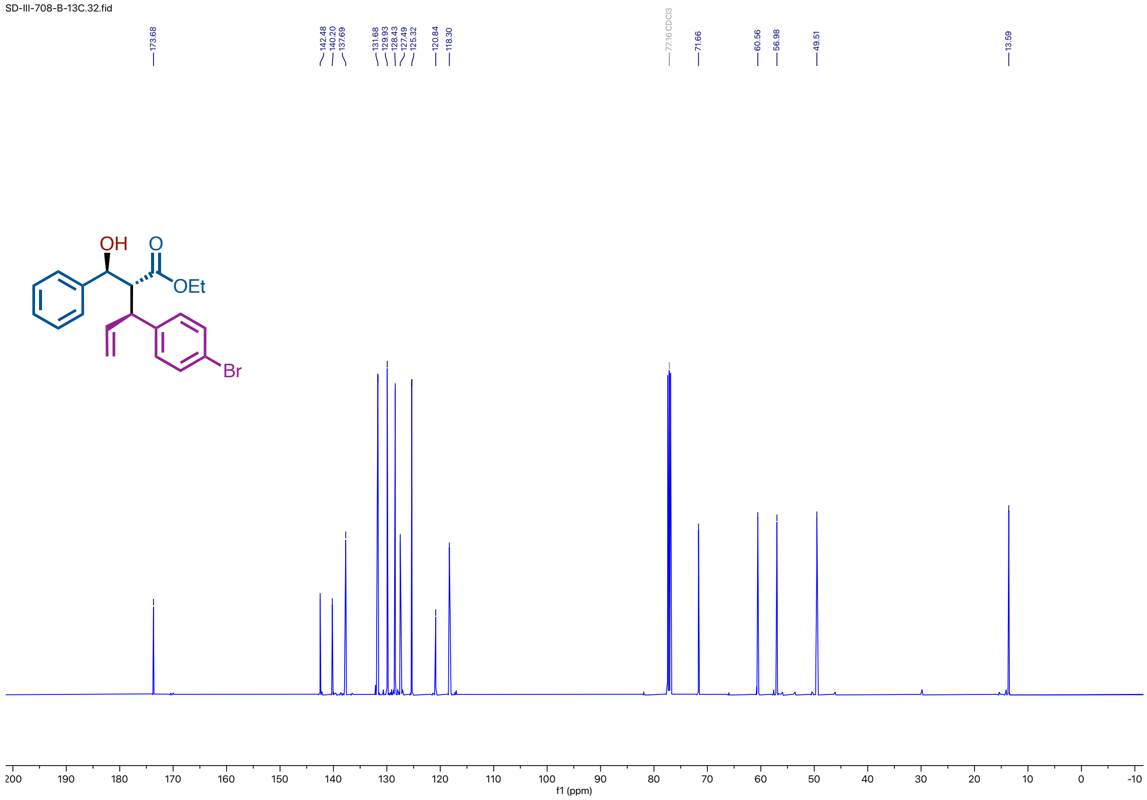

^{1}H NMR (500 MHz, $CDCl_3$) (24)

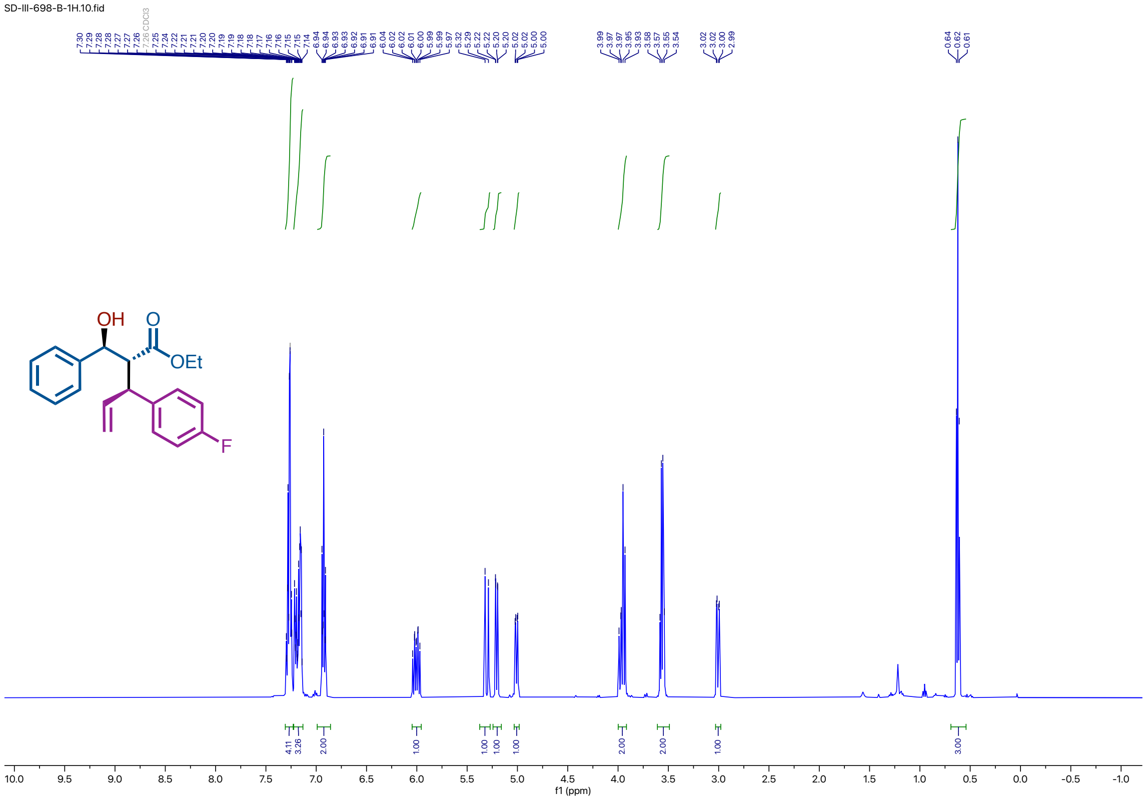

^{13}C NMR (126 MHz, $CDCl_3$) (24)

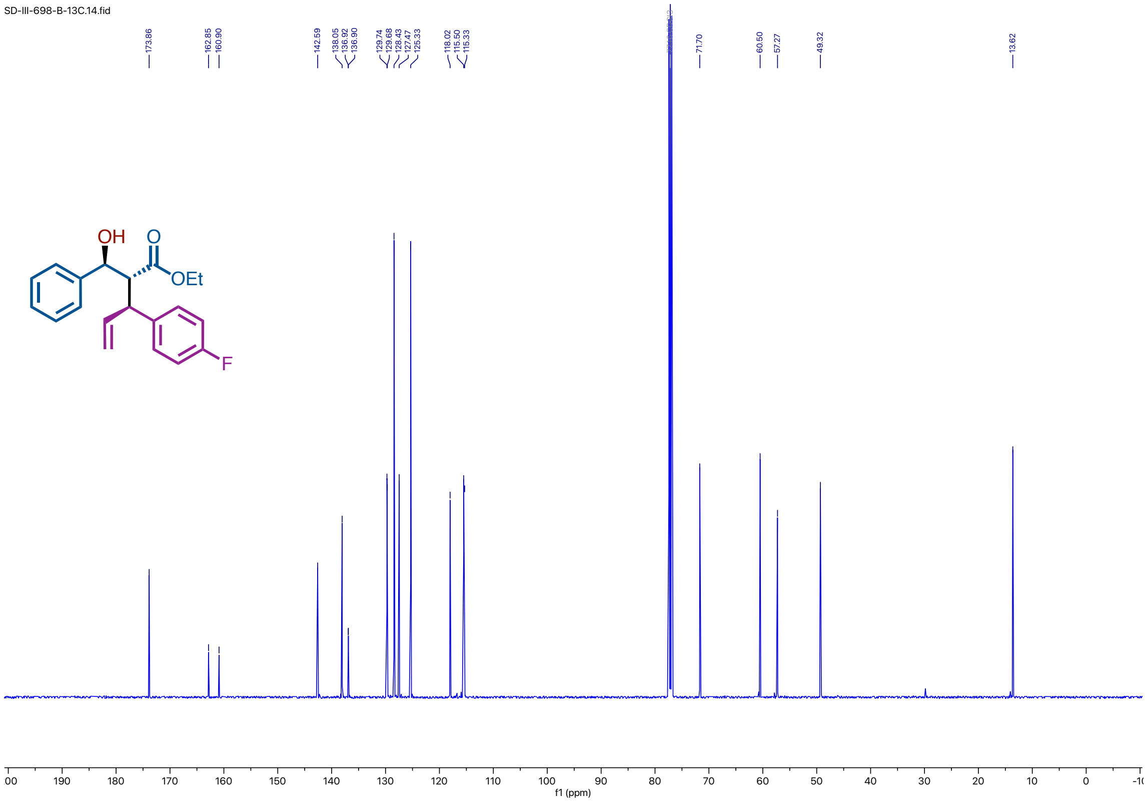

^{19}F NMR (471 MHz, $CDCl_3$) (24)

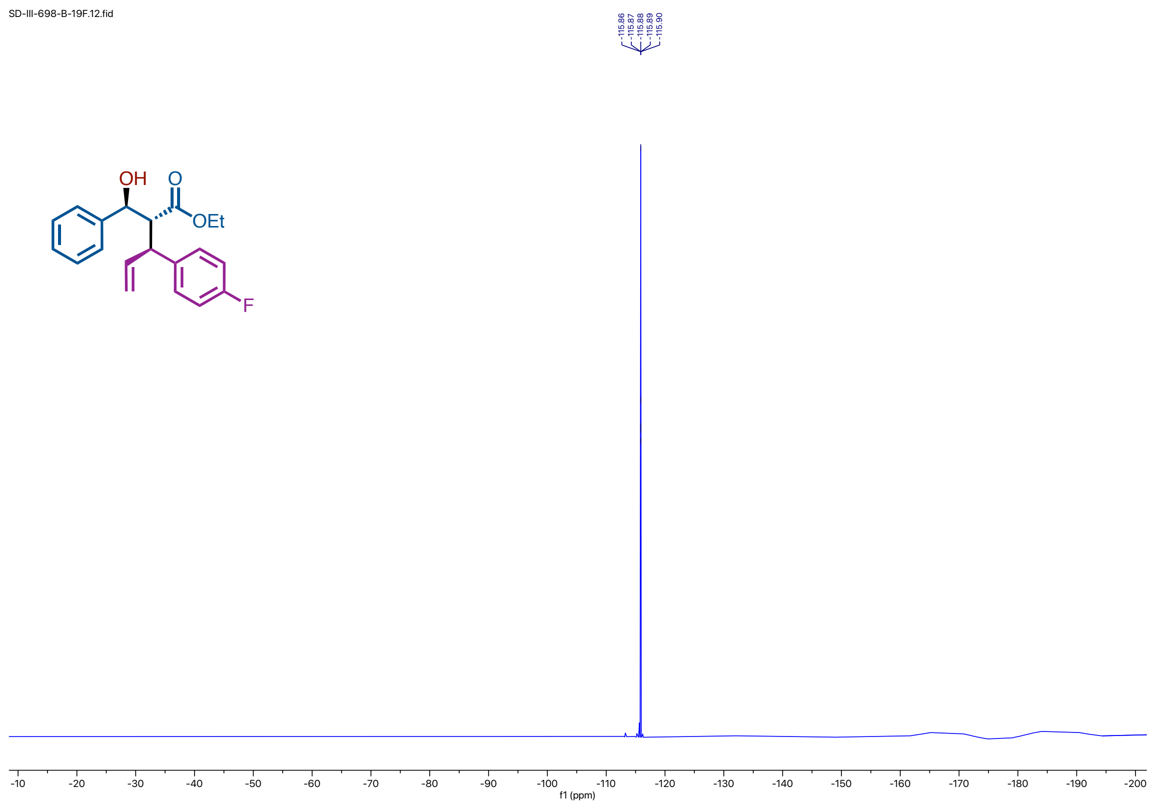

^{1}H NMR (500 MHz, $CDCl_3$) (25)

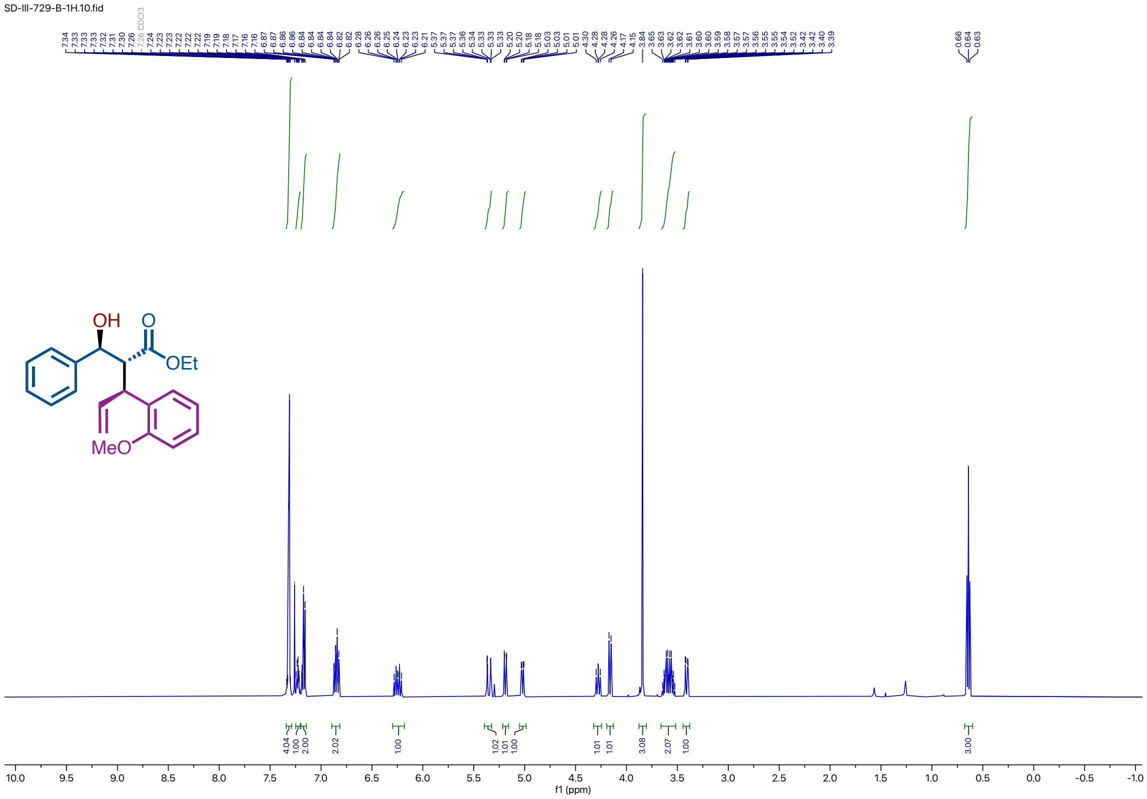

^{13}C NMR (126 MHz, $CDCl_3$) (25)

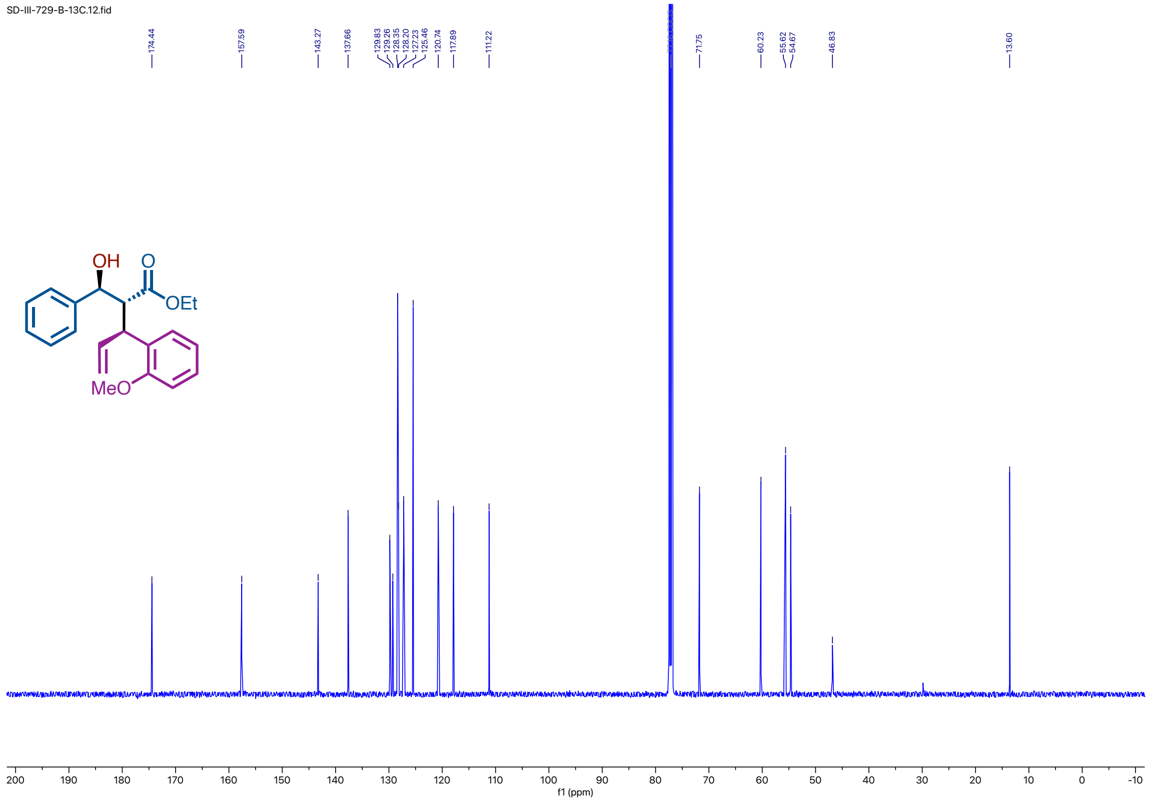

^{1}H NMR (500 MHz, $CDCl_3$) (26)

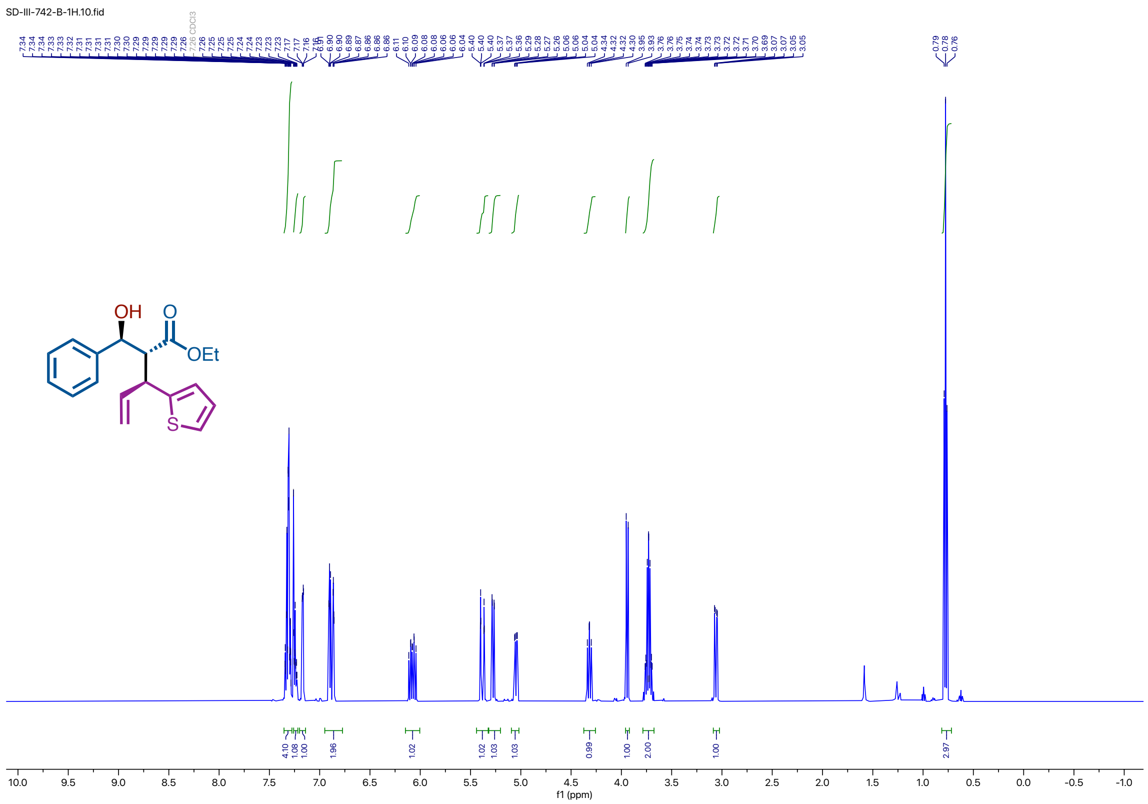

^{13}C NMR (126 MHz, $CDCl_3$) (26)

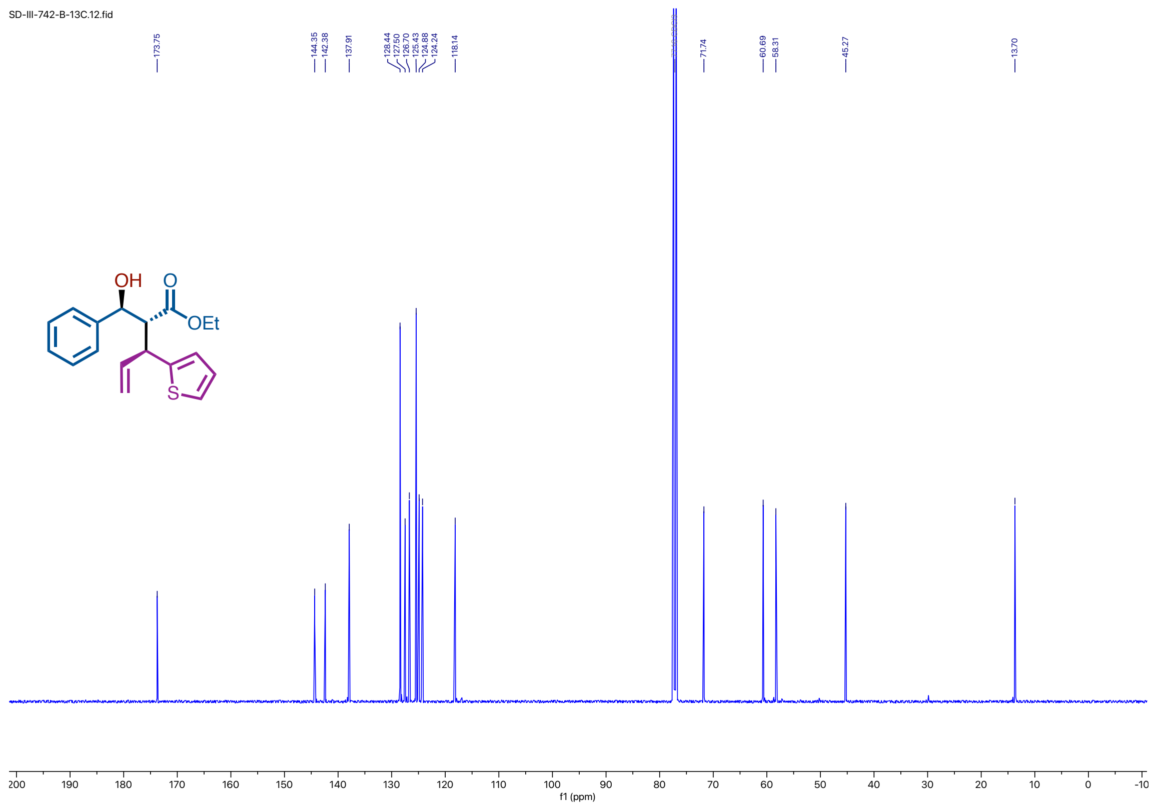

^{1}H NMR (500 MHz, $CDCl_3$) (27)

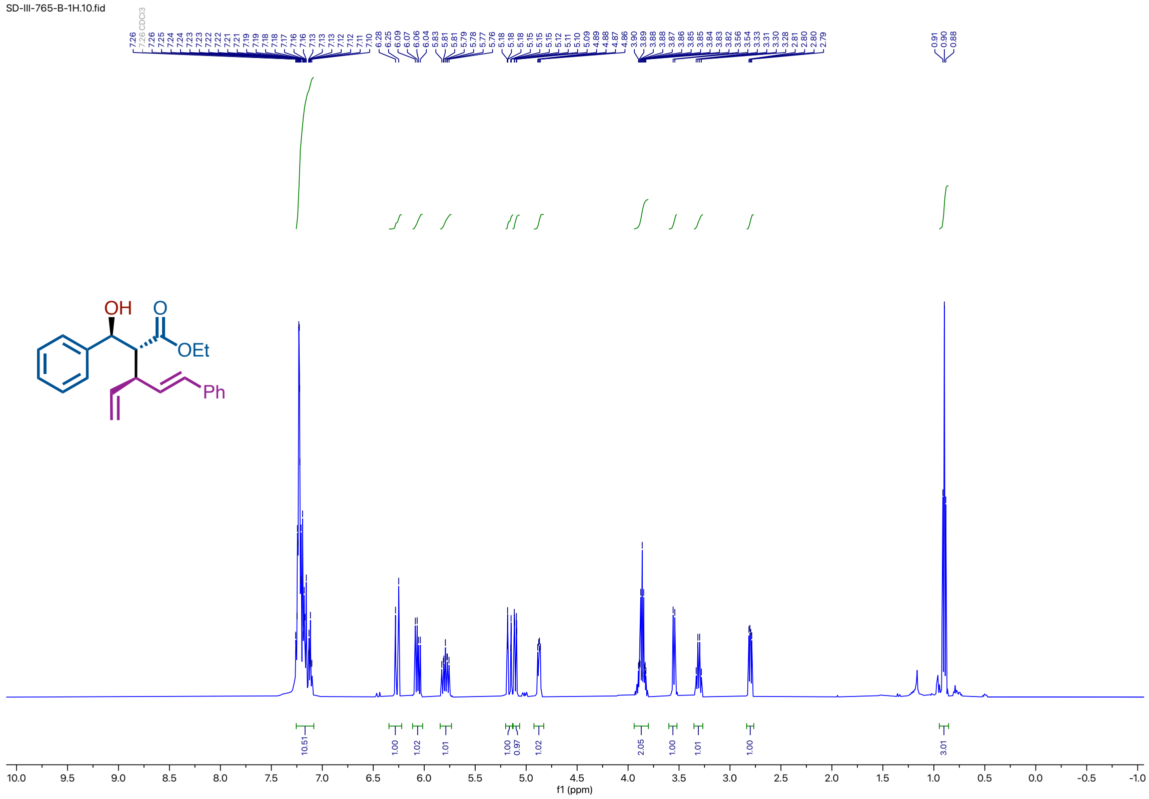

^{13}C NMR (126 MHz, $CDCl_3$) (27)

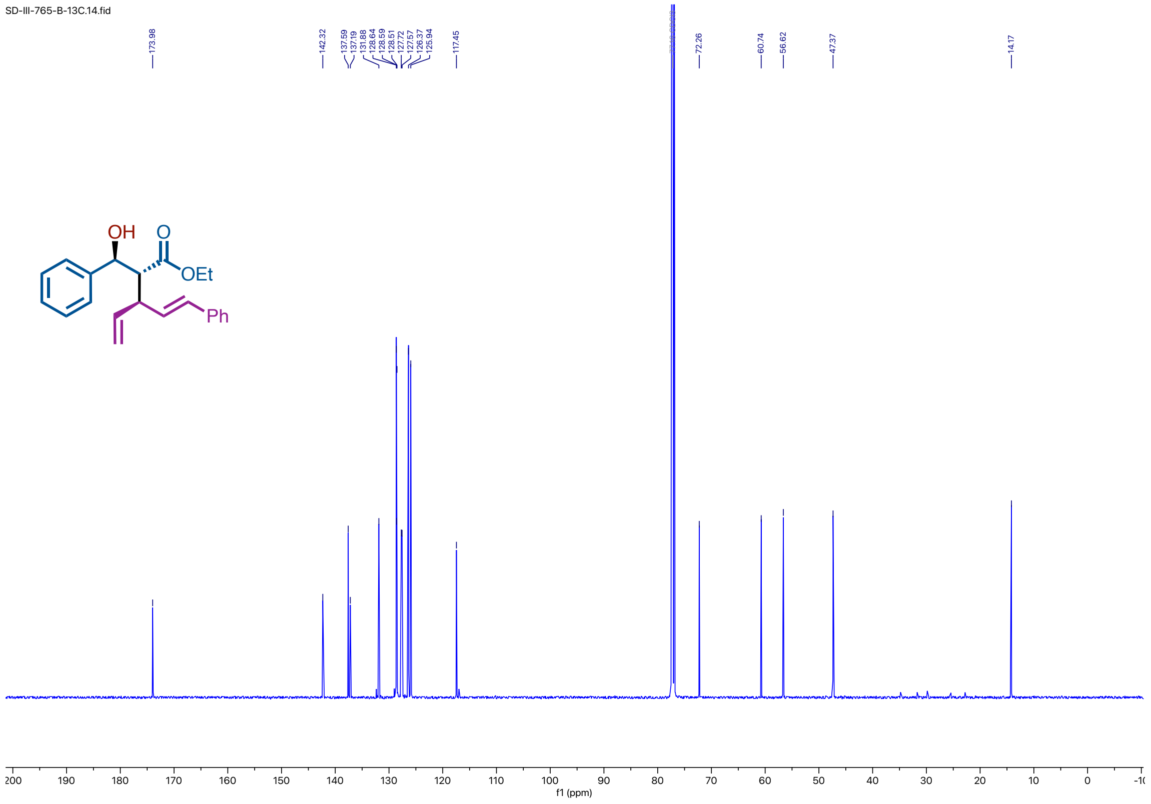

^{1}H NMR (500 MHz, $CDCl_3$) (28)

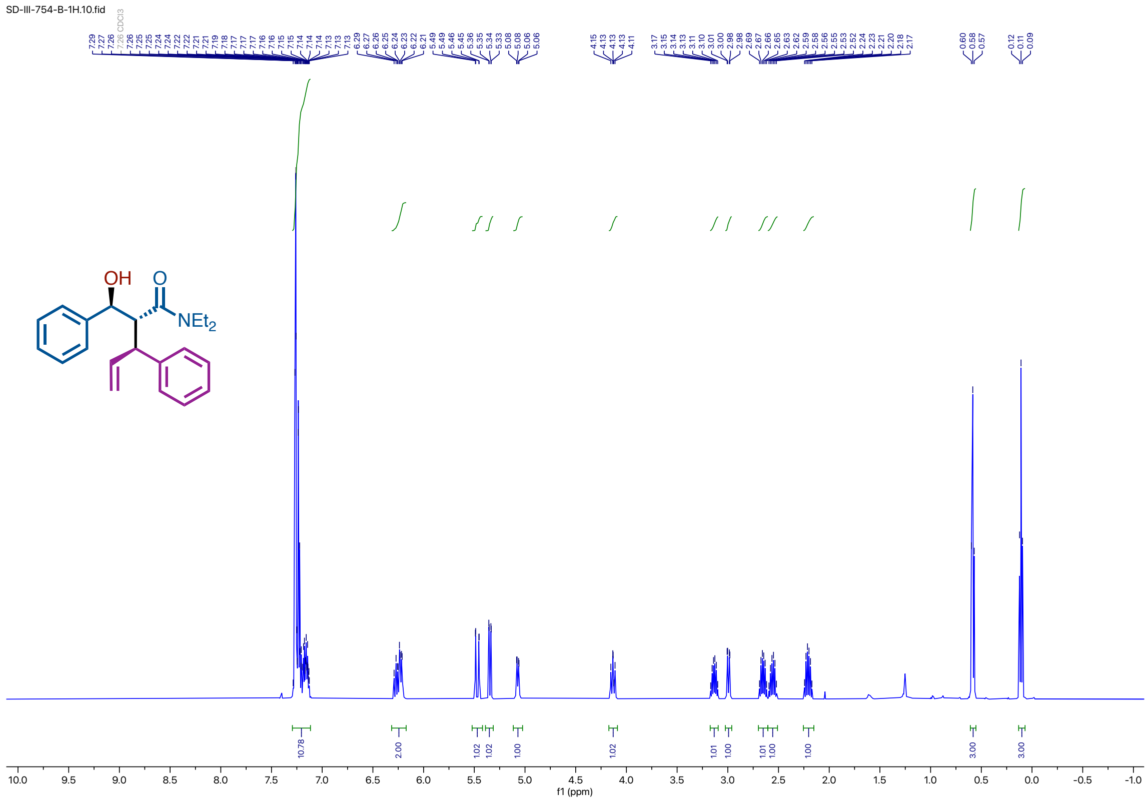

^{13}C NMR (126 MHz, $CDCl_3$) (28)

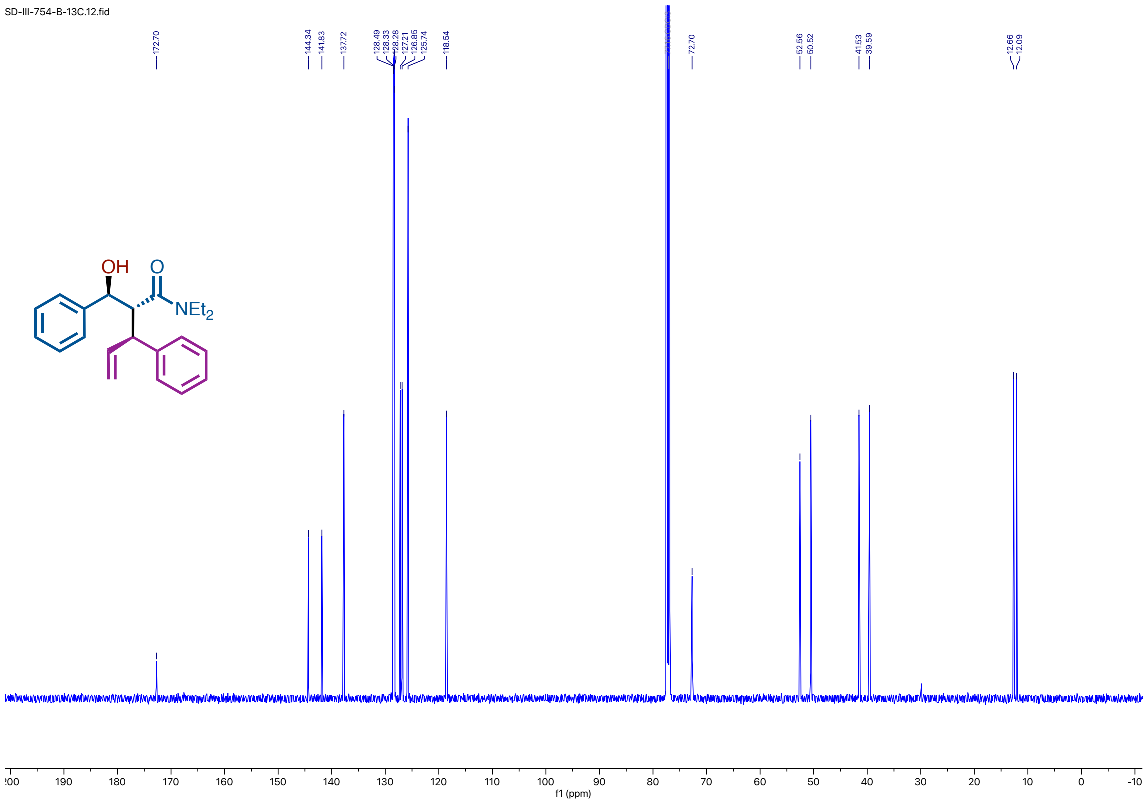

^{1}H NMR (500 MHz, $CDCl_3$) (3)

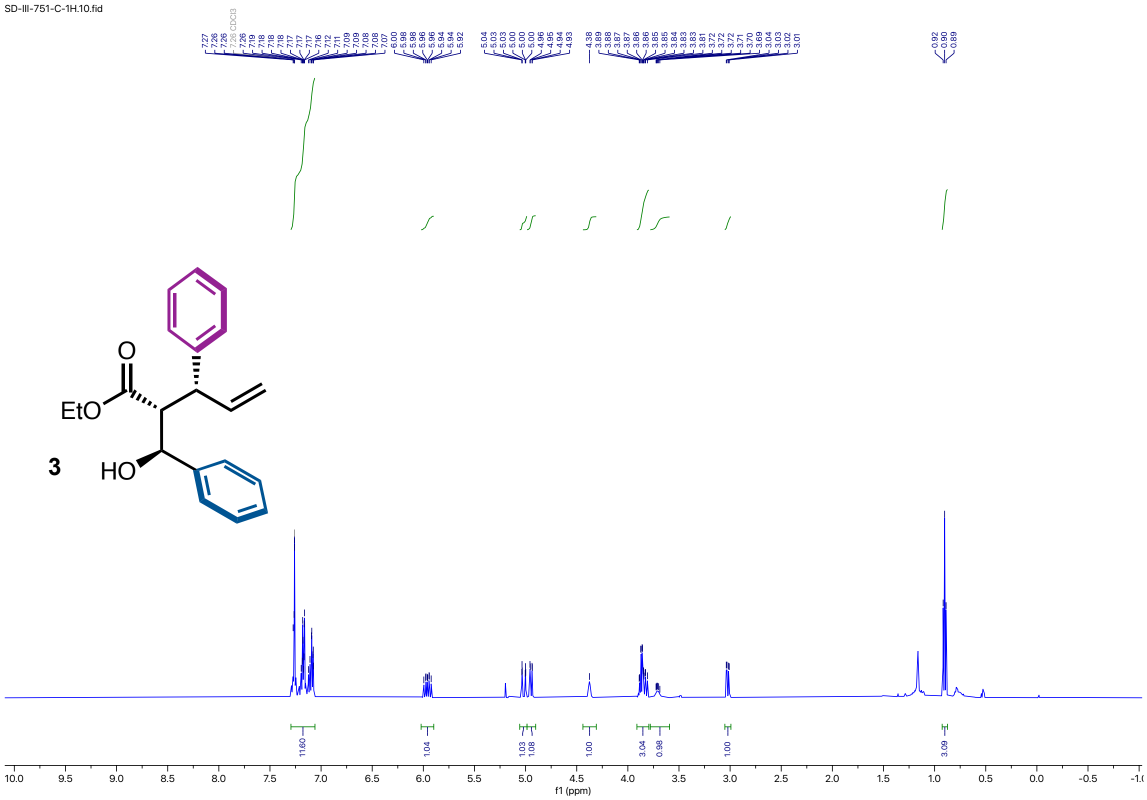

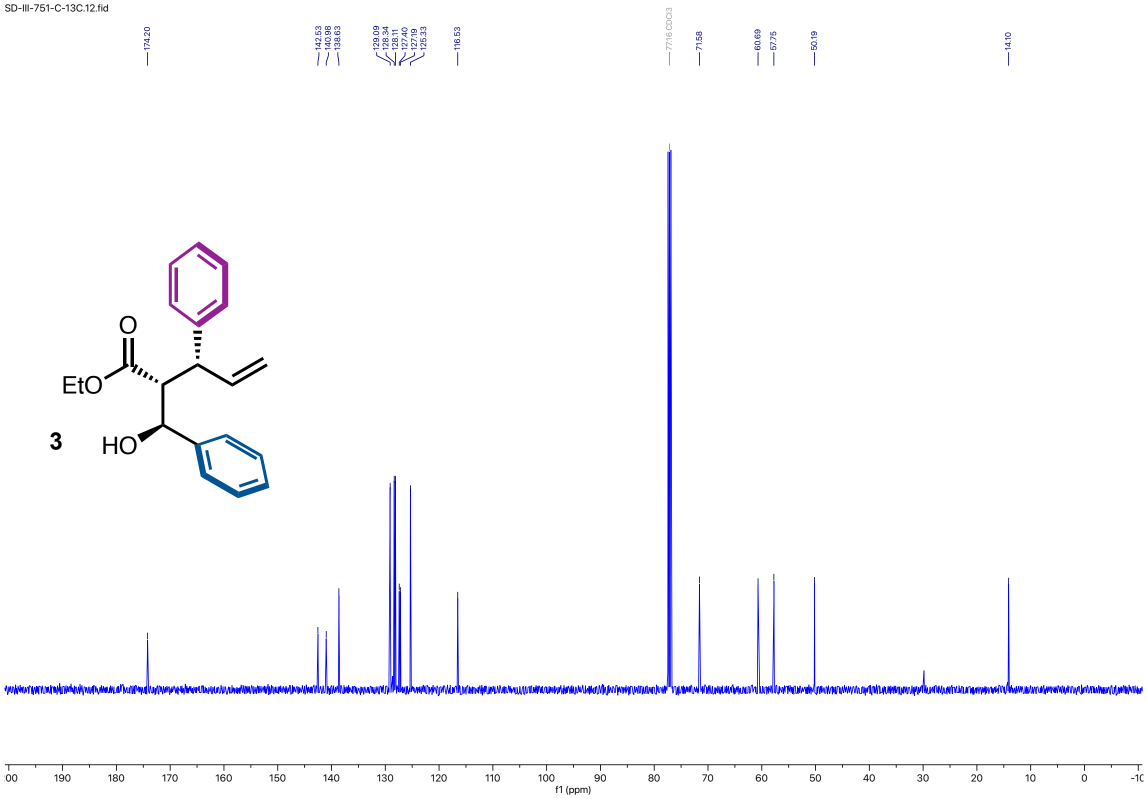

^{1}H NMR (500 MHz, $CDCl_3$) (5)

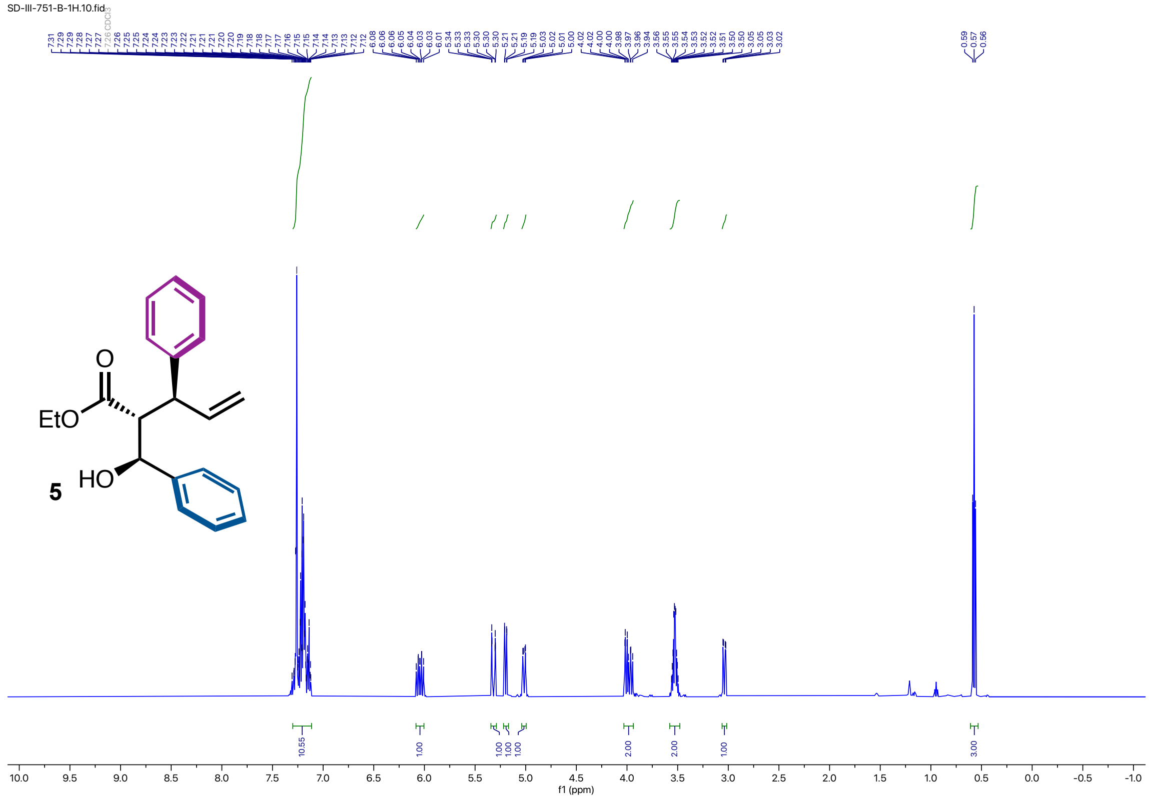

^{13}C NMR (126 MHz, $CDCl_3$) (5)

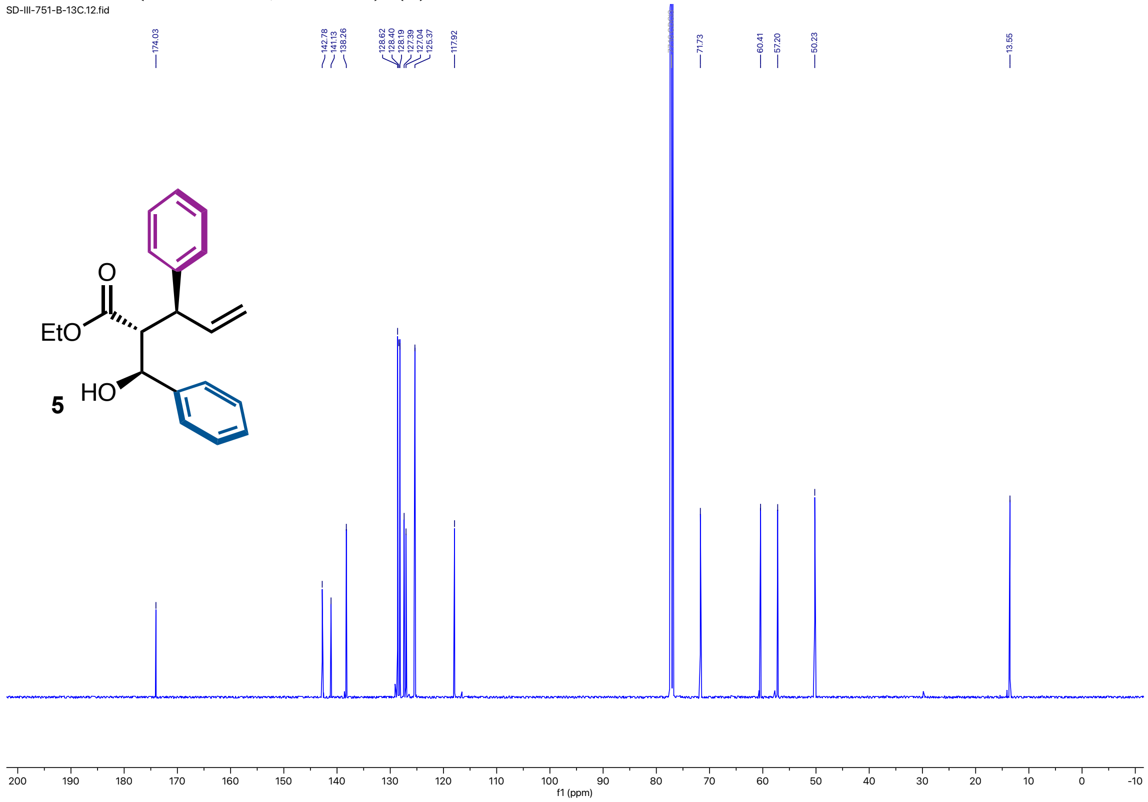

^{1}H NMR (500 MHz, CDCl$_3$) (30)

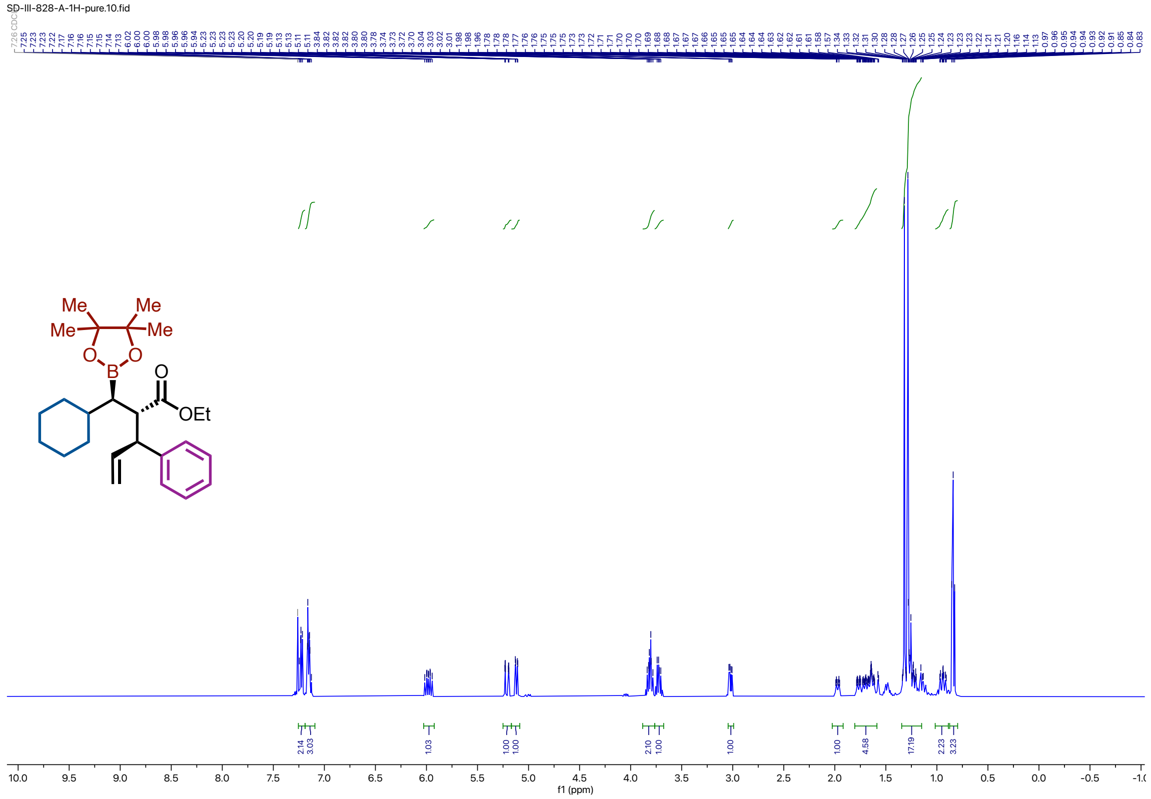

^{13}C NMR (126 MHz, CDCl$_3$) (30)

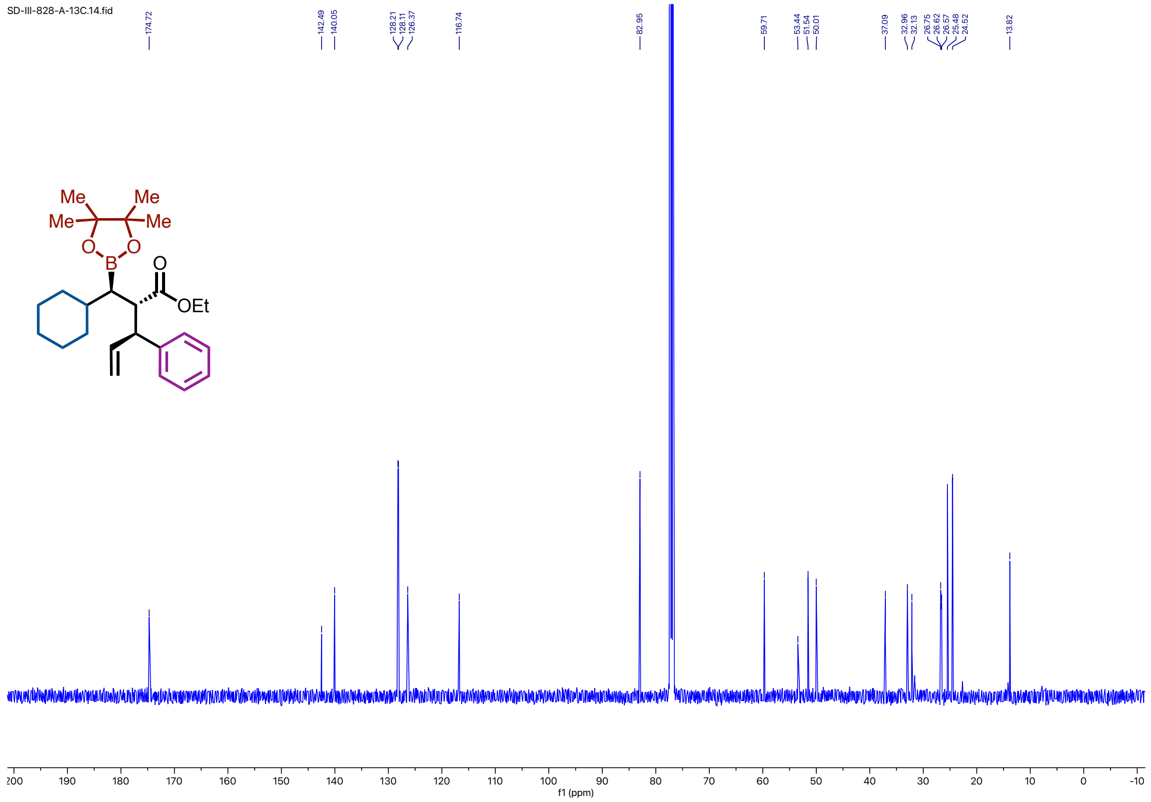

^{1}H NMR (500 MHz, $CDCl_3$) (31)

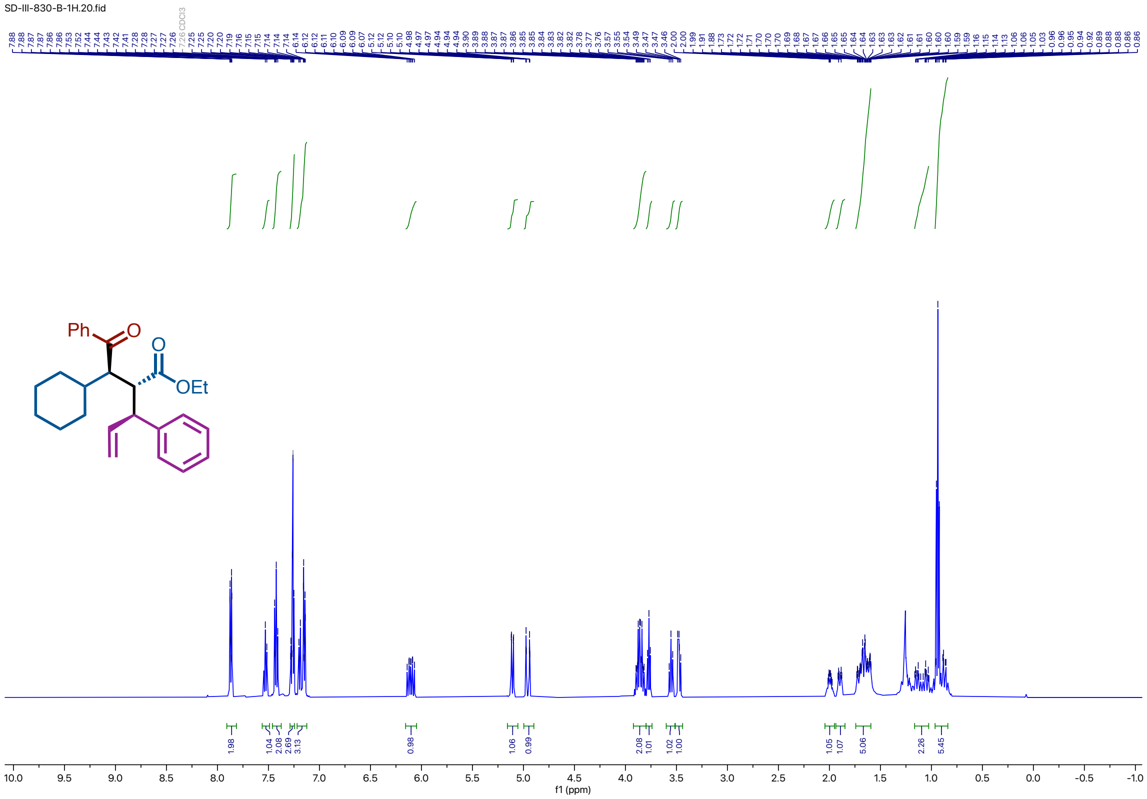

^{13}C NMR (126 MHz, $CDCl_3$) (31)

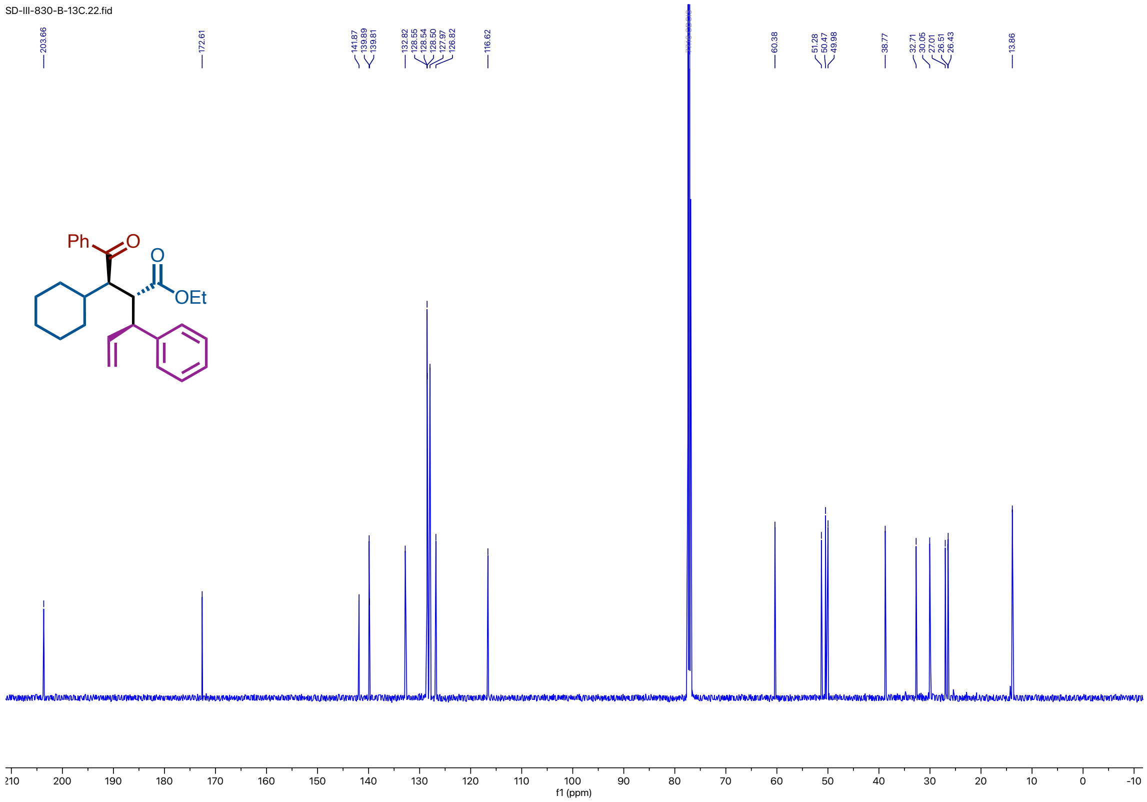

^{1}H NMR (500 MHz, CDCl$_3$) (32)

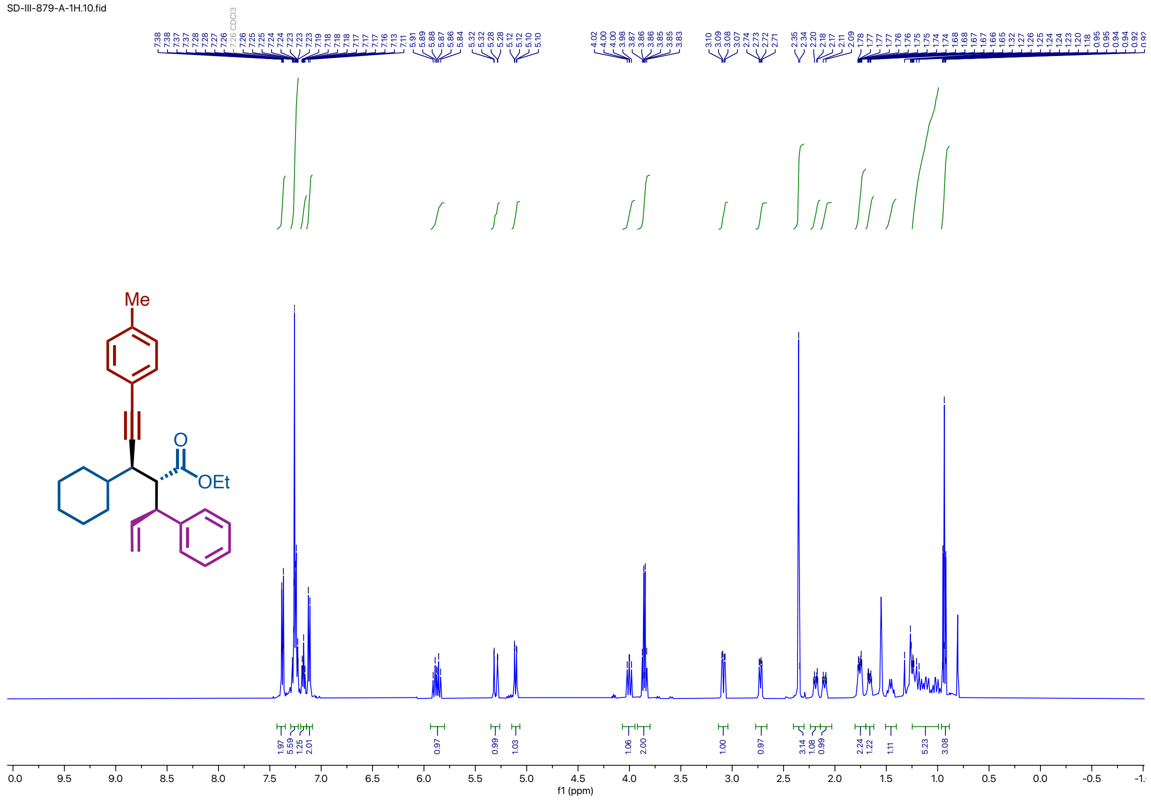

^{13}C NMR (126 MHz, CDCl$_3$) (32)

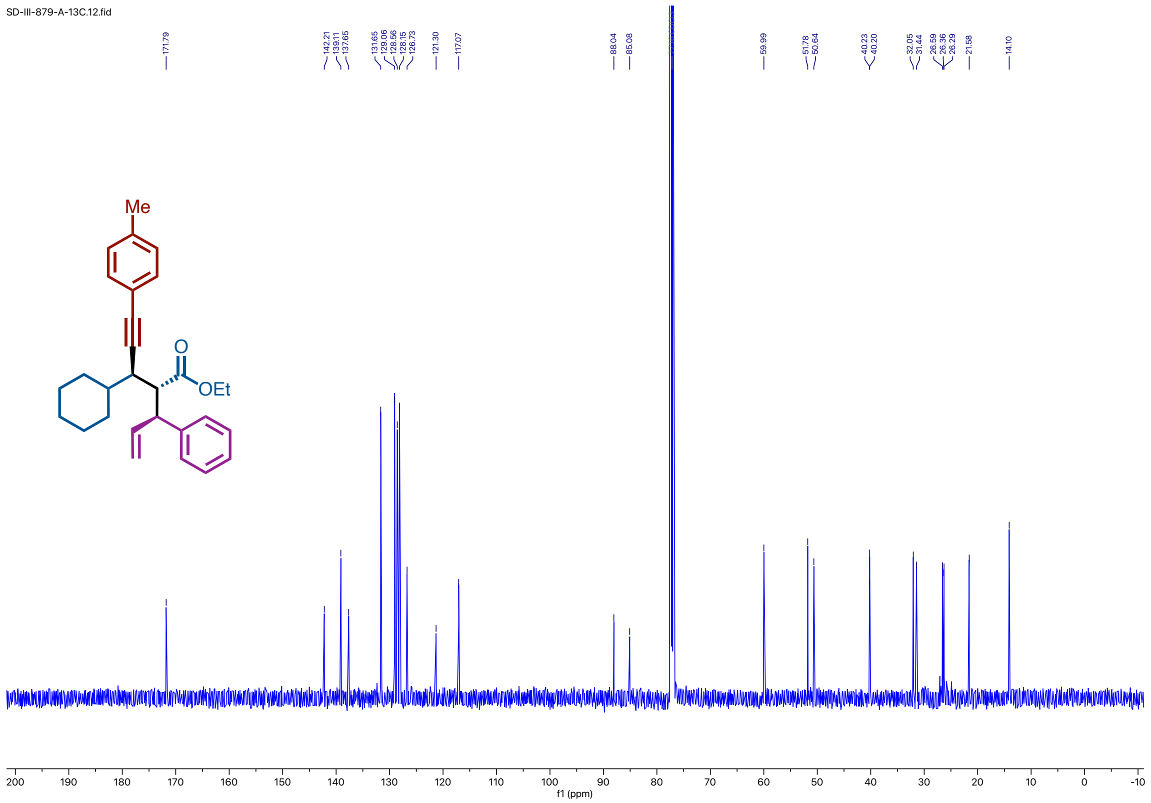

^{1}H NMR (500 MHz, $CDCl_3$) (33)

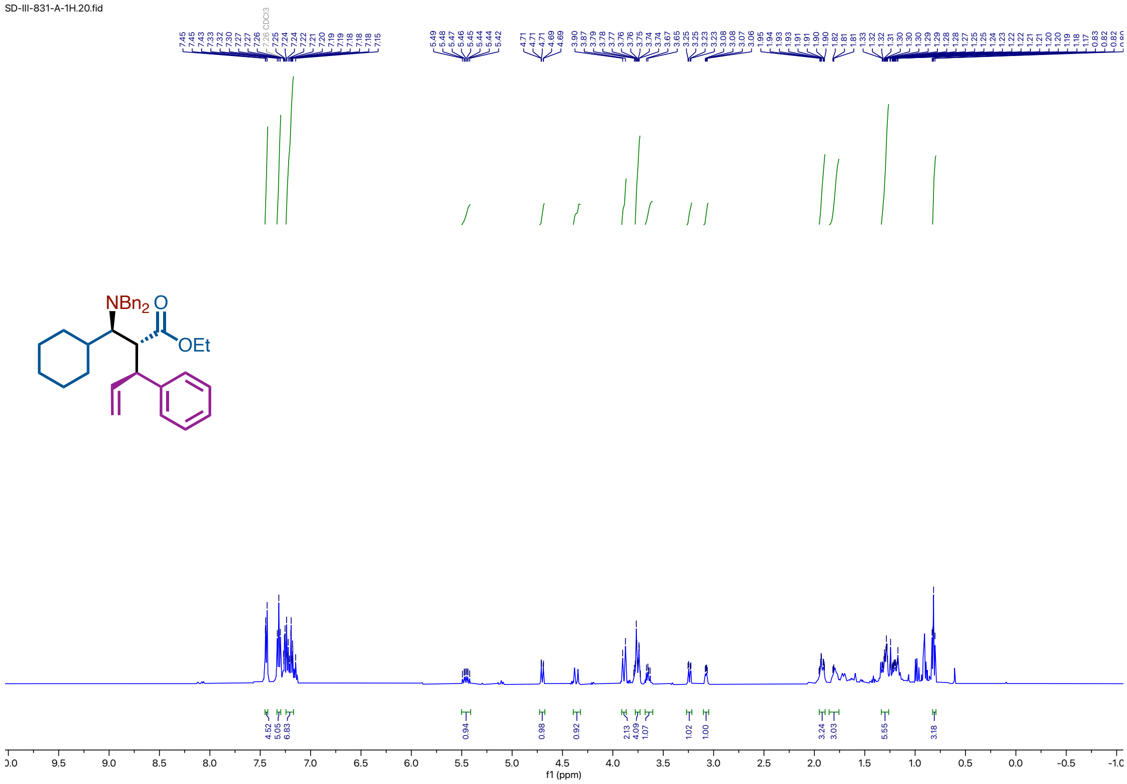

^{13}C NMR (126 MHz, $CDCl_3$) (33)

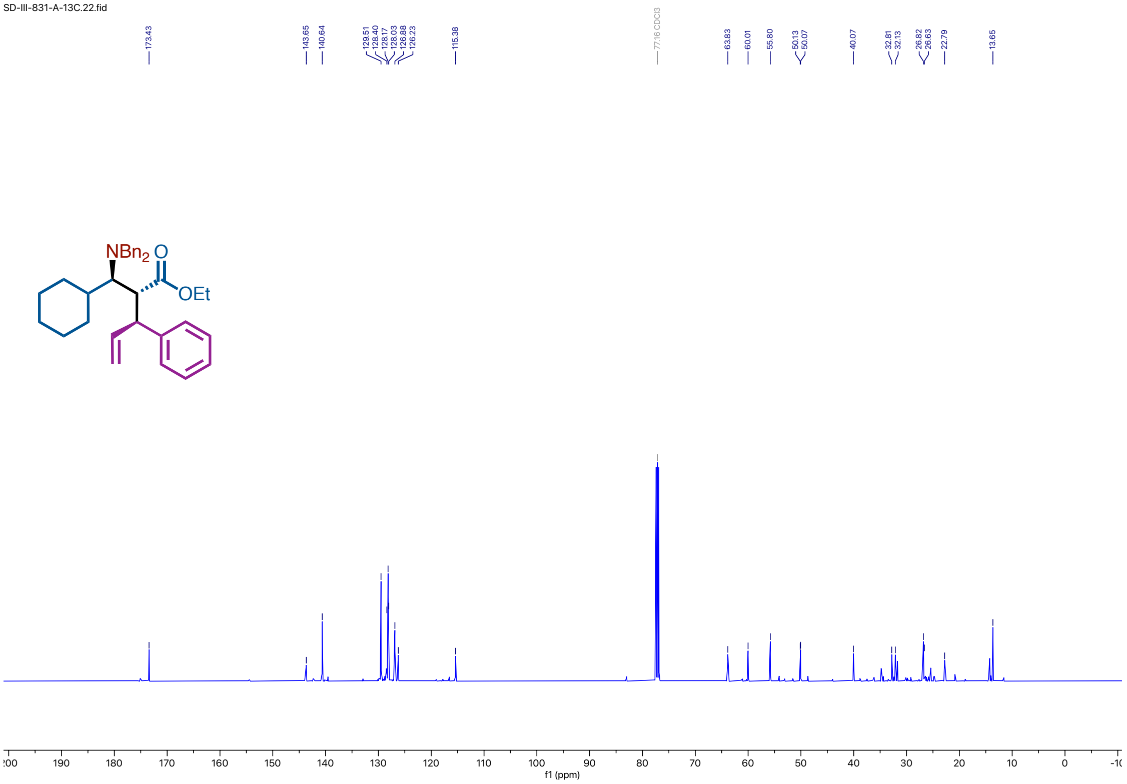

^{1}H NMR (500 MHz, $CDCl_3$) (340

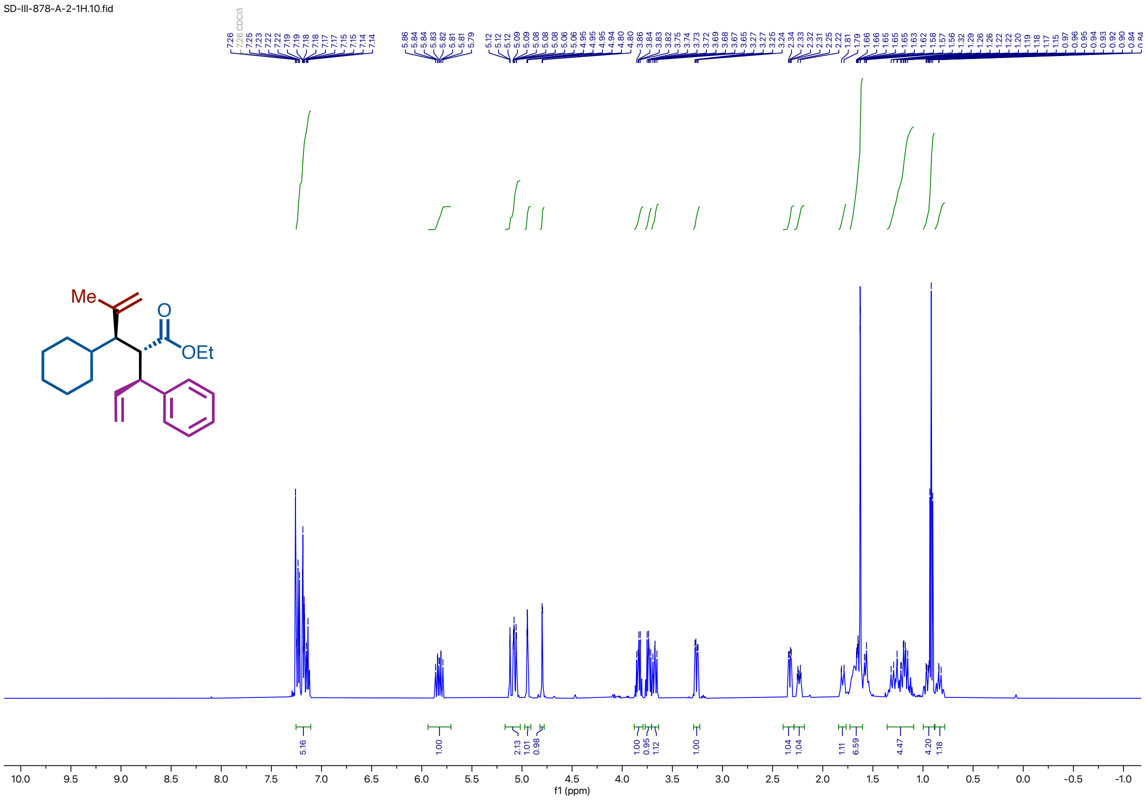

^{13}C NMR (126 MHz, $CDCl_3$) (34)

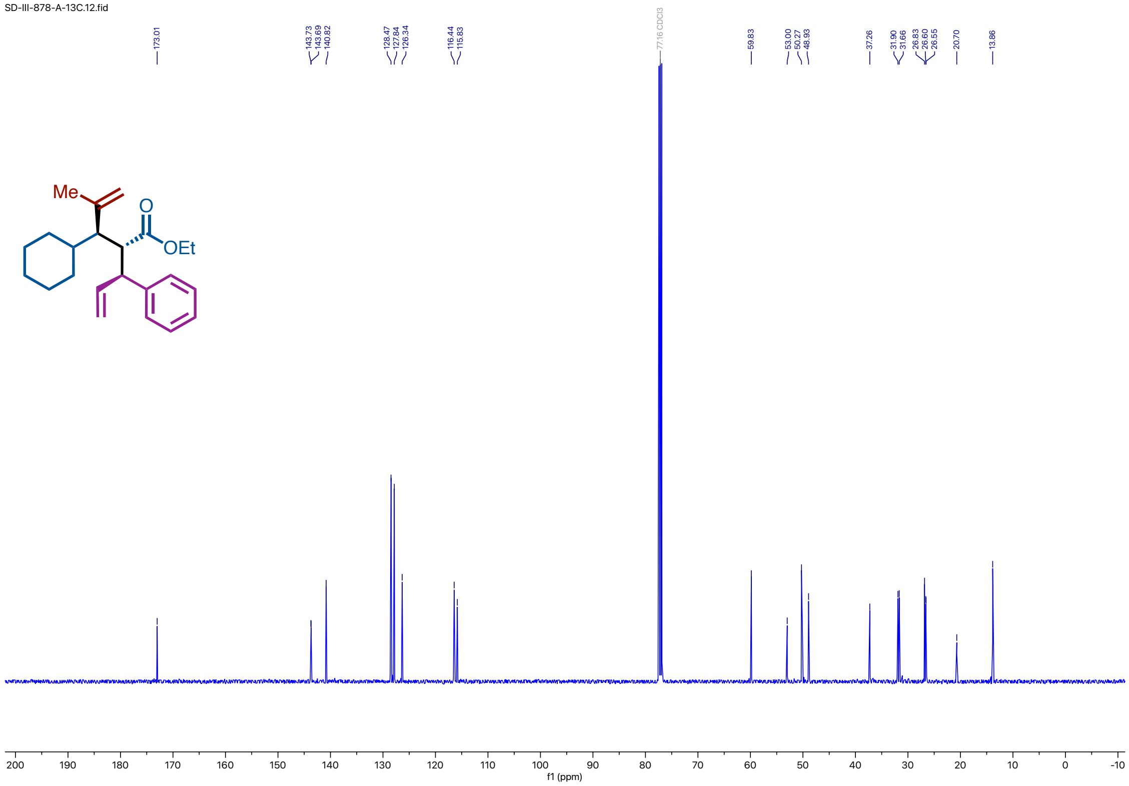

^{1}H NMR (500 MHz, CDCl$_3$) (35)

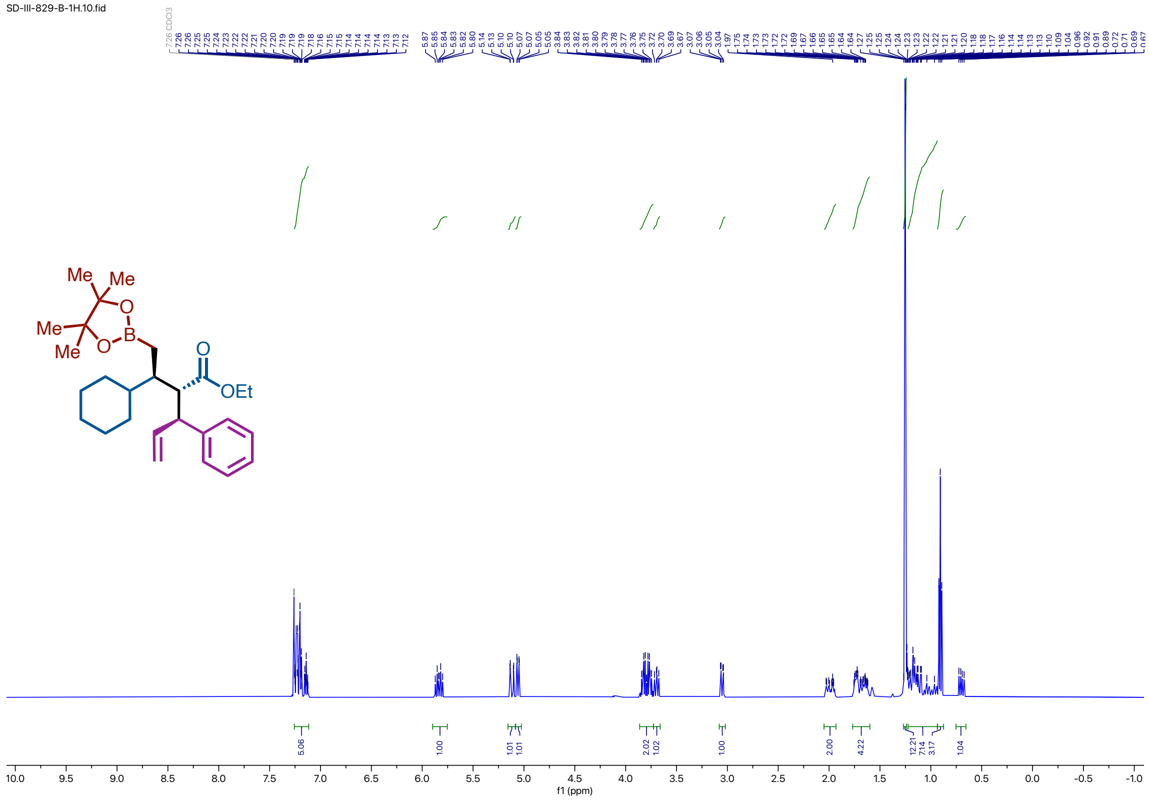

^{13}C NMR (126 MHz, CDCl$_3$) (35)

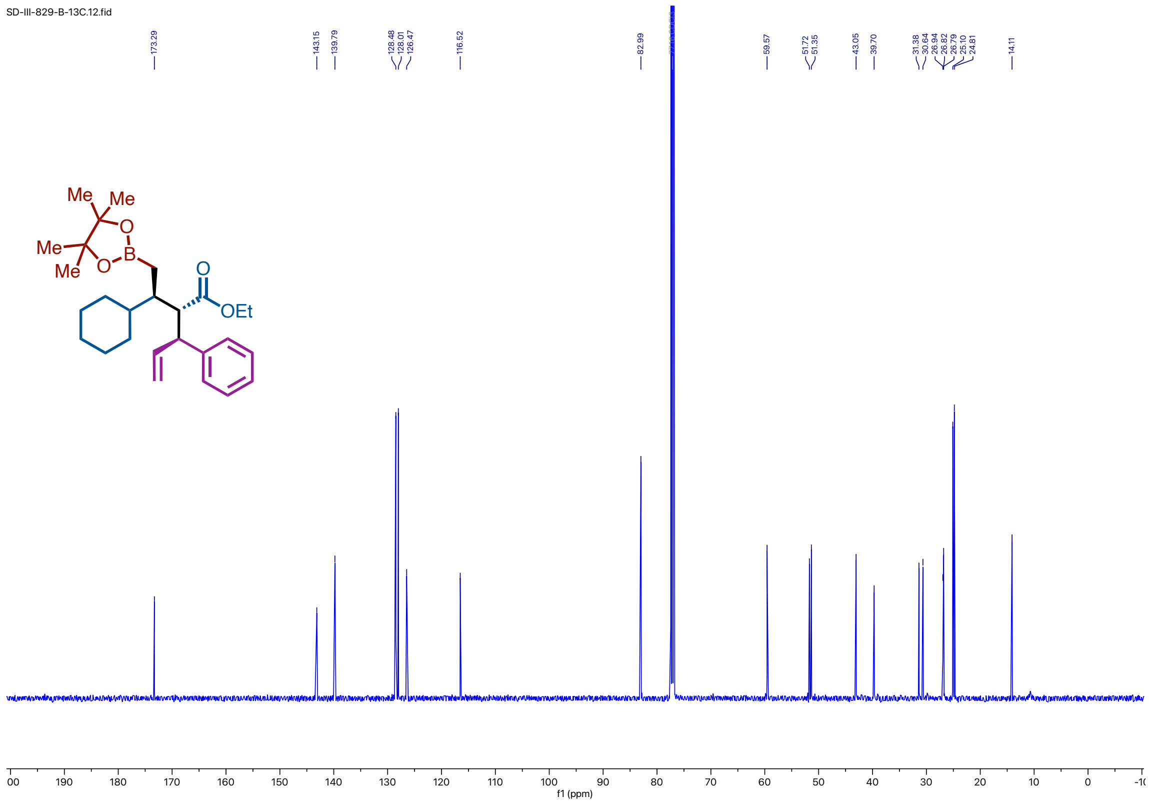

^{1}H NMR (500 MHz, $CDCl_3$) (36)

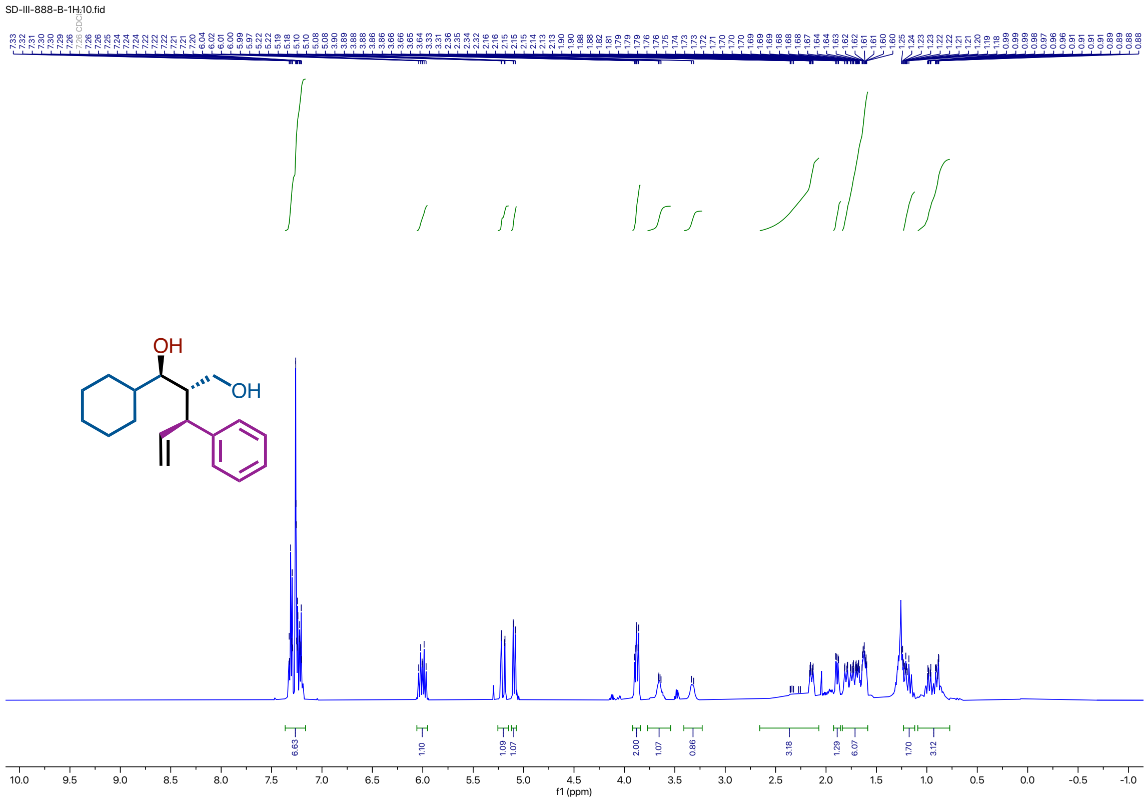

^{13}C NMR (126 MHz, $CDCl_3$) (36)

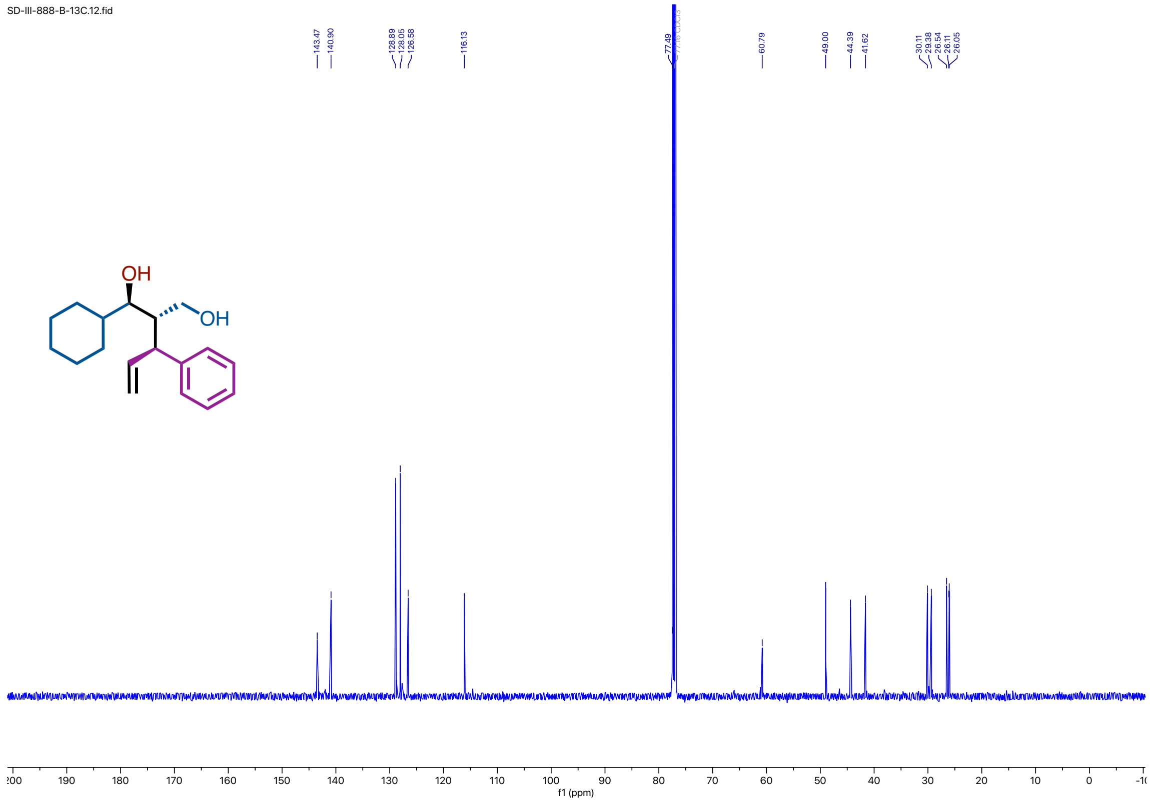

^{1}H NMR (500 MHz, CDCl$_3$) (37)

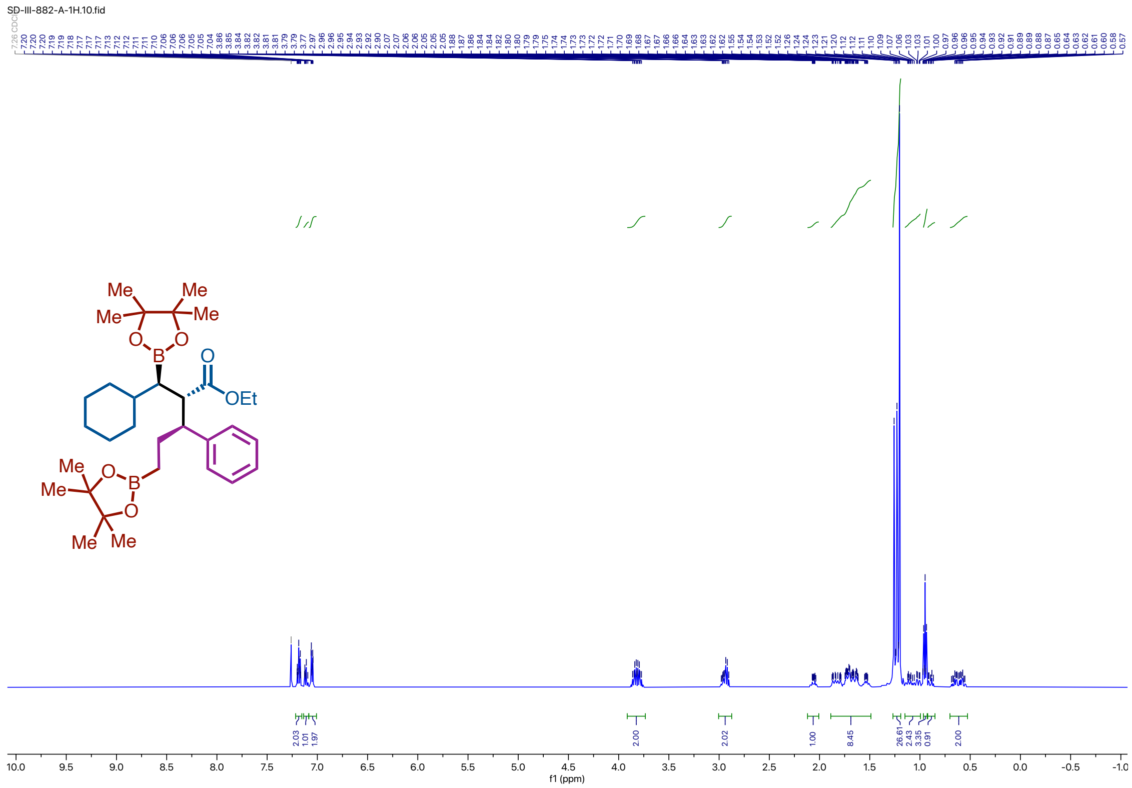

^{13}C NMR (126 MHz, CDCl$_3$) (37)

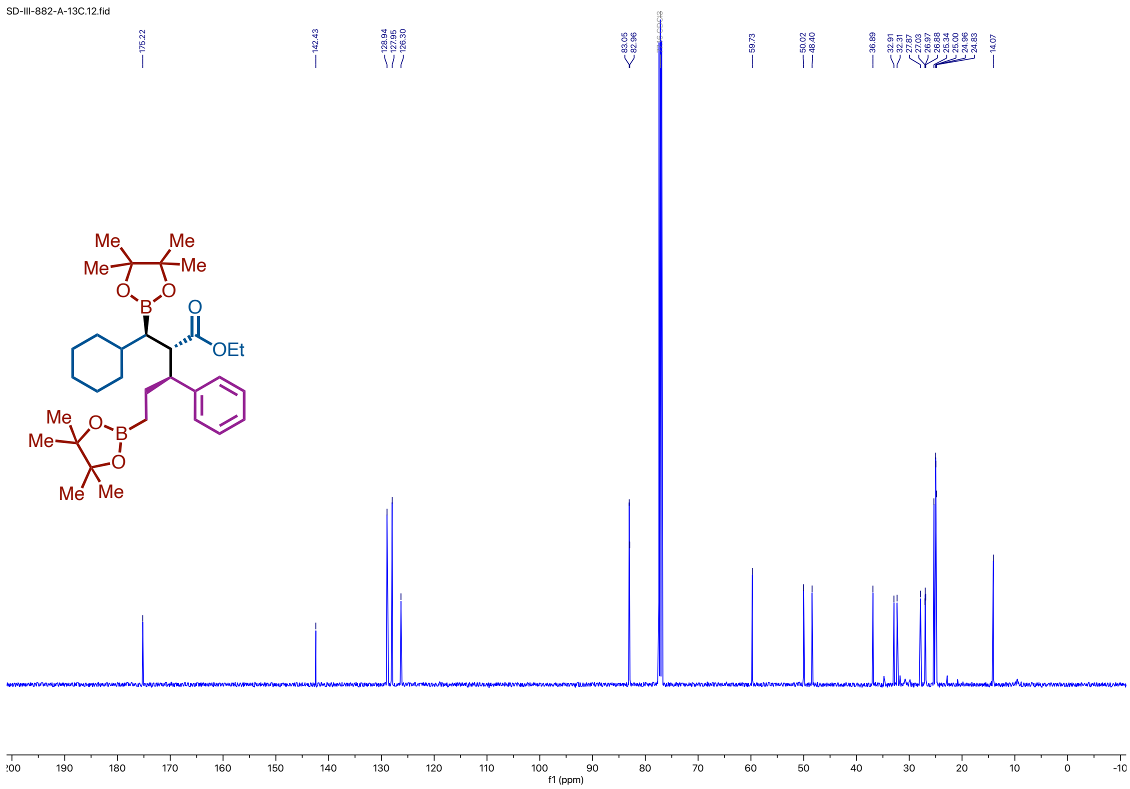

^{1}H NMR (500 MHz, CDCl$_3$)

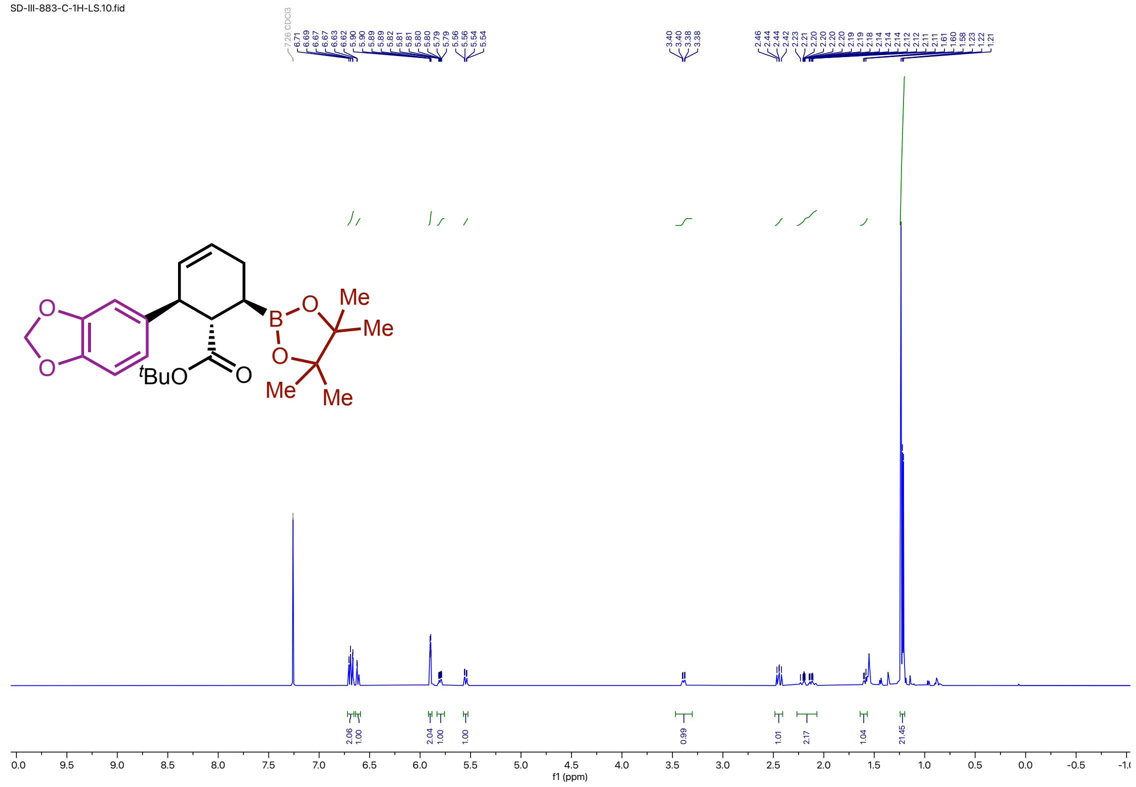

^{13}C NMR (126 MHz, CDCl$_3$)

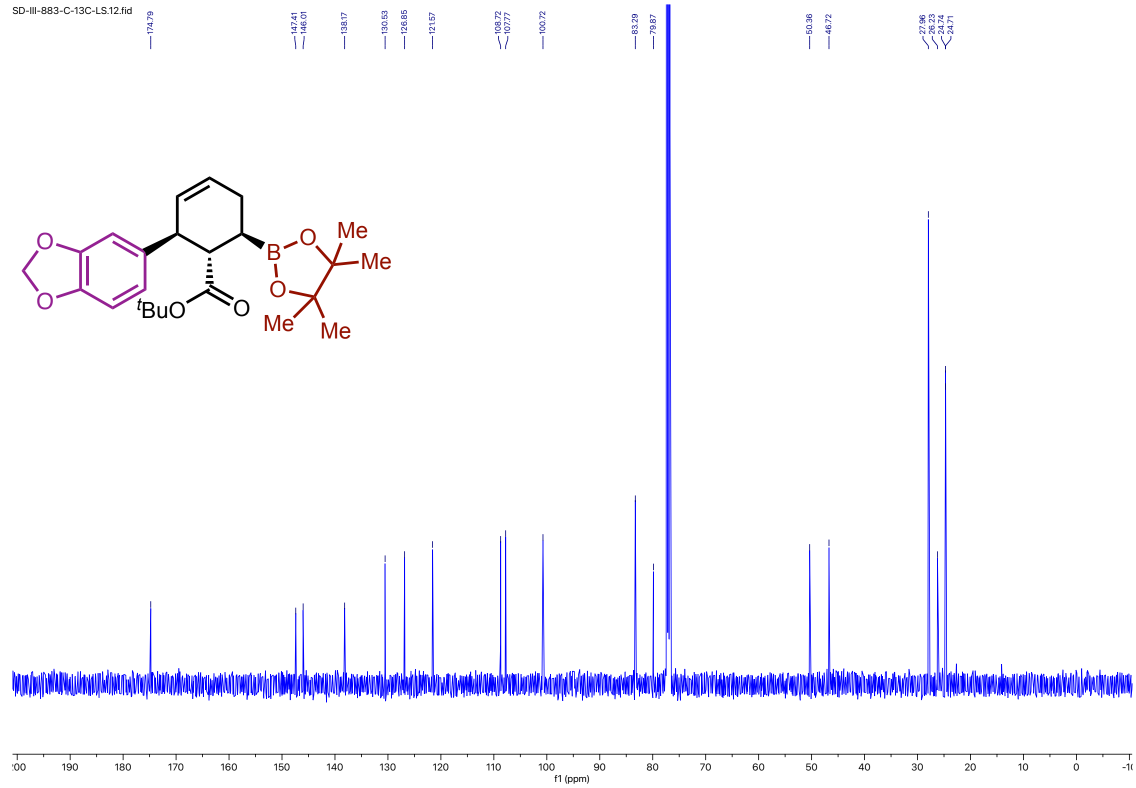

^{1}H NMR (500 MHz, CDCl$_3$)

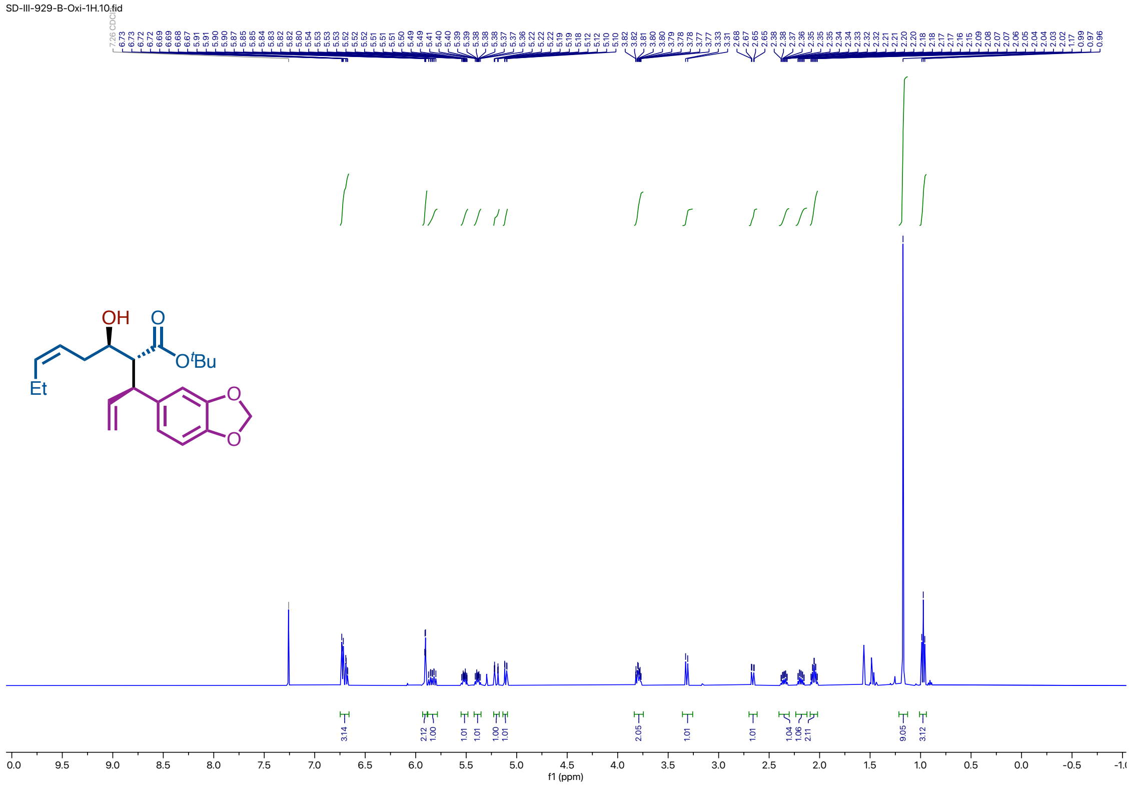

^{13}C NMR (126 MHz, CDCl$_3$)

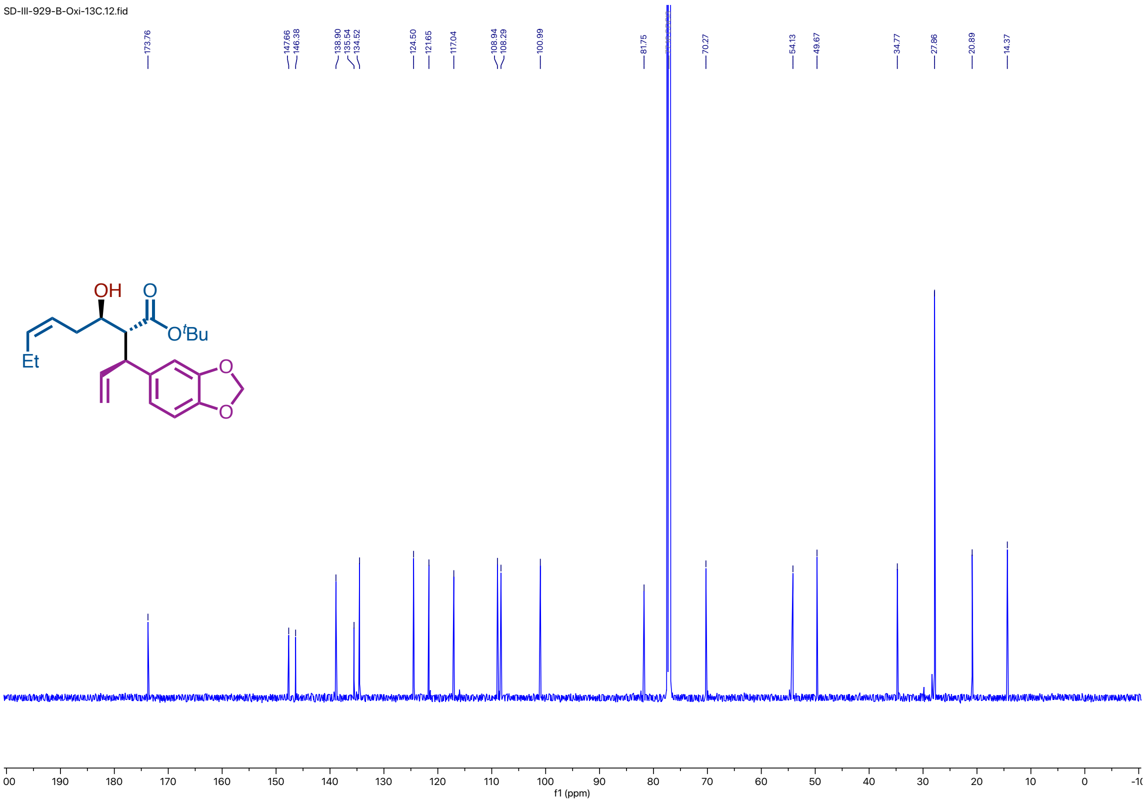

^{1}H NMR (500 MHz, CDCl$_3$)

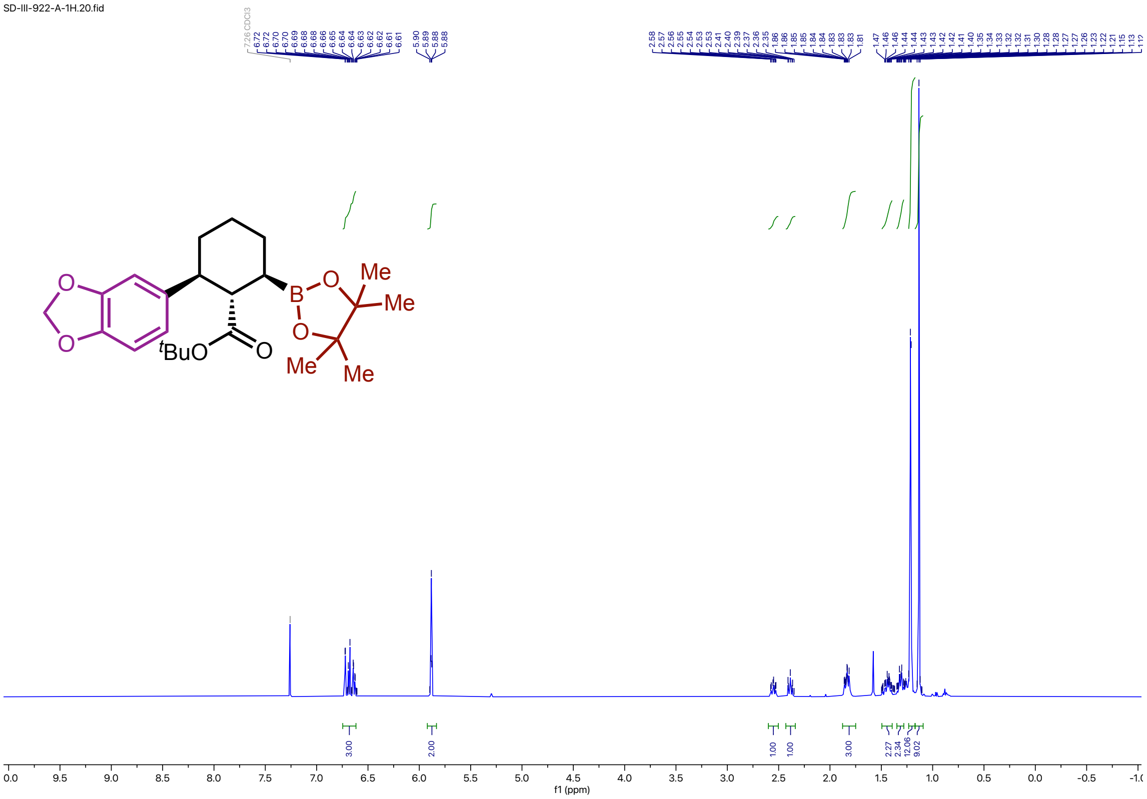

^{13}C NMR (126 MHz, CDCl$_3$)

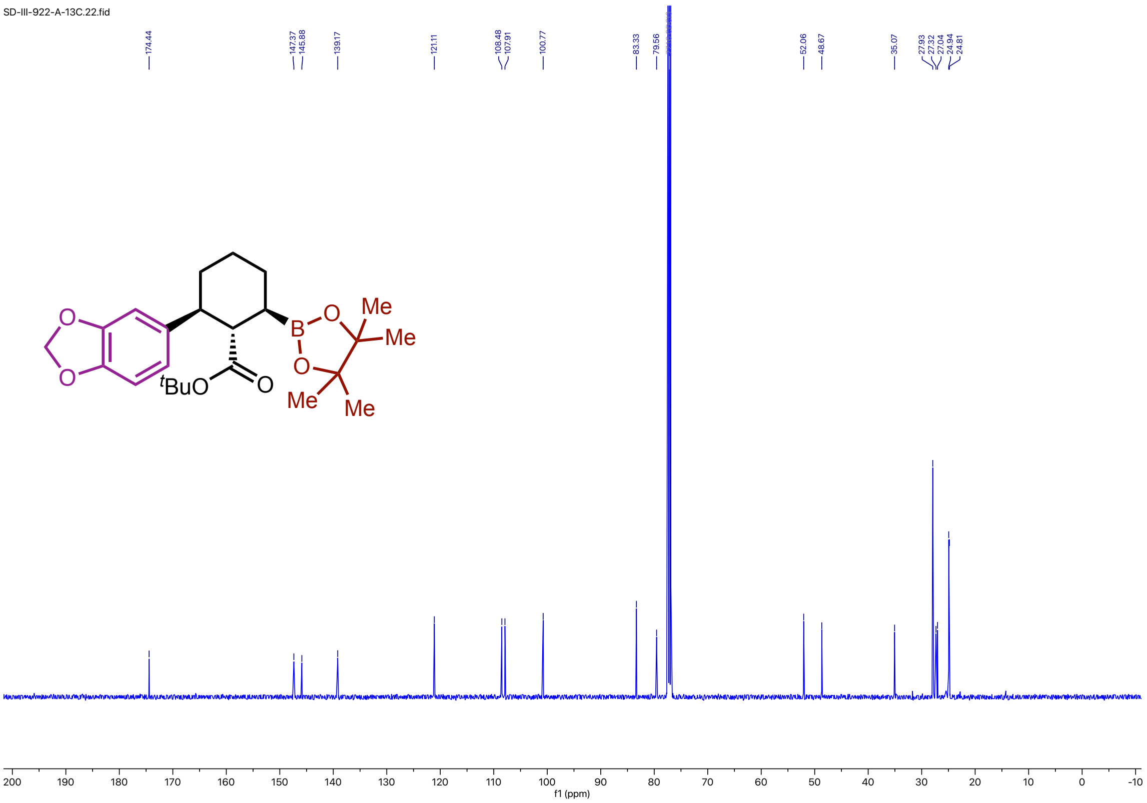

^{1}H NMR (500 MHz, $CDCl_3$)

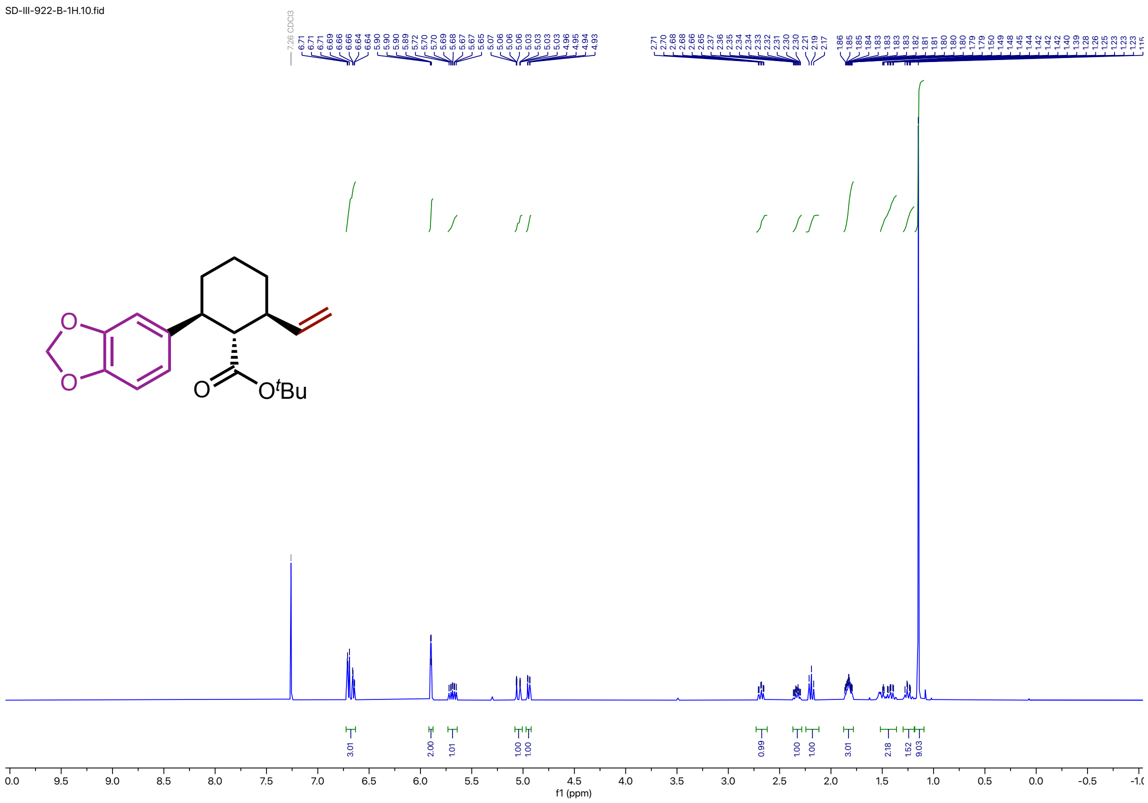

^{13}C NMR (126 MHz, $CDCl_3$)

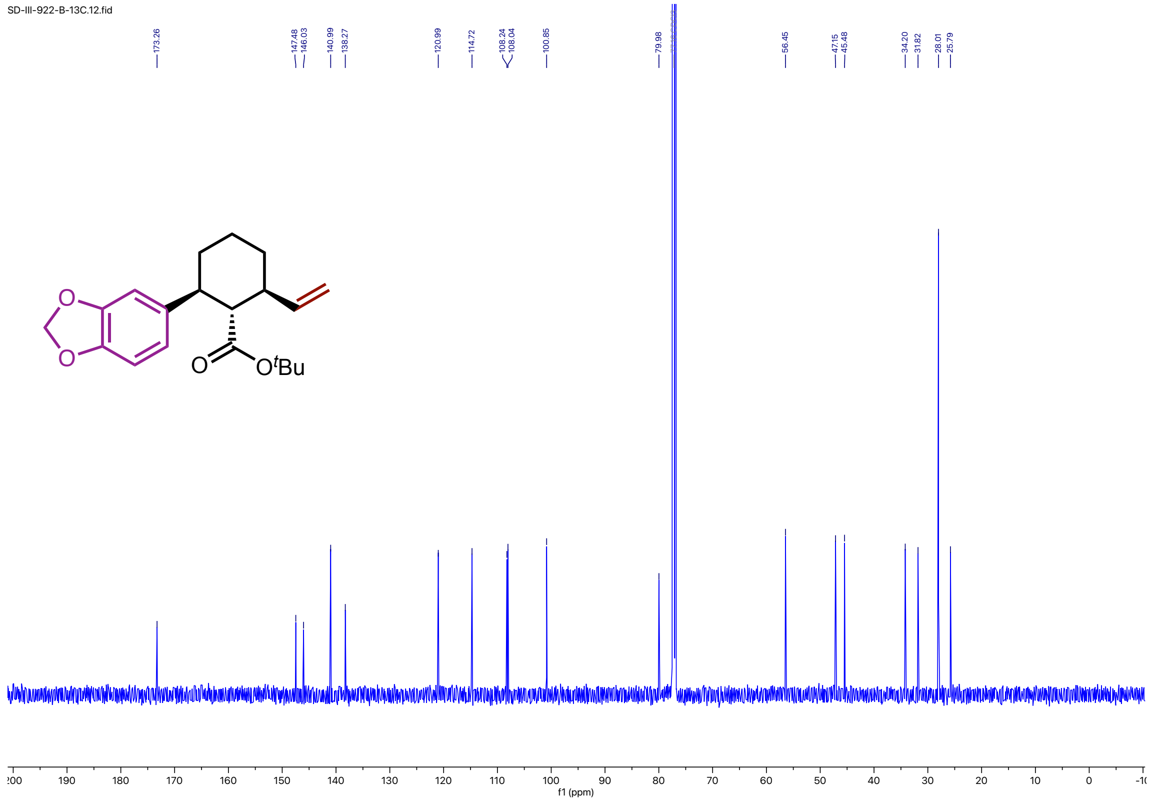

^{1}H NMR (500 MHz, $CDCl_3$)

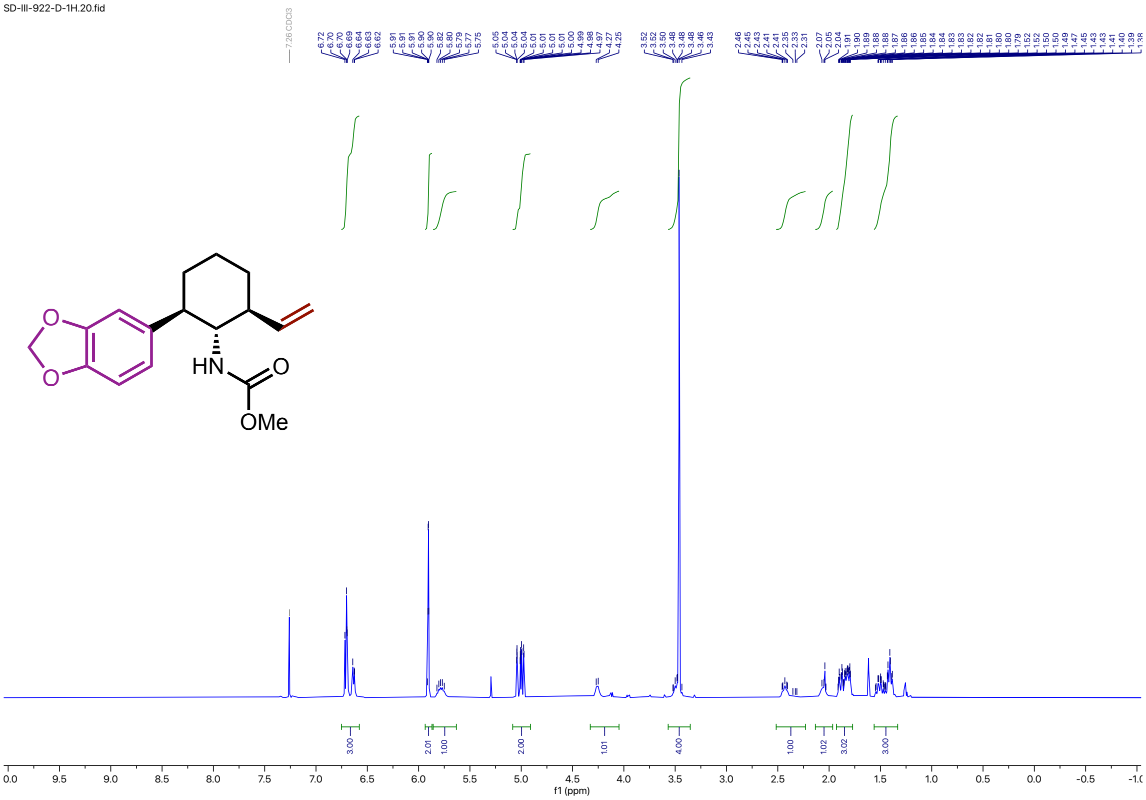

^{13}C NMR (126 MHz, $CDCl_3$)

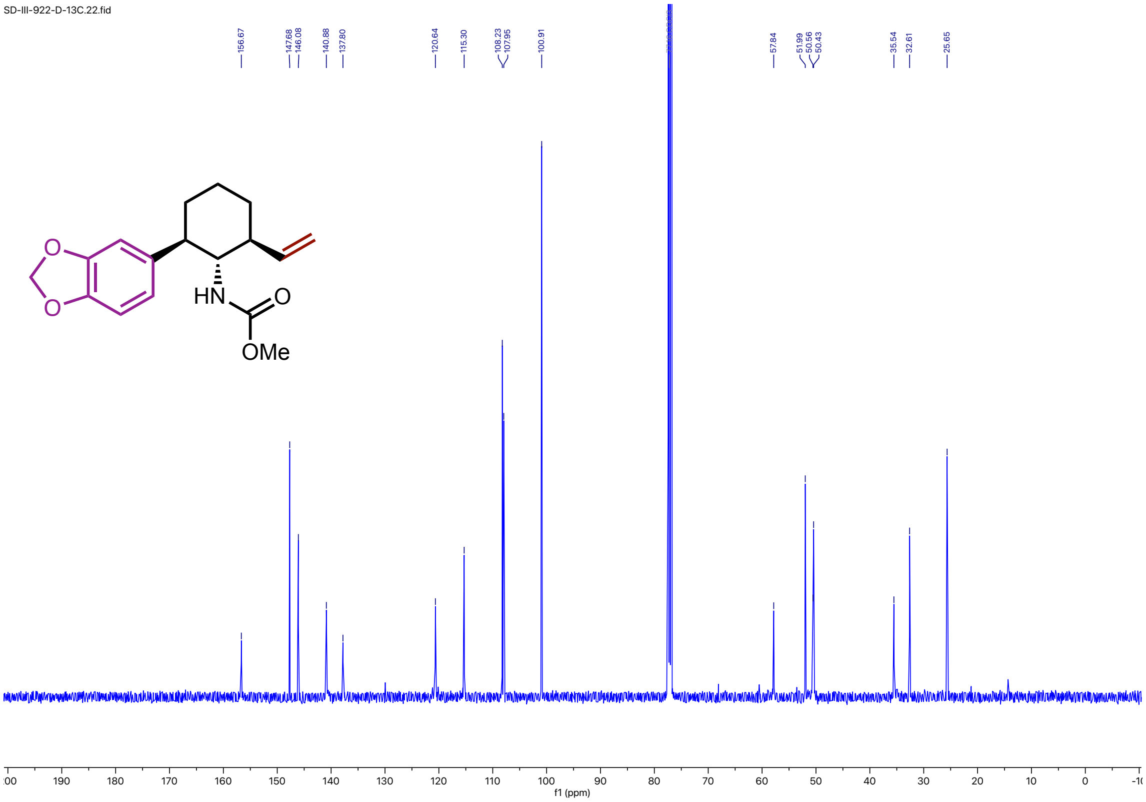

^{1}H NMR (500 MHz, $CDCl_3$)

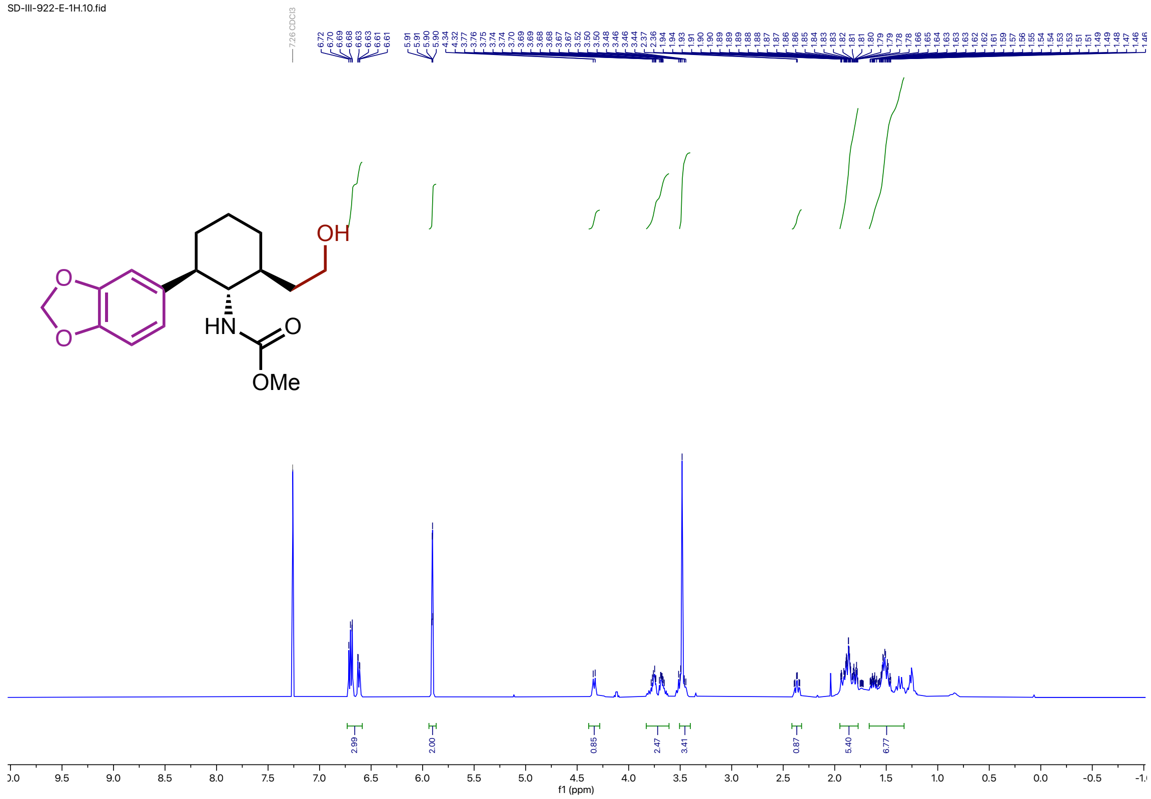

^{13}C NMR (126 MHz, $CDCl_3$)

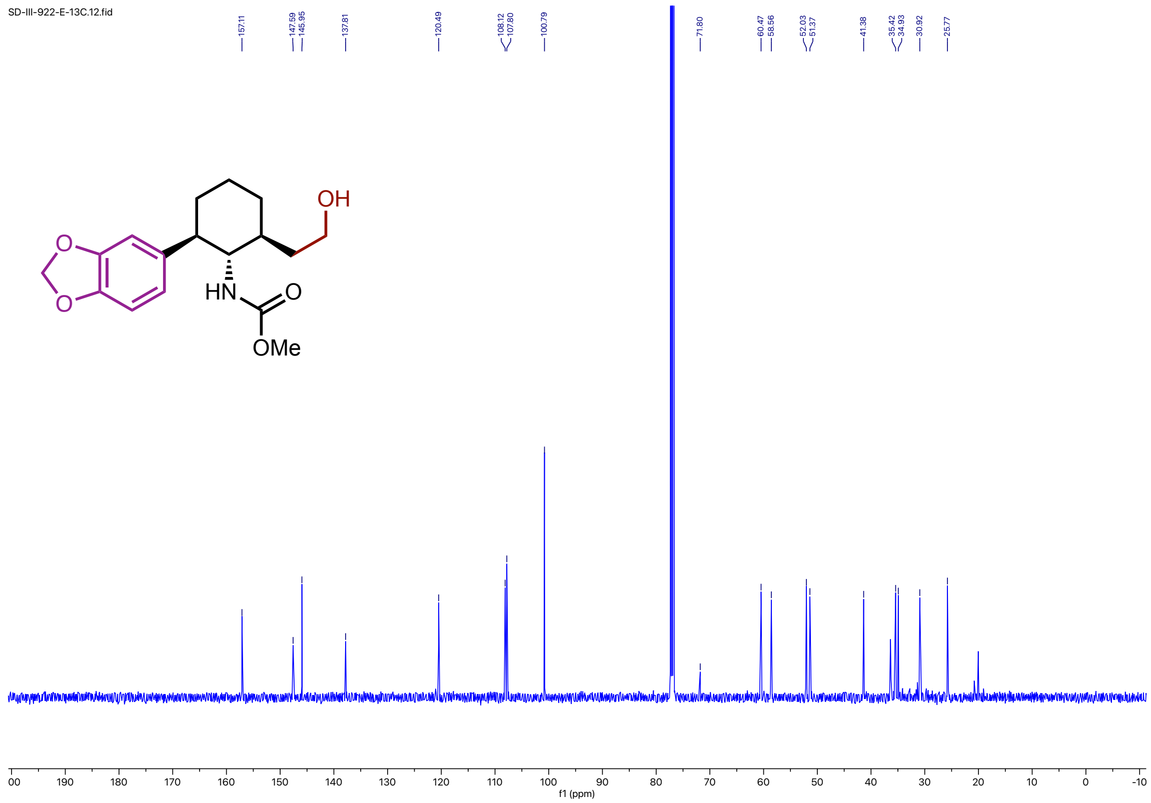

^{1}H NMR (500 MHz, $CDCl_3$)

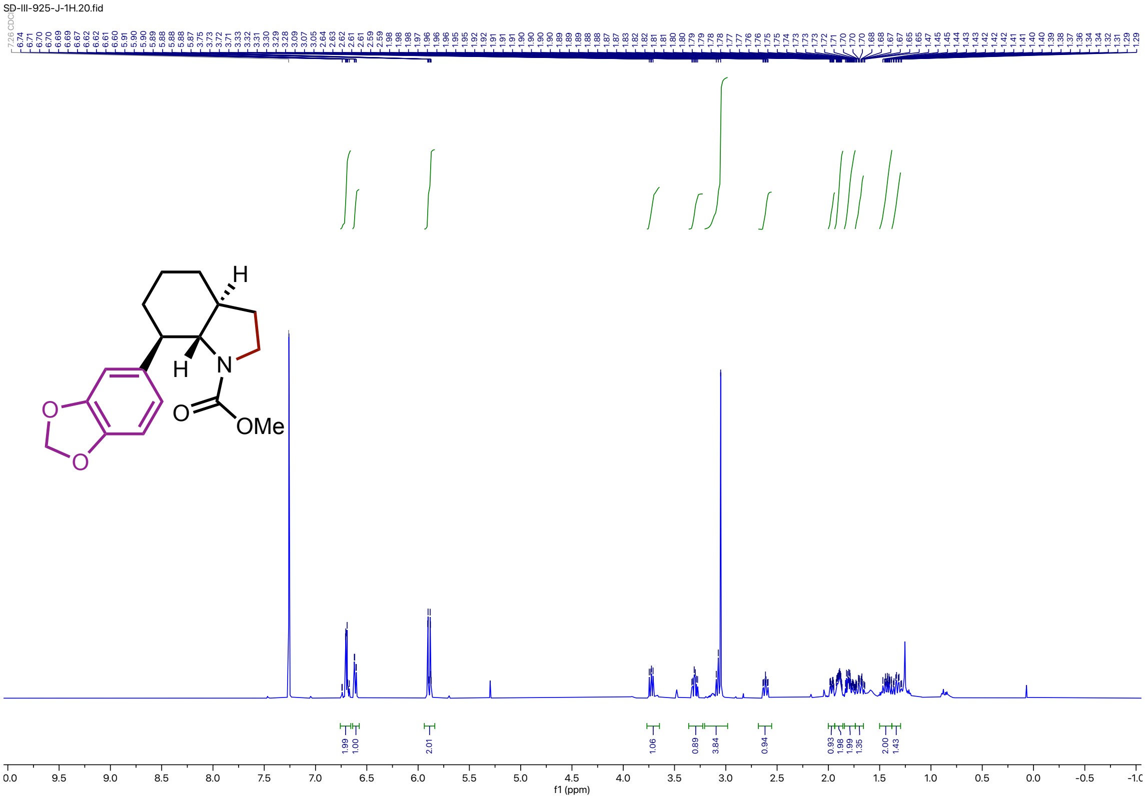

^{13}C NMR (126 MHz, $CDCl_3$)

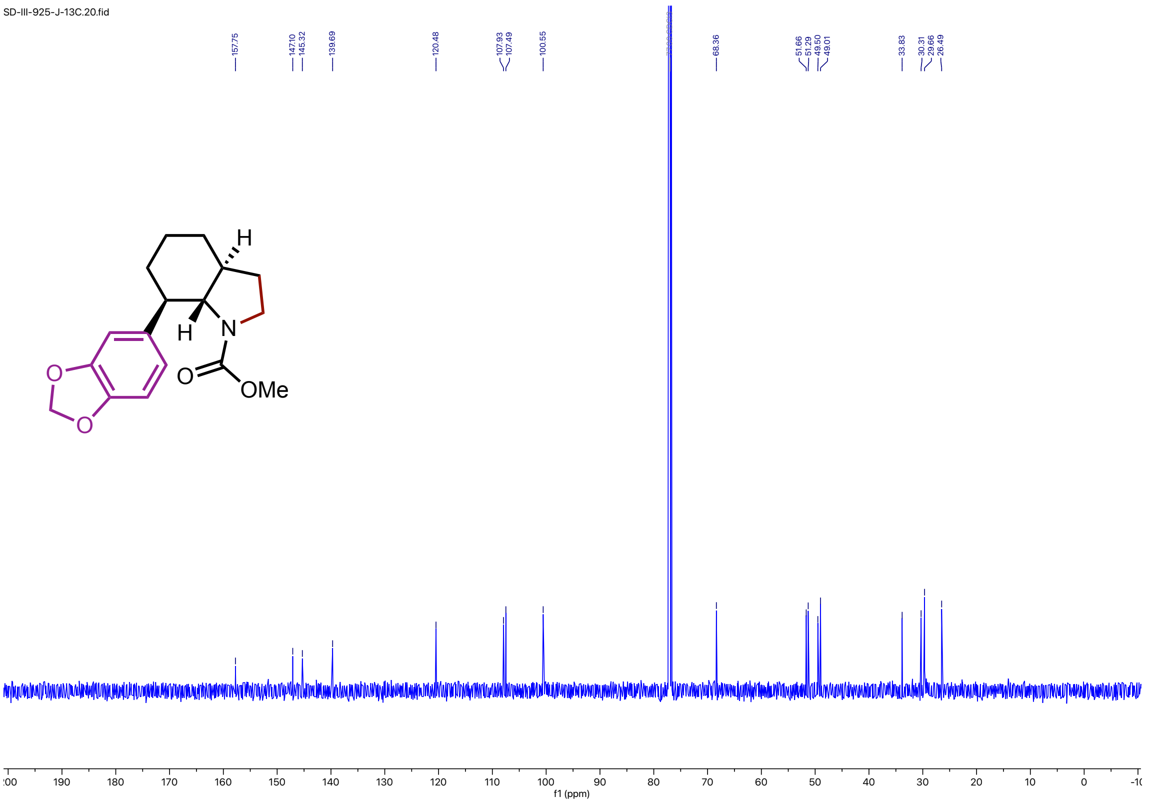

14. HPLC chromatograms:

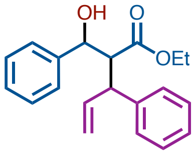

Racemic: (*rac-5*)

```
=====================================================================
Acq. Operator   : SYSTEM                           Seq. Line :   1
Acq. Instrument : 1220 HPLC                         Location : Vial 81
Injection Date  : 10/18/2024 3:27:59 PM                  Inj :   1
                                                  Inj Volume : 10.000 µl
Acq. Method     : C:\CHEM32\2\METHODS\MLC_VARIABLE.M
Last changed    : 10/18/2024 3:49:44 PM by SYSTEM
                  (modified after loading)
Analysis Method : C:\CHEM32\2\METHODS\DEF_LC.M
Last changed    : 1/4/2025 11:25:12 AM by SYSTEM
                  (modified after loading)
Sample Info     : IA3, 95:5 Hex:IPA, 1.0 mL/min, 220 nm


Additional Info : Peak(s) manually integrated
```

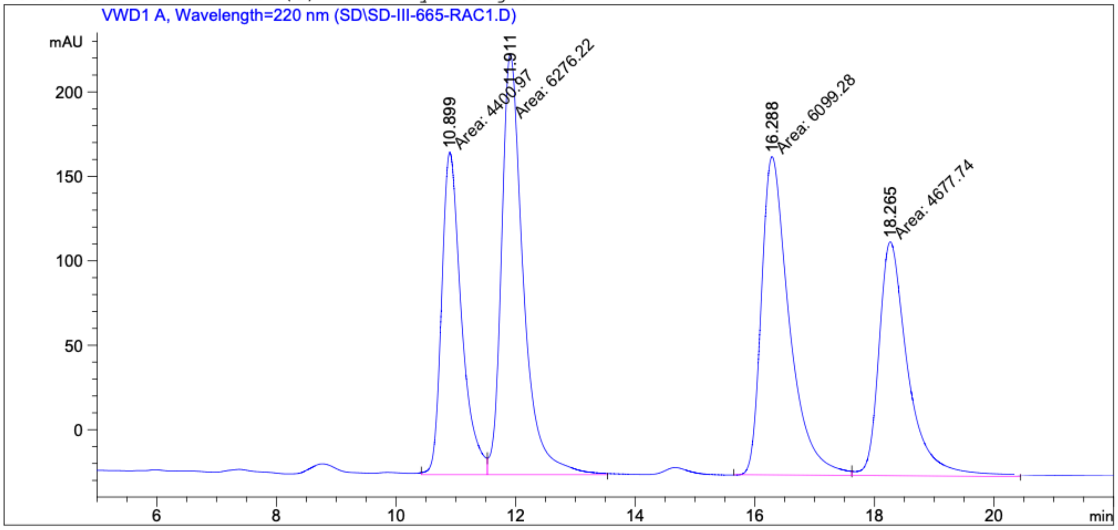


```
=====================================================================
                         Area Percent Report
=====================================================================

Sorted By             :      Signal
Multiplier:                   :      1.0000
Dilution:                     :      1.0000
Do not use Multiplier & Dilution Factor with ISTDs


Signal 1: VWD1 A, Wavelength=220 nm
```

Peak #	RetTime [min]	Type	Width [min]	Area [mAU*s]	Height [mAU]	Area %
1	10.899	MF	0.3844	4400.96680	190.79205	20.5133
2	11.911	FM	0.4200	6276.22119	249.05711	29.2540
3	16.288	MF	0.5397	6099.28418	188.35632	28.4293
4	18.265	FM	0.5632	4677.74219	138.43570	21.8034
Totals :				2.14542e4	766.64119	

Enantioenriched: (*ent-3*)

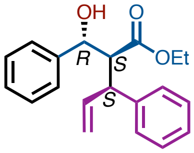


```
====================================================================
Acq. Operator   : SYSTEM                          Seq. Line :   1
Acq. Instrument : 1220 HPLC                        Location : Vial 81
Injection Date  : 1/4/2025 11:42:46 AM                  Inj :   1
                                                Inj Volume : 10.000 µl
Acq. Method     : C:\CHEM32\2\METHODS\MLC_VARIABLE.M
Last changed    : 1/4/2025 11:39:10 AM by SYSTEM
                  (modified after loading)
Analysis Method : C:\CHEM32\2\METHODS\DEF_LC.M
Last changed    : 1/4/2025 11:53:50 AM by SYSTEM
                  (modified after loading)
Sample Info     : IA-3_95:5  Hex:IPA; 1 mL/min, 220 nm


Additional Info : Peak(s) manually integrated
```

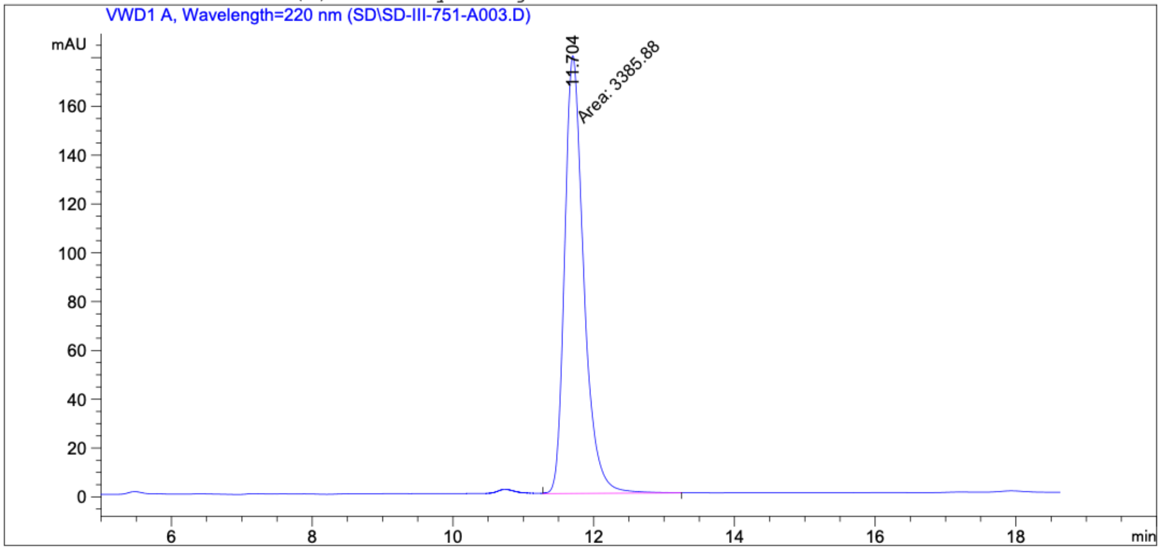


```
====================================================================
                         Area Percent Report
====================================================================

Sorted By             :      Signal
Multiplier:                   :      1.0000
Dilution:                     :      1.0000
Do not use Multiplier & Dilution Factor with ISTDs


Signal 1: VWD1 A, Wavelength=220 nm

Peak RetTime Type  Width     Area      Height     Area
  #   [min]        [min]   [mAU*s]     [mAU]        %
----|-------|----|-------|----------|----------|--------|
   1  11.704 MM    0.3148 3385.88306  179.25864 100.0000

Totals :                  3385.88306  179.25864
```

Enantioenriched: (*ent-5*)

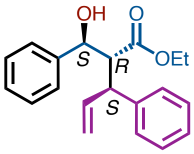


```
==================================================================
Acq. Operator   : SYSTEM                          Seq. Line :   1
Acq. Instrument : 1220 HPLC                        Location : Vial 81
Injection Date  : 12/29/2024 2:08:15 PM                 Inj :   1
                                                 Inj Volume : 10.000 µl
Acq. Method     : C:\CHEM32\2\METHODS\MLC_VARIABLE.M
Last changed    : 12/29/2024 2:04:28 PM by SYSTEM
Analysis Method : C:\CHEM32\2\METHODS\DEF_LC.M
Last changed    : 1/4/2025 11:25:12 AM by SYSTEM
                  (modified after loading)
Sample Info     : IA-3_95:5  Hex:IPA; 1 mL/min, 220 nm


Additional Info : Peak(s) manually integrated
```

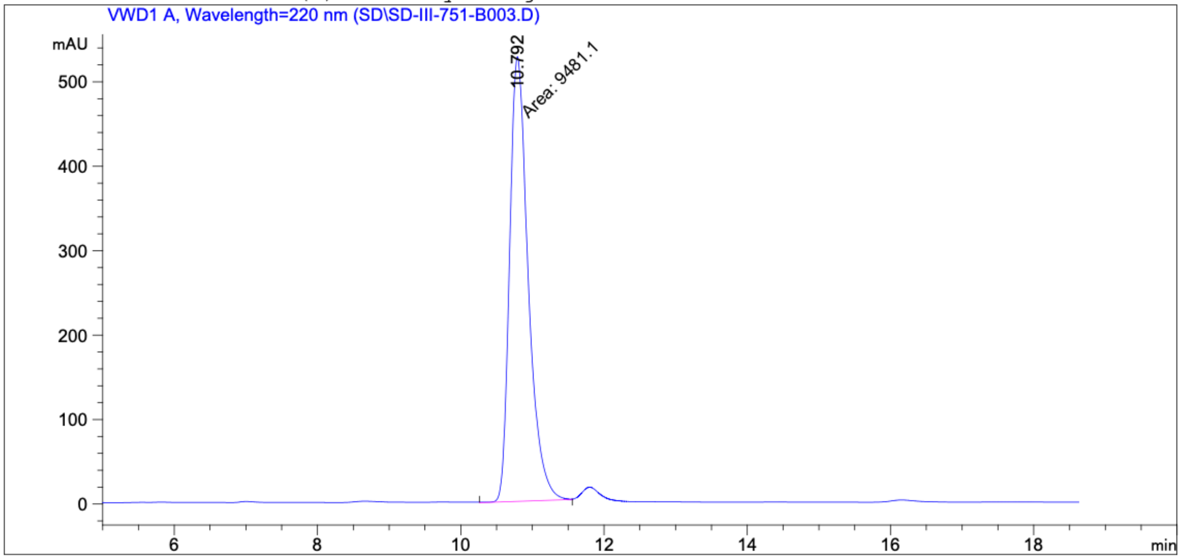


```
==================================================================
                         Area Percent Report
==================================================================

Sorted By             :      Signal
Multiplier:                   :      1.0000
Dilution:                     :      1.0000
Do not use Multiplier & Dilution Factor with ISTDs


Signal 1: VWD1 A, Wavelength=220 nm

Peak RetTime Type  Width     Area      Height     Area
  #   [min]        [min]   [mAU*s]     [mAU]        %
----|-------|----|-------|----------|----------|--------|
   1  10.792 MM    0.3002 9481.10449  526.42487 100.0000

Totals :                  9481.10449  526.42487
```

Enantioenriched: (*ent-29*)

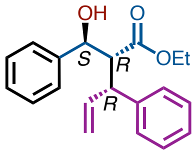


```
=====================================================================
Acq. Operator   : SYSTEM                          Seq. Line :    1
Acq. Instrument : 1220 HPLC                        Location : Vial 81
Injection Date  : 12/31/2024 6:14:02 PM                 Inj :    1
                                                 Inj Volume : 10.000 µl
Acq. Method     : C:\CHEM32\2\METHODS\MLC_VARIABLE.M
Last changed    : 12/31/2024 6:35:30 PM by SYSTEM
                  (modified after loading)
Analysis Method : C:\CHEM32\2\METHODS\DEF_LC.M
Last changed    : 1/4/2025 11:25:12 AM by SYSTEM
                  (modified after loading)
Sample Info     : IA-3_95:5  Hex:IPA; 1 mL/min, 220 nm


Additional Info : Peak(s) manually integrated
```

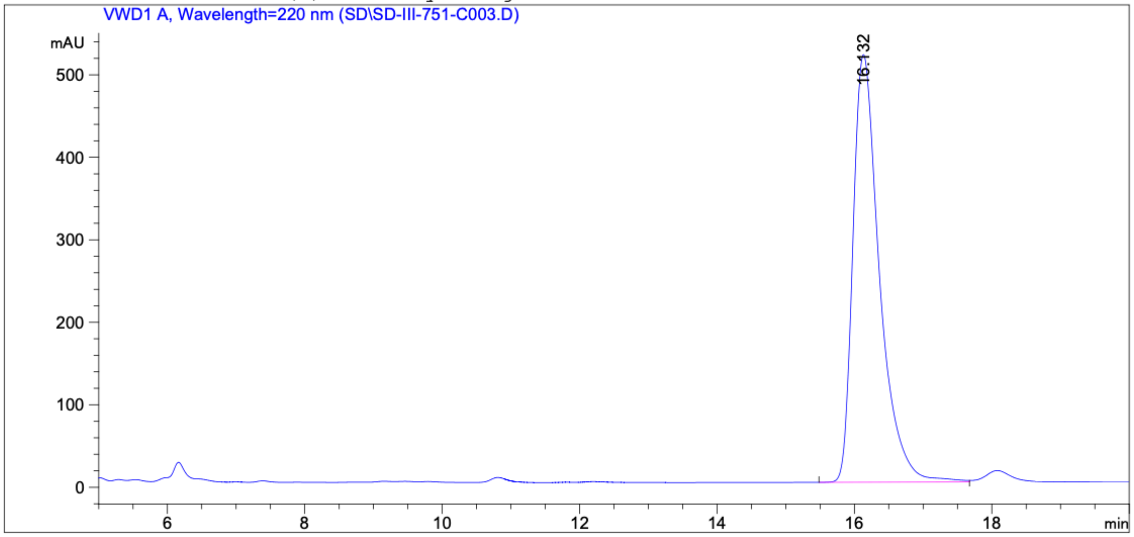


```
=====================================================================
                         Area Percent Report
=====================================================================

Sorted By             :      Signal
Multiplier:                  :      1.0000
Dilution:                    :      1.0000
Do not use Multiplier & Dilution Factor with ISTDs


Signal 1: VWD1 A, Wavelength=220 nm

Peak RetTime Type  Width     Area      Height     Area
  #   [min]        [min]   [mAU*s]     [mAU]        %
----|-------|----|-------|----------|----------|--------|
   1  16.132 BV    0.4073 1.39171e4   518.30762 100.0000

Totals :                  1.39171e4   518.30762
```

Enantioenriched: (*ent-4*)

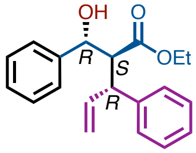


```
=====================================================================
Acq. Operator   : SYSTEM                          Seq. Line :   1
Acq. Instrument : 1220 HPLC                        Location : Vial 81
Injection Date  : 12/30/2024 5:54:32 PM                 Inj :   1
                                                 Inj Volume : 10.000 µl
Acq. Method     : C:\CHEM32\2\METHODS\MLC_VARIABLE.M
Last changed    : 12/30/2024 5:50:44 PM by SYSTEM
Analysis Method : C:\CHEM32\2\METHODS\DEF_LC.M
Last changed    : 12/11/2024 1:37:10 PM by SYSTEM
Sample Info     : IA-3_95:5  Hex:IPA; 1 mL/min, 220 nm


Additional Info : Peak(s) manually integrated
```

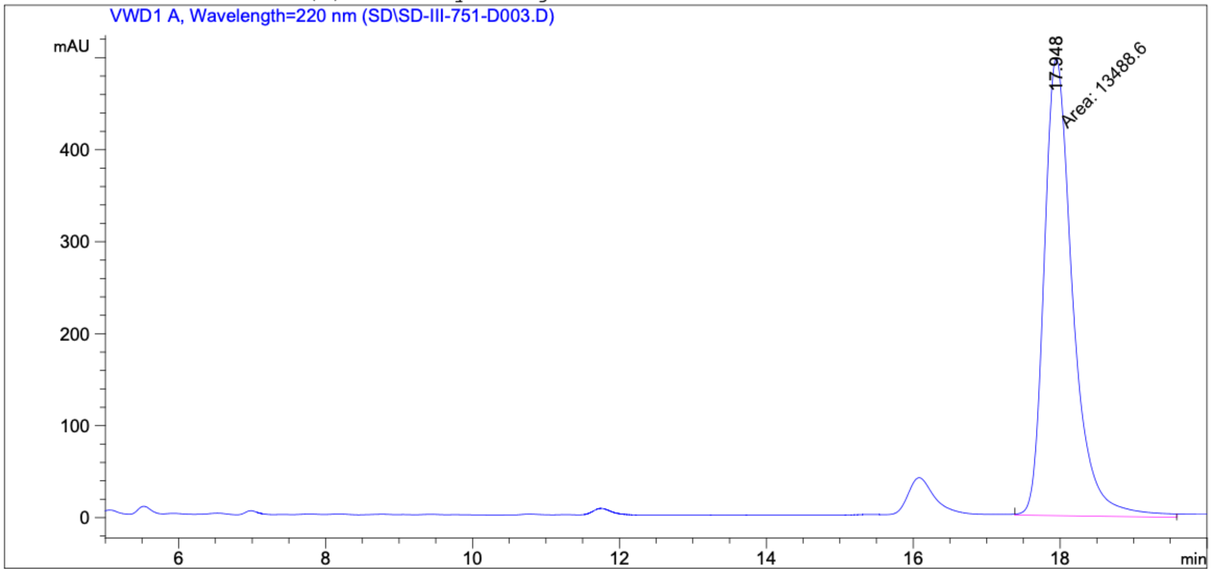


```
=====================================================================
                         Area Percent Report
=====================================================================

Sorted By             :      Signal
Multiplier:                    :      1.0000
Dilution:                      :      1.0000
Do not use Multiplier & Dilution Factor with ISTDs


Signal 1: VWD1 A, Wavelength=220 nm

Peak RetTime Type  Width     Area      Height     Area
  #   [min]        [min]   [mAU*s]     [mAU]        %
----|-------|----|-------|----------|----------|--------|
   1  17.948 MM    0.4522 1.34886e4  497.15436 100.0000

Totals :                  1.34886e4  497.15436
```

Racemic: (*rac-6*)

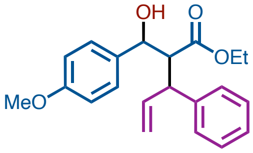


```
=====================================================================
Acq. Operator   : SYSTEM                          Seq. Line :    1
Acq. Instrument : 1220 HPLC                        Location : Vial 81
Injection Date  : 11/16/2024 8:43:20 AM                 Inj :    1
                                                 Inj Volume : 10.000 µl
Acq. Method     : C:\CHEM32\2\METHODS\MLC_VARIABLE.M
Last changed    : 11/16/2024 8:39:47 AM by SYSTEM
                  (modified after loading)
Analysis Method : C:\CHEM32\2\METHODS\DEF_LC.M
Last changed    : 4/24/2025 5:19:24 PM by SYSTEM
                  (modified after loading)
Sample Info     : IA-3, 95:5 HEX/IPA, 1ml/min, 220nm,


Additional Info : Peak(s) manually integrated
```

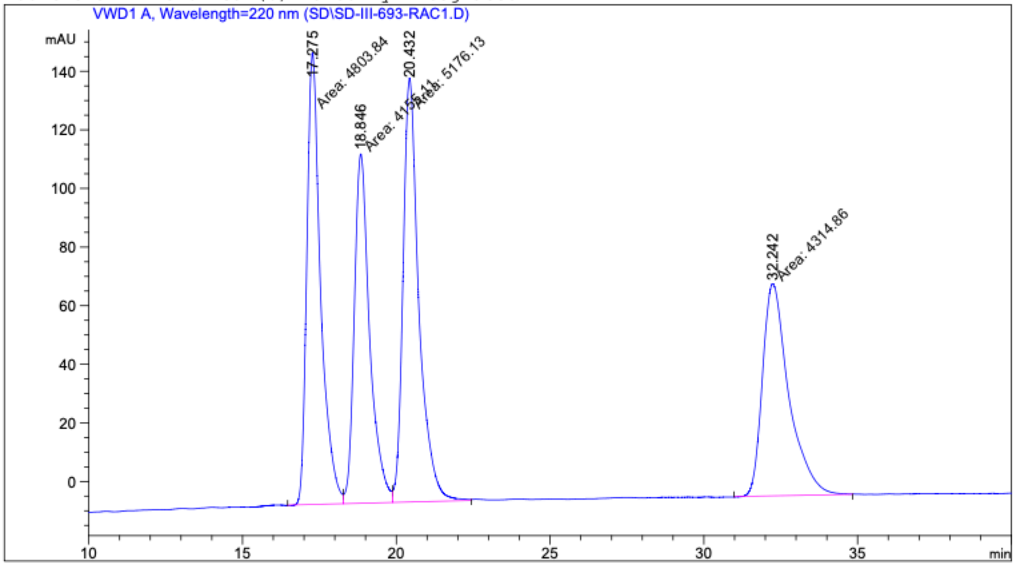


```
=====================================================================
                         Area Percent Report
=====================================================================

Sorted By             :      Signal
Multiplier:                  :      1.0000
Dilution:                    :      1.0000
Do not use Multiplier & Dilution Factor with ISTDs


Signal 1: VWD1 A, Wavelength=220 nm
```

Peak #	RetTime [min]	Type	Width [min]	Area [mAU*s]	Height [mAU]	Area %
1	17.275	MF	0.5198	4803.83838	154.01826	26.0372
2	18.846	MF	0.5811	4155.10840	119.16718	22.5210
3	20.432	FM	0.5969	5176.13184	144.52867	28.0550
4	32.242	MM	0.9941	4314.85791	72.34244	23.3868

Totals : 1.84499e4 490.05655

Enantioenriched: (*ent-6*)

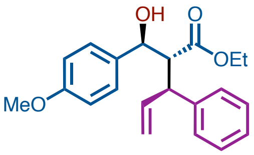


```
=====================================================================
Acq. Operator   : SYSTEM                          Seq. Line :    1
Acq. Instrument : 1220 HPLC                        Location : Vial 71
Injection Date  : 11/16/2024 9:27:55 AM                 Inj :    1
                                                 Inj Volume : 10.000 µl
Acq. Method     : C:\CHEM32\2\METHODS\MLC_VARIABLE.M
Last changed    : 11/16/2024 9:24:07 AM by SYSTEM
Analysis Method : C:\CHEM32\2\METHODS\DEF_LC.M
Last changed    : 4/25/2025 2:15:03 PM by SYSTEM
                  (modified after loading)
Sample Info     : IA-3, 95:5 HEX/IPA, 1ml/min, 220nm,

Additional Info : Peak(s) manually integrated
```

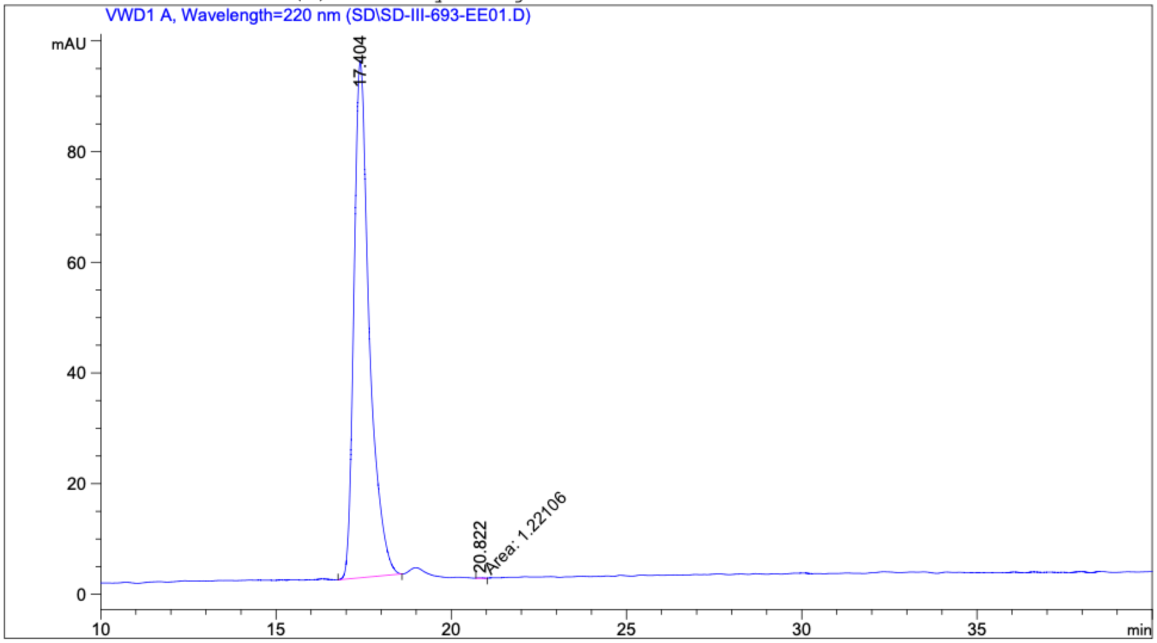


```
=====================================================================
                         Area Percent Report
=====================================================================

Sorted By             :      Signal
Multiplier:                   :      1.0000
Dilution:                     :      1.0000
Do not use Multiplier & Dilution Factor with ISTDs


Signal 1: VWD1 A, Wavelength=220 nm
```

Peak #	RetTime [min]	Type	Width [min]	Area [mAU*s]	Height [mAU]	Area %
1	17.404	BB	0.4548	2858.41626	93.47188	99.9573
2	20.822	MM	0.2294	1.22106	8.87324e-2	0.0427

Totals : 2859.63732 93.56061

Racemic: (*rac-7*)

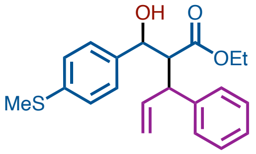


```
=====================================================================
Acq. Operator   : SYSTEM                            Seq. Line :   2
Acq. Instrument : 1220 HPLC                          Location : Vial 81
Injection Date  : 12/7/2024 7:18:43 PM                    Inj :   1
                                                   Inj Volume : 10.000 µl
Acq. Method     : C:\CHEM32\2\METHODS\MLC_VARIABLE.M
Last changed    : 12/7/2024 6:39:12 PM by SYSTEM
                  (modified after loading)
Analysis Method : C:\CHEM32\2\METHODS\DEF_LC.M
Last changed    : 4/25/2025 2:19:58 PM by SYSTEM
                  (modified after loading)
Sample Info     : C1; 95:5 Hex:IPA; 0.5 mL/min, 220 nm

Additional Info : Peak(s) manually integrated
```

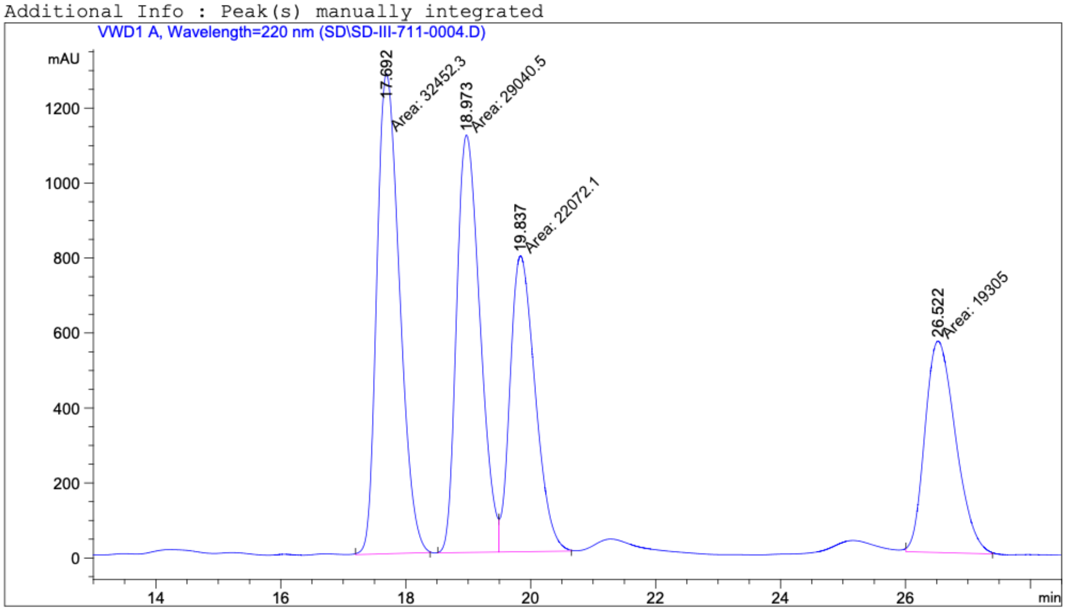


```
=====================================================================
                         Area Percent Report
=====================================================================

Sorted By             :      Signal
Multiplier:                   :      1.0000
Dilution:                     :      1.0000
Do not use Multiplier & Dilution Factor with ISTDs


Signal 1: VWD1 A, Wavelength=220 nm
```

Peak #	RetTime [min]	Type	Width [min]	Area [mAU*s]	Height [mAU]	Area %
1	17.692	MM	0.4224	3.24523e4	1280.55286	31.5469
2	18.973	MF	0.4347	2.90405e4	1113.35706	28.2303
3	19.837	FM	0.4661	2.20721e4	789.25677	21.4564
4	26.522	MM	0.5706	1.93050e4	563.89734	18.7664
Totals :				1.02870e5	3747.06403	

Enantioenriched: (*ent*-7)

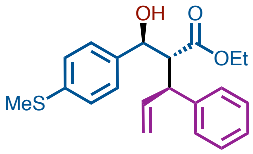


```
=====================================================================
Acq. Operator   : SYSTEM                          Seq. Line :    1
Acq. Instrument : 1220 HPLC                        Location : Vial 71
Injection Date  : 12/7/2024 6:42:49 PM                  Inj :    1
                                                Inj Volume : 10.000 µl
Acq. Method     : C:\CHEM32\2\METHODS\MLC_VARIABLE.M
Last changed    : 12/7/2024 6:39:12 PM by SYSTEM
                  (modified after loading)
Analysis Method : C:\CHEM32\2\METHODS\DEF_LC.M
Last changed    : 4/25/2025 2:19:58 PM by SYSTEM
                  (modified after loading)
Sample Info     : C1; 95:5 Hex:IPA; 0.5 mL/min, 220 nm


Additional Info : Peak(s) manually integrated
```

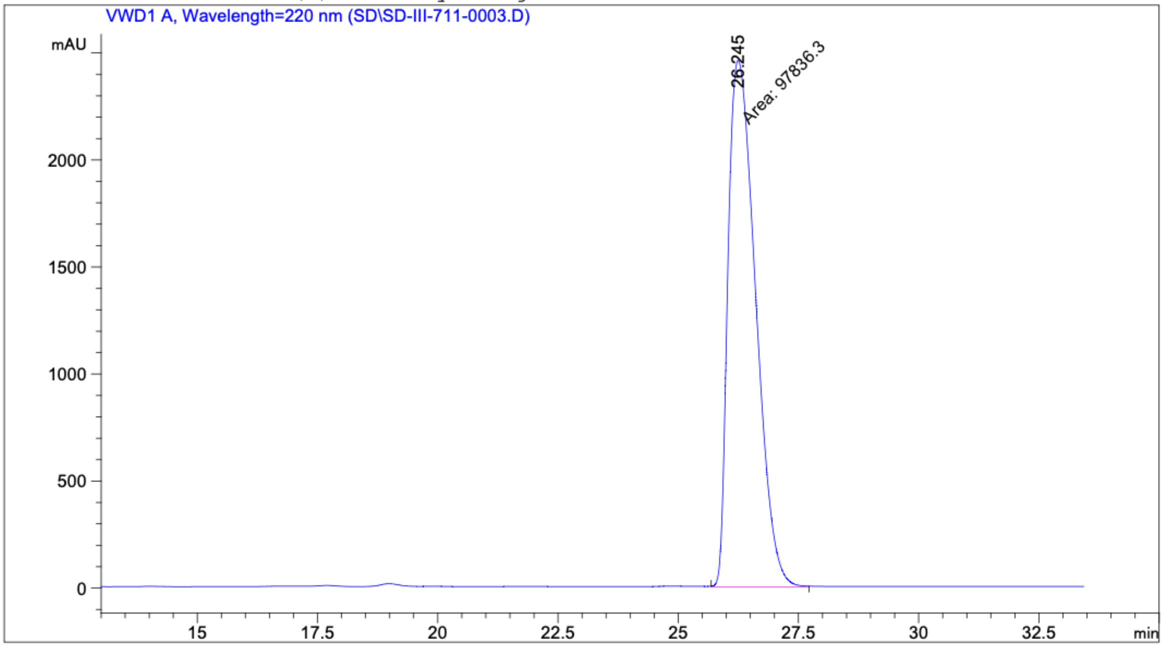


```
=====================================================================
                         Area Percent Report
=====================================================================

Sorted By             :      Signal
Multiplier:                   :      1.0000
Dilution:                     :      1.0000
Do not use Multiplier & Dilution Factor with ISTDs


Signal 1: VWD1 A, Wavelength=220 nm

Peak RetTime Type  Width     Area      Height     Area
  #   [min]        [min]   [mAU*s]     [mAU]        %
----|-------|----|-------|----------|----------|--------|
   1  26.245 MM    0.6631 9.78363e4  2459.05249 100.0000

Totals :                  9.78363e4  2459.05249
```

Racemic: (*rac-8*)

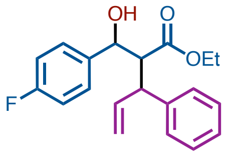


```
====================================================================
Acq. Operator   : SYSTEM                          Seq. Line :   1
Acq. Instrument : 1220 HPLC                        Location : Vial 81
Injection Date  : 12/9/2024 11:21:55 AM                 Inj :   1
                                                 Inj Volume : 10.000 µl
Acq. Method     : C:\CHEM32\2\METHODS\MLC_VARIABLE.M
Last changed    : 12/9/2024 11:16:07 AM by SYSTEM
Analysis Method : C:\CHEM32\2\METHODS\DEF_LC.M
Last changed    : 4/25/2025 2:25:03 PM by SYSTEM
                  (modified after loading)
Sample Info     : C2- 95:5 Hex:IPA; 1 mL/min, 220 nm


Additional Info : Peak(s) manually integrated
```

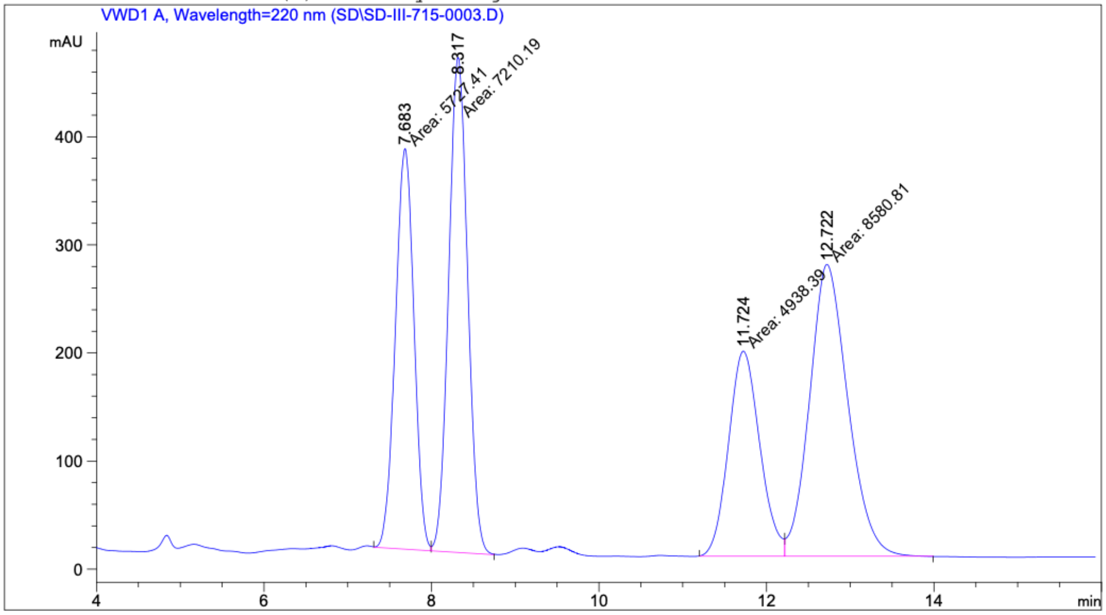


```
====================================================================
                         Area Percent Report
====================================================================

Sorted By             :      Signal
Multiplier:                    :      1.0000
Dilution:                      :      1.0000
Do not use Multiplier & Dilution Factor with ISTDs


Signal 1: VWD1 A, Wavelength=220 nm
```

Peak #	RetTime [min]	Type	Width [min]	Area [mAU*s]	Height [mAU]	Area %
1	7.683	MF	0.2577	5727.40869	370.40060	21.6482
2	8.317	FM	0.2624	7210.18604	457.94647	27.2527
3	11.724	MF	0.4338	4938.38672	189.72891	18.6659
4	12.722	FM	0.5298	8580.80859	269.91348	32.4333
Totals :				2.64568e4	1287.98947	

Enantioenriched: (*ent-8*)

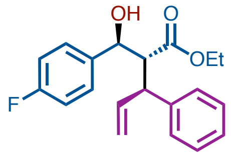


```
=====================================================================
Acq. Operator   : SYSTEM                          Seq. Line :    2
Acq. Instrument : 1220 HPLC                        Location : Vial 71
Injection Date  : 12/9/2024 11:42:49 AM                 Inj :    1
                                                 Inj Volume : 10.000 µl
Acq. Method     : C:\CHEM32\2\METHODS\MLC_VARIABLE.M
Last changed    : 12/9/2024 11:16:07 AM by SYSTEM
Analysis Method : C:\CHEM32\2\METHODS\DEF_LC.M
Last changed    : 4/25/2025 2:22:57 PM by SYSTEM
                  (modified after loading)
Sample Info     : C2; 95:5 Hex:IPA; 1 mL/min, 220 nm

Additional Info : Peak(s) manually integrated
```

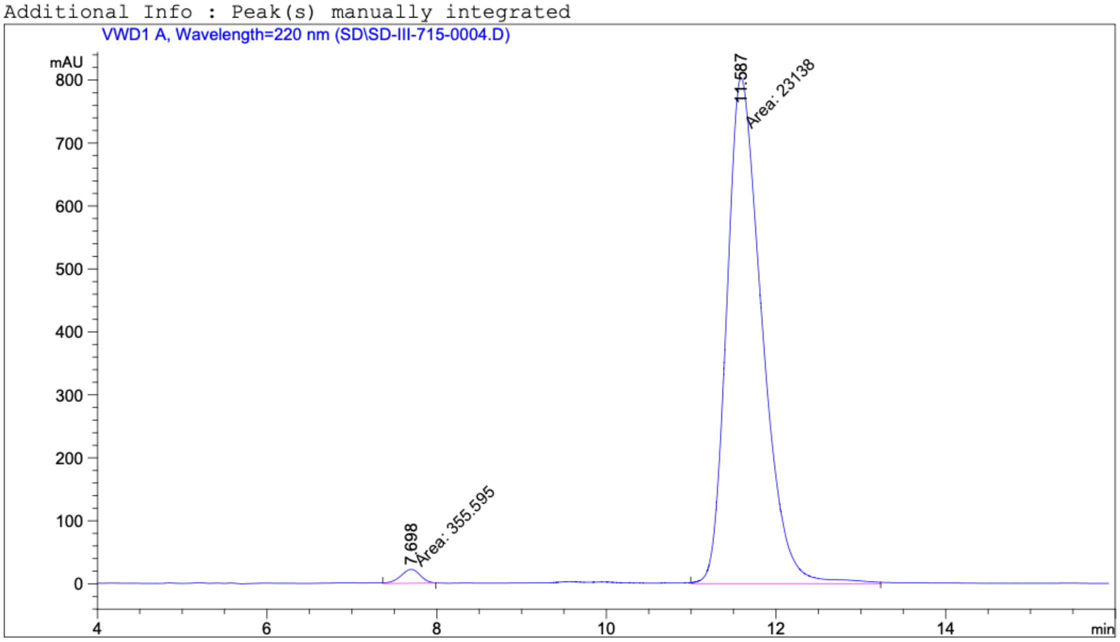


```
=====================================================================
                         Area Percent Report
=====================================================================

Sorted By             :      Signal
Multiplier:                  :      1.0000
Dilution:                    :      1.0000
Do not use Multiplier & Dilution Factor with ISTDs


Signal 1: VWD1 A, Wavelength=220 nm

Peak RetTime Type  Width     Area      Height     Area
  #   [min]        [min]   [mAU*s]     [mAU]       %
----|-------|----|-------|----------|----------|--------|
   1   7.698 MM    0.2682  355.59549   22.09881   1.5136
   2  11.587 MM    0.4798 2.31380e4  803.80383  98.4864

Totals :                  2.34936e4  825.90265
```

Racemic: (*rac-9*)

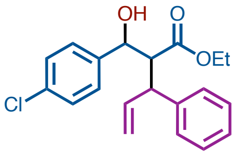


```
======================================================================
Acq. Operator   : SYSTEM                           Seq. Line :   1
Acq. Instrument : 1220 HPLC                         Location : Vial 71
Injection Date  : 12/8/2024 12:16:49 PM                  Inj :   1
                                                Inj Volume : 10.000 µl
Acq. Method     : C:\CHEM32\2\METHODS\MLC_VARIABLE.M
Last changed    : 12/8/2024 12:13:12 PM by SYSTEM
                  (modified after loading)
Analysis Method : C:\CHEM32\2\METHODS\DEF_LC.M
Last changed    : 4/25/2025 2:27:35 PM by SYSTEM
                  (modified after loading)
Sample Info     : IA-3; 95:5 Hex:IPA; 1 mL/min, 220 nm

Additional Info : Peak(s) manually integrated
```

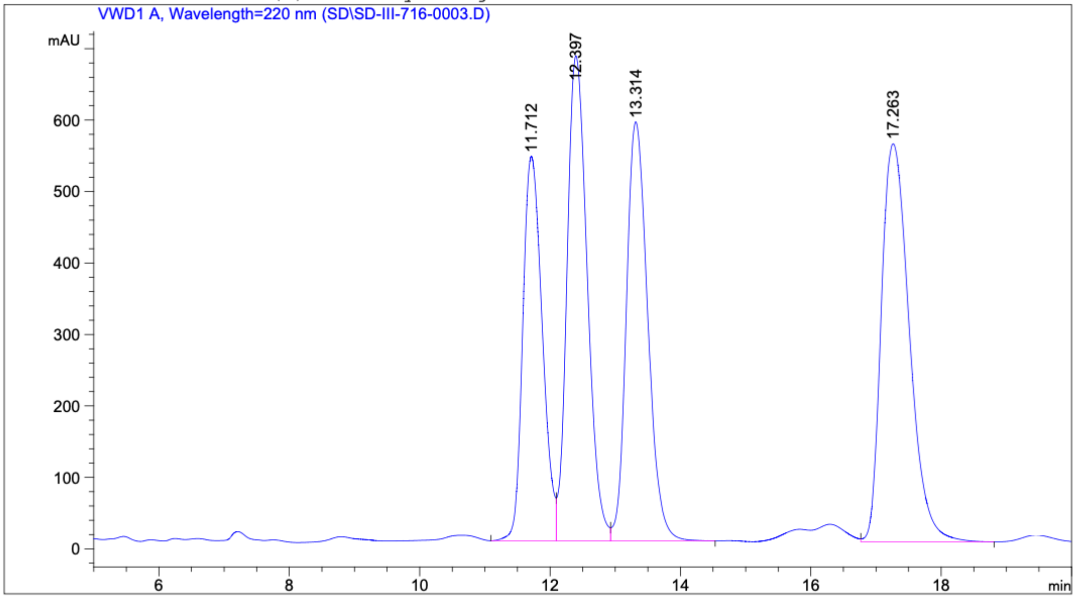


```
======================================================================
                         Area Percent Report
======================================================================

Sorted By             :      Signal
Multiplier:                   :      1.0000
Dilution:                     :      1.0000
Do not use Multiplier & Dilution Factor with ISTDs


Signal 1: VWD1 A, Wavelength=220 nm
```

Peak #	RetTime [min]	Type	Width [min]	Area [mAU*s]	Height [mAU]	Area %
1	11.712	BV	0.3268	1.11497e4	537.88513	19.9735
2	12.397	VV	0.3395	1.46847e4	678.59070	26.3062
3	13.314	VB	0.3567	1.33541e4	586.31750	23.9225
4	17.263	VB	0.4715	1.66338e4	556.92267	29.7978
Totals :				5.58223e4	2359.71600	

Enantioenriched: (*ent-9*)

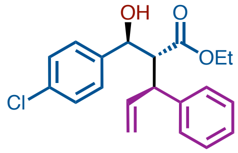


```
====================================================================
Acq. Operator   : SYSTEM                          Seq. Line :    2
Acq. Instrument : 1220 HPLC                        Location : Vial 81
Injection Date  : 12/8/2024 12:42:41 PM                 Inj :    1
                                                 Inj Volume : 10.000 µl
Acq. Method     : C:\CHEM32\2\METHODS\MLC_VARIABLE.M
Last changed    : 12/8/2024 12:13:12 PM by SYSTEM
                  (modified after loading)
Analysis Method : C:\CHEM32\2\METHODS\DEF_LC.M
Last changed    : 4/25/2025 2:25:03 PM by SYSTEM
                  (modified after loading)
Sample Info     : IA-3; 95:5 Hex:IPA; 0.5 mL/min, 220 nm


Additional Info : Peak(s) manually integrated
```

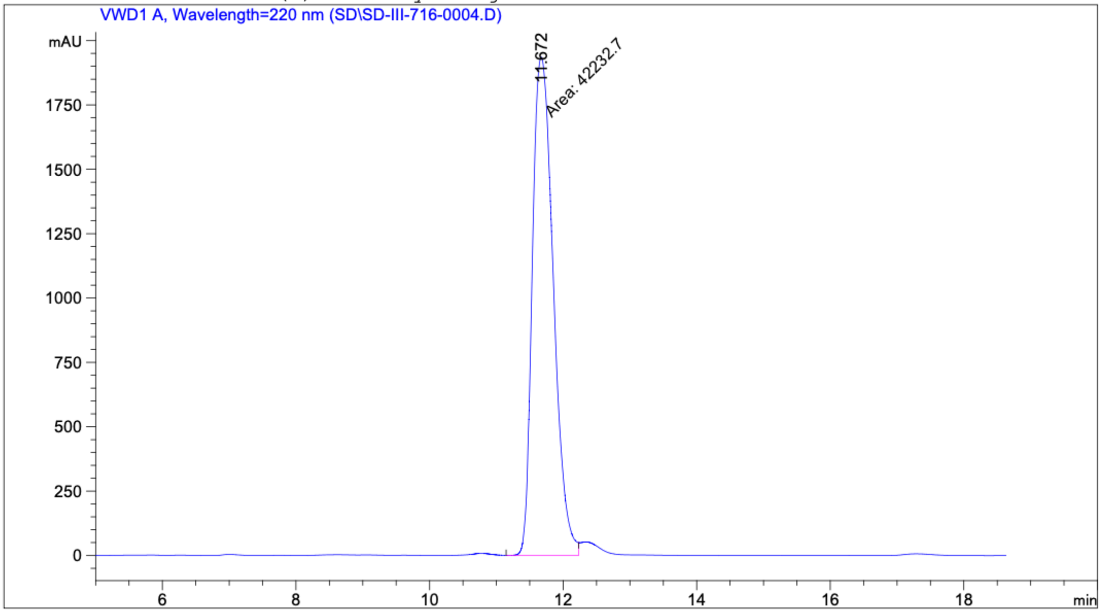


```
====================================================================
                         Area Percent Report
====================================================================

Sorted By             :      Signal
Multiplier:                  :      1.0000
Dilution:                    :      1.0000
Do not use Multiplier & Dilution Factor with ISTDs


Signal 1: VWD1 A, Wavelength=220 nm

Peak RetTime Type  Width     Area      Height     Area
  #   [min]        [min]   [mAU*s]     [mAU]        %
----|-------|----|-------|----------|----------|--------|
   1  11.672 MF    0.3638 4.22327e4  1934.82813 100.0000

Totals :                  4.22327e4  1934.82813
```

Racemic: (*rac-10*)

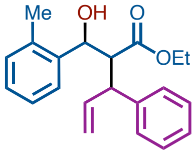


```
======================================================================
Acq. Operator   : SYSTEM                          Seq. Line :    1
Acq. Instrument : 1220 HPLC                        Location : Vial 81
Injection Date  : 12/11/2024 10:27:04 AM                Inj :    1
                                                 Inj Volume : 10.000 µl
Acq. Method     : C:\CHEM32\2\METHODS\MLC_VARIABLE.M
Last changed    : 12/11/2024 10:23:16 AM by SYSTEM
Analysis Method : C:\CHEM32\2\METHODS\DEF_LC.M
Last changed    : 4/25/2025 2:27:35 PM by SYSTEM
                  (modified after loading)
Sample Info     : IA-3- 95:5 Hex:IPA; 1 mL/min, 220 nm


Additional Info : Peak(s) manually integrated
```

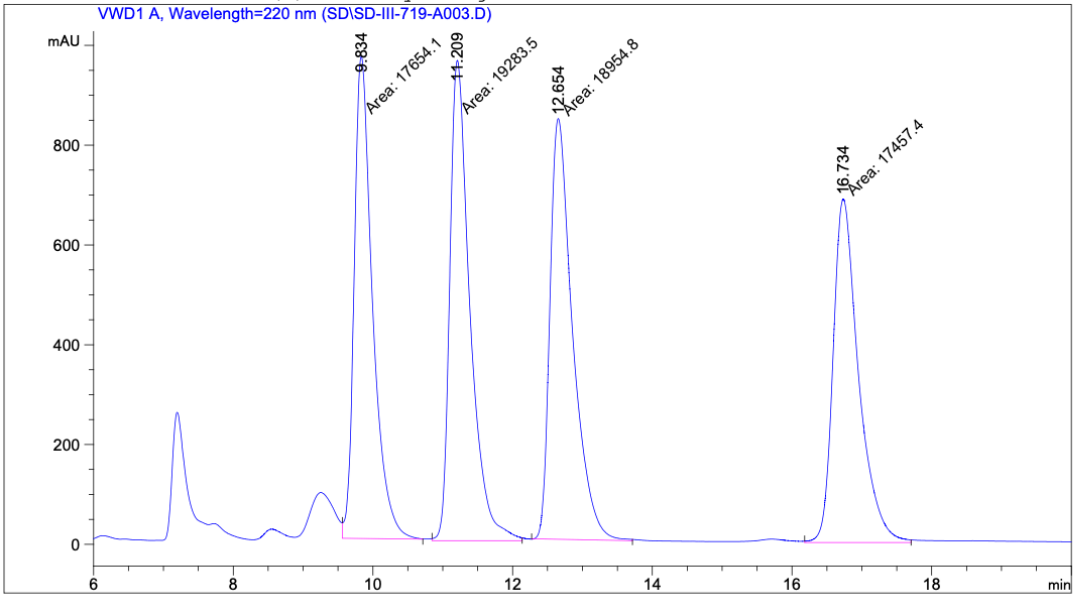


```
======================================================================
                          Area Percent Report
======================================================================

Sorted By              :      Signal
Multiplier:                   :      1.0000
Dilution:                     :      1.0000
Do not use Multiplier & Dilution Factor with ISTDs


Signal 1: VWD1 A, Wavelength=220 nm

Peak RetTime Type  Width     Area      Height     Area
  #   [min]        [min]   [mAU*s]     [mAU]        %
----|-------|----|-------|----------|----------|--------|
   1   9.834 FM    0.3041 1.76541e4  967.49127 24.0684
   2  11.209 MM    0.3339 1.92835e4  962.65466 26.2898
   3  12.654 MM    0.3747 1.89548e4  843.05048 25.8417
   4  16.734 MM    0.4224 1.74574e4  688.82751 23.8002

Totals :                  7.33498e4 3462.02393
```

Enantioenriched: (*ent-10*)

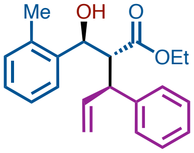


```
======================================================================
Acq. Operator   : SYSTEM                          Seq. Line :   1
Acq. Instrument : 1220 HPLC                        Location : Vial 71
Injection Date  : 12/11/2024 10:56:53 AM                Inj :   1
                                                 Inj Volume : 10.000 µl
Acq. Method     : C:\CHEM32\2\METHODS\MLC_VARIABLE.M
Last changed    : 12/11/2024 10:23:16 AM by SYSTEM
Analysis Method : C:\CHEM32\2\METHODS\DEF_LC.M
Last changed    : 4/25/2025 2:31:17 PM by SYSTEM
                  (modified after loading)
Sample Info     : IA-3- 95:5 Hex:IPA; 1 mL/min, 220 nm

Additional Info : Peak(s) manually integrated
```

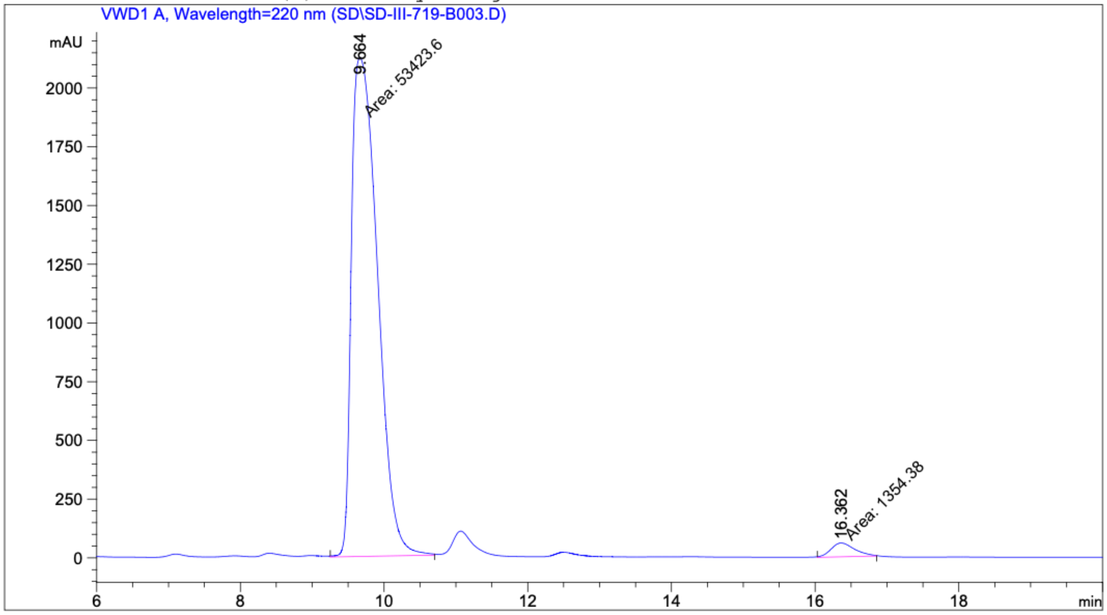


```
======================================================================
                         Area Percent Report
======================================================================

Sorted By             :      Signal
Multiplier:                    :      1.0000
Dilution:                      :      1.0000
Do not use Multiplier & Dilution Factor with ISTDs


Signal 1: VWD1 A, Wavelength=220 nm
```

Peak #	RetTime [min]	Type	Width [min]	Area [mAU*s]	Height [mAU]	Area %
1	9.664	MM	0.4192	5.34236e4	2124.26172	97.5275
2	16.362	MM	0.3838	1354.38196	58.80910	2.4725

Totals : 5.47780e4 2183.07082

Racemic: (*rac-11*)

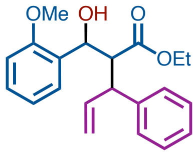


```
=====================================================================
Acq. Operator   : SYSTEM                          Seq. Line :   1
Acq. Instrument : 1220 HPLC                        Location : Vial 81
Injection Date  : 12/11/2024 12:33:51 PM                Inj :   1
                                                 Inj Volume : 10.000 µl
Acq. Method     : C:\CHEM32\2\METHODS\MLC_VARIABLE.M
Last changed    : 12/11/2024 12:29:04 PM by SYSTEM
Analysis Method : C:\CHEM32\2\METHODS\DEF_LC.M
Last changed    : 4/25/2025 2:31:17 PM by SYSTEM
                  (modified after loading)
Sample Info     : IA-3- 95:5 Hex:IPA; 1 mL/min, 220 nm

Additional Info : Peak(s) manually integrated
```

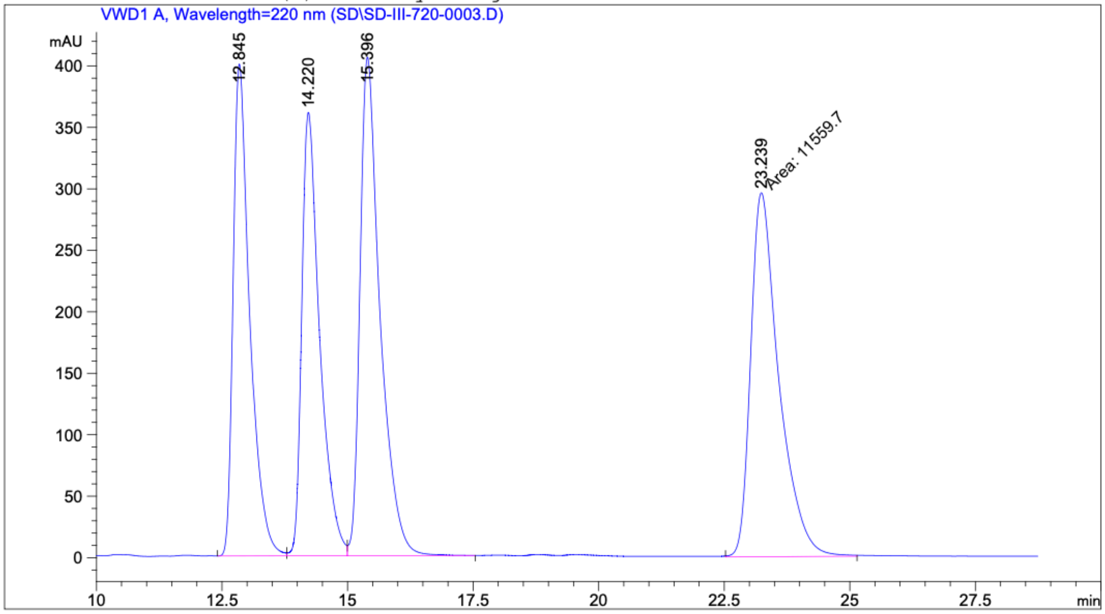


```
=====================================================================
                         Area Percent Report
=====================================================================

Sorted By             :      Signal
Multiplier:                   :      1.0000
Dilution:                     :      1.0000
Do not use Multiplier & Dilution Factor with ISTDs


Signal 1: VWD1 A, Wavelength=220 nm
```

Peak #	RetTime [min]	Type	Width [min]	Area [mAU*s]	Height [mAU]	Area %
1	12.845	BV	0.3394	9205.20020	400.06705	22.4614
2	14.220	VV	0.3680	8963.09766	360.63300	21.8706
3	15.396	VB	0.4079	1.12544e4	405.40820	27.4615
4	23.239	MM	0.6508	1.15597e4	296.02725	28.2065

Totals : 4.09823e4 1462.13550

Enantioenriched: (*ent-11*)

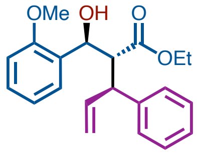


```
==================================================================
Acq. Operator   : SYSTEM                        Seq. Line :   2
Acq. Instrument : 1220 HPLC                      Location : Vial 71
Injection Date  : 12/11/2024 1:04:44 PM               Inj :   1
                                               Inj Volume : 10.000 µl
Acq. Method     : C:\CHEM32\2\METHODS\MLC_VARIABLE.M
Last changed    : 12/11/2024 12:29:04 PM by SYSTEM
Analysis Method : C:\CHEM32\2\METHODS\DEF_LC.M
Last changed    : 4/25/2025 2:33:23 PM by SYSTEM
                  (modified after loading)
Sample Info     : IA-3- 95:5 Hex:IPA; 1 mL/min, 220 nm


Additional Info : Peak(s) manually integrated
```

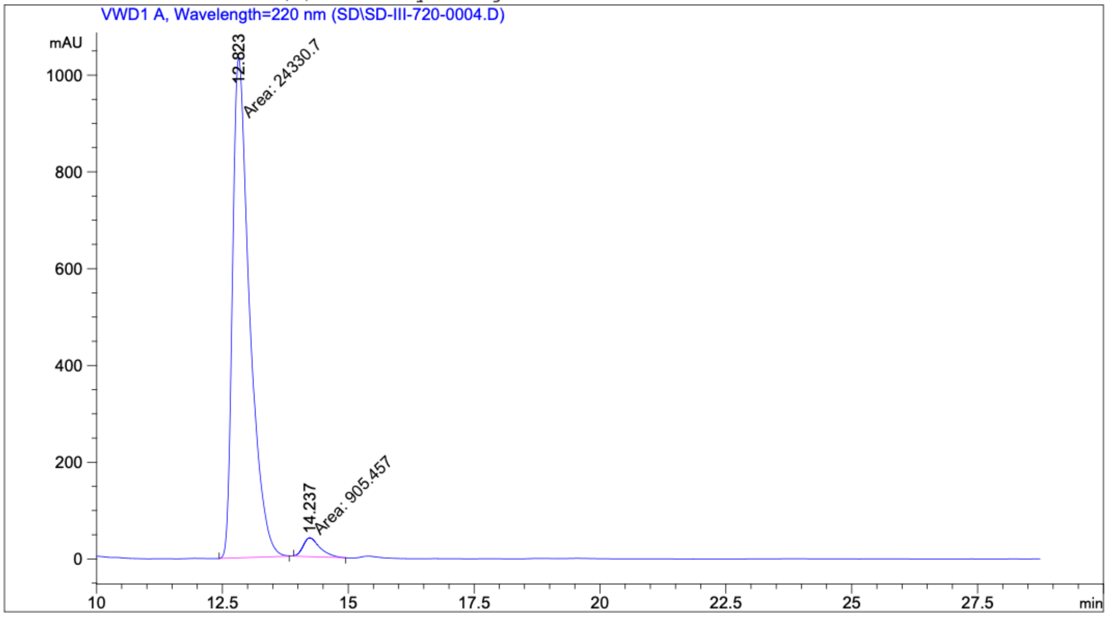


```
==================================================================
                         Area Percent Report
==================================================================

Sorted By             :      Signal
Multiplier:                   :      1.0000
Dilution:                     :      1.0000
Do not use Multiplier & Dilution Factor with ISTDs


Signal 1: VWD1 A, Wavelength=220 nm

Peak RetTime Type  Width     Area      Height     Area
  #   [min]        [min]   [mAU*s]     [mAU]        %
----|-------|----|-------|----------|----------|--------|
   1  12.823 MM     0.3924 2.43307e4  1033.35742  96.4121
   2  14.237 MM     0.3869  905.45679   39.00030   3.5879

Totals :                   2.52362e4  1072.35772
```

Racemic: (*rac-12*)

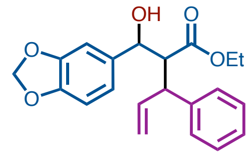


```
=====================================================================
Acq. Operator   : SYSTEM                          Seq. Line :    1
Acq. Instrument : 1220 HPLC                        Location : Vial 81
Injection Date  : 11/28/2024 3:24:40 PM                 Inj :    1
                                                 Inj Volume : 20.000 µl
Acq. Method     : C:\CHEM32\2\METHODS\MLC_VARIABLE.M
Last changed    : 11/28/2024 3:21:04 PM by SYSTEM
                  (modified after loading)
Analysis Method : C:\CHEM32\2\METHODS\DEF_LC.M
Last changed    : 7/19/2025 11:43:49 AM by SYSTEM
                  (modified after loading)
Sample Info     : IA-3; 95:5 Hex:IPA; 1 mL/min, 220 nm


Additional Info : Peak(s) manually integrated
```

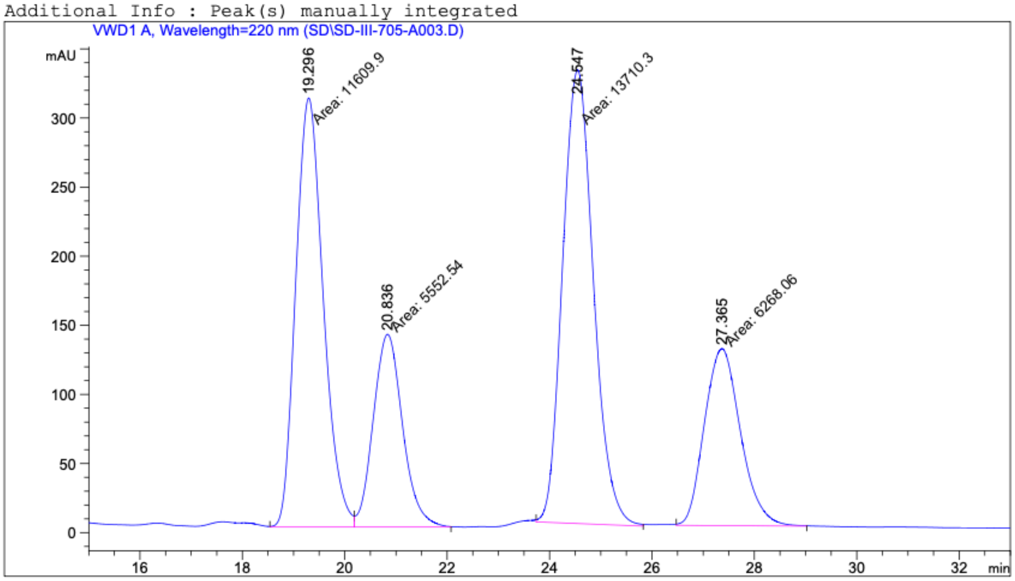


```
=====================================================================
                         Area Percent Report
=====================================================================

Sorted By             :      Signal
Multiplier:                   :      1.0000
Dilution:                     :      1.0000
Do not use Multiplier & Dilution Factor with ISTDs


Signal 1: VWD1 A, Wavelength=220 nm
```

Peak #	RetTime [min]	Type	Width [min]	Area [mAU*s]	Height [mAU]	Area %
1	19.296	MF	0.6234	1.16099e4	310.37576	31.2592
2	20.836	FM	0.6638	5552.54443	139.41255	14.9500
3	24.547	MM	0.6964	1.37103e4	328.14117	36.9144
4	27.365	MM	0.8162	6268.06006	127.98595	16.8765

Totals : 3.71408e4 905.91544

Enantioenriched: (*ent-12*)

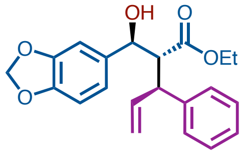


```
========================================================================
Acq. Operator   : SYSTEM                          Seq. Line :    1
Acq. Instrument : 1220 HPLC                        Location : Vial 71
Injection Date  : 11/28/2024 4:06:29 PM                  Inj :    1
                                                Inj Volume : 20.000 µl
Acq. Method     : C:\CHEM32\2\METHODS\MLC_VARIABLE.M
Last changed    : 11/28/2024 4:02:34 PM by SYSTEM
Analysis Method : C:\CHEM32\2\METHODS\DEF_LC.M
Last changed    : 7/19/2025 11:43:49 AM by SYSTEM
                  (modified after loading)
Sample Info     : IA-3; 95:5 Hex:IPA; 1 mL/min, 220 nm


Additional Info : Peak(s) manually integrated
```

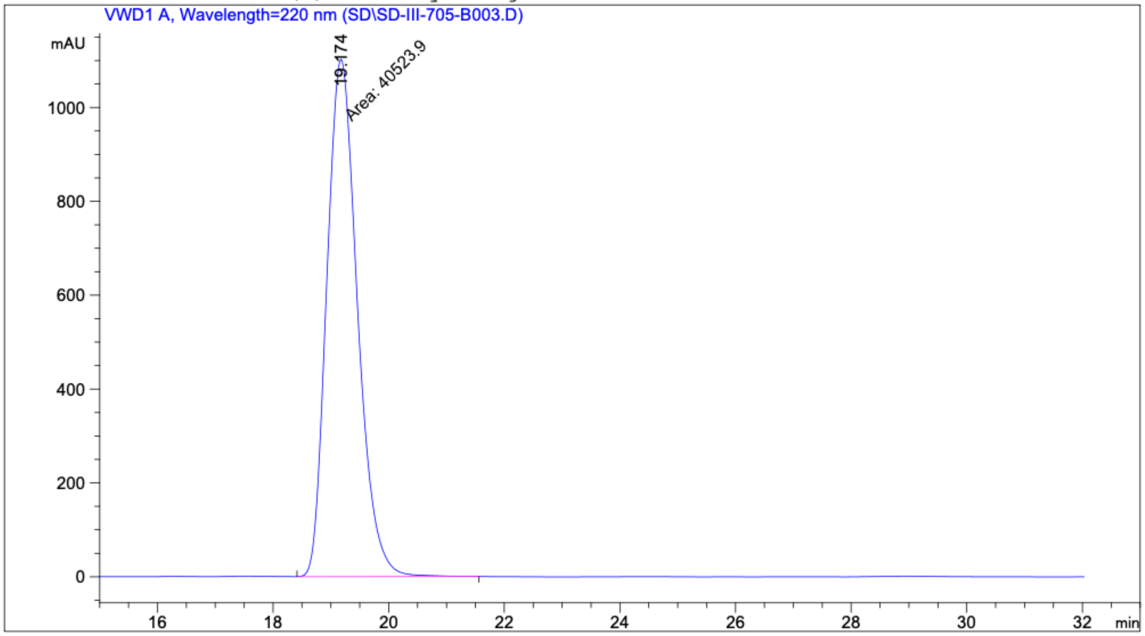


```
========================================================================
                          Area Percent Report
========================================================================

Sorted By             :      Signal
Multiplier:                   :      1.0000
Dilution:                     :      1.0000
Do not use Multiplier & Dilution Factor with ISTDs


Signal 1: VWD1 A, Wavelength=220 nm

Peak RetTime Type  Width     Area      Height     Area
  #   [min]        [min]   [mAU*s]     [mAU]        %
----|-------|----|-------|----------|----------|--------|
   1  19.174 MM    0.6129 4.05239e4  1102.05029 100.0000

Totals :                  4.05239e4  1102.05029
```

Racemic: (*rac-13*)

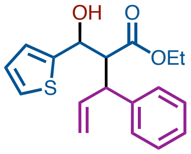


```
=====================================================================
Acq. Operator   : SYSTEM                          Seq. Line :   1
Acq. Instrument : 1220 HPLC                        Location : Vial 81
Injection Date  : 12/3/2024 3:59:12 PM                  Inj :   1
                                                 Inj Volume : 10.000 µl
Acq. Method     : C:\CHEM32\2\METHODS\MLC_VARIABLE.M
Last changed    : 12/3/2024 4:29:32 PM by SYSTEM
                  (modified after loading)
Analysis Method : C:\CHEM32\2\METHODS\DEF_LC.M
Last changed    : 4/25/2025 2:41:18 PM by SYSTEM
                  (modified after loading)
Sample Info     : IA-3; 95:5 Hex:IPA; 1 mL/min, 220 nm


Additional Info : Peak(s) manually integrated
```

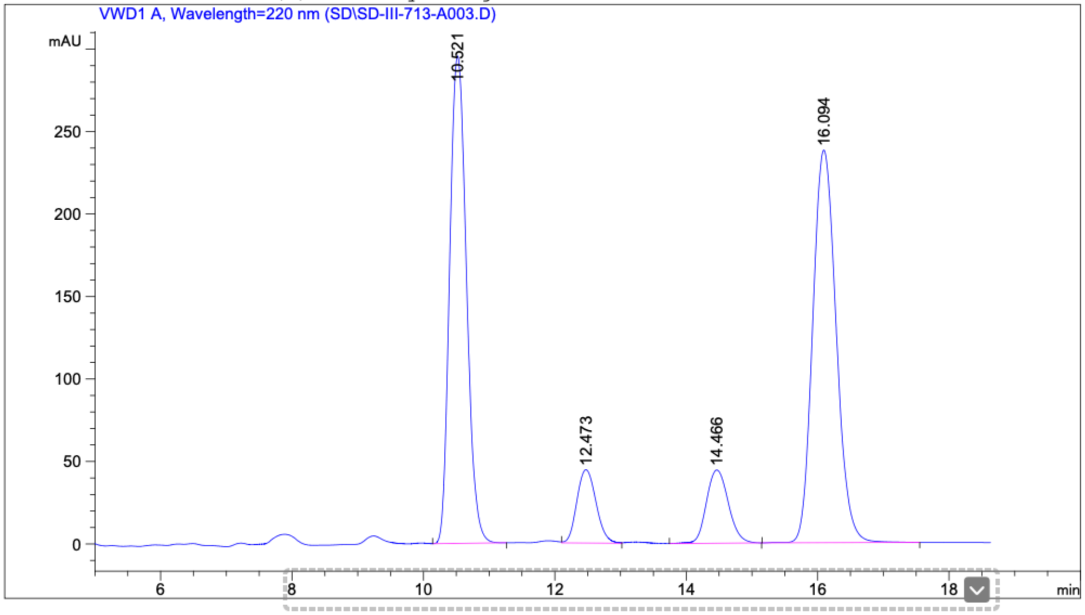


```
=====================================================================
                         Area Percent Report
=====================================================================

Sorted By             :      Signal
Multiplier:                  :      1.0000
Dilution:                    :      1.0000
Do not use Multiplier & Dilution Factor with ISTDs


Signal 1: VWD1 A, Wavelength=220 nm

Peak RetTime Type  Width     Area      Height     Area
  #   [min]        [min]   [mAU*s]     [mAU]       %
----|-------|----|-------|----------|----------|--------|
   1  10.521 BB    0.2816 5231.85156  295.37778  40.0801
   2  12.473 VV    0.3230  902.46753   44.42820   6.9136
   3  14.466 BB    0.3668 1025.73584   44.35796   7.8579
   4  16.094 BB    0.3909 5893.42627  237.96979  45.1483

Totals :                  1.30535e4   622.13372
```

Enantioenriched: (*ent-13*)

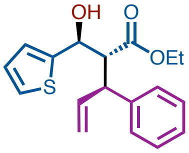


```
=====================================================================
Acq. Operator   : SYSTEM                          Seq. Line :   1
Acq. Instrument : 1220 HPLC                        Location : Vial 71
Injection Date  : 12/3/2024 4:34:11 PM                  Inj :   1
                                                 Inj Volume : 35.000 µl
Acq. Method     : C:\CHEM32\2\METHODS\MLC_VARIABLE.M
Last changed    : 12/3/2024 4:30:39 PM by SYSTEM
                  (modified after loading)
Analysis Method : C:\CHEM32\2\METHODS\DEF_LC.M
Last changed    : 4/25/2025 2:44:20 PM by SYSTEM
                  (modified after loading)
Sample Info     : IA-3; 95:5 Hex:IPA; 1 mL/min, 220 nm


Additional Info : Peak(s) manually integrated
```

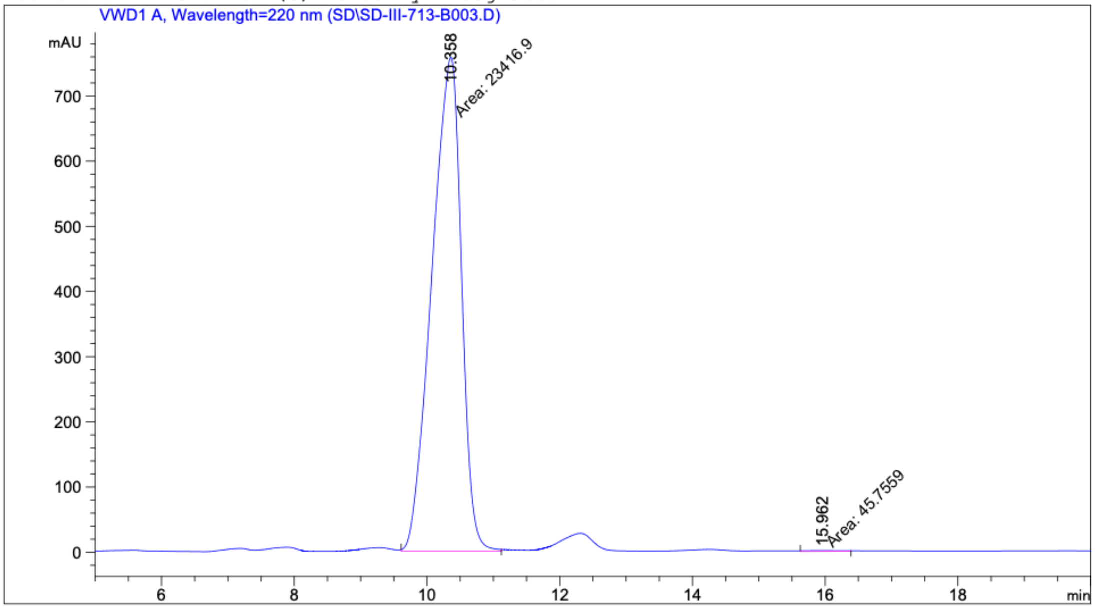


```
=====================================================================
                         Area Percent Report
=====================================================================

Sorted By             :      Signal
Multiplier:                   :      1.0000
Dilution:                     :      1.0000
Do not use Multiplier & Dilution Factor with ISTDs


Signal 1: VWD1 A, Wavelength=220 nm

Peak RetTime Type  Width     Area      Height     Area
  #   [min]        [min]   [mAU*s]     [mAU]        %
----|-------|----|-------|----------|----------|--------|
   1  10.358 MM    0.5153 2.34169e4  757.40106 99.8050
   2  15.962 MM    0.4986   45.75589    1.16400  0.1950

Totals :                  2.34627e4  758.56506
```

Racemic: (*rac-14)*

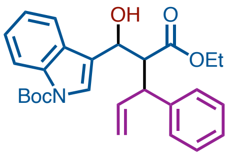

```
=====================================================================
Acq. Operator   : SYSTEM                          Seq. Line :   1
Acq. Instrument : 1220 HPLC                        Location : Vial 81
Injection Date  : 11/18/2024 7:18:45 PM                 Inj :   1
                                                 Inj Volume : 10.000 µl
Acq. Method     : C:\CHEM32\2\METHODS\MLC_VARIABLE.M
Last changed    : 11/18/2024 7:33:52 PM by SYSTEM
                  (modified after loading)
Analysis Method : C:\CHEM32\2\METHODS\DEF_LC.M
Last changed    : 4/25/2025 2:37:43 PM by SYSTEM
                  (modified after loading)
Sample Info     : C-1; Hex:IPA 99:1, 0.5 mL/min; 220nm


Additional Info : Peak(s) manually integrated
```

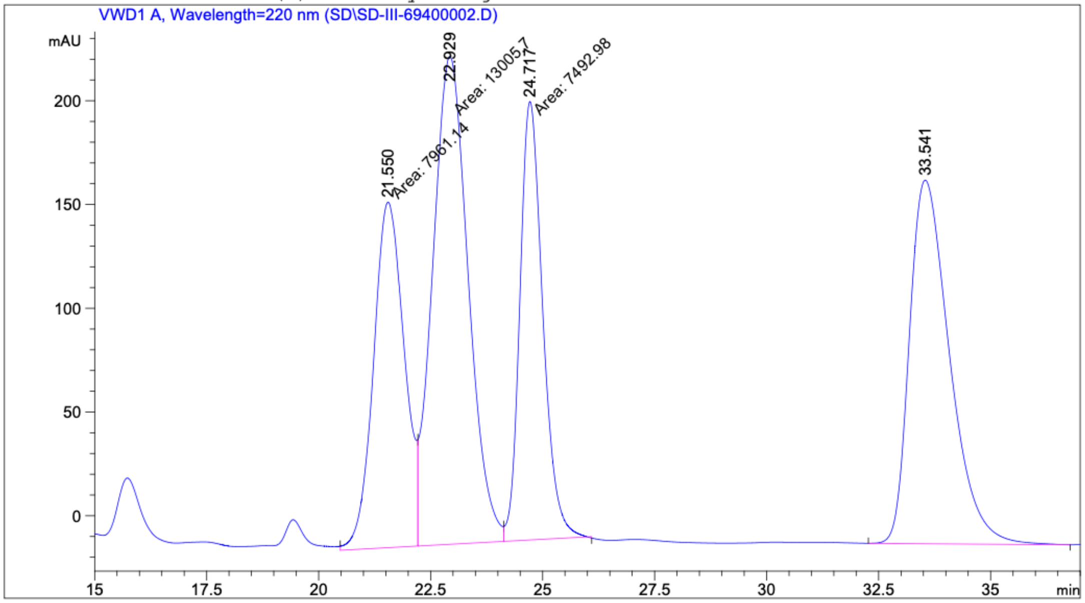


```
=====================================================================
                      Area Percent Report
=====================================================================

Sorted By             :      Signal
Multiplier:                   :      1.0000
Dilution:                     :      1.0000
Do not use Multiplier & Dilution Factor with ISTDs


Signal 1: VWD1 A, Wavelength=220 nm
```

Peak #	RetTime [min]	Type	Width [min]	Area [mAU*s]	Height [mAU]	Area %
1	21.550	MF	0.7969	7961.13574	166.49406	20.2334
2	22.929	FM	0.9220	1.30057e4	235.10927	33.0542
3	24.717	FM	0.5911	7492.98096	211.26907	19.0436
4	33.541	BB	0.9539	1.08867e4	175.21419	27.6689
Totals :				3.93465e4	788.08659	

Enantioenriched: (*ent-14*)

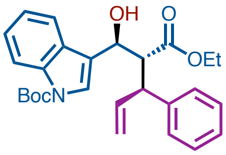


```
=====================================================================
Acq. Operator   : SYSTEM                          Seq. Line :   2
Acq. Instrument : 1220 HPLC                        Location : Vial 71
Injection Date  : 11/18/2024 7:56:38 PM                 Inj :   1
                                                 Inj Volume : 10.000 µl
Acq. Method     : C:\CHEM32\2\METHODS\MLC_VARIABLE.M
Last changed    : 11/18/2024 7:33:52 PM by SYSTEM
                  (modified after loading)
Analysis Method : C:\CHEM32\2\METHODS\DEF_LC.M
Last changed    : 4/25/2025 2:38:08 PM by SYSTEM
                  (modified after loading)
Sample Info     : IA-3, 95:5 HEX/IPA, 1.0 ml/min, 220 nm,


Additional Info : Peak(s) manually integrated
```

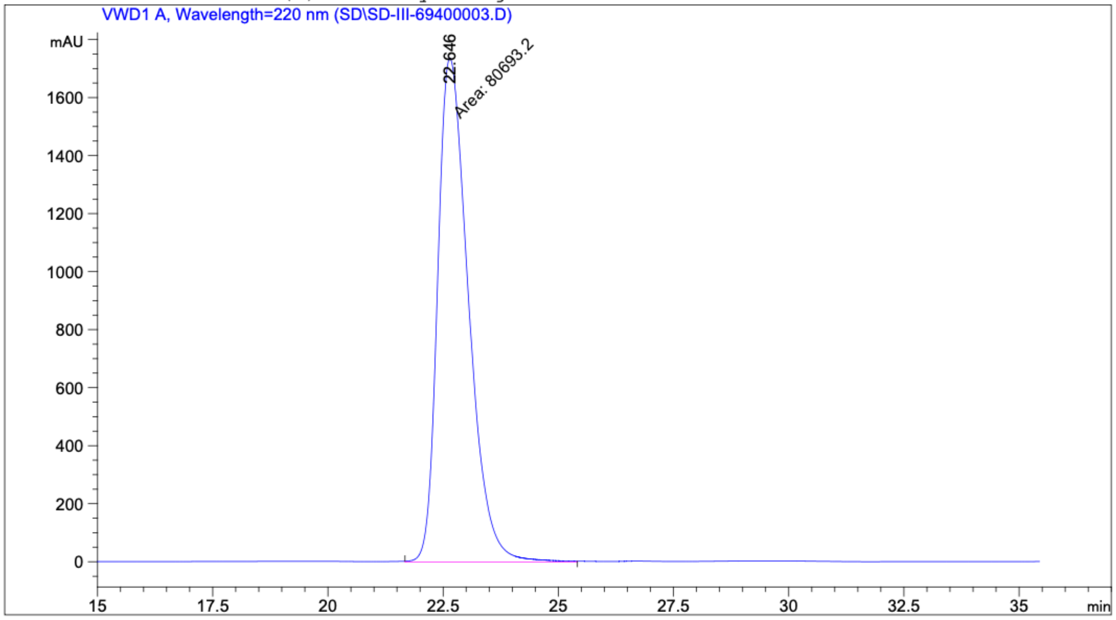


```
=====================================================================
                         Area Percent Report
=====================================================================

Sorted By             :      Signal
Multiplier:                   :      1.0000
Dilution:                     :      1.0000
Do not use Multiplier & Dilution Factor with ISTDs


Signal 1: VWD1 A, Wavelength=220 nm

Peak RetTime Type  Width     Area      Height     Area
  #   [min]        [min]   [mAU*s]     [mAU]        %
----|-------|----|-------|----------|----------|--------|
   1  22.646 MM    0.7738 8.06932e4  1738.05811 100.0000

Totals :                  8.06932e4  1738.05811
```

Racemic: (*rac-16*)

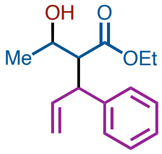


```
=====================================================================
Acq. Operator   : SYSTEM                          Seq. Line :   1
Acq. Instrument : 1220 HPLC                        Location : Vial 81
Injection Date  : 12/21/2024 3:34:20 PM                 Inj :   1
                                                 Inj Volume : 10.000 µl
Acq. Method     : C:\CHEM32\2\METHODS\MLC_VARIABLE.M
Last changed    : 12/21/2024 3:30:46 PM by SYSTEM
                  (modified after loading)
Analysis Method : C:\CHEM32\2\METHODS\DEF_LC.M
Last changed    : 4/25/2025 2:47:35 PM by SYSTEM
                  (modified after loading)
Sample Info     : IA-3- 98:2 Hex:IPA; 1 mL/min, 220 nm


Additional Info : Peak(s) manually integrated
```

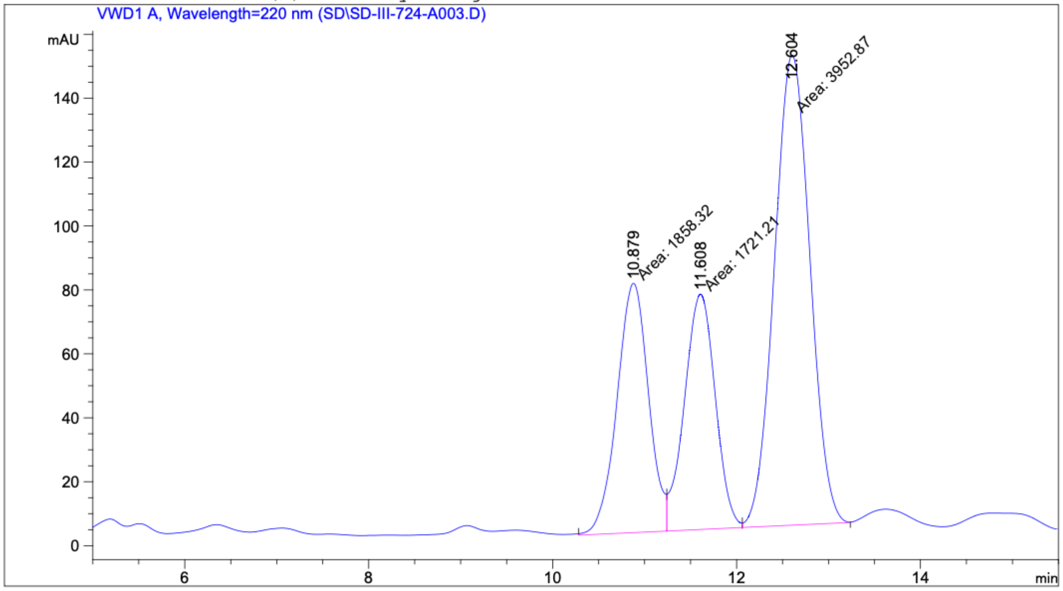


```
=====================================================================
                         Area Percent Report
=====================================================================

Sorted By             :      Signal
Multiplier:                  :      1.0000
Dilution:                    :      1.0000
Do not use Multiplier & Dilution Factor with ISTDs


Signal 1: VWD1 A, Wavelength=220 nm
```

Peak #	RetTime [min]	Type	Width [min]	Area [mAU*s]	Height [mAU]	Area %
1	10.879	MF	0.3971	1858.32324	77.99480	24.6710
2	11.608	MF	0.3898	1721.21191	73.59959	22.8508
3	12.604	FM	0.4479	3952.87256	147.07979	52.4782

Totals : 7532.40771 298.67419

Enantioenriched: (*ent-16*)

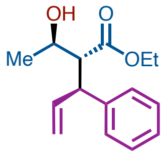


```
=====================================================================
Acq. Operator   : SYSTEM                            Seq. Line :   1
Acq. Instrument : 1220 HPLC                          Location : Vial 71
Injection Date  : 12/21/2024 3:57:32 PM                   Inj :   1
                                                   Inj Volume : 10.000 µl
Acq. Method     : C:\CHEM32\2\METHODS\MLC_VARIABLE.M
Last changed    : 12/21/2024 3:53:44 PM by SYSTEM
Analysis Method : C:\CHEM32\2\METHODS\DEF_LC.M
Last changed    : 4/25/2025 2:47:35 PM by SYSTEM
                  (modified after loading)
Sample Info     : IA-3- 98:2 Hex:IPA; 1 mL/min, 220 nm


Additional Info : Peak(s) manually integrated
```

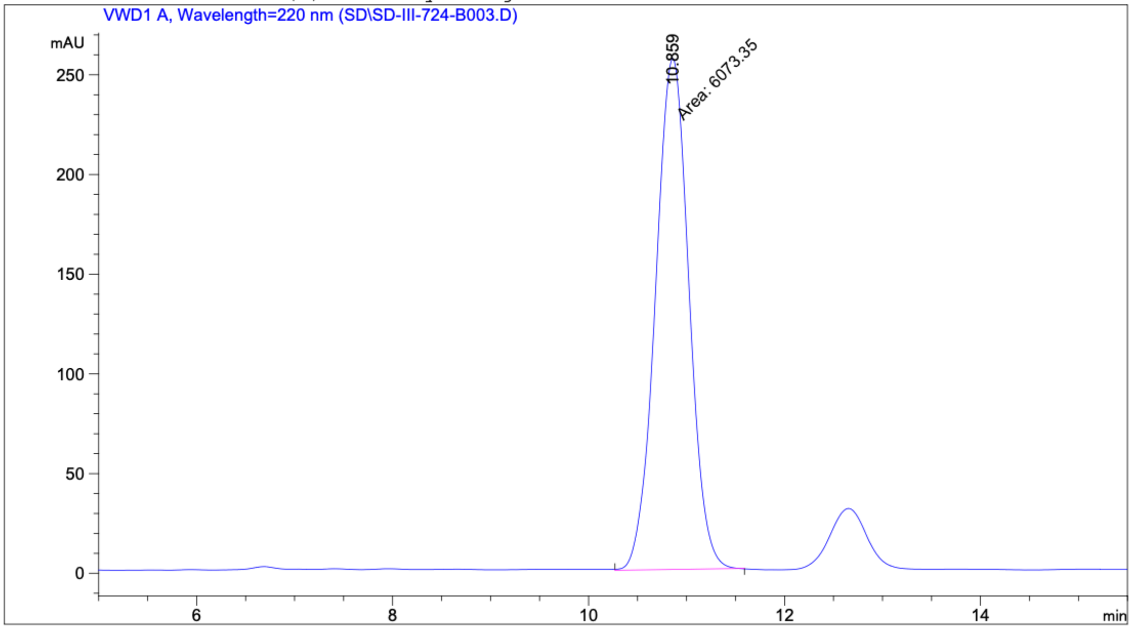


```
=====================================================================
                         Area Percent Report
=====================================================================

Sorted By             :      Signal
Multiplier:                   :      1.0000
Dilution:                     :      1.0000
Do not use Multiplier & Dilution Factor with ISTDs


Signal 1: VWD1 A, Wavelength=220 nm

Peak RetTime Type  Width     Area      Height     Area
  #   [min]        [min]   [mAU*s]     [mAU]       %
----|-------|----|-------|----------|----------|--------|
   1  10.859 MM    0.3951 6073.35010  256.17422 100.0000

Totals :                  6073.35010  256.17422
```

Racemic: (*rac-17*)

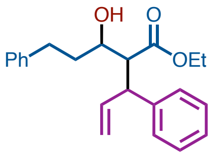


```
====================================================================
Acq. Operator   : SYSTEM                          Seq. Line :   1
Acq. Instrument : 1220 HPLC                        Location : Vial 81
Injection Date  : 1/4/2025 4:09:02 PM                   Inj :   1
                                                 Inj Volume : 10.000 µl
Acq. Method     : C:\CHEM32\2\METHODS\MLC_VARIABLE.M
Last changed    : 1/4/2025 4:04:27 PM by SYSTEM
                  (modified after loading)
Analysis Method : C:\CHEM32\2\METHODS\DEF_LC.M
Last changed    : 4/25/2025 2:47:35 PM by SYSTEM
                  (modified after loading)
Sample Info     : C-1_98:2  Hex:IPA;0.5 mL/min, 220 nm


Additional Info : Peak(s) manually integrated
```

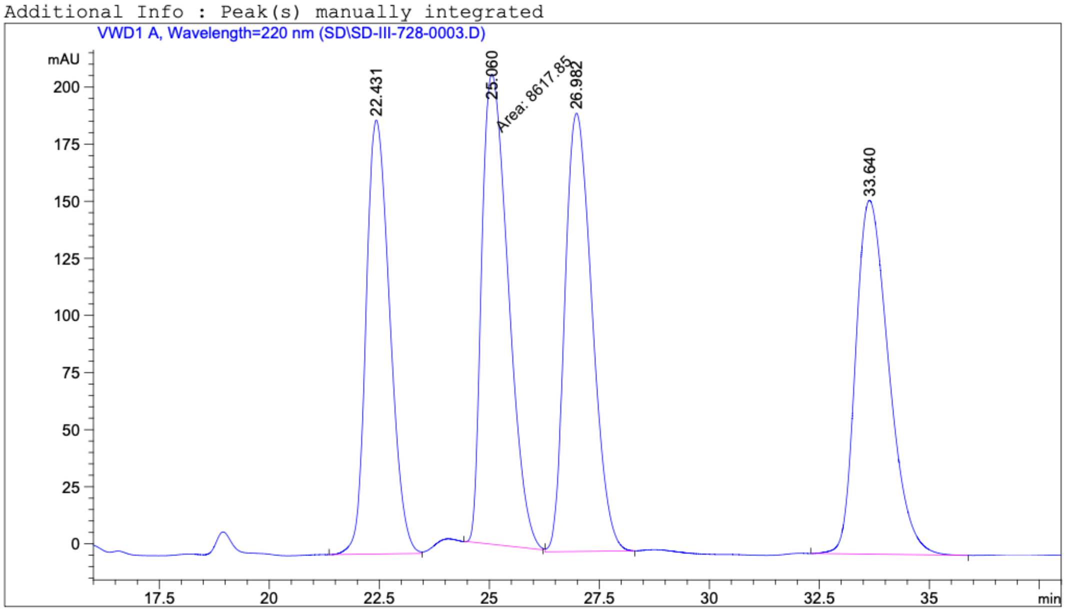


```
====================================================================
                         Area Percent Report
====================================================================

Sorted By             :      Signal
Multiplier:                  :      1.0000
Dilution:                    :      1.0000
Do not use Multiplier & Dilution Factor with ISTDs


Signal 1: VWD1 A, Wavelength=220 nm
```

Peak #	RetTime [min]	Type	Width [min]	Area [mAU*s]	Height [mAU]	Area %
1	22.431	BV	0.5899	7117.11426	190.04474	22.2005
2	25.060	MM	0.6978	8617.85156	205.82762	26.8817
3	26.982	VB	0.6803	8230.56152	191.92062	25.6736
4	33.640	BB	0.8143	8092.88428	154.94720	25.2442

Totals : 3.20584e4 742.74019

Enantioenriched: (*ent-17*)

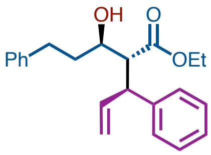


```
======================================================================
Acq. Operator   : SYSTEM                          Seq. Line :    2
Acq. Instrument : 1220 HPLC                        Location : Vial 71
Injection Date  : 1/4/2025 4:49:54 PM                   Inj :    1
                                                 Inj Volume : 10.000 µl
Acq. Method     : C:\CHEM32\2\METHODS\MLC_VARIABLE.M
Last changed    : 1/4/2025 4:04:27 PM by SYSTEM
                  (modified after loading)
Analysis Method : C:\CHEM32\2\METHODS\DEF_LC.M
Last changed    : 4/25/2025 3:02:57 PM by SYSTEM
                  (modified after loading)
Sample Info     : C-1_98:2  Hex:IPA; 1mL/min, 220 nm


Additional Info : Peak(s) manually integrated
```

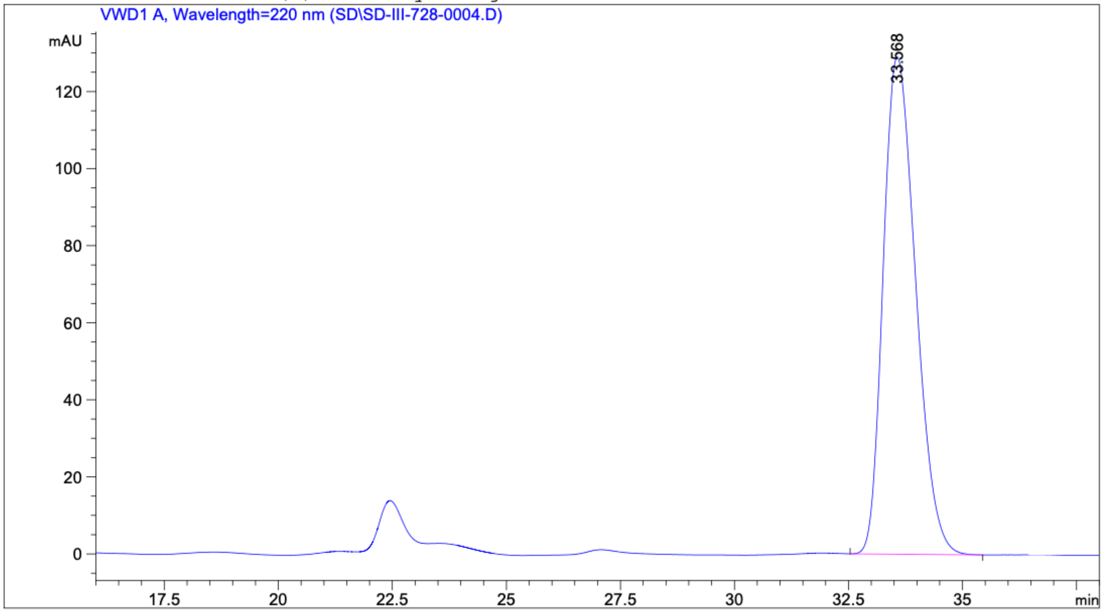


```
======================================================================
                         Area Percent Report
======================================================================

Sorted By             :      Signal
Multiplier:                   :      1.0000
Dilution:                     :      1.0000
Do not use Multiplier & Dilution Factor with ISTDs


Signal 1: VWD1 A, Wavelength=220 nm

Peak RetTime Type  Width     Area      Height     Area
  #   [min]        [min]   [mAU*s]     [mAU]        %
----|-------|----|-------|----------|----------|--------|
   1  33.568 BB    0.7789 6381.16699  129.04025 100.0000

Totals :                  6381.16699  129.04025
```

Racemic: (*rac-18*)

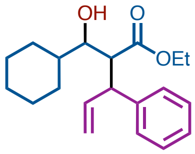


```
=====================================================================
Acq. Operator   : SYSTEM                          Seq. Line :    1
Acq. Instrument : 1220 HPLC                        Location : Vial 71
Injection Date  : 12/13/2024 12:24:16 PM                Inj :    1
                                                 Inj Volume : 10.000 µl
Acq. Method     : C:\CHEM32\2\METHODS\MLC_VARIABLE.M
Last changed    : 12/13/2024 12:20:41 PM by SYSTEM
                  (modified after loading)
Analysis Method : C:\CHEM32\2\METHODS\DEF_LC.M
Last changed    : 4/25/2025 3:02:57 PM by SYSTEM
                  (modified after loading)
Sample Info     : IA-3- 90:10 Hex:IPA; 1 mL/min, 220 nm

Additional Info : Peak(s) manually integrated
```

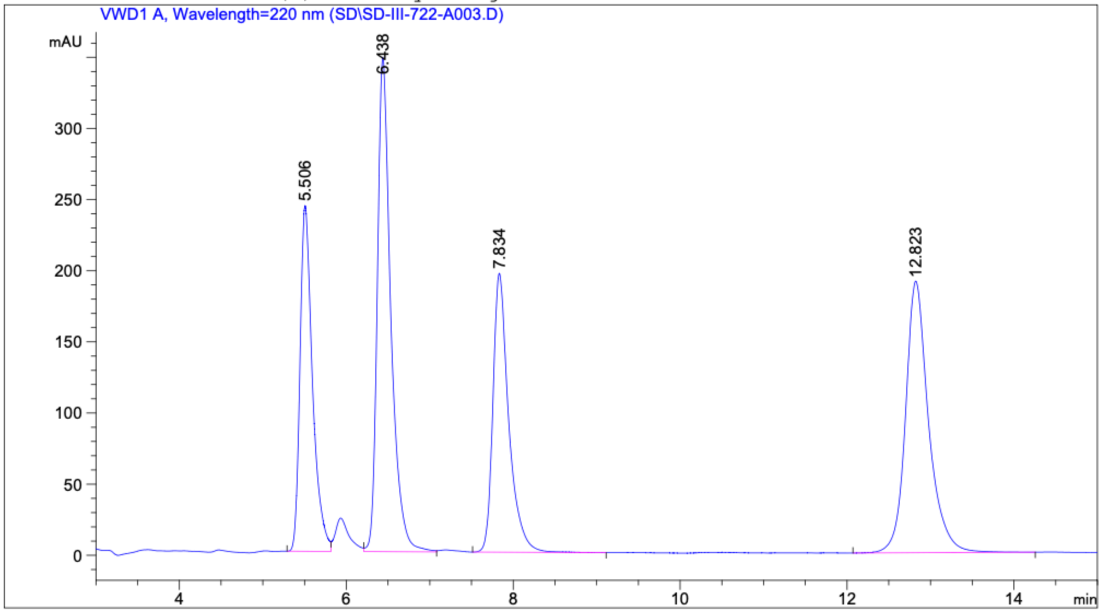


```
=====================================================================
                         Area Percent Report
=====================================================================

Sorted By             :      Signal
Multiplier:                  :      1.0000
Dilution:                    :      1.0000
Do not use Multiplier & Dilution Factor with ISTDs


Signal 1: VWD1 A, Wavelength=220 nm
```

Peak #	RetTime [min]	Type	Width [min]	Area [mAU*s]	Height [mAU]	Area %
1	5.506	BV	0.1550	2518.39624	242.84927	19.9220
2	6.438	VV	0.1659	3899.63184	347.49408	30.8483
3	7.834	BB	0.1951	2571.53271	195.94342	20.3423
4	12.823	BB	0.2832	3651.74756	190.83496	28.8874

Totals : 1.26413e4 977.12173

Enantioenriched: (*ent-18*)

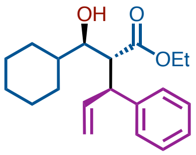


```
======================================================================
Acq. Operator   : SYSTEM                          Seq. Line :   1
Acq. Instrument : 1220 HPLC                        Location : Vial 61
Injection Date  : 12/13/2024 1:31:40 PM                 Inj :   1
                                                 Inj Volume : 25.000 µl
Acq. Method     : C:\CHEM32\2\METHODS\MLC_VARIABLE.M
Last changed    : 12/13/2024 1:26:05 PM by SYSTEM
                  (modified after loading)
Analysis Method : C:\CHEM32\2\METHODS\DEF_LC.M
Last changed    : 4/25/2025 3:07:40 PM by SYSTEM
                  (modified after loading)
Sample Info     : IA-3- 90:10 Hex:IPA; 1 mL/min, 220 nm


Additional Info : Peak(s) manually integrated
```

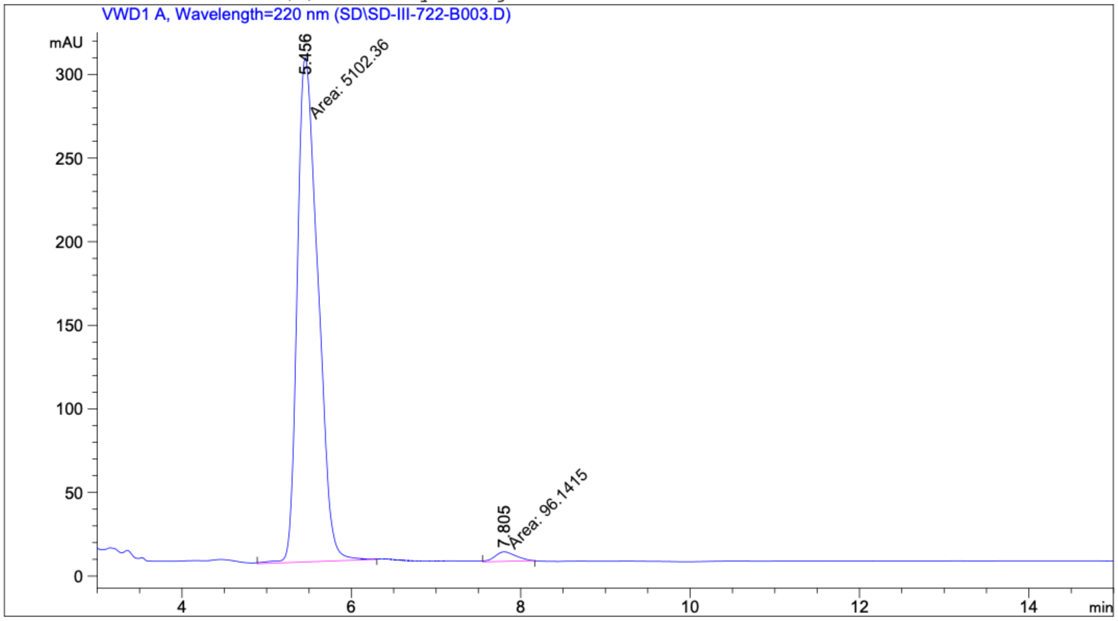


```
======================================================================
                         Area Percent Report
======================================================================

Sorted By             :      Signal
Multiplier:                    :      1.0000
Dilution:                      :      1.0000
Do not use Multiplier & Dilution Factor with ISTDs


Signal 1: VWD1 A, Wavelength=220 nm
```

Peak #	RetTime [min]	Type	Width [min]	Area [mAU*s]	Height [mAU]	Area %
1	5.456	MM	0.2821	5102.36377	301.48477	98.1506
2	7.805	MM	0.2835	96.14147	5.65215	1.8494

Totals : 5198.50524 307.13692

Racemic: (*rac-19*)

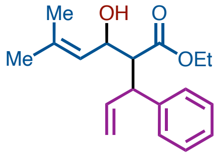


```
======================================================================
Acq. Operator   : SYSTEM                             Seq. Line :    1
Acq. Instrument : 1220 HPLC                           Location : Vial 81
Injection Date  : 11/22/2024 11:26:01 AM                   Inj :    1
                                                    Inj Volume : 10.000 µl
Acq. Method     : C:\CHEM32\2\METHODS\MLC_VARIABLE.M
Last changed    : 11/22/2024 12:08:11 PM by SYSTEM
                  (modified after loading)
Analysis Method : C:\CHEM32\2\METHODS\DEF_LC.M
Last changed    : 4/25/2025 3:07:40 PM by SYSTEM
                  (modified after loading)
Sample Info     : C2; 95:5 Hex:IPA; 1 mL/min, 220 nm


Additional Info : Peak(s) manually integrated
```

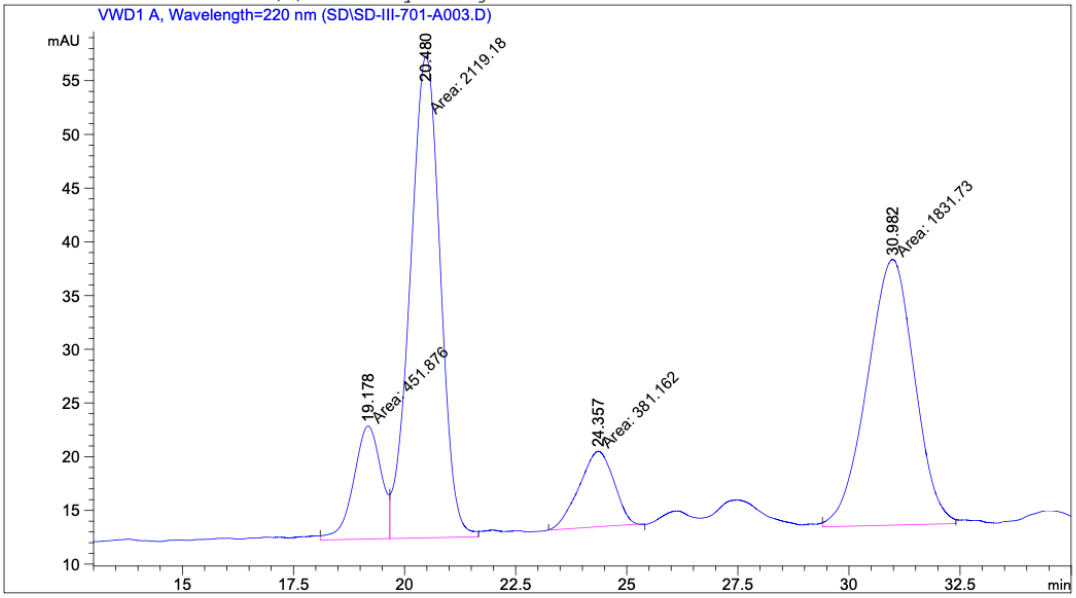


```
======================================================================
                         Area Percent Report
======================================================================

Sorted By             :      Signal
Multiplier:                   :      1.0000
Dilution:                     :      1.0000
Do not use Multiplier & Dilution Factor with ISTDs


Signal 1: VWD1 A, Wavelength=220 nm
```

Peak #	RetTime [min]	Type	Width [min]	Area [mAU*s]	Height [mAU]	Area %
1	19.178	MF	0.7160	451.87601	10.51836	9.4457
2	20.480	FM	0.7878	2119.18457	44.83556	44.2978
3	24.357	MM	0.9045	381.16235	7.02307	7.9675
4	30.982	MM	1.2351	1831.73059	24.71858	38.2891

Totals : 4783.95352 87.09557

Enantioenriched: (*ent-19*)

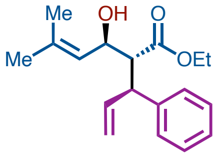


```
=====================================================================
Acq. Operator   : SYSTEM                        Seq. Line :    1
Acq. Instrument : 1220 HPLC                      Location : Vial 71
Injection Date  : 11/22/2024 1:24:53 PM               Inj :    1
                                               Inj Volume : 7.500 µl
Acq. Method     : C:\CHEM32\2\METHODS\MLC_VARIABLE.M
Last changed    : 11/22/2024 1:21:07 PM by SYSTEM
Analysis Method : C:\CHEM32\2\METHODS\DEF_LC.M
Last changed    : 4/25/2025 3:09:28 PM by SYSTEM
                  (modified after loading)
Sample Info     : C2; 98:2 Hex:IPA; 1 mL/min, 220 nm


Additional Info : Peak(s) manually integrated
```

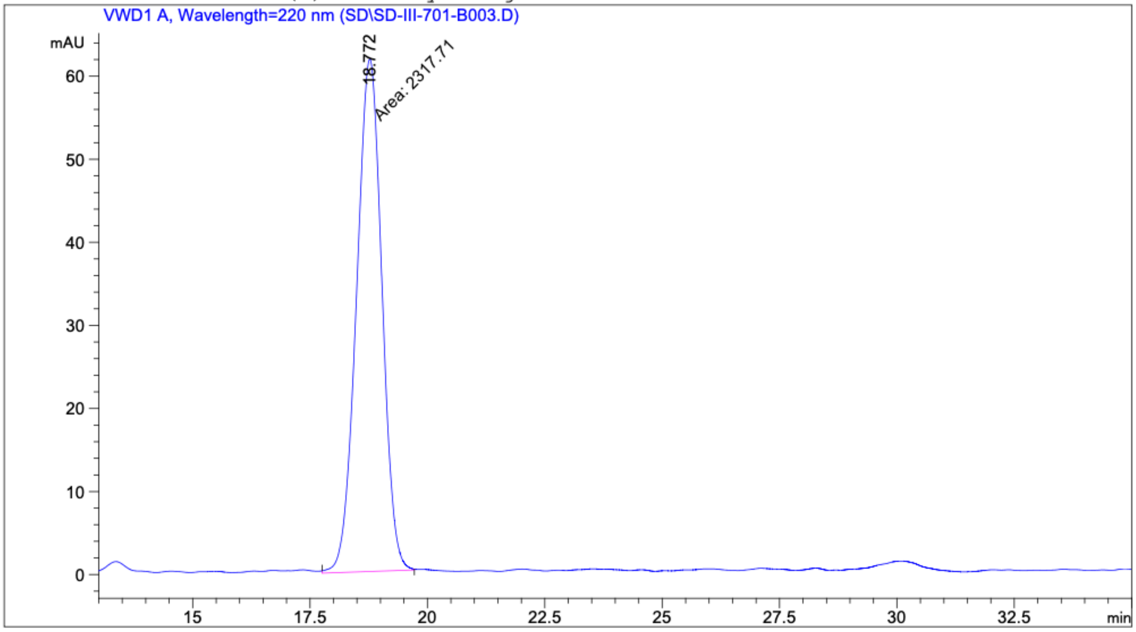


```
=====================================================================
                         Area Percent Report
=====================================================================

Sorted By             :      Signal
Multiplier:                  :      1.0000
Dilution:                    :      1.0000
Do not use Multiplier & Dilution Factor with ISTDs


Signal 1: VWD1 A, Wavelength=220 nm

Peak RetTime Type  Width     Area      Height     Area
  #   [min]        [min]   [mAU*s]     [mAU]       %
----|-------|----|-------|----------|----------|--------|
   1  18.772 MM    0.6262 2317.71216   61.68664 100.0000

Totals :                  2317.71216   61.68664
```

Racemic: (*rac-20*)

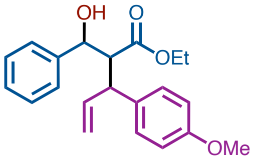


```
=====================================================================
Acq. Operator   : SYSTEM                        Seq. Line :   1
Acq. Instrument : 1220 HPLC                      Location : Vial 81
Injection Date  : 11/19/2024 5:30:57 PM               Inj :   1
                                               Inj Volume : 10.000 µl
Acq. Method     : C:\CHEM32\2\METHODS\MLC_VARIABLE.M
Last changed    : 11/19/2024 6:15:26 PM by SYSTEM
                  (modified after loading)
Analysis Method : C:\CHEM32\2\METHODS\DEF_LC.M
Last changed    : 4/26/2025 4:12:47 PM by SYSTEM
                  (modified after loading)
Sample Info     : C2; Hex:IPA 95:5 1.0 mL/min; 220nm


Additional Info : Peak(s) manually integrated
```

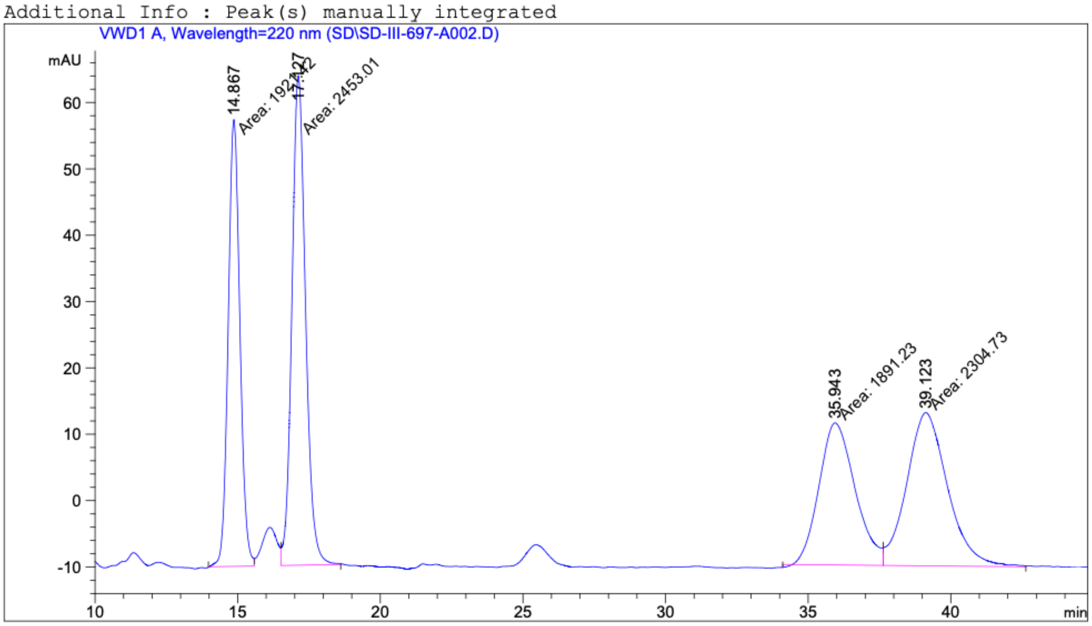


```
=====================================================================
                         Area Percent Report
=====================================================================

Sorted By             :      Signal
Multiplier:                   :      1.0000
Dilution:                     :      1.0000
Do not use Multiplier & Dilution Factor with ISTDs


Signal 1: VWD1 A, Wavelength=220 nm
```

Peak #	RetTime [min]	Type	Width [min]	Area [mAU*s]	Height [mAU]	Area %
1	14.867	MF	0.4757	1921.42200	67.32079	22.4193
2	17.127	FM	0.5542	2453.00830	73.77312	28.6219
3	35.943	MF	1.4719	1891.23401	21.41529	22.0671
4	39.123	FM	1.6630	2304.73193	23.09823	26.8918
Totals :				8570.39624	185.60743	

Enantioenriched: (*ent-20*)

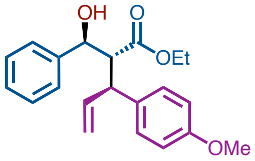


```
======================================================================
Acq. Operator   : SYSTEM                          Seq. Line :   1
Acq. Instrument : 1220 HPLC                        Location : Vial 81
Injection Date  : 11/21/2024 9:08:15 AM                 Inj :   1
                                                 Inj Volume : 5.000 µl
Acq. Method     : C:\CHEM32\2\METHODS\MLC_VARIABLE.M
Last changed    : 11/21/2024 9:02:41 AM by SYSTEM
                  (modified after loading)
Analysis Method : C:\CHEM32\2\METHODS\DEF_LC.M
Last changed    : 4/25/2025 4:56:49 PM by SYSTEM
                  (modified after loading)
Sample Info     : C-2; 95:5 Hex:IPA; 1 mL/min, 220 nm-


Additional Info : Peak(s) manually integrated
```

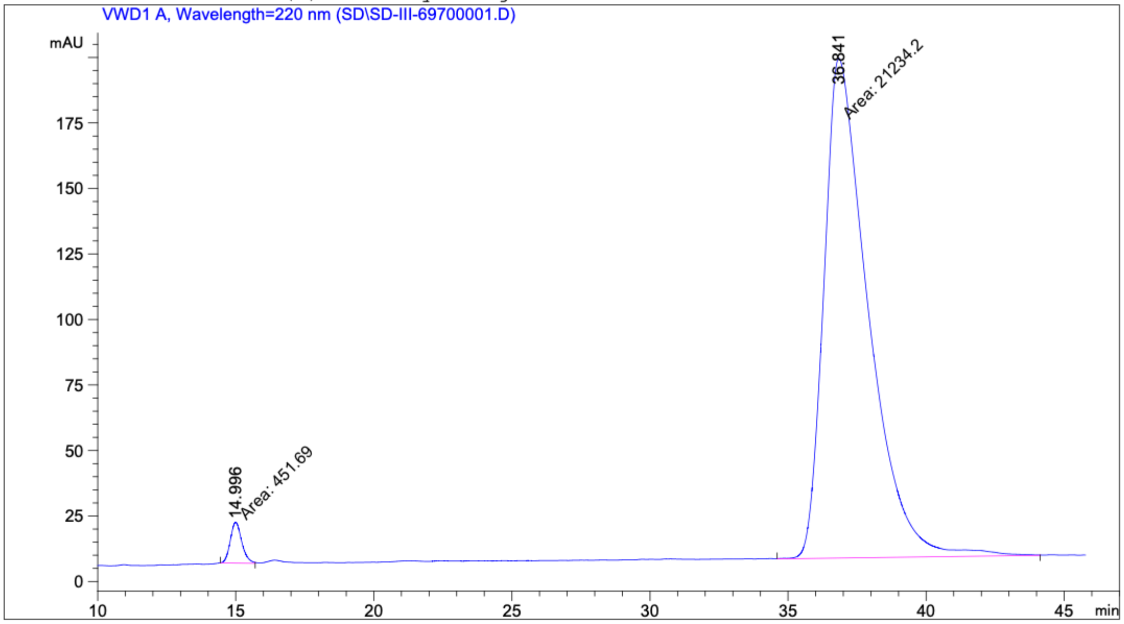


```
======================================================================
                          Area Percent Report
======================================================================

Sorted By             :      Signal
Multiplier:                  :      1.0000
Dilution:                    :      1.0000
Do not use Multiplier & Dilution Factor with ISTDs


Signal 1: VWD1 A, Wavelength=220 nm

Peak RetTime Type  Width     Area      Height     Area
  #   [min]        [min]   [mAU*s]     [mAU]        %
----|-------|----|-------|----------|----------|--------|
   1  14.996 MM    0.4828  451.69037   15.59284   2.0829
   2  36.841 MM    1.8560 2.12342e4  190.68488  97.9171

Totals :                  2.16859e4  206.27772
```

Racemic: (*rac-21*)

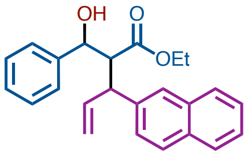


```
=====================================================================
Acq. Operator   : SYSTEM                          Seq. Line :   1
Acq. Instrument : 1220 HPLC                        Location : Vial 81
Injection Date  : 12/24/2024 11:10:08 AM                Inj :   1
                                                 Inj Volume : 10.000 µl
Acq. Method     : C:\CHEM32\2\METHODS\MLC_VARIABLE.M
Last changed    : 12/24/2024 11:05:19 AM by SYSTEM
Analysis Method : C:\CHEM32\2\METHODS\DEF_LC.M
Last changed    : 4/26/2025 4:13:46 PM by SYSTEM
                  (modified after loading)
Sample Info     : C-1_90:10 Hex:IPA; 1 mL/min, 220 nm

Additional Info : Peak(s) manually integrated
```

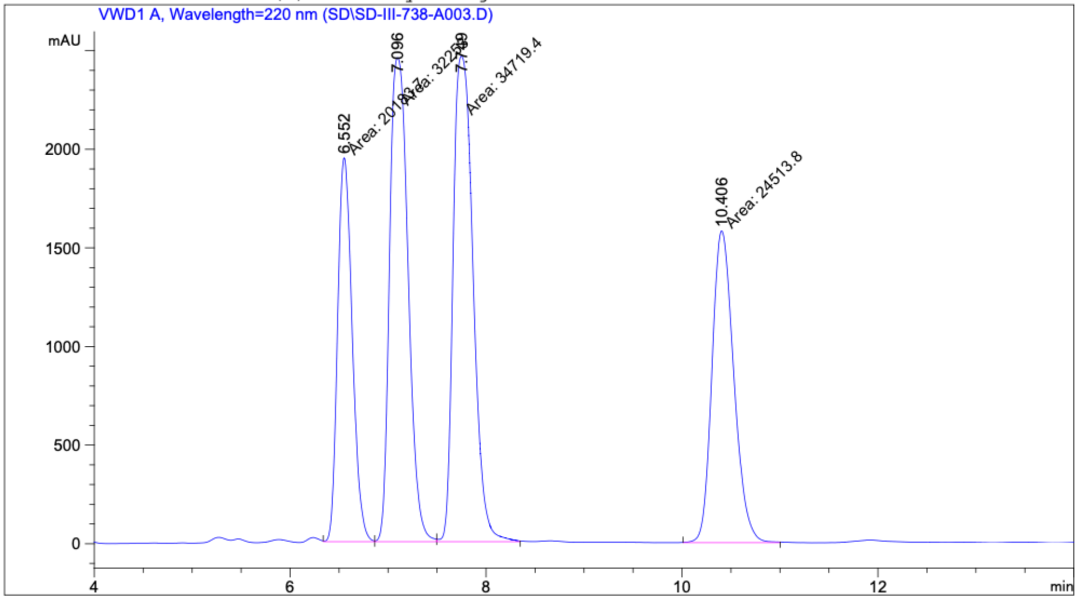


```
=====================================================================
                         Area Percent Report
=====================================================================

Sorted By             :      Signal
Multiplier:                  :      1.0000
Dilution:                    :      1.0000
Do not use Multiplier & Dilution Factor with ISTDs


Signal 1: VWD1 A, Wavelength=220 nm

Peak RetTime Type  Width     Area      Height     Area
  #   [min]        [min]   [mAU*s]     [mAU]        %
----|-------|----|-------|----------|----------|--------|
   1   6.552 MF    0.1728 2.01837e4  1946.61963  18.0744
   2   7.096 MF    0.2190 3.22530e4  2454.68726  28.8825
   3   7.749 FM    0.2350 3.47194e4  2462.19751  31.0911
   4  10.406 MM    0.2585 2.45138e4  1580.57471  21.9520

Totals :                  1.11670e5  8444.07910
```

Enantioenriched: (*ent-21*)

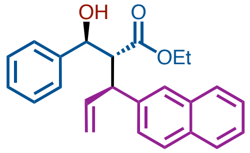


```
======================================================================
Acq. Operator   : SYSTEM                              Seq. Line :   2
Acq. Instrument : 1220 HPLC                            Location : Vial 71
Injection Date  : 12/24/2024 11:31:01 AM                    Inj :   1
                                                     Inj Volume : 10.000 µl
Acq. Method     : C:\CHEM32\2\METHODS\MLC_VARIABLE.M
Last changed    : 12/24/2024 11:05:19 AM by SYSTEM
Analysis Method : C:\CHEM32\2\METHODS\DEF_LC.M
Last changed    : 4/26/2025 4:14:58 PM by SYSTEM
                  (modified after loading)
Sample Info     : IA-3-95:5 Hex:IPA; 1 mL/min, 220 nm


Additional Info : Peak(s) manually integrated
```

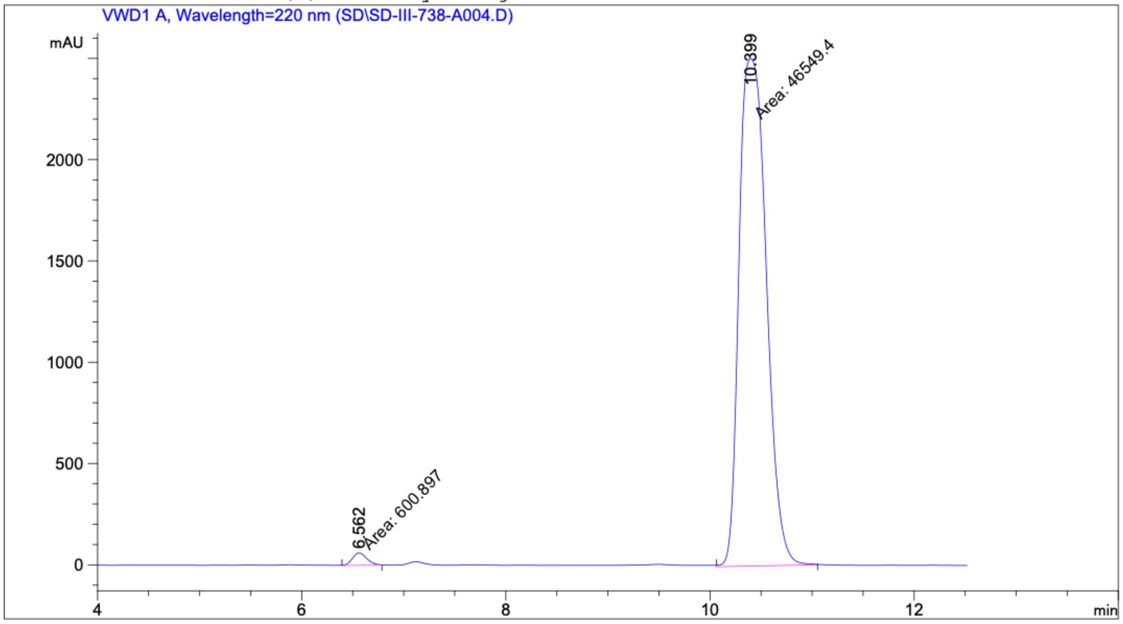


```
======================================================================
                         Area Percent Report
======================================================================

Sorted By             :      Signal
Multiplier:                    :      1.0000
Dilution:                      :      1.0000
Do not use Multiplier & Dilution Factor with ISTDs


Signal 1: VWD1 A, Wavelength=220 nm
```

Peak #	RetTime [min]	Type	Width [min]	Area [mAU*s]	Height [mAU]	Area %
1	6.562	MM	0.1633	600.89661	61.31010	1.2744
2	10.399	MM	0.3097	4.65494e4	2505.16797	98.7256

Totals : 4.71503e4 2566.47807

Racemic: (*rac-22*)

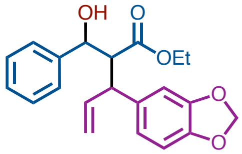


```
=====================================================================
Acq. Operator   : SYSTEM                          Seq. Line :    1
Acq. Instrument : 1220 HPLC                        Location : Vial 81
Injection Date  : 12/24/2024 3:08:25 PM                 Inj :    1
                                                 Inj Volume : 10.000 µl
Acq. Method     : C:\CHEM32\2\METHODS\MLC_VARIABLE.M
Last changed    : 12/24/2024 3:03:49 PM by SYSTEM
                  (modified after loading)
Analysis Method : C:\CHEM32\2\METHODS\DEF_LC.M
Last changed    : 4/26/2025 4:14:58 PM by SYSTEM
                  (modified after loading)
Sample Info     : C-2_90:10 Hex:IPA; 1 mL/min, 220 nm


Additional Info : Peak(s) manually integrated
```

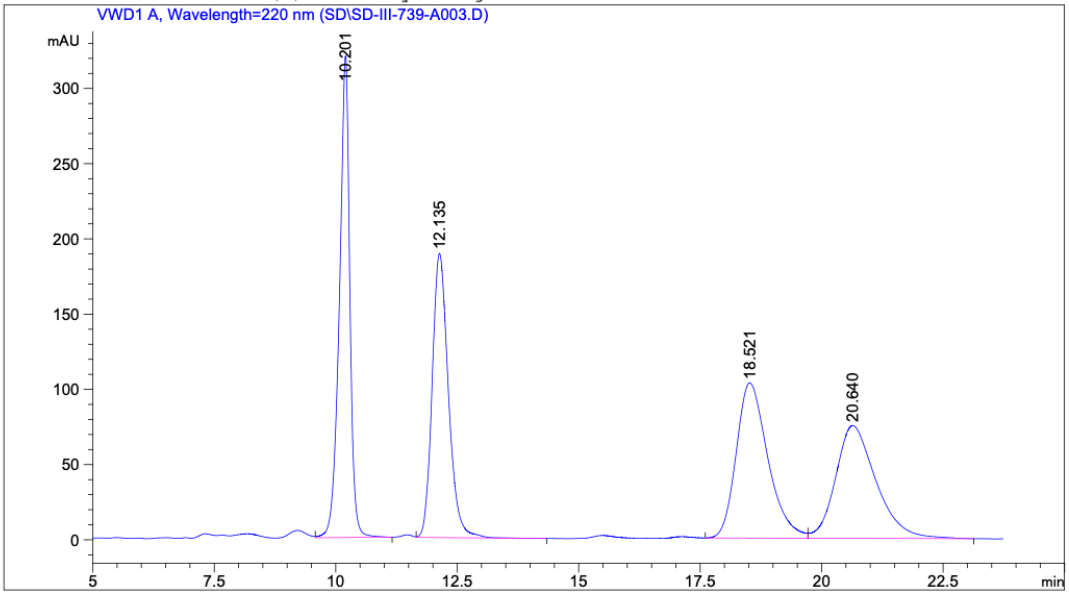


```
=====================================================================
                         Area Percent Report
=====================================================================

Sorted By             :      Signal
Multiplier:                   :      1.0000
Dilution:                     :      1.0000
Do not use Multiplier & Dilution Factor with ISTDs


Signal 1: VWD1 A, Wavelength=220 nm
```

Peak #	RetTime [min]	Type	Width [min]	Area [mAU*s]	Height [mAU]	Area %
1	10.201	VB	0.2157	4768.51660	320.19705	26.2779
2	12.135	VB	0.3547	4379.78662	188.77744	24.1357
3	18.521	BV	0.6899	4668.11963	103.16927	25.7246
4	20.640	VB	0.8679	4330.06689	74.95793	23.8617

Totals : 1.81465e4 687.10168

Enantioenriched: (*ent-22*)

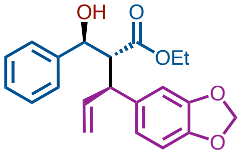


```
====================================================================
Acq. Operator   : SYSTEM                          Seq. Line :    1
Acq. Instrument : 1220 HPLC                        Location : Vial 71
Injection Date  : 12/24/2024 2:37:59 PM                 Inj :    1
                                                 Inj Volume : 10.000 µl
Acq. Method     : C:\CHEM32\2\METHODS\MLC_VARIABLE.M
Last changed    : 12/24/2024 3:00:09 PM by SYSTEM
                  (modified after loading)
Analysis Method : C:\CHEM32\2\METHODS\DEF_LC.M
Last changed    : 4/26/2025 4:16:52 PM by SYSTEM
                  (modified after loading)
Sample Info     : C-2_90:10 Hex:IPA; 1 mL/min, 220 nm


Additional Info : Peak(s) manually integrated
```

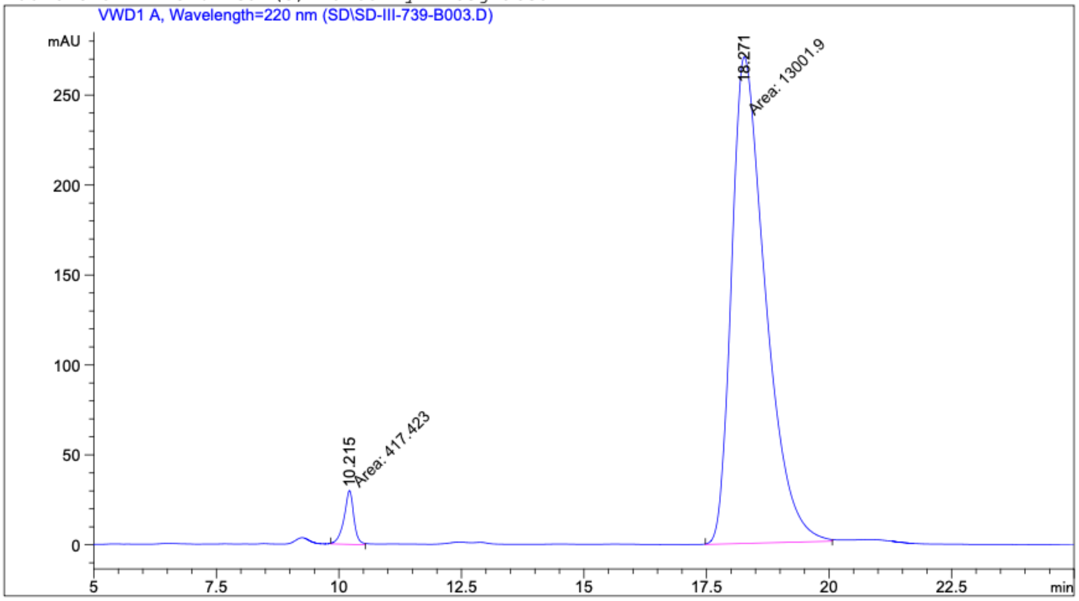


```
====================================================================
                         Area Percent Report
====================================================================

Sorted By             :      Signal
Multiplier:                  :      1.0000
Dilution:                    :      1.0000
Do not use Multiplier & Dilution Factor with ISTDs


Signal 1: VWD1 A, Wavelength=220 nm
```

Peak #	RetTime [min]	Type	Width [min]	Area [mAU*s]	Height [mAU]	Area %
1	10.215	MM	0.2326	417.42270	29.91023	3.1106
2	18.271	MM	0.8012	1.30019e4	270.46631	96.8894

Totals : 1.34193e4 300.37654

Racemic: (*rac-23*)

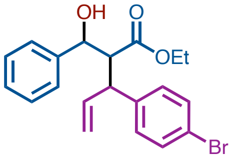


```
=====================================================================
Acq. Operator   : SYSTEM                          Seq. Line :    1
Acq. Instrument : 1220 HPLC                        Location : Vial 81
Injection Date  : 12/23/2024 1:10:44 PM                 Inj :    1
                                                 Inj Volume : 10.000 µl
Acq. Method     : C:\CHEM32\2\METHODS\MLC_VARIABLE.M
Last changed    : 12/23/2024 1:22:43 PM by SYSTEM
                  (modified after loading)
Analysis Method : C:\CHEM32\2\METHODS\DEF_LC.M
Last changed    : 4/26/2025 4:16:52 PM by SYSTEM
                  (modified after loading)
Sample Info     : C1-95:5 Hex:IPA; 0.5 mL/min, 220 nm

Additional Info : Peak(s) manually integrated
```

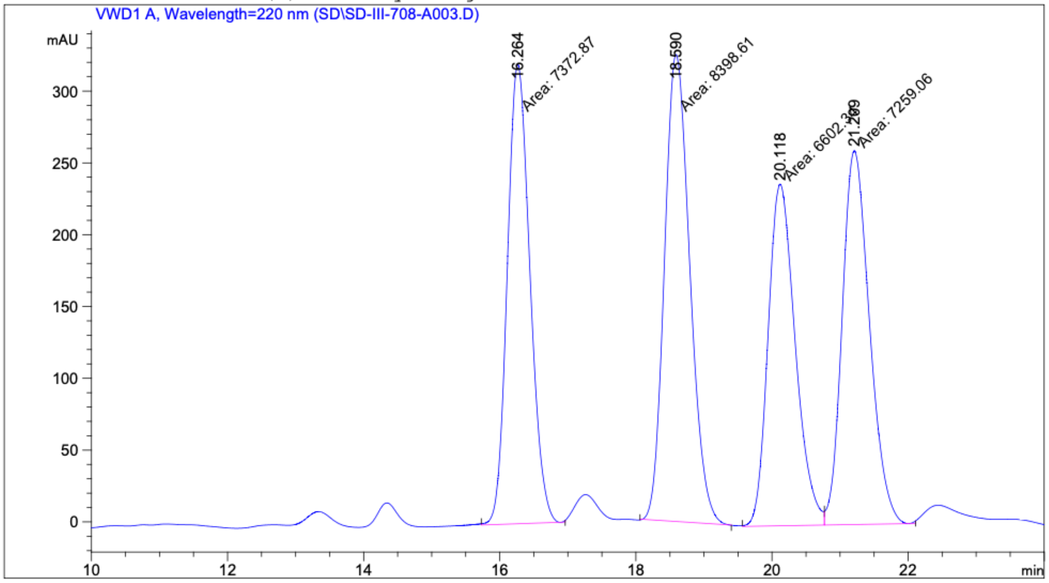


```
=====================================================================
                        Area Percent Report
=====================================================================

Sorted By             :      Signal
Multiplier:                  :      1.0000
Dilution:                    :      1.0000
Do not use Multiplier & Dilution Factor with ISTDs


Signal 1: VWD1 A, Wavelength=220 nm
```

Peak #	RetTime [min]	Type	Width [min]	Area [mAU*s]	Height [mAU]	Area %
1	16.264	MM	0.3843	7372.86914	319.74551	24.8807
2	18.590	MM	0.4306	8398.61230	325.08893	28.3422
3	20.118	MF	0.4626	6602.34229	237.88258	22.2805
4	21.209	FM	0.4649	7259.06152	260.24527	24.4966

Totals : 2.96329e4 1142.96230

Enantioenriched: (*int-23*)

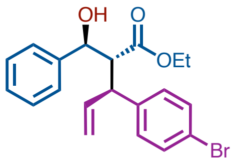


```
=====================================================================
Acq. Operator   : SYSTEM                          Seq. Line :    1
Acq. Instrument : 1220 HPLC                        Location : Vial 71
Injection Date  : 12/23/2024 1:48:03 PM                 Inj :    1
                                                 Inj Volume : 20.000 µl
Acq. Method     : C:\CHEM32\2\METHODS\MLC_VARIABLE.M
Last changed    : 12/23/2024 1:43:24 PM by SYSTEM
                  (modified after loading)
Analysis Method : C:\CHEM32\2\METHODS\DEF_LC.M
Last changed    : 4/29/2025 1:51:13 PM by SYSTEM
                  (modified after loading)
Sample Info     : C1-95:5 Hex:IPA; 0.5 mL/min, 220 nm


Additional Info : Peak(s) manually integrated
```

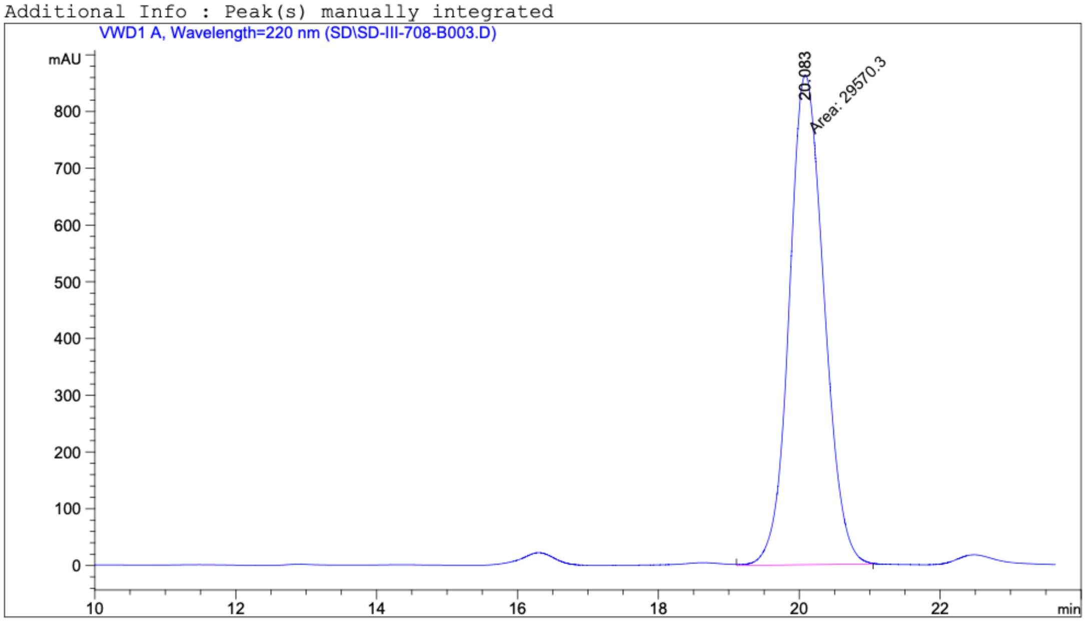


```
=====================================================================
                         Area Percent Report
=====================================================================

Sorted By             :      Signal
Multiplier:                   :       1.0000
Dilution:                     :       1.0000
Do not use Multiplier & Dilution Factor with ISTDs


Signal 1: VWD1 A, Wavelength=220 nm
```

Peak #	RetTime [min]	Type	Width [min]	Area [mAU*s]	Height [mAU]	Area %
1	20.083	MM	0.5710	2.95703e4	863.17908	100.0000

Totals : 2.95703e4 863.17908

Racemic: (*rac-24*)

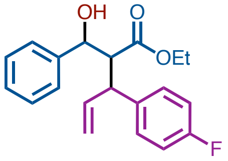


```
====================================================================
Acq. Operator   : SYSTEM                          Seq. Line :   1
Acq. Instrument : 1220 HPLC                        Location : Vial 81
Injection Date  : 11/20/2024 2:24:50 PM                 Inj :   1
                                                 Inj Volume : 50.000 µl
Acq. Method     : C:\CHEM32\2\METHODS\MLC_VARIABLE.M
Last changed    : 11/20/2024 2:53:33 PM by SYSTEM
                  (modified after loading)
Analysis Method : C:\CHEM32\2\METHODS\DEF_LC.M
Last changed    : 7/19/2025 11:32:57 AM by SYSTEM
                  (modified after loading)
Sample Info     : IA-3; Hex:IPA 95:5 1.0 mL/min; 220nm

Additional Info : Peak(s) manually integrated
```

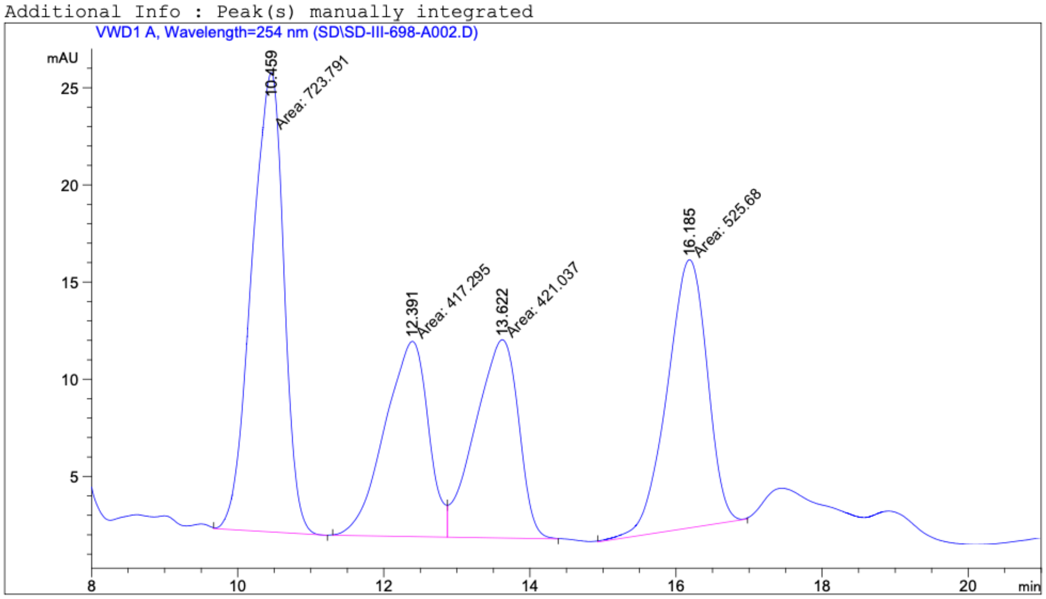


```
====================================================================
                         Area Percent Report
====================================================================

Sorted By             :      Signal
Multiplier:                  :      1.0000
Dilution:                    :      1.0000
Do not use Multiplier & Dilution Factor with ISTDs


Signal 1: VWD1 A, Wavelength=254 nm
```

Peak #	RetTime [min]	Type	Width [min]	Area [mAU*s]	Height [mAU]	Area %
1	10.459	MM	0.5105	723.79120	23.63156	34.6676
2	12.391	MF	0.6927	417.29507	10.04025	19.9873
3	13.622	FM	0.6877	421.03741	10.20349	20.1665
4	16.185	MM	0.6352	525.67981	13.79319	25.1786

Totals : 2087.80350 57.66849

Enantioenriched: (*ent-24*)

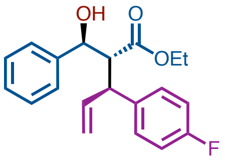


```
====================================================================
Acq. Operator   : SYSTEM                          Seq. Line :   1
Acq. Instrument : 1220 HPLC                        Location : Vial 71
Injection Date  : 11/20/2024 9:30:21 AM                 Inj :   1
                                                 Inj Volume : 20.000 µl
Acq. Method     : C:\CHEM32\2\METHODS\MLC_VARIABLE.M
Last changed    : 11/20/2024 9:26:45 AM by SYSTEM
                  (modified after loading)
Analysis Method : C:\CHEM32\2\METHODS\DEF_LC.M
Last changed    : 7/19/2025 11:37:02 AM by SYSTEM
                  (modified after loading)
Sample Info     : IA-3; Hex:IPA 95:5 1.0 mL/min; 220nm


Additional Info : Peak(s) manually integrated
```

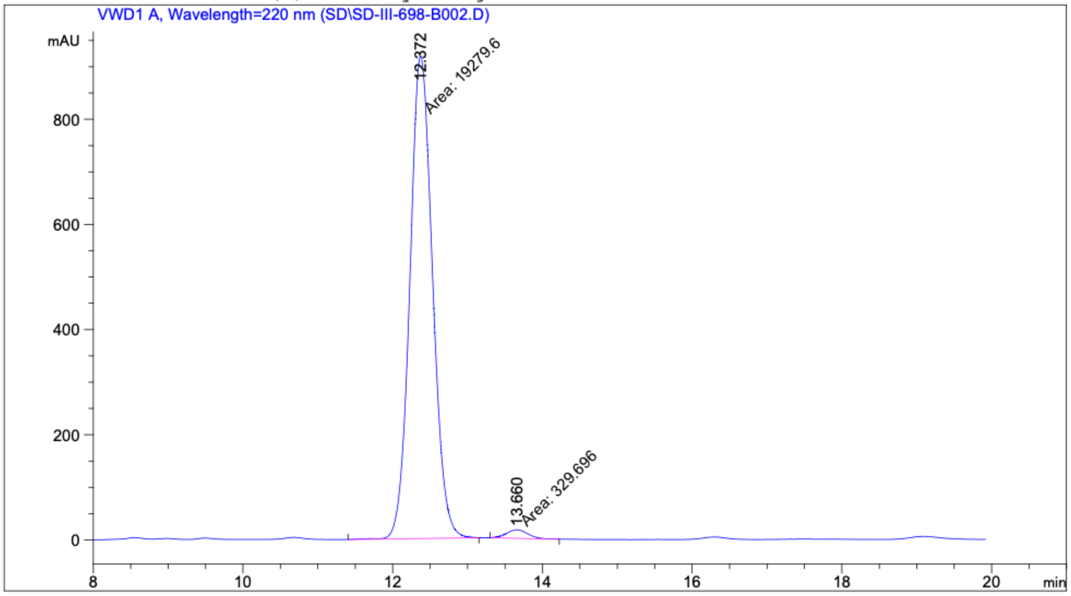


```
====================================================================
                         Area Percent Report
====================================================================

Sorted By             :      Signal
Multiplier:                  :      1.0000
Dilution:                    :      1.0000
Do not use Multiplier & Dilution Factor with ISTDs


Signal 1: VWD1 A, Wavelength=220 nm
```

Peak #	RetTime [min]	Type	Width [min]	Area [mAU*s]	Height [mAU]	Area %
1	12.372	MM	0.3500	1.92796e4	917.95935	98.3187
2	13.660	MM	0.3365	329.69568	16.33081	1.6813

Totals : 1.96093e4 934.29016

Racemic: (*rac-25*)

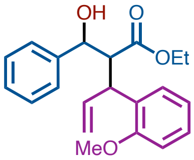


```
=====================================================================
Acq. Operator   : SYSTEM                          Seq. Line :    1
Acq. Instrument : 1220 HPLC                        Location : Vial 11
Injection Date  : 12/20/2024 2:48:53 PM                 Inj :    1
                                                Inj Volume : 10.000 µl
Acq. Method     : C:\CHEM32\2\METHODS\MLC_VARIABLE.M
Last changed    : 12/20/2024 3:06:49 PM by SYSTEM
                  (modified after loading)
Analysis Method : C:\CHEM32\2\METHODS\DEF_LC.M
Last changed    : 4/29/2025 1:51:13 PM by SYSTEM
                  (modified after loading)
Sample Info     : C-1- 95:5 Hex:IPA; 1 mL/min, 220 nm


Additional Info : Peak(s) manually integrated
```

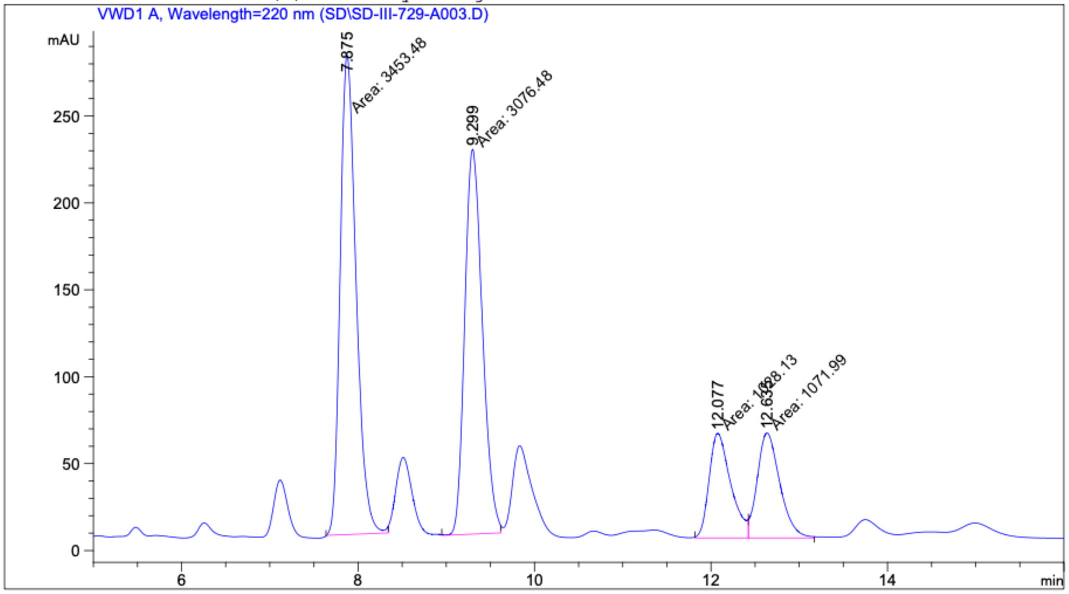


```
=====================================================================
                         Area Percent Report
=====================================================================

Sorted By             :      Signal
Multiplier:                  :      1.0000
Dilution:                    :      1.0000
Do not use Multiplier & Dilution Factor with ISTDs


Signal 1: VWD1 A, Wavelength=220 nm
```

Peak #	RetTime [min]	Type	Width [min]	Area [mAU*s]	Height [mAU]	Area %
1	7.875	MM	0.2088	3453.47827	275.65707	40.0168
2	9.299	MM	0.2317	3076.48071	221.28960	35.6483
3	12.077	MF	0.2850	1028.13281	60.11892	11.9134
4	12.635	FM	0.2957	1071.98767	60.42855	12.4215

Totals : 8630.07947 617.49413

Enantioenriched: (*ent*-25)

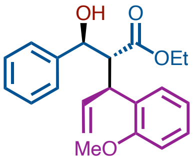


```
=====================================================================
Acq. Operator   : SYSTEM                              Seq. Line :    1
Acq. Instrument : 1220 HPLC                            Location : Vial 21
Injection Date  : 12/20/2024 3:13:19 PM                     Inj :    1
                                                    Inj Volume : 10.000 µl
Acq. Method     : C:\CHEM32\2\METHODS\MLC_VARIABLE.M
Last changed    : 12/20/2024 3:09:30 PM by SYSTEM
Analysis Method : C:\CHEM32\2\METHODS\DEF_LC.M
Last changed    : 4/29/2025 1:53:29 PM by SYSTEM
                  (modified after loading)
Sample Info     : C-1- 95:5 Hex:IPA; 1 mL/min, 220 nm


Additional Info : Peak(s) manually integrated
```

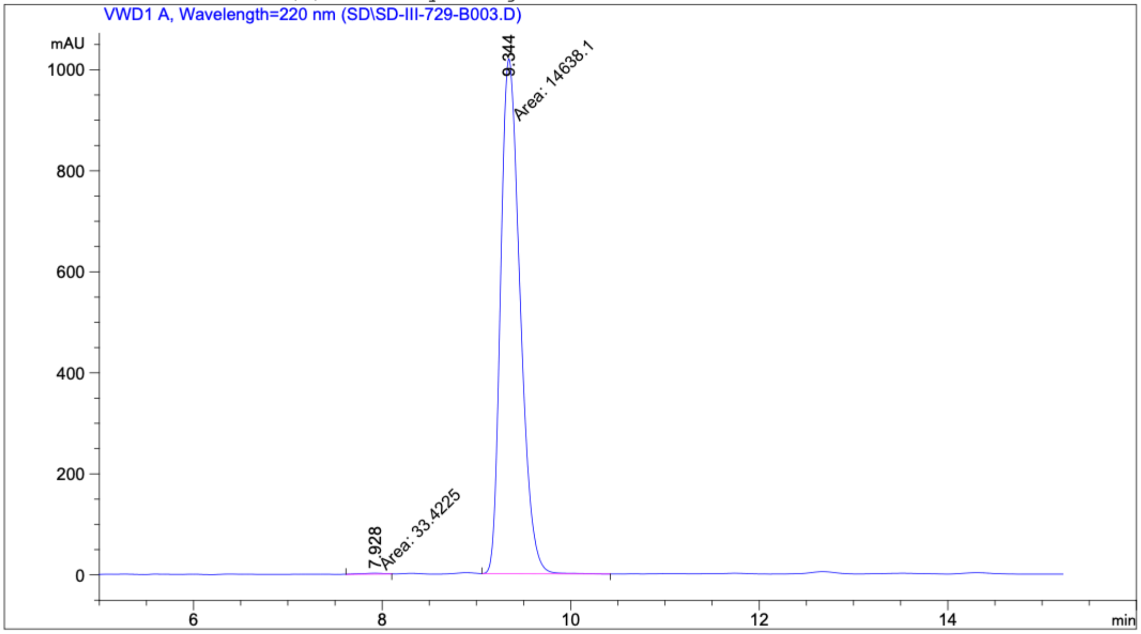


```
=====================================================================
                         Area Percent Report
=====================================================================

Sorted By             :      Signal
Multiplier:                   :      1.0000
Dilution:                     :      1.0000
Do not use Multiplier & Dilution Factor with ISTDs


Signal 1: VWD1 A, Wavelength=220 nm

Peak RetTime Type  Width     Area      Height     Area
  #   [min]        [min]   [mAU*s]     [mAU]        %
----|-------|----|-------|----------|----------|--------|
   1   7.928 MM    0.2925   33.42254    1.90469   0.2278
   2   9.344 MM    0.2394 1.46381e4 1019.12213  99.7722

Totals :                  1.46715e4  1021.02682
```

Racemic: (*rac-26*)

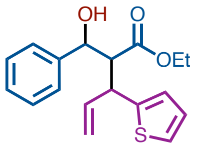

===

Acq. Operator : SYSTEM Seq. Line : 1
Acq. Instrument : 1220 HPLC Location : Vial 81
Injection Date : 8/3/2025 4:54:57 PM Inj : 1
Inj Volume : 10.000 µl
Acq. Method : C:\CHEM32\2\METHODS\MLC_VARIABLE.M
Last changed : 8/3/2025 5:35:43 PM by SYSTEM
(modified after loading)
Analysis Method : C:\CHEM32\2\METHODS\DEF_LC.M
Last changed : 7/19/2025 11:43:49 AM by SYSTEM
(modified after loading)
Sample Info : C1_98:2 IPA:Hex,0.5 ml/min, 220 nm, 15min

Additional Info : Peak(s) manually integrated

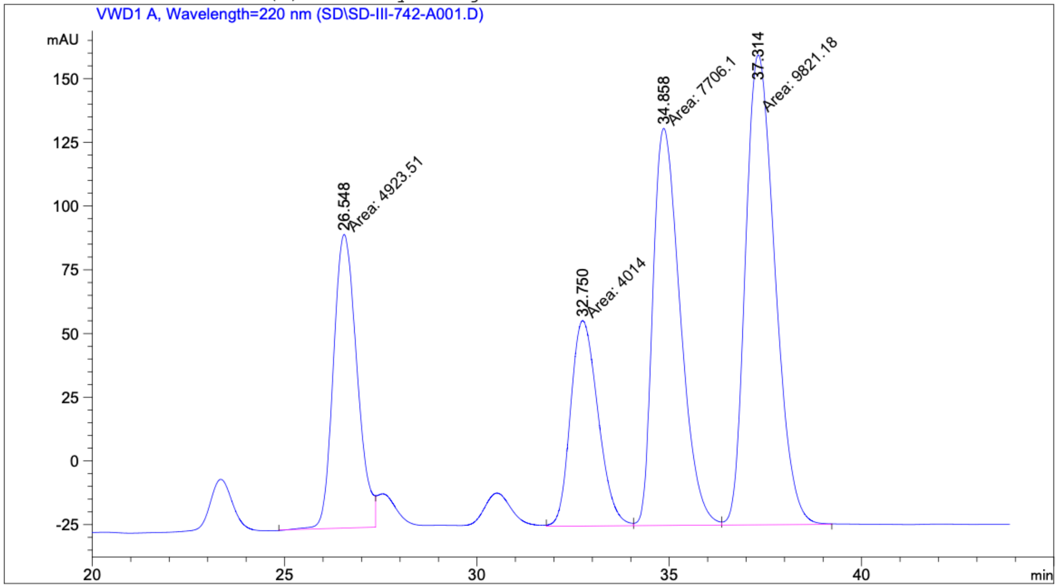

===

Area Percent Report

===

Sorted By : Signal
Multiplier: : 1.0000
Dilution: : 1.0000
Do not use Multiplier & Dilution Factor with ISTDs

Signal 1: VWD1 A, Wavelength=220 nm

Peak #	RetTime [min]	Type	Width [min]	Area [mAU*s]	Height [mAU]	Area %
1	26.548	MF	0.7124	4923.50830	115.17970	18.6040
2	32.750	MF	0.8303	4013.99951	80.57648	15.1673
3	34.858	FM	0.8242	7706.10107	155.82347	29.1183
4	37.314	FM	0.8878	9821.17871	184.37320	37.1104

Totals : 2.64648e4 535.95285

Enantioenriched: (*ent-26*)

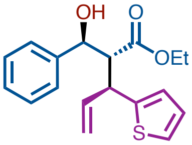


```
====================================================================
Acq. Operator   : SYSTEM                          Seq. Line :    1
Acq. Instrument : 1220 HPLC                        Location : Vial 71
Injection Date  : 8/3/2025 5:51:21 PM                   Inj :    1
                                                 Inj Volume : 5.000 µl
Acq. Method     : C:\CHEM32\2\METHODS\MLC_VARIABLE.M
Last changed    : 8/3/2025 5:48:58 PM by SYSTEM
                  (modified after loading)
Analysis Method : C:\CHEM32\2\METHODS\DEF_LC.M
Last changed    : 8/3/2025 6:39:54 PM by SYSTEM
                  (modified after loading)
Sample Info     : C1_98:2 IPA:Hex,0.5 ml/min, 220 nm, 15min


Additional Info : Peak(s) manually integrated
```

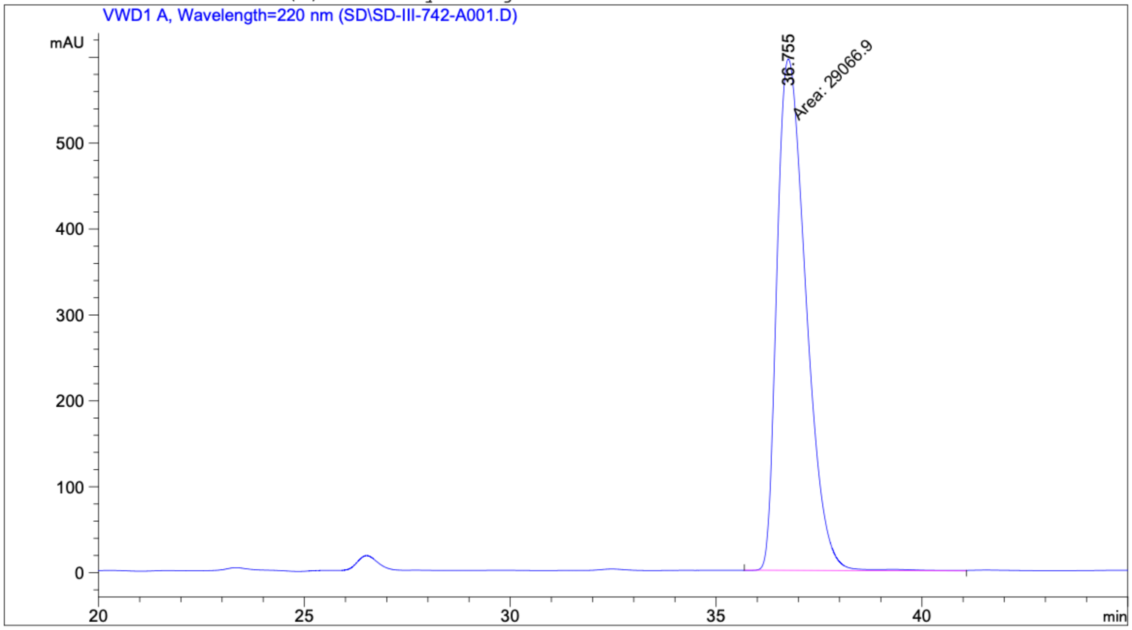


```
====================================================================
                         Area Percent Report
====================================================================

Sorted By             :      Signal
Multiplier:                   :      1.0000
Dilution:                     :      1.0000
Do not use Multiplier & Dilution Factor with ISTDs


Signal 1: VWD1 A, Wavelength=220 nm

Peak RetTime Type  Width     Area      Height     Area
  #   [min]        [min]   [mAU*s]     [mAU]        %
----|-------|----|-------|----------|----------|--------|
   1  36.755 MM    0.8139 2.90669e4   595.24689 100.0000

Totals :                  2.90669e4   595.24689
```

Racemic: (*rac-27*)

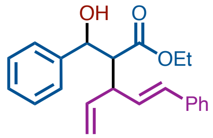


```
====================================================================
Acq. Operator   : SYSTEM                        Seq. Line :   1
Acq. Instrument : 1220 HPLC                      Location : Vial 71
Injection Date  : 1/11/2025 11:38:42 AM               Inj :   1
                                               Inj Volume : 10.000 µl
Acq. Method     : C:\CHEM32\2\METHODS\MLC_VARIABLE.M
Last changed    : 1/11/2025 11:03:07 AM by SYSTEM
Analysis Method : C:\CHEM32\2\METHODS\DEF_LC.M
Last changed    : 4/29/2025 1:57:35 PM by SYSTEM
                  (modified after loading)
Sample Info     : C1_95:5  Hex:IPA; 1mL/min, 220 nm


Additional Info : Peak(s) manually integrated
```

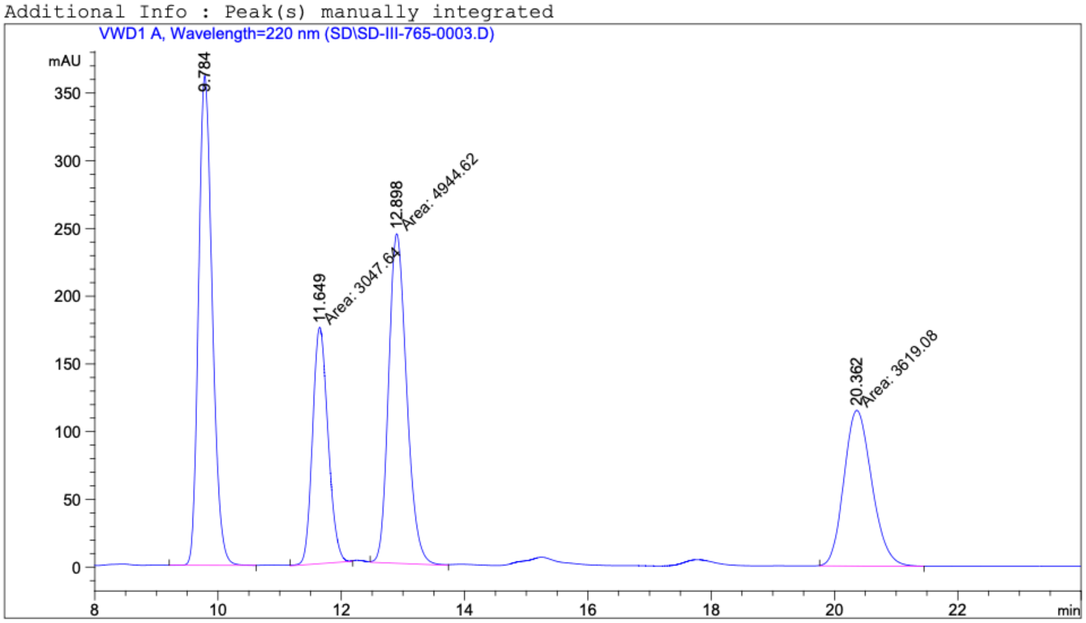


```
====================================================================
                         Area Percent Report
====================================================================

Sorted By             :      Signal
Multiplier:                   :       1.0000
Dilution:                     :       1.0000
Do not use Multiplier & Dilution Factor with ISTDs


Signal 1: VWD1 A, Wavelength=220 nm

Peak RetTime Type  Width     Area      Height     Area
  #   [min]        [min]   [mAU*s]     [mAU]        %
----|-------|----|-------|----------|----------|--------|
   1   9.784 BB    0.2408 5601.21484  361.39639  32.5415
   2  11.649 MM    0.2911 3047.63696  174.49150  17.7059
   3  12.898 MM    0.3388 4944.62402  243.22101  28.7269
   4  20.362 MM    0.5248 3619.07568  114.94546  21.0258

Totals :                  1.72126e4   894.05436
```

Enantioenriched: (*ent-27*)

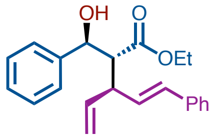


```
======================================================================
Acq. Operator   : SYSTEM                              Seq. Line :   2
Acq. Instrument : 1220 HPLC                            Location : Vial 81
Injection Date  : 1/11/2025 10:36:47 AM                     Inj :   1
                                                     Inj Volume : 10.000 µl
Acq. Method     : C:\CHEM32\2\METHODS\MLC_VARIABLE.M
Last changed    : 1/11/2025 10:06:16 AM by SYSTEM
                  (modified after loading)
Analysis Method : C:\CHEM32\2\METHODS\DEF_LC.M
Last changed    : 4/29/2025 1:53:29 PM by SYSTEM
                  (modified after loading)
Sample Info     : C1_95:5  Hex:IPA; 1mL/min, 220 nm


Additional Info : Peak(s) manually integrated
```

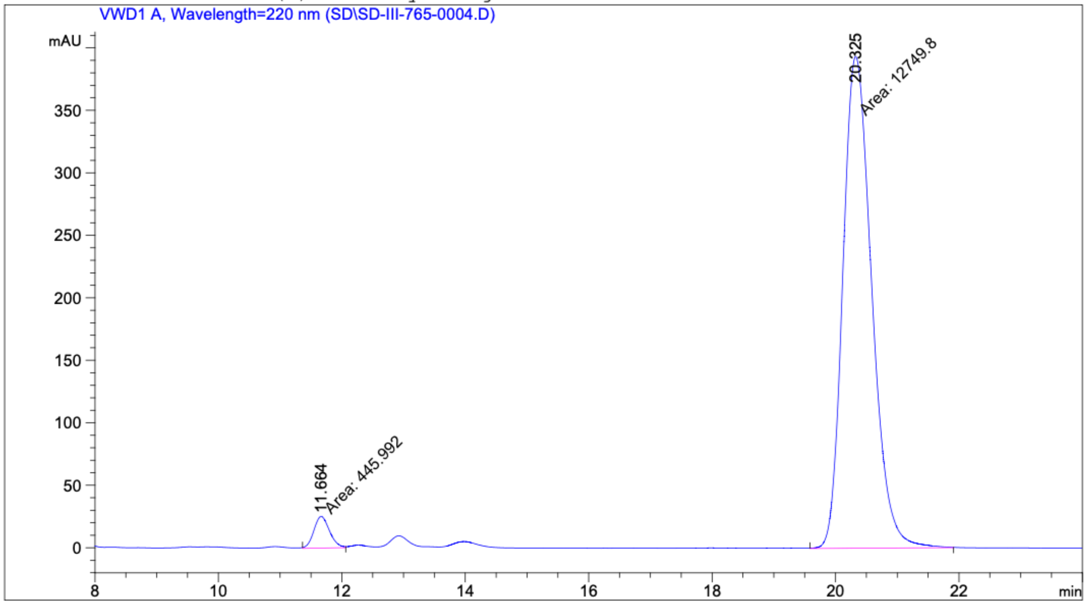


```
======================================================================
                         Area Percent Report
======================================================================

Sorted By             :      Signal
Multiplier:                   :      1.0000
Dilution:                     :      1.0000
Do not use Multiplier & Dilution Factor with ISTDs


Signal 1: VWD1 A, Wavelength=220 nm

Peak RetTime Type  Width     Area      Height     Area
  #   [min]        [min]   [mAU*s]     [mAU]        %
----|-------|----|-------|----------|----------|--------|
   1  11.664 MM    0.2946  445.99182   25.23156   3.3798
   2  20.325 MM    0.5402 1.27498e4  393.39120  96.6202

Totals :                  1.31958e4  418.62277
```

Racemic: (*rac-28*)

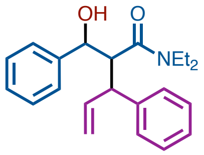


```
======================================================================
Acq. Operator   : SYSTEM                          Seq. Line :    1
Acq. Instrument : 1220 HPLC                        Location : Vial 71
Injection Date  : 1/3/2025 7:25:24 PM                   Inj :    1
                                                 Inj Volume : 10.000 µl
Acq. Method     : C:\CHEM32\2\METHODS\MLC_VARIABLE.M
Last changed    : 1/3/2025 7:21:34 PM by SYSTEM
Analysis Method : C:\CHEM32\2\METHODS\DEF_LC.M
Last changed    : 4/29/2025 2:01:51 PM by SYSTEM
                  (modified after loading)
Sample Info     : C-1_90:10  Hex:IPA; 1 mL/min, 220 nm

Additional Info : Peak(s) manually integrated
```

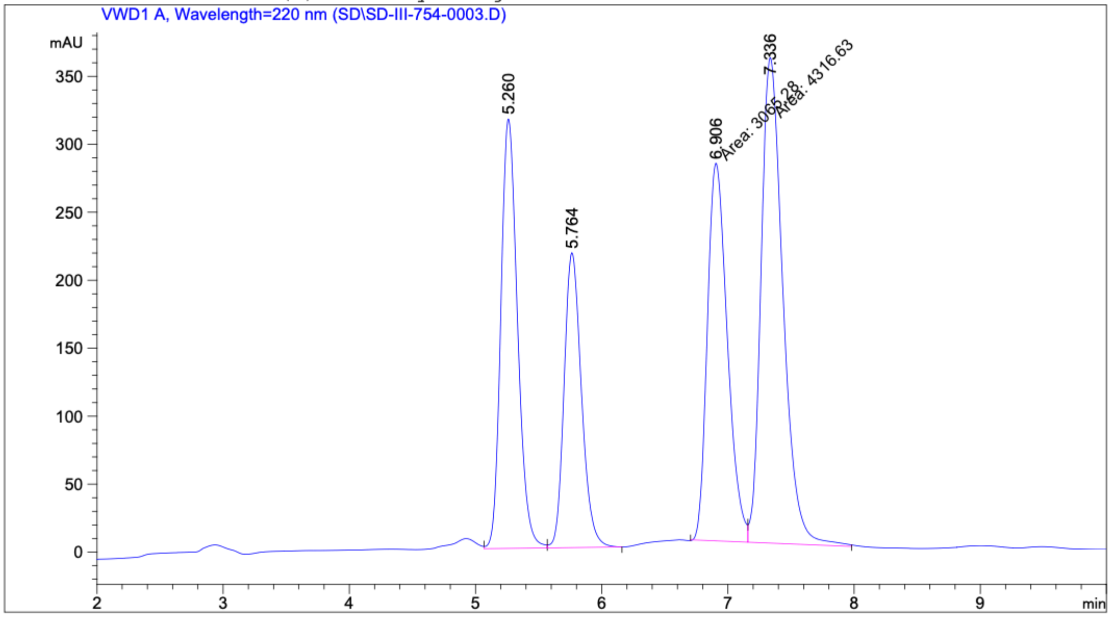


```
======================================================================
                        Area Percent Report
======================================================================

Sorted By             :      Signal
Multiplier:                   :      1.0000
Dilution:                     :      1.0000
Do not use Multiplier & Dilution Factor with ISTDs


Signal 1: VWD1 A, Wavelength=220 nm
```

Peak #	RetTime [min]	Type	Width [min]	Area [mAU*s]	Height [mAU]	Area %
1	5.260	VV	0.1418	2890.62939	315.85153	23.4096
2	5.764	VB	0.1477	2075.49170	216.84827	16.8083
3	6.906	MF	0.1839	3065.28467	277.73727	24.8241
4	7.336	FM	0.2015	4316.63330	357.03165	34.9580

Totals : 1.23480e4 1167.46872

Enantioenriched: (*ent-28*)

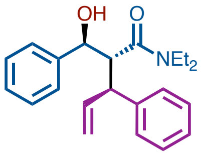


```
======================================================================
Acq. Operator   : SYSTEM                          Seq. Line :   2
Acq. Instrument : 1220 HPLC                        Location : Vial 81
Injection Date  : 1/3/2025 7:41:17 PM                   Inj :   1
                                                 Inj Volume : 10.000 µl
Acq. Method     : C:\CHEM32\2\METHODS\MLC_VARIABLE.M
Last changed    : 1/3/2025 7:21:34 PM by SYSTEM
Analysis Method : C:\CHEM32\2\METHODS\DEF_LC.M
Last changed    : 4/29/2025 2:01:51 PM by SYSTEM
                  (modified after loading)
Sample Info     : C-3_95:5  Hex:IPA; 1 mL/min, 220 nm

Additional Info : Peak(s) manually integrated
```

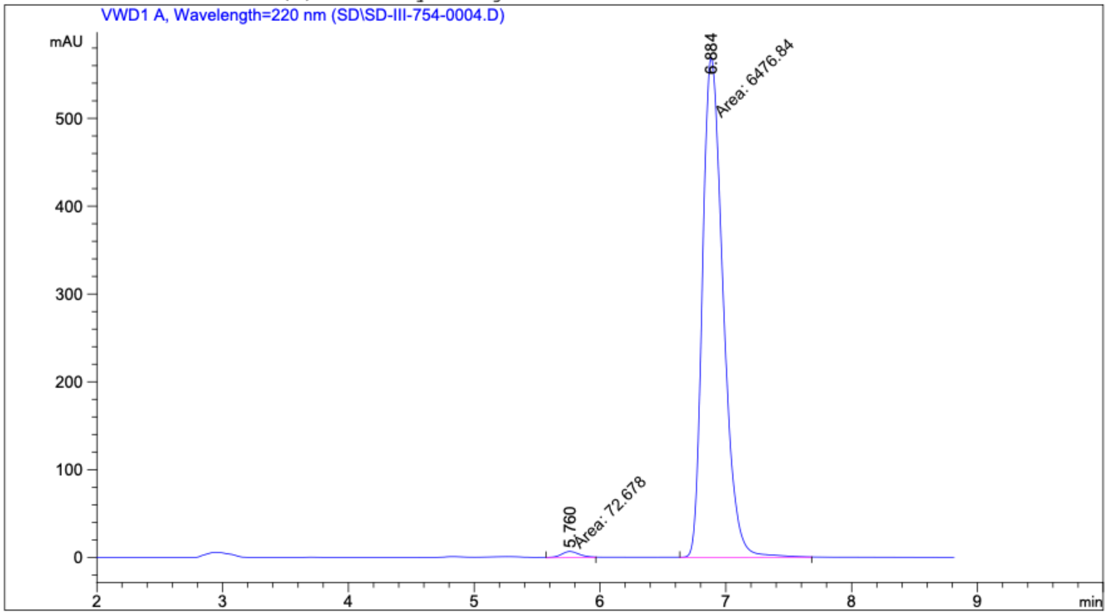


```
======================================================================
                        Area Percent Report
======================================================================

Sorted By             :      Signal
Multiplier:                   :      1.0000
Dilution:                     :      1.0000
Do not use Multiplier & Dilution Factor with ISTDs


Signal 1: VWD1 A, Wavelength=220 nm

Peak RetTime Type  Width     Area      Height     Area
  #   [min]        [min]   [mAU*s]     [mAU]        %
----|-------|----|-------|----------|----------|--------|
   1   5.760 MM    0.1722   72.67798    7.03384   1.1097
   2   6.884 MM    0.1896 6476.83936  569.21240  98.8903

Totals :                  6549.51733  576.24624
```

Racemic: (*rac-40*)

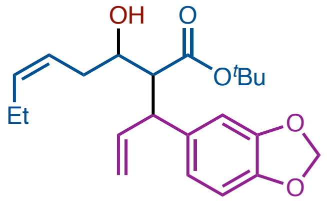


```
=====================================================================
Acq. Operator   : SYSTEM                          Seq. Line :    1
Acq. Instrument : 1220 HPLC                        Location : Vial 81
Injection Date  : 9/25/2025 9:38:04 AM                  Inj :    1
                                                 Inj Volume : 5.000 µl
Acq. Method     : C:\CHEM32\2\METHODS\MLC_VARIABLE.M
Last changed    : 9/25/2025 10:14:16 AM by SYSTEM
                  (modified after loading)
Analysis Method : C:\CHEM32\2\METHODS\DEF_LC.M
Last changed    : 9/21/2025 5:54:45 PM by SYSTEM
                  (modified after loading)
Sample Info     : C1_HEX:IPA_98:2_0.5 mL/min_220nm


Additional Info : Peak(s) manually integrated
```

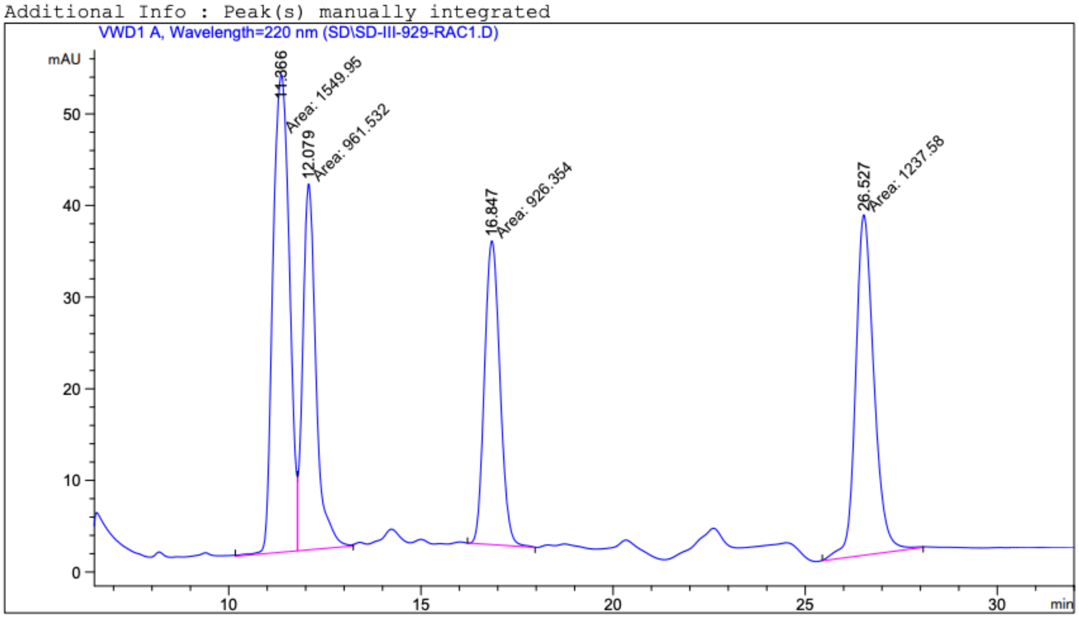


```
=====================================================================
                         Area Percent Report
=====================================================================

Sorted By             :      Signal
Multiplier:                   :       1.0000
Dilution:                     :       1.0000
Do not use Multiplier & Dilution Factor with ISTDs


Signal 1: VWD1 A, Wavelength=220 nm
```

Peak #	RetTime [min]	Type	Width [min]	Area [mAU*s]	Height [mAU]	Area %
1	11.366	MF	0.4955	1549.95178	52.13892	33.1511
2	12.079	FM	0.4013	961.53247	39.93679	20.5657
3	16.847	MM	0.4659	926.35370	33.13874	19.8133
4	26.527	MM	0.5551	1237.58228	37.15572	26.4700
Totals :				4675.42023	162.37016	

Enantioenriched: (*ent-40*)

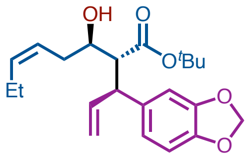


```
=====================================================================
Acq. Operator   : SYSTEM                        Seq. Line :   1
Acq. Instrument : 1220 HPLC                      Location : Vial 71
Injection Date  : 9/25/2025 10:19:45 AM               Inj :   1
                                               Inj Volume : 5.000 µl
Acq. Method     : C:\CHEM32\2\METHODS\MLC_VARIABLE.M
Last changed    : 9/25/2025 10:14:58 AM by SYSTEM
Analysis Method : C:\CHEM32\2\METHODS\DEF_LC.M
Last changed    : 9/25/2025 10:16:13 AM by SYSTEM
                  (modified after loading)
Sample Info     : C1_HEX:IPA_98:2_0.5 mL/min_220nm

Additional Info : Peak(s) manually integrated
```

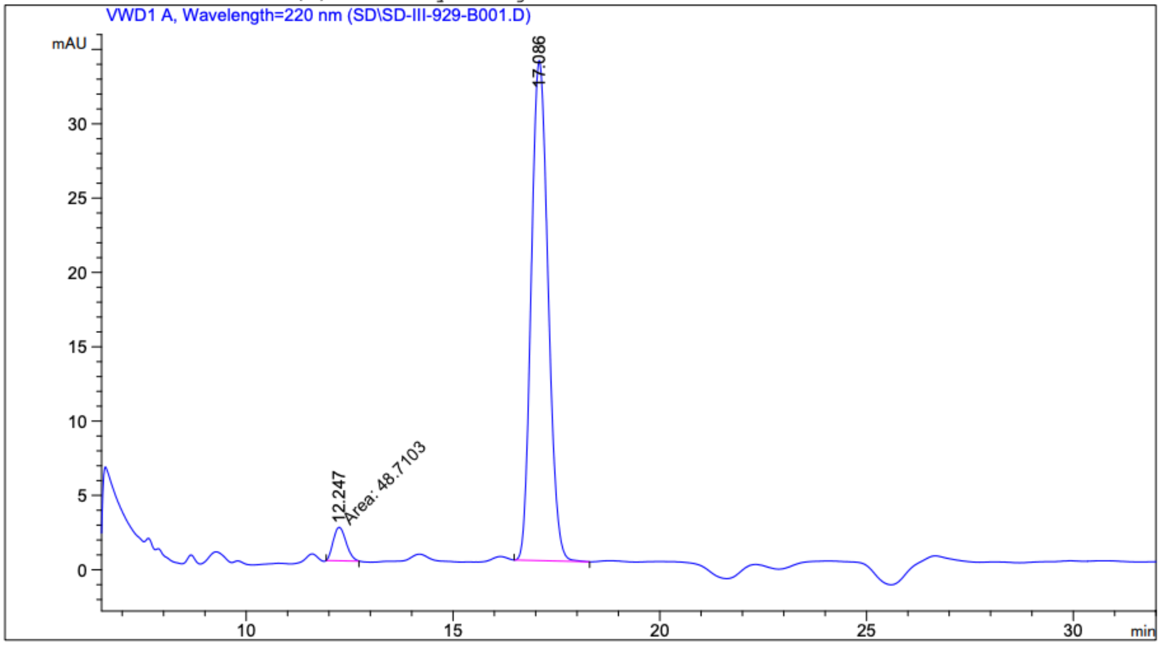


```
=====================================================================
                         Area Percent Report
=====================================================================

Sorted By             :      Signal
Multiplier:                  :      1.0000
Dilution:                    :      1.0000
Do not use Multiplier & Dilution Factor with ISTDs


Signal 1: VWD1 A, Wavelength=220 nm
```

Peak #	RetTime [min]	Type	Width [min]	Area [mAU*s]	Height [mAU]	Area %
1	12.247	MM	0.3606	48.71025	2.25123	4.8785
2	17.086	BB	0.4463	949.74744	33.64451	95.1215

Totals : 998.45769 35.89575

15. **References:**

[1] J. K. Park, H. H. Lackey, M. D. Rexford, K. Kovnir, M. Shatruk, D. T. Mcquade, A Chiral 6-membered N-heterocyclic Carbene Copper(i) Complex That Induces High Stereoselectivity. *Org. Lett.* **2010**, *12* (21), 5008–5011.

[2] C. M. Pearson, J. W. B. Fyfe, T. N. Snaddon, A Regio- and Stereodivergent Synthesis of Homoallylic Amines by a One-pot Cooperative-catalysis-based Allylic Alkylation/Hofmann Rearrangement Strategy. *Angew. Chem. Int. Ed.* **2019**, *58* (31), 10521–10527.

[3] S. Einaru, K. Shitamichi, T. Nagano, A. Matsumoto, K. Asano, S. Matsubara, trans-Cyclooctenes as Halolactonization Catalysts. *Angew. Chem. Int. Ed.* **2018**, *57,* 13863 –13867.

[4] D. Rozsar, A. J. M. Farley, I. Mclauchlan, B. D. A. Shennan, K. Yamazaki, D. J. Dixon, Bifunctional Iminophosphorane-catalyzed Enantioselective Nitroalkane Addition to Unactivated α,β-unsaturated Esters. *Angew. Chem. Int.l Ed.* **2023**, *62* (21).

[5] Kaga, A.; Hayashi, H.; Hakamata, H.; Oi, M.; Uchiyama, M.; Takita, R.; Chiba, S. Nucleophilic Amination of Methoxy Arenes Promoted by a Sodium Hydride/iodide Composite. *Angew. Chem. Int. Ed.* ***56*** (39), 11807–11811 (2017).

[6] S. Xing, C. Ma, W. Liu, S. Ni, D. Zhu, Xu, L., Shao, X. *Org. Lett.* ***25***, 1066−1071 (2023).

[7] Eli Lilly and Company *World Intellectual Property Organization*, WO9908699 A1 1999-02-25

[8] United States Dept. of Health and Human Services, United States, US6307090 B1 2001-10-23

[9] T. Jiang, T. Livinghouse, H. M. Lovick, On the Stereoselective Bicyclization of Aminodienes Catalyzed by Chelating Diamide Complexes of the Group 3 Metals. A Direct Comparison of Sc(iii) and Y(iii) Bis(amide)s with an Application to the Synthesis of Alkaloid 195F. *Chem. Commun.* **2011**, *47* (48), 12861.

[10] T. N. T. Nguyen, N. O. Thiell, F. Pape, J. F. Teichert, Copper(I)-Catalyzed Allylic Substitutions with a Hydride Nucleophile. *Org. Lett.* **2016**, *18*, 10, 2455–2458.

[11] R. Lui, Z. Yang, Y. Ni, K. Song, K. Shen, S. Lin, Q. Pan, Pd (II)/Bipyridine-Catalyzed Conjugate Addition of Arylboronic Acids to α,β-Unsaturated Carboxylic Acids. Synthesis of β-Quaternary Carbons Substituted Carboxylic Acids. *J. Org. Chem.* **2017**, *82*, 15, 8023–8030.

[12] M. Yoshida, H. Otaka, T. Doi, An Efficient Partial Reduction of α,β-unsaturated Esters Using DIBAL-H in Flow. *European J. Org. Chem.* **2014**, *27*, 6010–6016.

[13] F. Burg, T. Rovis, Diastereoselective Three-Component 3,4-Amino Oxygenation of 1,3-Dienes Catalyzed by a Cationic Heptamethylindenyl Rhodium (III) Complex. *J. Am. Chem. Soc.* **2021**, *143*, 43, 17964–17969.

[14] Syngenta Crop Protection AG World Intellectual Property Organization, WO2021009229 A1 2021-0121

[15] Esfam Biotech Pty Ltd.World Intellectual Property Organization, WO2022020888 A1 2022-02-03

[16] G. Qiu, M. Mamboury, Q. Wang, J. Zhu, Ketenimines from Isocyanides and Allyl Carbonates: Palladium-Catalyzed Synthesis of β,γ-Unsaturated Amides and Tetrazoles *Angew. Chem. Int. Ed.* **2016**, *55,* 15377–15381.

[17] O. V. Singh, H. Han, Iridium(I)-catalyzed regio- and enantioselective allylic amidation. *Tett. Lett.* **2007**, *48* (40), 7094–7098.

[18] S. A. Herbert, D. C. Castell, J. Clayden, G. E. Arnott, Manipulating the Diastereoselectivity of Ortholithiation in Planar Chiral Ferrocenes. *Org. Lett.* **2013**, *15*, 13, 3334–3337.

[19](a) C.J. Richards, T. Damalidis, D.E. Hibbs, M.B. Hursthouse, Synthesis of 2-[2-(diphenylphosphino)ferrocenyl]oxazoline Ligands. *Synlett*. **1995**, 74-76. (b) C.J. Richards, T. Damalidis, D.E. Hibbs, M.B. Hursthouse, *Tet. Lett.* **1995**, *36,* 3745-3748.
[20] N. Xu, H. Liang, J. P. Morken, Copper-Catalyzed Stereospecific Transformations of Alkylboronic Esters. *J. Am. Chem. Soc*. **2022**, *144,* 26, 11546–11552
[21] S. Das, M. A. Reilly, S. K. Dorn, A. M. Pearson, M. K. Brown, Approach Toward Stereoselective A-arylation by Pd/cu-catalyzed Arylboration of Electron Deficient Alkenes. *Angew. Chem. Int. Ed.* **2025**, *64*, e202424073.
[22] Y. Yamamoto, R. Fujikawa, T. Umemoto, N. Miyaura, Iridium-catalyzed Hydroboration of Alkenes with Pinacolborane. *Tetrahedron* **2004**, *60*, 10695–10700.
[23] S. Adak, P. S. Hazra, C. B. Fox, M. K. Brown, Boron Enabled Directed [2+2]- and Dearomative [4+2]-cycloadditions Initiated by Energy Transfer. *Angew. Chem. Int. Ed.* **2025**, *64*, e2024162.
[24] M-Y. Lyu, G. N. Morais, S. Chen, M. K. Brown, Ni-Catalyzed 1,1- and 1,3-Aminoboration of Unactivated Alkenes *J. Am. Chem. Soc.* **2023**,*145*, 50, 27254–27261.
[25] (a) M. P. Wiesenfeldt, J. A. Rossi-Ashton, I. B. Perry, J. Diesel, O. L. Garry, F. Bartels, S. C. Coote, X. Ma, C. S. Yeung, D. J. Bennett, D. W. C. Macmillan, General Access to Cubanes as Benzene Bioisosteres. *Nature*, **2023**, *618*, 7965, 513–518. (b) T. P. Le, S. Tanaka, M. Yoshimura, K. Sato, M. Kitamura, Stereodivergent Dehydrative Allylation of Β-keto Esters Using a Ru/pd Synergistic Catalyst. *Nat. Comm.* **2022**, *13,* 5876.
[26] C. Song, H-H. Zhang, S. Yu, Regio- and Enantioselective Decarboxylative Allylic Benzylation Enabled by Dual Palladium/Photoredox Catalysis. *ACS Catal.* **2022**, *12*, 2, 1428–1432.
[27] (a) Y. Chen, S. Cai, C. Wang, J. Cheng, S. Kramer, X. Sun, Asymmetric Total Syntheses of (−)-α-lycorane, (−)-zephyranthine, and Formal Synthesis of (+)-clivonine. *Chem. A. J.* **2017**, *12*, 12, 1309–1313. (b) J. Renault, C. Bouvry, J-F. Cupif, J-P. Hurvois, Alkylation of N,N-Dibenzylaminoacetonitrile: From Five- to SevenMembered Nitrogen-Containing Heterocyclic Systems. *J. Org. Chem.* **2023**, *88*, 6, 3582–3598.
[28] T-Y. Zhang, L-Y. Zhang, X. Liang, K. Wei, Y-R. Yang, Catalytic, Asymmetric Total Synthesis of (+)-α-, (+)-β-, (+)-γ-, and (−)-δ-Lycorane. *Org. Lett.* **2022**, *24,* 2905−2909.
